# Changes in and Recognition of Electrochemical Fingerprints of *Acer* spp. in Different Seasons

**DOI:** 10.3390/bios12121114

**Published:** 2022-12-02

**Authors:** Pengchong Zhang, Xiaolong Li, Yuhong Zheng, Li Fu

**Affiliations:** 1 Hangzhou Botanical Garden (Hangzhou West Lake Research Institute of Garden Science), Hangzhou 310013, China; 2 College of Materials and Environmental Engineering, Hangzhou Dianzi University, Hangzhou 310018, China; 3 Institute of Botany, Jiangsu Province and Chinese Academy of Sciences (Nanjing Botanical Garden, Mem. Sun Yat-Sen), Nanjing 210014, China

**Keywords:** *Acer* spp., electrochemical fingerprints, species identification, leaf extraction, electrochemical reaction

## Abstract

Electroanalytical chemistry is a metrological analysis technique that provides information feedback by measuring the voltammetric signal that changes when a molecule is involved in an electrochemical reaction. There is variability in the type and content of electrochemically active substances among different plants, and the signal differences presented by such differences in electrochemical reactions can be used for plant identification and physiological monitoring. This work used electroanalytical chemistry to monitor the growth of three *Acer* spp. This work explores the feasibility of the electrochemical analysis technique for the physiological monitoring of highly differentiated plants within the genus and further validates the technique. Changes in the electrochemical fingerprints of *A. cinnamomifolium*, *A. sinopurpurascens* and *A. palmatum* ‘Matsumurae’ were recorded during the one-year developmental cycle. The results show that the differences in the electrochemical fingerprint profiles of *Acer* spp. can be used to distinguish different species and identify the growth status in each season. This work also concludes with an identification flowchart based on electrochemical fingerprinting.

## 1. Introduction

The Aceraceae is the largest family in deciduous broadleaf and evergreen broadleaf forests. The genus *Acer* belongs to the Aceraceae and is a small deciduous or evergreen tree with more than 200 species, most of which are found in temperate regions of East Asia, eastern North America and Europe. Although *Acer* spp. are mainly distributed in temperate to subtropical regions, some species have extended their distribution into the tropics (e.g., Acer decandrum Merr in Hainan, China) [1]. The genus can be easily identified by opposite leaves and winged schizocarpic fruits (samaras), but other morphological characteristics are also highly diversified. Significant variations are observed in leaf shape. Even though the most common 3 or 5-lobed leaves are generally regarded as a characteristic of the genus, entire, 7–13 lobed, trifoliate, palmately, or pinnately compound leaves also occur [2]. In addition to the taxonomic difficulties in species delimitation, the phylogenetic schemes and intra-generic dispositions in the genus by different workers are very controversial.

Various phylogenetic studies have been carried out on the genome during the last two decades, significantly enhancing our understanding and resolving numerous evolutionary relationships among closely related species [3]. In the phylogenetic tree, the *Acer* spp. were primarily divided into three genetic branches under 10 different sections. As seeds and pollen are spread by wind and birds, interspecific hybridization and gene penetration occur widely in closely related species, leading to excessive intraspecific morphological variation, such as similar shapes of leaves, inflorescences, and winged fruits. Therefore, it is difficult to accurately identify and delineate maple species by the available morphological characters [4,5,6]. The use of electrochemical fingerprinting of plant tissues for species identification is an identification method that has emerged in recent years [7]. The principle of this technique is the diversity of electrochemically active components in different plant tissues [8,9,10,11,12]. This variation reflects, to some extent, genetic differences between species. The chemical composition of plant tissue can be affected by the environment in which it is grown in terms of the region, climate and soil. However, they are more limited by their genes. Our previous work also proved that the differences in electrochemically active molecules caused by plant species or variation were significantly greater than those caused by different environments [13]. However, the composition of plant tissue can also be affected by different growth stages. Therefore, collecting and comparing electrochemical fingerprints from different seasons is a challenge worthy of verification of the feasibility of this technology.

In our previous work [14], we studied 38 species of *Acer* spp. (including two outgroups) using this technique. Their interspecific relationships were explored based on statistical differences in electrochemical fingerprinting. In this work, we further monitored three *Acer* spp. over a long period of time. Changes in their fingerprint profiles were recorded. The differences in different fingerprint profiles can be used to achieve species and growing season identification.

## 2. Materials and Methods

### 2.1. Sample Collection

Information on the three *Acer* spp. used for physiological monitoring studies is shown in Table 1. Three species of *Acer* spp. leaves were collected at the Hangzhou Botanical Garden, two of which have a deciduous period, and the other has no deciduous period. The collection period is from July 2021 to July 2022, with half-monthly intervals. Due to the COVID-19 pandemic, only one collection was made in January, February, March, October and December, and three collections were made in July. All samples were kept frozen after adding colorimetric cards to take pictures before analysis.

### 2.2. Electrochemical Fingerprints Recording

**Figure 1:**
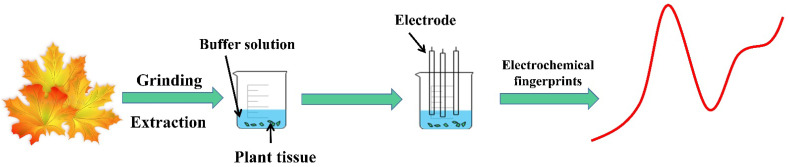
Schematic diagram of the electrochemical fingerprint recording of *Acer* spp.

Phosphate-buffered solution (0.1 M, PBS, pH 7.0) was prepared by mixing KH_2_PO_4_ and K_2_HPO_4_. Acetate-buffered solution (0.1 M, ABS, pH 4.5) was prepared by mixing CH_3_COONa and CH_3_COOH. An amount of 2 g of leaves was added to 2 mL of extraction solvent (water or ethanol). Then three grinding beads were added to a high-throughput tissue grinder, and the mixture was sonicated after grinding. After it was left to stand, 0.5 mL of the supernatant was put into a 10 mL beaker. Then, 2 mL of 0.1 M ABS buffer solution or 0.1 M PBS buffer solution was added. The addition of the extract does not alter the pH of the electrolyte because the buffer solution can maintain a buffer zone, stabilizing the pH of the whole system. Each plant sample was subjected to four different treatments: water as extraction solution with ABS buffer solution, water as extraction solution with PBS buffer solution, ethanol as extraction solution with ABS buffer solution, and ethanol as extraction solution with PBS buffer solution. A three-electrode system (a glassy carbon electrode Φ = 3 mm 28.26 mm^2^, a Pt wire and an Ag/AgCl electrode were used as working electrode, and a counter electrode and reference electrode, respectively) was inserted into the beaker for recording electrochemical fingerprints, and each condition was repeated three times. Differential pulse voltammetry (DPV) was used for scanning in the range of 0 to 1.3 V (pulse amplitude: 50 mV; pulse width: 0.05 s; pulse period: 0.5 s). Electrochemical fingerprint recordings were all performed at the CHI 760E electrochemical workstation. Each color of the DPV curve represents the collection of a species fingerprint. Data standardization was carried out to establish the quantitative criteria of recognition [15], where the ratios between the current and the maximum peak current were obtained at different potentials. Figure 1 represents a schematic diagram of the electrochemical fingerprint recording of maple trees. This study comprehensively analyzed the x-axis coordinates, peak ratios and peak area ratios of the electrochemical fingerprints of four conditions of three maples. These features were manually determined based on the electrochemical fingerprint information without using a computer-assisted decision tree.

## 3. Results and Discussion

### 3.1. Electrochemical Fingerprints of A. cinnamomifolium

For this experiment, we used commercially available glassy carbon electrodes. The choice of electrodes plays a very important role in electrochemical sensing. We have made a series of attempts at electrode modification [8,9,10,11,12]. The results indicated that the electrode modification of nanomaterials inevitably produces some disturbing signals. On the other hand, the specific adsorption of nanomaterials to different molecules in the plant can distort the ratio between signal and composition. Therefore, we decided to use the simplest glassy carbon electrode for electrochemical fingerprinting.

**Figure 2:**
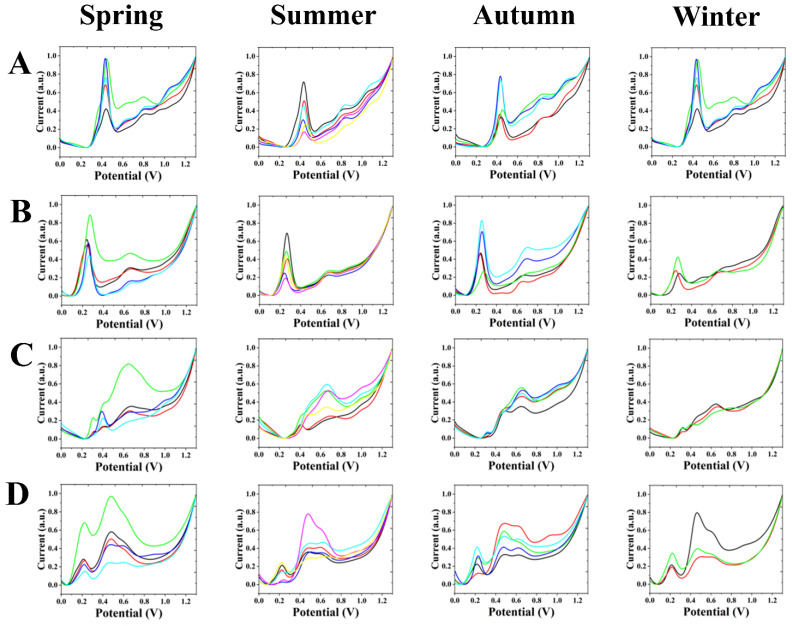
Electrochemical fingerprints of *A. cinnamomifolium* during different seasons under (**A**) water-ABS, (**B**) water-PBS, (**C**) ethanol-ABS and (**D**) ethanol-PBS conditions.

Figure 2A shows the electrochemical fingerprints of *A. cinnamomifolium* obtained during different seasons after extraction with water and under ABS conditions (each curve represents an individual sample). Significant oxidation peaks during all four seasons can be observed between 0.40 to 0.50 V and 0.80 to 1.00 V. There is a weak oxidation peak between 0.60 and 0.70 V in both the spring and winter.

Figure 2B shows the electrochemical fingerprints obtained after extraction with water and under PBS conditions for *A. cinnamomifolium* from different seasons. It can be observed that there are significant oxidation peaks during all four seasons between 0.20 and 0.30 V and between 0.60 and 0.70 V.

Figure 2C shows the electrochemical fingerprints obtained for *A. cinnamomifolium* during different seasons after extraction with ethanol and under ABS conditions. The electrochemical fingerprints from the spring had distinct oxidation peaks near 0.40 V, and between 0.60 and 0.70 V. A faint oxidation peak may be present near 0.30 V. The electrochemical fingerprints in the summer, autumn and winter had distinct oxidation peaks between 0.60 and 0.80 V. The oxidation peaks around 0.30 V become progressively more pronounced in the summer, autumn and winter, while they fade away around 0.40 V.

Figure 2D shows the electrochemical fingerprints obtained for different seasons of *A. cinnamomifolium* after extraction with ethanol and under PBS conditions. It can be found that the individual seasons have distinct oxidation peaks around 0.22 V and between 0.40 and 0.50 V. Some months showed a weak oxidation peak between 0.60 and 0.70 V.

### 3.2. Electrochemical Fingerprints of A. palmatum ‘Matsumurae’

**Figure 3:**
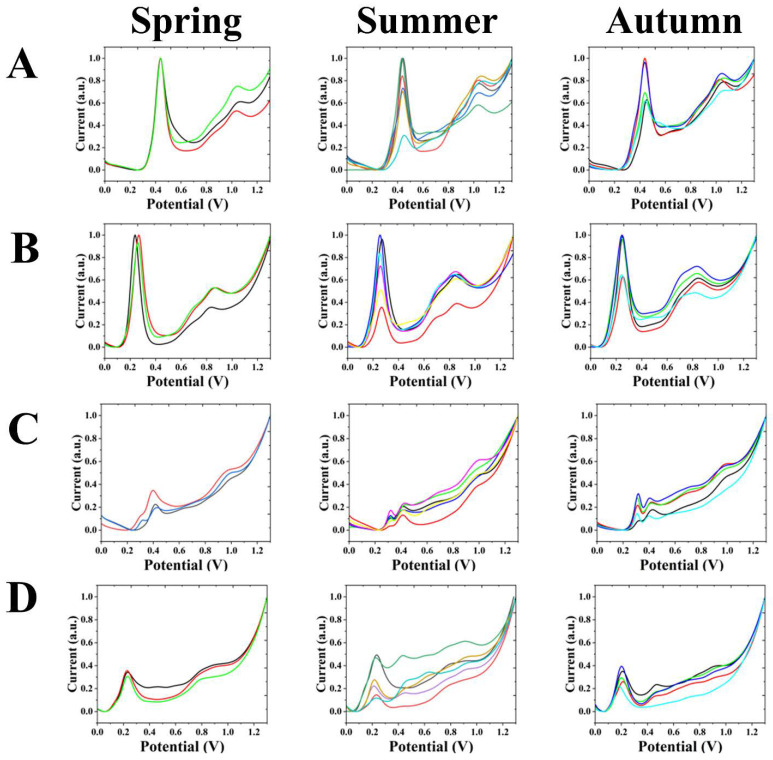
Electrochemical fingerprints of *A. palmatum* ‘Matsumurae’ in different seasons under (**A**) water-ABS, (**B**) water-PBS, (**C**) ethanol-ABS and (**D**) ethanol-PBS conditions.

Figure 3A shows the electrochemical fingerprints obtained after extraction with water and under ABS conditions for *A. palmatum* ‘Matsumurae’ from different seasons. It can be observed that the spring, summer and autumn have significant oxidation peaks between 0.40 to 0.50 V and 1.00 to 1.10 V.

Figure 3B shows the electrochemical fingerprints obtained after extraction with water and under PBS conditions for *A. palmatum* ‘Matsumurae’ from different seasons. It can be observed that spring, summer and autumn have significant oxidation peaks between 0.20 and 0.30 V and between 0.80 and 0.90 V.

Figure 3C shows the electrochemical fingerprints obtained from *A. palmatum* ‘Matsumurae’ in different seasons after extraction with ethanol and under ABS conditions. It can be noticed that the oxidation peaks are smaller in the spring, summer and autumn. A clear oxidation peak exists near 0.40 V in the spring. The oxidation peak near 0.30 V shows a gradual appearance compared to the summer and autumn. A clear oxidation peak was found near 0.30 V and near 0.40 V in the summer and autumn. At around 1.00 V, an outgoing peak trend exists in the spring, summer and autumn. In the mid-summer, some months do not show apparent peaks. The oxidation peak near 1.00 V shows a change with the season, showing a change from absent to present and then absent.

Figure 3D shows the electrochemical fingerprints obtained after extraction with ethanol and under PBS conditions for the *A. palmatum* ‘Matsumurae’ in different seasons. It can be noticed that the electrochemical fingerprints in the summer show a less stable appearance. There is a clear oxidation peak near 0.20 V in the spring, summer and autumn.

### 3.3. Electrochemical Fingerprints of A. sinopurpurascens

**Figure 4:**
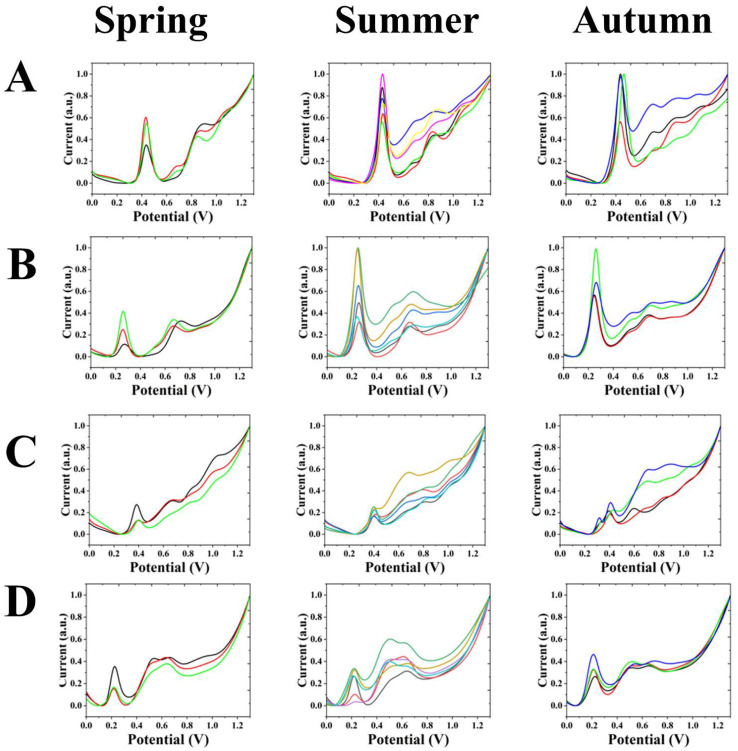
Electrochemical fingerprints of *A. sinopurpurascens* in different seasons under (**A**) water-ABS, (**B**) water-PBS, (**C**) ethanol-ABS and (**D**) ethanol-PBS conditions.

Figure 4A shows the electrochemical fingerprints obtained after extraction with water and under ABS conditions during different seasons for *A. sinopurpurascens*. A clear oxidation peak exists between 0.40 to 0.50 V and 0.80 to 1.00 V in the spring. Moreover, there is an outgoing peak between 0.60 and 0.70 V. Significant oxidation peaks were also present between 0.40 to 0.50 V and 0.80 to 1.00 V in the summer. Faint oxidation peaks exist between 0.60 to 0.70 V and 1.00 to 1.20 V.

Figure 4B shows the electrochemical fingerprints obtained after extraction with water and under PBS conditions for *A. sinopurpurascens* from different seasons. Significant oxidation peaks exist in the spring between 0.20 to 0.30 V and 0.60 to 0.80 V. The oxidation peaks are also evident in the summer between 0.20 to 0.30 V and 0.60 to 0.80 V. In the autumn; there are significant oxidation peaks between 0.20 and 0.30 V and between 0.60 and 0.80 V. There is also a tendency to show peaks between 0.40 and 0.60 V and around 0.90 V. Moreover, weak oxidation peaks already appear in some months.

Figure 4C shows the electrochemical fingerprints obtained after extraction with ethanol and under ABS conditions for different seasons of *A. sinopurpurascens*. Significant oxidation peaks were present near 0.40 V in the spring, summer and autumn. An oxidation peak exists between 0.60 and 0.80 V in the spring, and a few faint oxidation peaks are also present in this interval in autumn. A weak oxidation peak is present at around 0.80 V in the summer.

Figure 4D shows the electrochemical fingerprints obtained after extraction with ethanol and under PBS conditions for *A. sinopurpurascens* from different seasons. Significant oxidation peaks are present near 0.30 V and between 0.60 and 0.70 V in the spring, summer and autumn. Spring and summer tend to peak near 0.50 V, and a weak oxidation peak is already present in some curves. In the autumn, the oxidation peak in this interval is more stable.

### 3.4. Electrochemical Fingerprint Analysis

**Figure 5:**
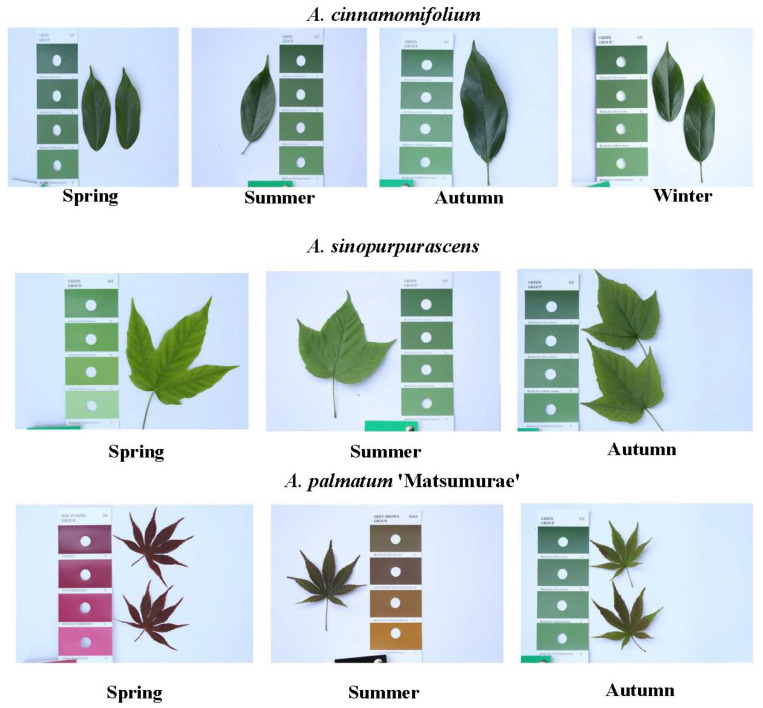
Digital photographs of *A. cinnamomifolium* in four seasons, and representative digital photographs of *A. sinopurpurascens* and *A. palmatum* ‘Matsumurae’ in the spring, summer and autumn.

Referring to the contents of existing seasonal divisions, March, April and May were classified as spring for data analysis. June, July and August are divided into summer months. September, October and November are classified as autumn. December, January and February are classified as winter. Figure 5 shows representative digital photographs of *A. cinnamomifolium* in four seasons, and representative digital photographs of *A. sinopurpurascens* and *A. palmatum* ‘Matsumurae’ in the spring, summer and autumn.

A series of oxidation peaks were observed in all seasons of *A. cinnamomifolium* after extraction with water and under ABS conditions. These peaks can be attributed to the oxidation of flavonols, phenolic acids, proanthocyanidins, alkaloids and pigments in plant tissues [16,17,18,19,20,21]. The molecules oxidized at lower potentials are usually small-molecular-weight phenols [22]. The oxidation peaks in this interval in the summer and autumn appear weaker than in spring and winter. A comparison of the electrochemical fingerprints of the four seasons reveals that after one year, the positions of the peaks of *A. cinnamomifolium* under water-ABS conditions did not change much. Throughout the year, the potential of the peak emergence of *A. cinnamomifolium* under water-PBS conditions changed slightly, and the greatest variation was in the size of the peak from season to season. This was caused by changes in the content of electrochemically active molecules in the leaves of *A. cinnamomifolium*, while the type of electrochemically active molecules did not change. Compared with the water-ABS condition, it can be observed that there are only two distinct oxidation peaks in the fingerprint profile under this condition. Moreover, the potentials of the peaks have all shifted to the left. It can be observed that the electrochemical profiles of *A. cinnamomifolium* under ethanol-ABS conditions appear to not be very stable in comparison to the water-ABS conditions. Moreover, a weak oxidation peak exists during this period in several of the summer and autumn months in the electrochemical profiles of *A. cinnamomifolium* under ethanol-PBS conditions. The overall comparison reveals that the oxidation peak between 0.60 and 0.70 V shows a phenomenon from absence to presence and then disappearance with the seasons. It can also be observed that the greatest variation is in the position of the oxidation peak in each season. Comparing these four conditions, it can be observed that there is no sudden appearance of oxidation peaks and no sudden disappearance of oxidation peaks. Under the same conditions, the potentials of the emergence peaks in all seasons were not very different; only the size of the peaks changed. Some peaks gradually disappear or appear with the change of seasons, which is closely related to the changes in the content of electrochemically active substances in the leaves of *A. cinnamomifolium*.

The electrochemical fingerprints of *A. palmatum* ‘Matsumurae’ obtained after extraction with water and under ABS conditions in the spring, summer and autumn were very similar, the only difference being the size of the peaks. In comparison with the same condition of *A. cinnamomifolium*, it can be observed that the location of the first peak of the two is not much different, but the location of the second peak is different. A leftward shift of the peak potential occurred compared to the water-ABS condition. By observing the electrochemical fingerprints in the summer and autumn, an oxidation peak near 0.70 V can be observed. Moreover, one electrochemical fingerprint collected in the autumn showed a good curve shape. The electrochemical fingerprints of *A. palmatum* ‘Matsumurae’ recorded after extraction with ethanol and under PBS conditions showed the spring has a tendency to peak between 0.80 and 1.00 V, and some months of summer and autumn have oxidation peaks in this interval. Most of the electrochemical curves in the summer and autumn have an oxidation peak between 0.40 and 0.50 V.

In the electrochemical fingerprints of *A. sinopurpurascens* obtained after extraction with water and under ABS conditions, the oxidation peaks between 0.40 to 0.50 V, 0.60 to 0.70 V and 1.00 to 1.20 V were all more pronounced in the autumn compared to the summer. In the summer electrochemical fingerprints of *A. sinopurpurascens* obtained after extraction with water and under PBS conditions, there is a tendency to show peaks between 0.40 and 0.60 V and around 0.90 V. Compared with the water-ABS condition, the electrochemical curves of this condition in the summer and autumn are similar to those of the water-ABS condition, except that the potential of the peak emergence shifts to the left. By comparing the electrochemical fingerprints of spring, summer and autumn, it can be observed that they are more different and not as stable as previously observed. The cause of this phenomenon may be the oxidation of certain electrochemically active substances in the air during the experiment.

From the comparison of the above electrochemical fingerprints, it can be found that the electrochemical curves of *A. cinnamomifolium* in the summer and autumn seasons of the same condition have a similarity. They both have obvious oxidation peaks near 0.20 V. *A. cinnamomifolium* had a distinct oxidation peak between 0.40 and 0.50 V, while *A. sinopurpurascens* had a distinct oxidation peak between 0.60 and 0.70 V. There is a tendency for *A. cinnamomifolium* to have peaks between 0.60 and 0.70 V, and some already have faint oxidation peaks. In contrast, there was a tendency for peaks around 0.50 V in the spring and summer in *A. sinopurpurascens*, and some of the fingerprints already had a weak oxidation peak. In the autumn, the oxidation peak in this interval is more stable.

**Figure 6:**
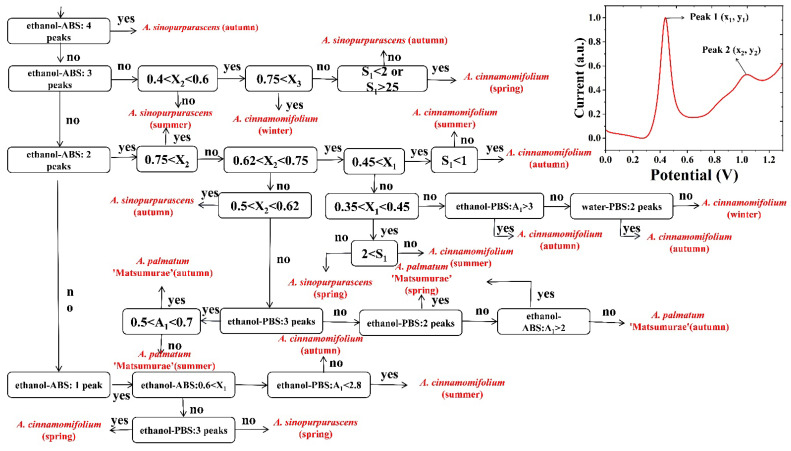
Flow chart of species and growing season identification of *A. sinopurpurascens, A. palmatum* ‘Matsumurae’ and *A. cinnamomifolium*.

**Figure 7:**
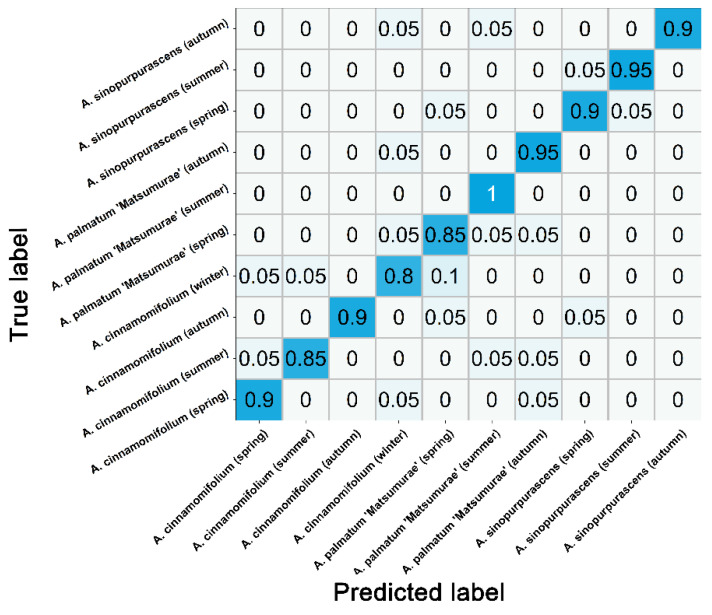
Confusion matrix of proposed flow chart.

In this study, the x-axis coordinates, peak ratios and peak area ratios of the electrochemical fingerprints of four conditions of three maples were comprehensively analyzed, and a flow chart for identifying *Acer* spp. and the growing seasons was proposed. An example of peaks in an electrochemical fingerprint is in the upper right corner of Figure 6. A_1_ denotes the ratio of the intensity of peak 2 to the intensity of peak 1. A_2_ denotes the ratio of the intensity of peak 3 to the intensity of peak 1. S_1_ denotes the ratio of the peak area of peak 2 to the peak area of peak 1. S_2_ denotes the ratio of the peak area of peak 3 to the peak area of peak 1. For example, the electrochemical fingerprint of species under ethanol-ABS conditions had two peaks, with peak 2 having x-axis coordinate values ranging from 0.62 to 0.75. The x-axis coordinate value of peak 1 was again after 0.45, and the ratio of the peak area of peak 2 to the peak area of peak 1 was less than 1. It can be identified that the species was *A. cinnamomifolium*, and the collection time was in autumn. The identification flowchart is a meaningful reference for future studies of highly differentiated plants within the genus. Figure 7 shows the confusion matrix of the proposed flowchart. The flowchart can successfully classify at least 80% of each species. The accuracy rate can reach 90%.

## 4. Conclusions

This study investigated the feasibility of applying electroanalytical chemical techniques to *Acer* spp. physiological monitoring and identification. Voltammetric signals of the reactions occurring in *Acer* spp. leaf extracts were obtained using an electroanalytical chemistry method combined with an electrochemical cell consisting of a three-electrode system. The voltammetric signals were processed to obtain electrochemical fingerprints. Physiological monitoring of three *Acer* spp. was realized based on electrochemical fingerprinting, as well as the identification of species and growing season determination. It was found that the collected electrochemical fingerprints were less stable, especially under ethanol-ABS conditions. When collecting the fingerprint curve of *A. cinnamomifolium*, there should be two oxidation peaks near 0.20 V and near 0.40 V. These two peaks alternate at times and appeared to be less stable. There are many reasons for these peaks, which are likely due to excessive exposure to air and the oxidation of electrochemically active molecules. It is also possible that the environment changed at that time, and coercive behavior was occurring. There have also been cases where the electrochemical profiles collected under the same conditions and from the same samples were completely different. Reducing the exposure time of the solution to be tested in the air resulted in a more stable electrochemical fingerprint. Finally, a flow chart for maple species and growing season identification was proposed by comparing the electrochemical fingerprints of each condition.

## Figures and Tables

**Table 1:** *Acer* spp. information for physiological monitoring studies.

No.	Botanical Name	Note
1	*Acer cinnamomifolium*	Evergreen
2	*Acer sinopurpurascens*	Deciduous
3	*Acer palmatum* ‘Matsumurae’	Deciduous

## Data Availability

The data presented in this study are available in Supplementary Materials.

## Supplementary Materials

The following supporting information can be downloaded at: https://www.mdpi.com/article/10.3390/bios12121114/s1, Table S1. Electrochemical fingerprints of *Acer cinnamomifolium*.
