# Supplementary material for: Changes in and Recognition of Electrochemical Fingerprints of Acer spp. in Different Seasons
Source: Biosensors (Basel). 2022 Dec 2;12(12):1114. doi: 10.3390/bios12121114 (PMC9775163; doi:10.3390/bios12121114)
Supplement: Supplementary file 1 [file biosensors-12-01114-s001.zip › biosensors-1989139-supplementary.pdf]

Raw table S1. Electrochemical fingerprints of *Acer cinnamomifolium*

Acer cinnamomifolium

Ethanol-ABS (Spring)					Ethanol-ABS (Summer)					Ethanol-ABS (Autumn)					Ethanol-ABS (Winter)			
0.004	0.09873	0.12495	0.10505	0.12345	0.004	0.12305	0.23208	0.23352	0.08894	0.004	0.17971	0.14933	0.1348	0.13105	0.004	0.06832	0.11458	0.09425
0.008	0.09385	0.11903	0.09868	0.11761	0.008	0.117	0.22319	0.22436	0.08341	0.008	0.17116	0.14233	0.12747	0.12393	0.008	0.06392	0.10858	0.08918
0.012	0.08965	0.11414	0.09348	0.11274	0.012	0.11235	0.21639	0.21638	0.0788	0.012	0.16397	0.1362	0.12131	0.11748	0.012	0.06003	0.10405	0.08521
0.016	0.08589	0.10985	0.08876	0.10836	0.016	0.10863	0.21007	0.20869	0.07514	0.016	0.15756	0.13098	0.116	0.11206	0.016	0.0568	0.09994	0.08198
0.02	0.08252	0.10618	0.08475	0.10471	0.02	0.10491	0.20468	0.20219	0.07171	0.02	0.15187	0.12614	0.11122	0.10742	0.02	0.05398	0.09642	0.07924
0.024	0.07959	0.10291	0.08121	0.10082	0.024	0.10212	0.2	0.19598	0.06877	0.024	0.1466	0.12189	0.10698	0.10278	0.024	0.05133	0.09328	0.07671
0.028	0.07693	0.09985	0.07814	0.0979	0.028	0.09933	0.19555	0.19066	0.0661	0.028	0.14233	0.11807	0.10337	0.09875	0.028	0.04909	0.09077	0.07461
0.032	0.07448	0.0972	0.07507	0.09498	0.032	0.09677	0.19133	0.18534	0.06367	0.032	0.13806	0.11458	0.09981	0.09514	0.032	0.0469	0.08826	0.07258
0.036	0.07211	0.09455	0.07224	0.0923	0.036	0.09467	0.18735	0.18031	0.06152	0.036	0.134	0.11103	0.09625	0.09143	0.036	0.04491	0.08589	0.07091
0.04	0.06995	0.0923	0.06917	0.08963	0.04	0.09235	0.18384	0.17558	0.05942	0.04	0.1303	0.10764	0.09317	0.08802	0.04	0.04296	0.0839	0.06928
0.044	0.06794	0.08985	0.06704	0.08744	0.044	0.09025	0.18033	0.17085	0.05743	0.044	0.12666	0.1043	0.09015	0.08482	0.044	0.04126	0.08188	0.06754
0.048	0.06581	0.08761	0.06468	0.085	0.048	0.08816	0.17681	0.16642	0.05564	0.048	0.12289	0.10134	0.08728	0.08178	0.048	0.03956	0.07972	0.06584
0.052	0.06395	0.08557	0.06232	0.08281	0.052	0.08607	0.1733	0.16199	0.05372	0.052	0.11962	0.09828	0.08463	0.07889	0.052	0.03795	0.07777	0.06424
0.056	0.06202	0.08332	0.0602	0.08038	0.056	0.08421	0.16956	0.15755	0.05189	0.056	0.11598	0.09527	0.08176	0.07579	0.056	0.03633	0.07568	0.06241
0.06	0.06025	0.08148	0.05807	0.07819	0.06	0.08258	0.16651	0.15341	0.05034	0.06	0.11307	0.09274	0.07953	0.07321	0.06	0.03501	0.07411	0.06087
0.064	0.05853	0.07944	0.05619	0.076	0.064	0.08072	0.163	0.14898	0.04863	0.064	0.11	0.08956	0.07687	0.07058	0.064	0.03347	0.07223	0.05911
0.068	0.05664	0.0772	0.05406	0.07381	0.068	0.07862	0.15948	0.14455	0.047	0.068	0.1068	0.08677	0.07438	0.06779	0.068	0.03207	0.07024	0.05697
0.072	0.0549	0.07536	0.05217	0.07162	0.072	0.07676	0.15574	0.14011	0.04549	0.072	0.10374	0.08386	0.0721	0.06527	0.072	0.0307	0.06843	0.05517
0.076	0.05312	0.07332	0.05028	0.06943	0.076	0.0749	0.15246	0.13627	0.04386	0.076	0.10053	0.08112	0.0696	0.06279	0.076	0.02933	0.06658	0.05307
0.08	0.05138	0.07128	0.04839	0.06748	0.08	0.07304	0.14871	0.13184	0.0425	0.08	0.09754	0.07848	0.06726	0.06031	0.08	0.02809	0.06456	0.05087
0.084	0.04975	0.06944	0.04651	0.06529	0.084	0.07095	0.14496	0.1277	0.04091	0.084	0.09455	0.07552	0.06493	0.05789	0.084	0.02664	0.0626	0.04911
0.088	0.048	0.0674	0.04485	0.06334	0.088	0.06909	0.14122	0.12356	0.03948	0.088	0.09149	0.073	0.0627	0.05531	0.088	0.02548	0.06062	0.04687
0.092	0.04647	0.06557	0.0432	0.0614	0.092	0.06699	0.13747	0.11942	0.03805	0.092	0.08879	0.07015	0.06058	0.05309	0.092	0.02436	0.0586	0.04484
0.096	0.04472	0.06353	0.04155	0.05921	0.096	0.06513	0.13372	0.11528	0.03649	0.096	0.08572	0.06772	0.0584	0.05092	0.096	0.02312	0.05657	0.04264
0.1	0.04295	0.06149	0.03966	0.05702	0.1	0.06327	0.12951	0.11114	0.03498	0.1	0.08259	0.06477	0.05601	0.0484	0.1	0.022	0.05455	0.04044
0.104	0.04138	0.05945	0.03824	0.05531	0.104	0.06141	0.12553	0.1073	0.03363	0.104	0.07981	0.06235	0.05383	0.04623	0.104	0.02076	0.05253	0.03821
0.108	0.03969	0.0574	0.03636	0.05337	0.108	0.05932	0.12178	0.10287	0.03212	0.108	0.07668	0.05971	0.05176	0.04396	0.108	0.01964	0.05044	0.03597
0.112	0.03798	0.05536	0.03494	0.05142	0.112	0.05722	0.11756	0.09902	0.0308	0.112	0.07398	0.05707	0.04953	0.04174	0.112	0.0186	0.04838	0.03384
0.116	0.03632	0.05353	0.03329	0.04923	0.116	0.05513	0.11358	0.09459	0.02937	0.116	0.0712	0.05487	0.04741	0.03968	0.116	0.01744	0.04636	0.03177
0.12	0.03463	0.05128	0.03163	0.04704	0.12	0.05327	0.10937	0.09075	0.02794	0.12	0.06821	0.05207	0.04518	0.03756	0.12	0.01632	0.04423	0.02967
0.124	0.03295	0.04924	0.03022	0.04534	0.124	0.05141	0.10515	0.08661	0.0267	0.124	0.06529	0.05008	0.04332	0.0355	0.124	0.01533	0.04214	0.02754
0.128	0.03129	0.04741	0.02856	0.04339	0.128	0.04931	0.10117	0.08277	0.02535	0.128	0.06244	0.04739	0.0413	0.03343	0.128	0.01421	0.04019	0.0256
0.132	0.02964	0.04516	0.02668	0.04144	0.132	0.04699	0.09672	0.07863	0.024	0.132	0.05974	0.04502	0.03907	0.03132	0.132	0.0133	0.03817	0.0236
0.136	0.02792	0.04332	0.02526	0.0395	0.136	0.04489	0.09251	0.07449	0.02256	0.136	0.05689	0.04271	0.03716	0.02946	0.136	0.01226	0.03618	0.0218
0.14	0.02641	0.04108	0.02361	0.03755	0.14	0.04303	0.08829	0.07065	0.02133	0.14	0.05433	0.04045	0.0353	0.0276	0.14	0.01135	0.03413	0.02004
0.144	0.02473	0.03904	0.02195	0.0356	0.144	0.04071	0.08407	0.06651	0.02006	0.144	0.05141	0.03814	0.03318	0.02564	0.144	0.01048	0.03193	0.01824
0.148	0.02307	0.03679	0.0203	0.03366	0.148	0.03861	0.07986	0.06267	0.01874	0.148	0.04877	0.03561	0.03116	0.02378	0.148	0.00949	0.02998	0.01657
0.152	0.02133	0.03475	0.01865	0.03171	0.152	0.03652	0.07564	0.05853	0.01735	0.152	0.04592	0.03362	0.02931	0.02203	0.152	0.00866	0.02792	0.01494
0.156	0.0197	0.03251	0.01723	0.02976	0.156	0.03466	0.07143	0.05469	0.01616	0.156	0.04322	0.03147	0.02745	0.02028	0.156	0.00779	0.02597	0.0133
0.16	0.01819	0.03047	0.01558	0.02757	0.16	0.03233	0.06745	0.05084	0.01492	0.16	0.04094	0.02937	0.02559	0.01857	0.16	0.00708	0.02405	0.0119
0.164	0.01653	0.02822	0.01369	0.02562	0.164	0.03024	0.06323	0.047	0.01361	0.164	0.03823	0.02727	0.02373	0.01692	0.164	0.00609	0.02213	0.01047
0.168	0.01491	0.02598	0.01204	0.02368	0.168	0.02791	0.05902	0.04316	0.0123	0.168	0.03567	0.02512	0.02187	0.01522	0.168	0.00534	0.02011	0.009
0.172	0.0134	0.02394	0.01039	0.02173	0.172	0.02582	0.05504	0.03961	0.01114	0.172	0.03318	0.02313	0.02007	0.01362	0.172	0.00468	0.01827	0.00773
0.176	0.01183	0.0219	0.00873	0.01954	0.176	0.02373	0.05035	0.03577	0.00991	0.176	0.03062	0.02109	0.01837	0.01207	0.176	0.00394	0.01631	0.00647
0.18	0.01023	0.01965	0.00755	0.01759	0.18	0.02163	0.04637	0.03222	0.00872	0.18	0.02827	0.01931	0.01651	0.01053	0.18	0.00319	0.0144	0.00533
0.184	0.00878	0.01761	0.0059	0.01565	0.184	0.01931	0.04239	0.02867	0.00752	0.184	0.02592	0.01732	0.01471	0.00918	0.184	0.00257	0.01258	0.0043
0.188	0.00742	0.01557	0.00472	0.0137	0.188	0.01745	0.03841	0.02572	0.00637	0.188	0.02392	0.01555	0.01327	0.00764	0.188	0.00203	0.01088	0.0034
0.192	0.00606	0.01353	0.00331	0.012	0.192	0.01559	0.03443	0.02247	0.00533	0.192	0.02164	0.01409	0.01179	0.0064	0.192	0.00145	0.0092	0.00247
0.196	0.00476	0.01169	0.00212	0.01029	0.196	0.01349	0.03044	0.01921	0.00434	0.196	0.01944	0.01221	0.00998	0.00511	0.196	0.00108	0.00756	0.0016
0.2	0.00373	0.00986	0.00118	0.00859	0.2	0.01163	0.02693	0.01626	0.00342	0.2	0.01744	0.0107	0.00855	0.00408	0.2	6.63E-04	0.00607	0.001
0.204	0.00266	0.00802	4.72E-04	0.00689	0.204	0.01	0.02342	0.0136	0.00259	0.204	0.01545	0.00941	0.00722	0.00304	0.204	3.73E-04	0.00474	6.00E-04
0.208	0.00198	0.00639	2.36E-04	0.00555	0.208	0.00814	0.02014	0.01123	0.00187	0.208	0.0136	0.00785	0.006	0.00212	0.208	2.07E-04	0.00356	3.00E-04
0.212	0.00112	0.00496	0	0.00416	0.212	0.00675	0.01686	0.00857	0.00107	0.212	0.01182	0.00662	0.00462	0.00139	0.212	8.29E-05	0.00237	0
0.216	5.03E-04	0.00353	2.36E-04	0.00297	0.216	0.00512	0.01382	0.0068	5.97E-04	0.216	0.01011	0.00538	0.00361	7.22E-04	0.216	0	0.00153	6.67E-05
0.22	2.07E-04	0.00249	7.08E-04	0.00202	0.22	0.00395	0.01101	0.00503	2.79E-04	0.22	0.00876	0.00425	0.0026	5.16E-04	0.22	2.49E-04	8.02E-04	3.33E-04
0.224	0	0.00135	0.00212	0.00129	0.224	0.00279	0.00867	0.00355	7.96E-05	0.224	0.00755	0.00328	0.0017	2.06E-04	0.224	4.56E-04	3.49E-04	8.67E-04
0.228	8.87E-05	6.53E-04	0.00378	6.08E-04	0.228	0.00163	0.00632	0.00207	0	0.228	0.00605	0.00247	0.00111	0	0.228	9.53E-04	0	0.00173
0.232	2.37E-04	1.22E-04	0.00661	1.95E-04	0.232	0.00116	0.00445	0.00118	2.79E-04	0.232	0.00506	0.00167	3.72E-04	1.55E-04	0.232	0.00157	1.05E-04	0.00267
0.236	7.39E-04	0	0.00968	0	0.236	4.65E-04	0.00281	5.91E-04	5.97E-04	0.236	0.00399	0.00118	1.06E-04	4.64E-04	0.236	0.00232	4.18E-04	0.00413
0.24	0.00169	1.02E-04	0.01393	2.68E-04	0.24	2.33E-04	0.00164	0	0.00115	0.24	0.00313	5.38E-04	0	8.77E-04	0.24	0.00344	0.00108	0.00607
0.244	0.00284	5.92E-04	0.01959	7.06E-04	0.244	0	7.03E-04	0	0.00199	0.244	0.00228	2.69E-04	5.31E-05	0.0017	0.244	0.00485	0.00202	0.0084
0.248	0.00441	0.00151	0.0262	0.00136	0.248	0	0	2.96E-04	0.00302	0.248	0.0015	2.15E-04	1.59E-04	0.00237	0.248	0.0065	0.00324	0.01103
0.252	0.00642	0.00296	0.03447	0.00243	0.252	4.65E-04	0	8.87E-04	0.0045	0.252	9.97E-04	0	6.90E-04	0.00356	0.252	0.00874	0.00498	0.0144
0.256	0.00878	0.00496	0.04415	0.00404	0.256	9.30E-04	2.34E-04	0.00207	0.00625	0.256	6.41E-04	1.61E-04	0.00159	0.0049	0.256	0.01127	0.00708	0.0183
0.26	0.01154	0.0072	0.05548	0.00577	0.26	0.00186	7.03E-04	0.00325	0.00812	0.26	2.14E-04	5.38E-04	0.0025	0.00635	0.26	0.01433	0.00962	0.02274
0.264	0.01494	0.01047	0.06893	0.00786	0.264	0.00302	0.00164	0.00532	0.01051	0.264	1.42E-04	0.00108	0.00425	0.0082	0.264	0.01819	0.01272	0.0282
0.268	0.01875	0.01394	0.08381	0.01054	0.268	0.00465	0.00328	0.00769	0.01309	0.268	0	0.00183	0.00605	0.01042	0.268	0.02262	0.01621	0.03407
0.272	0.02304	0.01843	0.1008	0.01346	0.272	0.00651	0.00492	0.01035	0.01584	0.272	3.56E-04	0.0028	0.00828	0.01285	0.272	0.02788	0.02029	0.04081
0.276	0.02783	0.02333	0.11898	0.01662	0.276	0.00837	0.00703	0.01301	0.01898	0.276	7.12E-04	0.00382	0.01078	0.01563	0.276	0.03393	0.02475	0.04824
0.28	0.03298	0.02924	0.13834	0.02003	0.28	0.01093	0.00937	0.01626	0.02229	0.28	0.00135	0.00495	0.01375	0.01904	0.28	0.04089	0.02977	0.05611
0.284	0.03851	0.03557	0.15793	0.02392	0.284	0.01326	0.01194	0.0198	0.02599	0.284	0.00221	0.00662	0.01736	0.02291	0.284	0.04876	0.03517	0.06468
0.288	0.0444	0.0423	0.17705	0.02781	0.288	0.01605	0.01475	0.02335	0.02965	0.288	0.00313	0.00828	0.02097	0.02719	0.288	0.05754	0.04071	0.07338
0.292	0.05031	0.04945	0.19452	0.03195	0.292	0.01907	0.01756	0.0269	0.03351	0.292	0.00449	0.01011	0.02495	0.03178	0.292	0.06666	0.04629	0.08201
0.296	0.05617	0.05679	0.20987	0.03609	0.296	0.02187	0.02037	0.03045	0.03737	0.296	0.00562	0.01194	0.0292	0.03679	0.296	0.07648	0.05194	0.09038
0.3	0.06173	0.06373	0.22144	0.04096	0.3	0.02489	0.02319	0.0337	0.04123	0.3	0.00733	0.01404	0.0335	0.042	0.3	0.0863	0.05724	0.09788
0.304	0.06646	0.07006	0.22852	0.04558	0.304	0.02791	0.026	0.03695	0.04505	0.304	0.0089	0.01614	0.03759	0.047	0.304	0.09558	0.06194	0.10421
0.308	0.07045	0.07557	0.23111	0.0502	0.308	0.03047	0.0281	0.03991	0.04863	0.308	0.01032	0.01807	0.04125	0.05154	0.308	0.10399	0.06588	0.10855
0.312	0.07326	0.07985	0.22946	0.05556	0.312	0.03303	0.03021	0.04227	0.05193	0.312	0.01139	0.02012	0.04428	0.05526	0.312	0.1107	0.06874	0.11121
0.316	0.0751	0.08271	0.22521	0.0614	0.316	0.03559	0.03279	0.04523	0.05508	0.316	0.01274	0.02232	0.04688	0.05799	0.316	0.11546	0.07069	0.11255
0.32	0.07589	0.08414	0.21884	0.06772	0.32	0.03815	0.03466	0.04789	0.05782	0.32	0.01396	0.02442	0.04863	0.05944	0.32	0.11786	0.0717	0.11188
0.324	0.07587	0.08434	0.21199	0.07478	0.324	0.04047	0.0363	0.05084	0.06033	0.324	0.01488	0.02684	0.04969	0.05949	0.324	0.11766	0.07195	0.11055
0.328	0.07542	0.08353	0.20609	0.08306	0.328	0.0428	0.03841	0.05439	0.06252	0.328	0.01566	0.0291	0.05033	0.05871	0.328	0.11542	0.07184	0.10888
0.332	0.07492	0.08251	0.20161	0.09255	0.332	0.04559	0.04052	0.05853	0.06447	0.332	0.01616	0.03174	0.05065	0.05711	0.332	0.11161	0.07121	0.10688
0.336	0.07465	0.08108	0.19924	0.1035	0.336	0.04862	0.04286	0.06355	0.06618	0.336	0.0168	0.0347	0.05081	0.05562	0.336	0.10668	0.07121	0.10555
0.34	0.07507	0.08006	0.19924	0.11615	0.34	0.05187	0.04567	0.06917	0.06761	0.34	0.01759	0.03792	0.05113	0.05412	0.34	0.10196	0.07121	0.10455
0.344	0.07622	0.07965	0.20231	0.13002	0.344	0.05583	0.04918	0.07597	0.06897	0.344	0.0188	0.04153	0.05176	0.05345	0.344	0.09744	0.07184	0.10455
0.348	0.0782	0.07985	0.20774	0.14584	0.348	0.06048	0.0534	0.08395	0.0704	0.348	0.02001	0.04567	0.05304	0.05376	0.348	0.09404	0.07292	0.10488
0.352	0.08092	0.08108	0.21577	0.16336	0.352	0.06583	0.05831	0.09282	0.07171	0.352	0.022	0.0503	0.05495	0.05505	0.352	0.09168	0.0748	0.10655
0.356	0.08468	0.08312	0.22592	0.18113	0.356	0.07188	0.0637	0.10257	0.07295	0.356	0.02442	0.05541	0.05755	0.05717	0.356	0.09069	0.07714	0.10888

0.36	0.08915	0.08618	0.23796	0.19986	0.36	0.07886	0.07049	0.11351	0.07454	0.36	0.02777	0.06089	0.06095	0.06067	0.36	0.09106	0.08028	0.11221
0.364	0.09453	0.09026	0.25189	0.21909	0.364	0.08653	0.07822	0.12563	0.07645	0.364	0.03161	0.06762	0.06525	0.06527	0.364	0.09288	0.0838	0.11622
0.368	0.10006	0.09475	0.26653	0.23734	0.368	0.09467	0.08595	0.13804	0.07832	0.368	0.03588	0.07407	0.07003	0.07068	0.368	0.09558	0.08802	0.12055
0.372	0.10568	0.09965	0.28163	0.25437	0.372	0.10305	0.09438	0.15046	0.08023	0.372	0.04087	0.08123	0.07555	0.07682	0.372	0.09926	0.09213	0.12555
0.376	0.1113	0.10475	0.29674	0.26922	0.376	0.11165	0.10304	0.16317	0.0825	0.376	0.04635	0.08903	0.0816	0.08379	0.376	0.10378	0.09677	0.13022
0.38	0.11653	0.10965	0.31161	0.28212	0.38	0.1198	0.11194	0.17588	0.08496	0.38	0.05247	0.09726	0.08824	0.09137	0.38	0.10925	0.10147	0.13522
0.384	0.12156	0.11454	0.32578	0.29161	0.384	0.1277	0.12014	0.18829	0.08759	0.384	0.05917	0.10613	0.09551	0.09927	0.384	0.11488	0.10628	0.13989
0.388	0.1257	0.11903	0.33876	0.29745	0.388	0.13468	0.12787	0.19982	0.09022	0.388	0.06629	0.11544	0.10321	0.10778	0.388	0.12081	0.11109	0.14422
0.392	0.12955	0.12291	0.3508	0.29988	0.392	0.14096	0.13466	0.21106	0.0932	0.392	0.07412	0.12528	0.11144	0.11686	0.392	0.12706	0.11601	0.14789
0.396	0.13251	0.12618	0.36143	0.29818	0.396	0.14585	0.14052	0.2214	0.09647	0.396	0.08259	0.13561	0.1202	0.12615	0.396	0.1334	0.12089	0.15122
0.4	0.13458	0.12862	0.3704	0.29355	0.4	0.14934	0.14496	0.23056	0.09981	0.4	0.09171	0.14669	0.12954	0.13616	0.4	0.13966	0.1257	0.15355
0.404	0.13605	0.13046	0.37771	0.28528	0.404	0.1512	0.14778	0.23855	0.10351	0.404	0.10132	0.15804	0.13973	0.14632	0.404	0.14604	0.13037	0.15522
0.408	0.13694	0.13128	0.38362	0.27506	0.408	0.15143	0.14871	0.24534	0.10745	0.408	0.11178	0.17015	0.15051	0.15695	0.408	0.15266	0.13483	0.15622
0.412	0.13753	0.13169	0.3881	0.26289	0.412	0.1505	0.14871	0.25126	0.11163	0.412	0.12289	0.18273	0.16192	0.16809	0.412	0.15913	0.13936	0.15655
0.416	0.13753	0.13128	0.39117	0.24951	0.416	0.14817	0.14684	0.25599	0.11593	0.416	0.13457	0.19521	0.17366	0.17939	0.416	0.16576	0.14354	0.15589
0.42	0.13753	0.13087	0.39353	0.23564	0.42	0.14468	0.14379	0.26012	0.12038	0.42	0.14674	0.20775	0.18587	0.19095	0.42	0.17243	0.14773	0.15489
0.424	0.13724	0.13005	0.39566	0.22225	0.424	0.14073	0.14028	0.26367	0.12504	0.424	0.15956	0.22017	0.19829	0.20266	0.424	0.17955	0.15191	0.15422
0.428	0.13724	0.12924	0.39754	0.20984	0.428	0.13608	0.1356	0.26663	0.12954	0.428	0.17266	0.23238	0.21077	0.21386	0.428	0.18647	0.15574	0.15289
0.432	0.13694	0.12862	0.39943	0.19816	0.432	0.13119	0.13068	0.26929	0.13419	0.432	0.1859	0.24373	0.22308	0.22444	0.432	0.19368	0.15888	0.15122
0.436	0.13694	0.12781	0.40109	0.1877	0.436	0.12584	0.12553	0.27165	0.13841	0.436	0.19858	0.25444	0.23455	0.23424	0.436	0.20043	0.16237	0.14989
0.44	0.13694	0.1272	0.40321	0.17845	0.44	0.12073	0.12061	0.27402	0.14263	0.44	0.21118	0.26401	0.24575	0.24306	0.44	0.20723	0.16516	0.14855
0.444	0.13753	0.1272	0.40628	0.17091	0.444	0.11607	0.11593	0.27727	0.14665	0.444	0.22342	0.27278	0.25605	0.25059	0.444	0.21348	0.16829	0.14789
0.448	0.13783	0.1272	0.41029	0.16458	0.448	0.11165	0.11171	0.28052	0.15043	0.448	0.23453	0.2809	0.2655	0.25694	0.448	0.21936	0.17108	0.14722
0.452	0.13872	0.12781	0.41478	0.15947	0.452	0.10747	0.10796	0.28407	0.15401	0.452	0.2445	0.28784	0.2741	0.26215	0.452	0.22434	0.17387	0.14689
0.456	0.1396	0.12862	0.42044	0.15606	0.456	0.10398	0.10515	0.2885	0.15775	0.456	0.25397	0.29462	0.28196	0.26654	0.456	0.22898	0.17631	0.14689
0.46	0.14108	0.13005	0.42682	0.15387	0.46	0.10119	0.10281	0.29353	0.16129	0.46	0.26223	0.3007	0.28918	0.27046	0.46	0.23308	0.17875	0.14755
0.464	0.14256	0.13169	0.4339	0.15241	0.464	0.09863	0.10141	0.29914	0.165	0.464	0.26978	0.30608	0.29592	0.27355	0.464	0.23672	0.18154	0.14789
0.468	0.14463	0.13413	0.44216	0.15217	0.468	0.09723	0.1007	0.30535	0.16866	0.468	0.27604	0.31092	0.30235	0.27716	0.468	0.24004	0.18433	0.14922
0.472	0.1467	0.13679	0.45042	0.15265	0.472	0.09607	0.10094	0.31156	0.1724	0.472	0.28124	0.31522	0.30835	0.28026	0.472	0.2429	0.18677	0.15055
0.476	0.14966	0.13985	0.4594	0.15411	0.476	0.09537	0.10141	0.31836	0.17626	0.476	0.28572	0.31899	0.31408	0.28387	0.476	0.24571	0.18956	0.15222
0.48	0.15262	0.14311	0.46931	0.15606	0.48	0.09514	0.10281	0.32545	0.18	0.48	0.28985	0.32222	0.31992	0.28748	0.48	0.24845	0.19235	0.15389
0.484	0.15587	0.14638	0.47946	0.15849	0.484	0.09537	0.10445	0.33195	0.1837	0.484	0.29263	0.32544	0.32523	0.29058	0.484	0.25093	0.19479	0.15589
0.488	0.15942	0.14985	0.49008	0.16141	0.488	0.09607	0.10703	0.33905	0.1876	0.488	0.29491	0.3276	0.33054	0.29419	0.488	0.25354	0.19792	0.15822
0.492	0.16327	0.15373	0.50142	0.16433	0.492	0.09723	0.10984	0.34614	0.19146	0.492	0.29626	0.32921	0.33532	0.2978	0.492	0.25636	0.20036	0.16055
0.496	0.16711	0.1576	0.51251	0.1675	0.496	0.09839	0.11265	0.35265	0.19524	0.496	0.29712	0.33082	0.34062	0.30141	0.496	0.2588	0.20315	0.16322
0.5	0.17155	0.16148	0.52384	0.17091	0.5	0.10002	0.11593	0.35885	0.19882	0.5	0.29733	0.33244	0.3454	0.30451	0.5	0.26129	0.20594	0.16589
0.504	0.17598	0.16536	0.53588	0.17431	0.504	0.10165	0.11967	0.36506	0.2024	0.504	0.29676	0.33297	0.34965	0.3076	0.504	0.26365	0.20908	0.16889
0.508	0.18072	0.16944	0.54839	0.17796	0.508	0.10375	0.12342	0.37097	0.2061	0.508	0.29605	0.33405	0.35496	0.3107	0.508	0.26614	0.21187	0.17189
0.512	0.18545	0.17352	0.56114	0.18137	0.512	0.10607	0.1274	0.37718	0.20969	0.512	0.29477	0.33513	0.35921	0.3138	0.512	0.26854	0.215	0.17522
0.516	0.19018	0.1774	0.57342	0.18502	0.516	0.10816	0.13068	0.38191	0.21299	0.516	0.29341	0.33566	0.36345	0.31689	0.516	0.27044	0.21779	0.17822
0.52	0.19521	0.18168	0.58687	0.18843	0.52	0.11072	0.13466	0.38723	0.21645	0.52	0.29199	0.33728	0.36823	0.31999	0.52	0.27293	0.22093	0.18189
0.524	0.20024	0.18556	0.60009	0.19208	0.524	0.11351	0.13817	0.39196	0.22007	0.524	0.29035	0.33889	0.37354	0.3236	0.524	0.27583	0.22407	0.18556
0.528	0.20526	0.18985	0.61308	0.19548	0.528	0.11631	0.14192	0.39669	0.22354	0.528	0.289	0.34051	0.37832	0.32721	0.528	0.27873	0.2272	0.18956
0.532	0.21029	0.19393	0.62606	0.19889	0.532	0.1191	0.14543	0.40083	0.22692	0.532	0.28779	0.34266	0.38363	0.33134	0.532	0.28122	0.23069	0.19356
0.536	0.21591	0.19801	0.63905	0.20205	0.536	0.12189	0.14871	0.40526	0.23038	0.536	0.28693	0.34481	0.38947	0.33547	0.536	0.28412	0.23452	0.19756
0.54	0.22124	0.20229	0.65227	0.20546	0.54	0.12491	0.15222	0.4094	0.23432	0.54	0.28672	0.34804	0.39531	0.34063	0.54	0.28702	0.23836	0.20189
0.544	0.22686	0.20658	0.66501	0.20862	0.544	0.12817	0.1555	0.41383	0.2379	0.544	0.28686	0.35126	0.40168	0.3463	0.544	0.29033	0.24254	0.20623
0.548	0.23248	0.21086	0.67682	0.21154	0.548	0.13119	0.15878	0.41738	0.24188	0.548	0.28693	0.35503	0.40805	0.35198	0.548	0.29323	0.24637	0.21023
0.552	0.23839	0.21515	0.68862	0.21471	0.552	0.13445	0.16206	0.42152	0.24586	0.552	0.28779	0.35933	0.41495	0.35817	0.552	0.29696	0.25056	0.21456
0.556	0.24431	0.21964	0.69972	0.21787	0.556	0.13771	0.16511	0.42595	0.24984	0.556	0.28921	0.36364	0.42291	0.36487	0.556	0.30027	0.25474	0.21923
0.56	0.25022	0.22393	0.70963	0.22128	0.56	0.14073	0.16838	0.4298	0.25422	0.56	0.29078	0.36848	0.43035	0.3721	0.56	0.304	0.25892	0.22323
0.564	0.25584	0.22801	0.71931	0.2242	0.564	0.14399	0.17119	0.43393	0.2586	0.564	0.29292	0.37386	0.43778	0.37932	0.564	0.30814	0.26346	0.22756
0.568	0.26176	0.23229	0.72828	0.22736	0.568	0.14748	0.174	0.43807	0.26297	0.568	0.29527	0.37924	0.44574	0.38706	0.568	0.31187	0.26799	0.23156
0.572	0.26738	0.23678	0.73702	0.23101	0.572	0.15073	0.17728	0.4431	0.26815	0.572	0.29833	0.38515	0.45371	0.39583	0.572	0.31643	0.27287	0.2359
0.576	0.27329	0.24127	0.74504	0.23418	0.576	0.15399	0.17986	0.44783	0.27292	0.576	0.30153	0.39053	0.46167	0.40409	0.576	0.32057	0.27775	0.2399
0.58	0.27862	0.24535	0.75165	0.23758	0.58	0.15701	0.18267	0.45226	0.2777	0.58	0.30452	0.39645	0.4691	0.41234	0.58	0.32513	0.28228	0.24357
0.584	0.28424	0.24964	0.75826	0.24148	0.584	0.16004	0.18548	0.45729	0.28247	0.584	0.30829	0.40237	0.4776	0.42111	0.584	0.32969	0.28716	0.24757
0.588	0.28956	0.25392	0.7644	0.24513	0.588	0.16329	0.18829	0.46231	0.28765	0.588	0.31178	0.40828	0.48503	0.42937	0.588	0.33424	0.29204	0.25123
0.592	0.29459	0.25821	0.77007	0.24902	0.592	0.16609	0.19133	0.46734	0.29282	0.592	0.31556	0.41366	0.49299	0.43814	0.592	0.3388	0.29657	0.25423
0.596	0.29991	0.26249	0.77502	0.25243	0.596	0.16911	0.19368	0.47266	0.29799	0.596	0.31912	0.41958	0.50042	0.44639	0.596	0.34294	0.30145	0.25757
0.6	0.30464	0.26637	0.77998	0.25608	0.6	0.1719	0.19649	0.47768	0.30317	0.6	0.32268	0.42496	0.50733	0.45465	0.6	0.3475	0.30598	0.26057
0.604	0.30938	0.27045	0.78494	0.25973	0.604	0.17469	0.19906	0.48271	0.30834	0.604	0.32624	0.4298	0.51423	0.4629	0.604	0.35164	0.31051	0.26357
0.608	0.31411	0.27453	0.78942	0.26338	0.608	0.17748	0.20211	0.48803	0.31351	0.608	0.32973	0.4341	0.5206	0.47064	0.608	0.35579	0.31504	0.26657
0.612	0.31825	0.27821	0.79367	0.26654	0.612	0.17981	0.20468	0.49276	0.31829	0.612	0.33293	0.43841	0.52697	0.47787	0.612	0.3591	0.31888	0.26924
0.616	0.32239	0.28168	0.79792	0.27019	0.616	0.18214	0.20749	0.49749	0.32307	0.616	0.33585	0.44271	0.53228	0.48561	0.616	0.36283	0.32271	0.27157
0.62	0.32653	0.28494	0.8017	0.27336	0.62	0.18469	0.2103	0.50192	0.32784	0.62	0.3387	0.44594	0.53759	0.4918	0.62	0.36614	0.32655	0.27424
0.624	0.33008	0.28821	0.80548	0.27628	0.624	0.18702	0.21311	0.50636	0.33222	0.624	0.34098	0.44863	0.54183	0.4985	0.624	0.36863	0.33003	0.2769
0.628	0.33333	0.29106	0.80831	0.2792	0.628	0.18911	0.21522	0.5099	0.3362	0.628	0.3429	0.45132	0.54555	0.50366	0.628	0.37112	0.33317	0.2789
0.632	0.33659	0.29372	0.81114	0.28163	0.632	0.19121	0.21803	0.51315	0.33978	0.632	0.34482	0.45347	0.54927	0.50882	0.632	0.37277	0.33596	0.28124
0.636	0.33984	0.29637	0.81374	0.28406	0.636	0.1933	0.22084	0.51641	0.34376	0.636	0.34639	0.45562	0.55245	0.51398	0.636	0.37443	0.33875	0.28324
0.64	0.3425	0.29861	0.81563	0.28601	0.64	0.19539	0.22342	0.51907	0.34655	0.64	0.3476	0.45777	0.55458	0.51811	0.64	0.37567	0.34119	0.28557
0.644	0.34516	0.30025	0.81681	0.28747	0.644	0.19702	0.22553	0.52084	0.34973	0.644	0.34831	0.45885	0.55617	0.52172	0.644	0.37609	0.34293	0.28724
0.648	0.34723	0.30188	0.81752	0.28917	0.648	0.19888	0.22787	0.52261	0.35212	0.648	0.34874	0.45992	0.55776	0.52482	0.648	0.37609	0.34433	0.28924
0.652	0.3493	0.3031	0.81775	0.29039	0.652	0.20074	0.23021	0.5238	0.35411	0.652	0.34902	0.46046	0.55829	0.5274	0.652	0.37567	0.34572	0.29091
0.656	0.35078	0.30392	0.81704	0.29112	0.656	0.20261	0.23255	0.52439	0.3561	0.656	0.34859	0.461	0.55829	0.52894	0.656	0.37484	0.34677	0.29257
0.66	0.35226	0.30453	0.81586	0.29161	0.66	0.20423	0.23443	0.52439	0.35769	0.66	0.34802	0.46046	0.55723	0.52998	0.66	0.3736	0.34711	0.29391
0.664	0.35345	0.30453	0.81398	0.29185	0.664	0.20563	0.2363	0.5238	0.35928	0.664	0.34724	0.45992	0.55617	0.53049	0.664	0.37153	0.34746	0.29557
0.668	0.35404	0.30412	0.81185	0.29209	0.668	0.20726	0.23794	0.52261	0.36008	0.668	0.34603	0.45939	0.55405	0.52998	0.668	0.36946	0.34746	0.29691
0.672	0.35492	0.30392	0.80949	0.29234	0.672	0.20889	0.23981	0.52143	0.36127	0.672	0.34475	0.45831	0.55192	0.52946	0.672	0.36697	0.34711	0.29857
0.676	0.35492	0.3027	0.80595	0.29185	0.676	0.21051	0.24075	0.51877	0.36127	0.676	0.34276	0.4567	0.54874	0.5274	0.676	0.36407	0.34642	0.29957
0.68	0.35463	0.30127	0.80241	0.29136	0.68	0.21191	0.24192	0.51611	0.36127	0.68	0.34069	0.45455	0.54449	0.52585	0.68	0.36117	0.34502	0.30124
0.684	0.35463	0.29984	0.79863	0.29088	0.684	0.21331	0.24286	0.51286	0.36087	0.684	0.33784	0.45293	0.54024	0.52275	0.684	0.35786	0.34398	0.30257
0.688	0.35374	0.298	0.79438	0.29039	0.688	0.21447	0.24356	0.50902	0.36047	0.688	0.33499	0.45024	0.53546	0.51966	0.688	0.35454	0.34223	0.30357
0.692	0.35315	0.29596	0.7899	0.2899	0.692	0.21563	0.24379	0.50458	0.35968	0.692	0.33186	0.44755	0.53016	0.51605	0.692	0.35123	0.34014	0.30491
0.696	0.35197	0.29372	0.78565	0.28917	0.696	0.21679	0.24403	0.49985	0.35848	0.696	0.32852	0.44432	0.52485	0.51192	0.696	0.3475	0.33805	0.30591
0.7	0.35108	0.29147	0.78116	0.28893	0.7	0.21819	0.24426	0.49512	0.35729	0.7	0.32517	0.44164	0.51901	0.50779	0.7	0.34419	0.33596	0.30757
0.704	0.3499	0.28902	0.77691	0.28844	0.704	0.21959	0.24403	0.4901	0.3557	0.704	0.32175	0.43895	0.51264	0.50315	0.704	0.34046	0.33352	0.30891
0.708	0.34871	0.28657	0.77195	0.2882	0.708	0.22052	0.24356	0.48448	0.35371	0.708	0.31776	0.43518	0.50626	0.4985	0.708	0.33756	0.33108	0.31024
0.712	0.34723	0.28453	0.76771	0.2882	0.712	0.22168	0.24356	0.47916	0.35172	0.712	0.31435	0.43195	0.49989	0.49386	0.712	0.33424	0.32864	0.31157
0.716	0.34605	0.28229	0.76322	0.2882	0.716	0.22308	0.24309	0.47384	0.34973	0.716	0.311	0.42873	0.49352	0.48922	0.716	0.33134	0.3262	0.31324
0.72	0.34487	0.28025	0.75873	0.2882	0.72	0.22424	0.24262	0.46822	0.34734	0.72	0.30744	0.42604	0.48768	0.48457	0.72	0.32886	0.32411	0.31491
0.724	0.34339	0.278	0.75401	0.2882	0.724	0.2254	0.24192	0.46261	0.34535	0.724	0.30381	0.42281	0.48131	0.47993	0.724	0.32596	0.32167	0.31624
0.728	0.34221	0.27617	0.74953	0.28844	0.728	0.22656	0.24122	0.45699	0.34296	0.728	0.30061	0.41958	0.47494	0.47529	0.728	0.32389	0.31923	0.31791
0.732	0.34132	0.27453	0.74504	0.28869	0.732	0.22796	0.24075	0.45167	0.34097	0.732	0.2974	0.41689	0.4691	0.47116	0.732	0.32182	0.31714	0.31958

0.736	0.34014	0.2729	0.74079	0.28893	0.736	0.22936	0.24005	0.44635	0.33898	0.736	0.29455	0.4142	0.46379	0.46755	0.736	0.32016	0.31504	0.32124
0.74	0.33925	0.27127	0.73584	0.28917	0.74	0.23052	0.23911	0.44103	0.3366	0.74	0.29142	0.41151	0.45795	0.46342	0.74	0.3185	0.31295	0.32224
0.744	0.33836	0.27004	0.73111	0.28917	0.744	0.23168	0.23841	0.436	0.33461	0.744	0.28907	0.40936	0.45264	0.45981	0.744	0.31726	0.31121	0.32424
0.748	0.33747	0.26902	0.72663	0.28966	0.748	0.23308	0.23747	0.43127	0.33301	0.748	0.28693	0.40721	0.4484	0.45723	0.748	0.31643	0.30947	0.32558
0.752	0.33688	0.2678	0.72144	0.28966	0.752	0.23424	0.23653	0.42625	0.33142	0.752	0.28487	0.40559	0.44362	0.45413	0.752	0.3156	0.30772	0.32658
0.756	0.336	0.26658	0.71601	0.28966	0.756	0.2354	0.23536	0.42152	0.32943	0.756	0.28288	0.40398	0.43937	0.45155	0.756	0.31519	0.30633	0.32758
0.76	0.3354	0.26556	0.71081	0.28966	0.76	0.23633	0.23443	0.41679	0.32784	0.76	0.2811	0.40237	0.43512	0.44949	0.76	0.31477	0.30494	0.32858
0.764	0.33481	0.26474	0.70515	0.29015	0.764	0.2375	0.23326	0.41236	0.32665	0.764	0.27953	0.40129	0.43194	0.44794	0.764	0.31477	0.30389	0.32991
0.768	0.33422	0.26392	0.69948	0.29015	0.768	0.23866	0.23208	0.40792	0.32545	0.768	0.27846	0.40075	0.42875	0.44639	0.768	0.31477	0.30284	0.33058
0.772	0.33333	0.2629	0.69311	0.29015	0.772	0.23936	0.23044	0.40319	0.32426	0.772	0.27732	0.40022	0.42557	0.44485	0.772	0.31477	0.30145	0.33124
0.776	0.33333	0.26249	0.68815	0.29112	0.776	0.24099	0.22974	0.39994	0.32426	0.776	0.27711	0.40075	0.42344	0.44485	0.776	0.3156	0.3011	0.33258
0.78	0.33274	0.26168	0.68178	0.29112	0.78	0.24168	0.22857	0.3958	0.32307	0.78	0.27654	0.40075	0.42185	0.44485	0.78	0.31643	0.30075	0.33291
0.784	0.33215	0.26086	0.6754	0.29161	0.784	0.24261	0.22693	0.39196	0.32267	0.784	0.27618	0.40129	0.41973	0.44433	0.784	0.31684	0.30006	0.33358
0.788	0.33156	0.26025	0.66879	0.29161	0.788	0.24355	0.22576	0.38812	0.32227	0.788	0.2759	0.40129	0.41814	0.44485	0.788	0.31767	0.29936	0.33391
0.792	0.33097	0.25923	0.66242	0.29185	0.792	0.24448	0.22412	0.38427	0.32147	0.792	0.27576	0.40183	0.41654	0.44536	0.792	0.31892	0.29866	0.33424
0.796	0.33038	0.25862	0.65604	0.29258	0.796	0.24517	0.22319	0.38102	0.32147	0.796	0.27611	0.4029	0.41601	0.44639	0.796	0.31974	0.29866	0.33458
0.8	0.32978	0.258	0.64967	0.29307	0.8	0.2461	0.22201	0.37777	0.32187	0.8	0.27654	0.40398	0.41495	0.44743	0.8	0.32099	0.29831	0.33491
0.804	0.3289	0.25719	0.64306	0.2938	0.804	0.24703	0.22084	0.37422	0.32187	0.804	0.27682	0.40506	0.41442	0.44846	0.804	0.32223	0.29796	0.33491
0.808	0.32831	0.25637	0.63669	0.29428	0.808	0.24796	0.21991	0.37127	0.32187	0.808	0.27782	0.40667	0.41442	0.45001	0.808	0.32347	0.29796	0.33524
0.812	0.32742	0.25576	0.63078	0.29526	0.812	0.2489	0.21897	0.36861	0.32227	0.812	0.27875	0.40775	0.41442	0.45155	0.812	0.32472	0.29831	0.33558
0.816	0.32683	0.25494	0.62465	0.29623	0.816	0.24983	0.21827	0.36624	0.32307	0.816	0.27989	0.40936	0.41442	0.45362	0.816	0.32637	0.29831	0.33524
0.82	0.32594	0.25433	0.61851	0.29672	0.82	0.25076	0.21756	0.36329	0.32346	0.82	0.28067	0.41097	0.41495	0.45516	0.82	0.32762	0.29831	0.33524
0.824	0.32476	0.25331	0.61213	0.29745	0.824	0.25122	0.21639	0.36063	0.32386	0.824	0.28152	0.41205	0.41495	0.45671	0.824	0.32886	0.29831	0.33491
0.828	0.32387	0.2527	0.60694	0.29866	0.828	0.25238	0.21616	0.35856	0.32506	0.828	0.28302	0.4142	0.41548	0.45878	0.828	0.33052	0.29866	0.33491
0.832	0.32298	0.25188	0.60127	0.29988	0.832	0.25331	0.21569	0.35678	0.32585	0.832	0.28423	0.41528	0.41654	0.46084	0.832	0.33176	0.29866	0.33491
0.836	0.32209	0.25107	0.59585	0.3011	0.836	0.25425	0.21546	0.35501	0.32705	0.836	0.2853	0.41743	0.41707	0.4629	0.836	0.33342	0.29901	0.33424
0.84	0.32121	0.25045	0.59089	0.30256	0.84	0.25541	0.21546	0.35353	0.32824	0.84	0.28672	0.41904	0.41814	0.46497	0.84	0.33466	0.29936	0.33458
0.844	0.32062	0.25005	0.58617	0.30402	0.844	0.2568	0.21546	0.35235	0.32943	0.844	0.28815	0.42119	0.4192	0.46703	0.844	0.33632	0.30006	0.33424
0.848	0.31973	0.24943	0.58121	0.30548	0.848	0.25773	0.21546	0.35176	0.33063	0.848	0.28964	0.42281	0.42079	0.46961	0.848	0.33756	0.3004	0.33391
0.852	0.31884	0.24882	0.57649	0.30694	0.852	0.2589	0.21593	0.35087	0.33222	0.852	0.29085	0.42442	0.42185	0.47167	0.852	0.33922	0.3011	0.33358
0.856	0.31825	0.24841	0.57224	0.30888	0.856	0.26029	0.21616	0.35028	0.33381	0.856	0.29242	0.42657	0.42344	0.47374	0.856	0.34046	0.3018	0.33358
0.86	0.31766	0.24821	0.56799	0.31059	0.86	0.26192	0.21663	0.35028	0.3354	0.86	0.29405	0.42819	0.42504	0.4758	0.86	0.3417	0.3025	0.33358
0.864	0.31707	0.24801	0.56398	0.31253	0.864	0.26332	0.21756	0.35058	0.33699	0.864	0.29583	0.43034	0.42663	0.47838	0.864	0.34377	0.30354	0.33358
0.868	0.31647	0.2476	0.55996	0.31424	0.868	0.26495	0.21827	0.35058	0.33898	0.868	0.29726	0.43195	0.42769	0.48045	0.868	0.34502	0.30424	0.33324
0.872	0.31618	0.2476	0.55642	0.31667	0.872	0.26681	0.2192	0.35117	0.34097	0.872	0.29904	0.4341	0.42982	0.48251	0.872	0.34667	0.30528	0.33358
0.876	0.31618	0.2478	0.55312	0.31911	0.876	0.26867	0.22061	0.35235	0.34296	0.876	0.30117	0.43679	0.43194	0.48561	0.876	0.34833	0.30668	0.33391
0.88	0.31588	0.2478	0.54981	0.3213	0.88	0.27053	0.22201	0.35353	0.34495	0.88	0.30274	0.43895	0.43406	0.48767	0.88	0.34999	0.30772	0.33391
0.884	0.31559	0.24801	0.54651	0.32349	0.884	0.27239	0.22319	0.35442	0.34734	0.884	0.30466	0.44164	0.43566	0.49025	0.884	0.35123	0.30912	0.33391
0.888	0.31588	0.24841	0.54344	0.32592	0.888	0.27472	0.22459	0.35619	0.34933	0.888	0.3068	0.44379	0.43778	0.49231	0.888	0.35289	0.31051	0.33458
0.892	0.31588	0.24903	0.5406	0.3286	0.892	0.27704	0.2267	0.35797	0.35172	0.892	0.30879	0.44701	0.44043	0.49489	0.892	0.35454	0.31191	0.33524
0.896	0.31647	0.24964	0.53801	0.33152	0.896	0.2796	0.22834	0.35974	0.3545	0.896	0.31086	0.4497	0.44256	0.49799	0.896	0.3562	0.31365	0.33591
0.9	0.31677	0.25025	0.53541	0.33419	0.9	0.28169	0.23044	0.36181	0.35689	0.9	0.31307	0.45239	0.44521	0.50005	0.9	0.35827	0.31539	0.33624
0.904	0.31736	0.25107	0.53329	0.33711	0.904	0.28448	0.23255	0.36417	0.35968	0.904	0.31534	0.45562	0.44787	0.50315	0.904	0.35993	0.31714	0.33724
0.908	0.31795	0.25209	0.53116	0.34028	0.908	0.28728	0.23513	0.36654	0.36246	0.908	0.31755	0.45885	0.45105	0.50624	0.908	0.36159	0.31923	0.33825
0.912	0.31884	0.25331	0.52904	0.34344	0.912	0.2903	0.23747	0.3692	0.36525	0.912	0.32004	0.46261	0.45371	0.50934	0.912	0.36366	0.32132	0.33958
0.916	0.31973	0.25433	0.52715	0.3466	0.916	0.29309	0.24005	0.37186	0.36804	0.916	0.32239	0.46584	0.45689	0.51192	0.916	0.36532	0.32341	0.34058
0.92	0.32091	0.25576	0.52526	0.35001	0.92	0.29612	0.24239	0.37482	0.37122	0.92	0.32467	0.46961	0.46008	0.51553	0.92	0.36739	0.32585	0.34191
0.924	0.32209	0.25739	0.52408	0.35342	0.924	0.2996	0.24567	0.37807	0.3744	0.924	0.32731	0.47283	0.46326	0.51863	0.924	0.36946	0.32829	0.34358
0.928	0.32328	0.25882	0.52266	0.35707	0.928	0.30286	0.24848	0.38132	0.37759	0.928	0.33001	0.4766	0.46645	0.52172	0.928	0.37153	0.33073	0.34491
0.932	0.32476	0.26045	0.52125	0.36048	0.932	0.30612	0.25152	0.38457	0.38077	0.932	0.3325	0.48037	0.46963	0.52482	0.932	0.3736	0.33317	0.34658
0.936	0.32653	0.26229	0.5203	0.36413	0.936	0.30984	0.2548	0.38812	0.38435	0.936	0.33521	0.48413	0.47282	0.52843	0.936	0.37567	0.33596	0.34858
0.94	0.32831	0.26433	0.51936	0.36826	0.94	0.31356	0.25808	0.39196	0.38793	0.94	0.33799	0.4879	0.47653	0.53204	0.94	0.37774	0.3391	0.35058
0.944	0.33008	0.26637	0.51865	0.37216	0.944	0.31752	0.26183	0.3958	0.39152	0.944	0.34055	0.49166	0.47972	0.53514	0.944	0.37982	0.34189	0.35291
0.948	0.33185	0.26841	0.51794	0.37605	0.948	0.321	0.26534	0.39935	0.3951	0.948	0.34311	0.49543	0.48344	0.53875	0.948	0.38189	0.34467	0.35491
0.952	0.33363	0.27066	0.51747	0.37994	0.952	0.32519	0.26885	0.40319	0.39908	0.952	0.34582	0.49919	0.48662	0.54236	0.952	0.38437	0.34781	0.35725
0.956	0.3357	0.2729	0.51723	0.38408	0.956	0.32915	0.2726	0.40733	0.40266	0.956	0.34852	0.50296	0.49034	0.54597	0.956	0.38686	0.35095	0.35991
0.96	0.33807	0.27535	0.517	0.38822	0.96	0.3331	0.27635	0.41117	0.40664	0.96	0.35137	0.50672	0.49352	0.54958	0.96	0.38893	0.35409	0.36258
0.964	0.34014	0.2778	0.51653	0.39235	0.964	0.33729	0.28009	0.41531	0.40982	0.964	0.354	0.50941	0.49671	0.55268	0.964	0.391	0.35722	0.36492
0.968	0.34221	0.28025	0.51676	0.39625	0.968	0.34171	0.28407	0.41915	0.4138	0.968	0.35707	0.51318	0.50042	0.55629	0.968	0.39349	0.36071	0.36758
0.972	0.34457	0.2829	0.51676	0.40014	0.972	0.34589	0.28806	0.423	0.41738	0.972	0.35963	0.51641	0.50361	0.55938	0.972	0.39639	0.36385	0.37058
0.976	0.34664	0.28535	0.51676	0.40379	0.976	0.35008	0.2918	0.42654	0.42136	0.976	0.36226	0.51963	0.5068	0.56248	0.976	0.39846	0.36698	0.37325
0.98	0.34901	0.28821	0.51723	0.4072	0.98	0.35427	0.29578	0.43039	0.42494	0.98	0.36525	0.52286	0.50998	0.56558	0.98	0.40094	0.37047	0.37592
0.984	0.35108	0.29066	0.51771	0.41061	0.984	0.35822	0.2993	0.43393	0.42813	0.984	0.36775	0.52555	0.51317	0.56919	0.984	0.40343	0.37326	0.37858
0.988	0.35315	0.2931	0.51794	0.41328	0.988	0.36218	0.30281	0.43719	0.43171	0.988	0.37031	0.52824	0.51582	0.57177	0.988	0.40592	0.37605	0.38125
0.992	0.35552	0.29576	0.51865	0.4162	0.992	0.36613	0.30632	0.44014	0.43529	0.992	0.37294	0.53093	0.51901	0.57486	0.992	0.40799	0.37918	0.38425
0.996	0.35729	0.2978	0.51865	0.41815	0.996	0.36962	0.30937	0.4428	0.43808	0.996	0.37544	0.53308	0.52113	0.57744	0.996	0.41006	0.38197	0.38658
1	0.35907	0.30025	0.51912	0.42034	1	0.37311	0.31241	0.44576	0.44166	1	0.37814	0.53577	0.52432	0.58002	1	0.41254	0.38476	0.38925
1.004	0.36143	0.3027	0.51983	0.42229	1.004	0.3766	0.31546	0.44812	0.44484	1.004	0.38092	0.53792	0.52644	0.5826	1.004	0.41462	0.38755	0.39192
1.008	0.36291	0.30494	0.5203	0.42399	1.008	0.37962	0.31803	0.45019	0.44803	1.008	0.38313	0.53954	0.52856	0.58467	1.008	0.41669	0.39034	0.39425
1.012	0.36469	0.30698	0.52077	0.42521	1.012	0.38265	0.32061	0.45226	0.45081	1.012	0.38555	0.54115	0.53069	0.58673	1.012	0.41876	0.39278	0.39659
1.016	0.36646	0.30923	0.52101	0.42667	1.016	0.38544	0.32319	0.45433	0.4536	1.016	0.38747	0.54276	0.53281	0.58879	1.016	0.42083	0.39557	0.39892
1.02	0.36823	0.31127	0.52172	0.42788	1.02	0.38846	0.32576	0.4561	0.45638	1.02	0.39003	0.54438	0.53493	0.59086	1.02	0.4229	0.39801	0.40125
1.024	0.37001	0.31351	0.52266	0.42934	1.024	0.39125	0.3281	0.45817	0.45917	1.024	0.39195	0.54599	0.53706	0.59241	1.024	0.42497	0.40079	0.40359
1.028	0.37149	0.31535	0.52266	0.43032	1.028	0.39358	0.33021	0.45936	0.46195	1.028	0.39409	0.54707	0.53865	0.59344	1.028	0.42663	0.40323	0.40559
1.032	0.37356	0.31759	0.52361	0.43202	1.032	0.3966	0.33279	0.46142	0.46514	1.032	0.39623	0.54868	0.54077	0.59499	1.032	0.4287	0.40602	0.40759
1.036	0.37533	0.31963	0.52432	0.43324	1.036	0.3994	0.33536	0.4632	0.46792	1.036	0.39836	0.54976	0.54183	0.59653	1.036	0.43036	0.40846	0.40992
1.04	0.3774	0.32188	0.52502	0.43494	1.04	0.40242	0.3377	0.46497	0.47071	1.04	0.4005	0.55083	0.54343	0.59756	1.04	0.43202	0.4109	0.41225
1.044	0.37918	0.32392	0.52573	0.4364	1.044	0.40521	0.34052	0.46645	0.4735	1.044	0.40335	0.55245	0.54502	0.5986	1.044	0.43367	0.41334	0.41426
1.048	0.38125	0.32637	0.52644	0.43835	1.048	0.40847	0.34333	0.46852	0.47668	1.048	0.40548	0.55352	0.54661	0.59963	1.048	0.43574	0.41613	0.41659
1.052	0.38332	0.32861	0.52762	0.44054	1.052	0.41149	0.34637	0.47059	0.47986	1.052	0.40762	0.55514	0.54821	0.60066	1.052	0.4374	0.41927	0.41926
1.056	0.38568	0.33127	0.5288	0.44273	1.056	0.41521	0.34965	0.47295	0.48305	1.056	0.40975	0.55675	0.5498	0.60118	1.056	0.43947	0.42206	0.42159
1.06	0.38805	0.33371	0.52951	0.44516	1.06	0.41847	0.35293	0.47502	0.48623	1.06	0.4126	0.55836	0.55139	0.60169	1.06	0.44113	0.42485	0.42426
1.064	0.39042	0.33637	0.53069	0.44784	1.064	0.42219	0.35644	0.47768	0.48941	1.064	0.41474	0.55998	0.55298	0.60272	1.064	0.4432	0.42833	0.42726
1.068	0.39337	0.33922	0.53211	0.451	1.068	0.42638	0.36019	0.48064	0.493	1.068	0.41759	0.56213	0.55511	0.60376	1.068	0.44527	0.43147	0.42992
1.072	0.39604	0.34229	0.53376	0.45416	1.072	0.43033	0.3644	0.48359	0.49658	1.072	0.42043	0.56428	0.55723	0.60427	1.072	0.44734	0.43496	0.43292
1.076	0.39899	0.34535	0.53517	0.45757	1.076	0.43475	0.36862	0.48655	0.50016	1.076	0.42328	0.56643	0.55882	0.6053	1.076	0.449	0.43844	0.43592
1.08	0.40195	0.34882	0.53706	0.46147	1.08	0.43917	0.3733	0.4898	0.50414	1.08	0.42613	0.56859	0.56095	0.60582	1.08	0.45149	0.44228	0.43926
1.084	0.4058	0.35269	0.53942	0.46609	1.084	0.44429	0.37822	0.49394	0.50891	1.084	0.4304	0.57181	0.56413	0.60788	1.084	0.45397	0.44611	0.44293
1.088	0.40935	0.35657	0.54155	0.47023	1.088	0.44917	0.38337	0.49778	0.51289	1.088	0.43325	0.5745	0.56679	0.60892	1.088	0.45646	0.45064	0.44693
1.092	0.4129	0.36065	0.54344	0.47461	1.092	0.45429	0.38852	0.50133	0.51727	1.092	0.43681	0.57719	0.56891	0.60995	1.092	0.45894	0.45448	0.45059
1.096	0.41704	0.36494	0.54603	0.47947	1.096	0.45987	0.39415	0.50576	0.52205	1.096	0.44037	0.58042	0.5721	0.6115	1.096	0.46226	0.45936	0.45493
1.1	0.42118	0.36943	0.54863	0.48458	1.1	0.46569	0.4	0.5102	0.52682	1.1	0.44464	0.58365	0.57475	0.61304	1.1	0.46516	0.46389	0.45926
1.104	0.42561	0.37412	0.55146	0.48994	1.104	0.47151	0.40609	0.51493	0.532	1.104	0.44891	0.58687	0.57794	0.61511	1.104	0.46847	0.46877	0.4636
1.108	0.43035	0.37902	0.5543	0.49529	1.108	0.47755	0.41265	0.51966	0.53677	1.108	0.45319	0.5901	0.58112	0.61665	1.108	0.47179	0.474	0.46826

1.112	0.43537	0.38412	0.55737	0.50137	1.112	0.4843	0.41897	0.52468	0.54195	1.112	0.45746	0.59387	0.58484	0.61872	1.112	0.4751	0.47922	0.47326
1.116	0.4407	0.38963	0.56091	0.50746	1.116	0.49081	0.42623	0.5303	0.54752	1.116	0.46173	0.59817	0.58908	0.62078	1.116	0.47924	0.4848	0.4786
1.12	0.44632	0.39534	0.56445	0.51379	1.12	0.49756	0.43349	0.53591	0.55349	1.12	0.46671	0.60194	0.5928	0.62388	1.12	0.48297	0.49038	0.48393
1.124	0.45194	0.40126	0.56799	0.52011	1.124	0.50454	0.44052	0.54153	0.55906	1.124	0.4717	0.60624	0.59652	0.62594	1.124	0.48712	0.49596	0.48927
1.128	0.45785	0.40738	0.572	0.52693	1.128	0.51175	0.44848	0.54774	0.56543	1.128	0.47739	0.61054	0.6013	0.62904	1.128	0.49167	0.50223	0.49527
1.132	0.46436	0.41391	0.57625	0.53423	1.132	0.51942	0.45644	0.55365	0.57179	1.132	0.48238	0.61538	0.60554	0.63265	1.132	0.49623	0.50816	0.50127
1.136	0.47057	0.42085	0.58074	0.54153	1.136	0.5271	0.4644	0.56015	0.57776	1.136	0.48807	0.62023	0.61032	0.63574	1.136	0.5012	0.51478	0.50727
1.14	0.47737	0.42759	0.58499	0.54858	1.14	0.53501	0.47307	0.56666	0.58413	1.14	0.49377	0.62507	0.6151	0.63884	1.14	0.50659	0.5214	0.5136
1.144	0.48447	0.43473	0.59018	0.55613	1.144	0.54292	0.48126	0.57346	0.59089	1.144	0.50018	0.62991	0.61988	0.64297	1.144	0.51156	0.52803	0.51994
1.148	0.49157	0.44269	0.59537	0.56416	1.148	0.55129	0.49087	0.58085	0.59766	1.148	0.50659	0.63583	0.62519	0.6471	1.148	0.51736	0.53535	0.52694
1.152	0.49926	0.45065	0.6008	0.57219	1.152	0.56013	0.5	0.58794	0.60443	1.152	0.51299	0.6412	0.63103	0.65122	1.152	0.52357	0.54267	0.53427
1.156	0.50665	0.4586	0.606	0.57998	1.156	0.5685	0.50937	0.59503	0.61119	1.156	0.52011	0.64658	0.6358	0.65535	1.156	0.52937	0.54999	0.54161
1.16	0.51494	0.46738	0.61213	0.58849	1.16	0.57758	0.5192	0.60272	0.61875	1.16	0.52723	0.6525	0.64217	0.66051	1.16	0.536	0.558	0.54894
1.164	0.52351	0.47615	0.61804	0.59726	1.164	0.58642	0.52927	0.611	0.62592	1.164	0.53435	0.65842	0.64801	0.66515	1.164	0.54304	0.56602	0.55694
1.168	0.53209	0.48513	0.62441	0.60577	1.168	0.59572	0.53981	0.61868	0.63348	1.168	0.54219	0.66487	0.65439	0.67031	1.168	0.55009	0.57439	0.56461
1.172	0.54096	0.49452	0.63126	0.61453	1.172	0.60526	0.54988	0.62725	0.64104	1.172	0.55002	0.67133	0.66023	0.67547	1.172	0.55713	0.58275	0.57261
1.176	0.54984	0.50432	0.63763	0.62354	1.176	0.61479	0.56066	0.63583	0.6486	1.176	0.55785	0.67725	0.6666	0.68115	1.176	0.565	0.59112	0.58094
1.18	0.5593	0.51432	0.64471	0.63278	1.18	0.6248	0.5719	0.6441	0.65656	1.18	0.56639	0.68424	0.6735	0.68682	1.18	0.57287	0.60018	0.58961
1.184	0.56965	0.52554	0.65274	0.64276	1.184	0.6355	0.58384	0.65415	0.66531	1.184	0.57565	0.69177	0.68146	0.69353	1.184	0.58157	0.60994	0.59928
1.188	0.57941	0.53574	0.66006	0.65177	1.188	0.64527	0.59508	0.66273	0.67367	1.188	0.58491	0.69876	0.68836	0.69921	1.188	0.58986	0.619	0.60828
1.192	0.58977	0.54697	0.66808	0.66174	1.192	0.6555	0.60703	0.67218	0.68203	1.192	0.59416	0.70629	0.6958	0.70591	1.192	0.59897	0.62876	0.61795
1.196	0.60041	0.55819	0.67635	0.67148	1.196	0.6662	0.61897	0.68164	0.69118	1.196	0.60413	0.71382	0.70376	0.71262	1.196	0.60809	0.63852	0.62762
1.2	0.61106	0.56982	0.68437	0.68121	1.2	0.67667	0.63115	0.6914	0.69994	1.2	0.6141	0.72136	0.71172	0.71933	1.2	0.61762	0.64863	0.63762
1.204	0.62201	0.58186	0.69263	0.69119	1.204	0.68714	0.64333	0.70115	0.70869	1.204	0.62478	0.72942	0.71969	0.72655	1.204	0.62714	0.65909	0.64729
1.208	0.63324	0.59411	0.70137	0.70141	1.208	0.69807	0.65597	0.7115	0.71784	1.208	0.63546	0.73749	0.72765	0.73377	1.208	0.6375	0.6692	0.65762
1.212	0.64478	0.60717	0.71058	0.71187	1.212	0.70947	0.66909	0.72155	0.72779	1.212	0.64685	0.7461	0.73614	0.74151	1.212	0.64827	0.68035	0.66862
1.216	0.65691	0.62043	0.72025	0.72258	1.216	0.7211	0.68244	0.73189	0.73734	1.216	0.65753	0.75471	0.74517	0.74977	1.216	0.65946	0.69151	0.67962
1.22	0.66903	0.6339	0.72946	0.73329	1.22	0.73203	0.69508	0.74224	0.7469	1.22	0.66892	0.76331	0.75366	0.75802	1.22	0.67064	0.70231	0.69063
1.224	0.68205	0.64798	0.73961	0.74448	1.224	0.74366	0.7089	0.75347	0.75684	1.224	0.68103	0.773	0.76322	0.76679	1.224	0.68266	0.71417	0.70263
1.228	0.69536	0.66267	0.75	0.75592	1.228	0.75529	0.72248	0.76471	0.76719	1.228	0.69384	0.78214	0.77278	0.77608	1.228	0.69509	0.72637	0.71496
1.232	0.70896	0.67757	0.76086	0.76736	1.232	0.76739	0.7363	0.77564	0.77794	1.232	0.70666	0.79129	0.78233	0.78537	1.232	0.70793	0.73857	0.7273
1.236	0.72227	0.69226	0.77148	0.77855	1.236	0.77902	0.74965	0.78658	0.78868	1.236	0.72019	0.80097	0.79189	0.79465	1.236	0.72077	0.75042	0.73997
1.24	0.73647	0.70777	0.78258	0.79023	1.24	0.79088	0.76347	0.79811	0.79982	1.24	0.733	0.81065	0.80197	0.80446	1.24	0.73444	0.76332	0.75297
1.244	0.75126	0.72389	0.79462	0.80264	1.244	0.80368	0.77799	0.81023	0.81097	1.244	0.74795	0.82141	0.81259	0.81478	1.244	0.74894	0.77621	0.76664
1.248	0.76634	0.74063	0.80689	0.8153	1.248	0.81624	0.79227	0.82235	0.82291	1.248	0.76219	0.83217	0.82374	0.82561	1.248	0.76386	0.78946	0.78064
1.252	0.78143	0.75736	0.81893	0.82746	1.252	0.8288	0.80656	0.83388	0.83445	1.252	0.77714	0.84239	0.83436	0.83645	1.252	0.77836	0.80305	0.79464
1.256	0.79769	0.77491	0.83215	0.84036	1.256	0.84182	0.82131	0.84629	0.84639	1.256	0.79352	0.85368	0.84657	0.84831	1.256	0.7941	0.81735	0.80964
1.26	0.81396	0.79307	0.84537	0.85375	1.26	0.85532	0.83677	0.859	0.85912	1.26	0.80918	0.86498	0.85825	0.85966	1.26	0.81026	0.83233	0.82498
1.264	0.83082	0.81164	0.85883	0.86689	1.264	0.86834	0.85176	0.87171	0.87186	1.264	0.82627	0.87682	0.87046	0.87205	1.264	0.82683	0.84732	0.84065
1.268	0.84738	0.83042	0.87276	0.87978	1.268	0.88207	0.86674	0.88472	0.88459	1.268	0.84265	0.88919	0.8832	0.88494	1.268	0.84381	0.86266	0.85665
1.272	0.86483	0.84981	0.88716	0.8939	1.272	0.89579	0.88244	0.89802	0.89812	1.272	0.86045	0.90102	0.89647	0.89733	1.272	0.86121	0.87835	0.87298
1.276	0.88258	0.8698	0.90203	0.90826	1.276	0.90975	0.89836	0.91221	0.91165	1.276	0.87896	0.91447	0.91028	0.91074	1.276	0.87986	0.89438	0.88999
1.28	0.90121	0.89001	0.91737	0.9231	1.28	0.92417	0.91452	0.92581	0.92598	1.28	0.89818	0.92792	0.92408	0.92467	1.28	0.89891	0.91111	0.90732
1.284	0.91985	0.91082	0.93272	0.93722	1.284	0.93836	0.93044	0.9397	0.93951	1.284	0.91741	0.94137	0.93842	0.9386	1.284	0.91756	0.92784	0.92466
1.288	0.93966	0.93286	0.94948	0.95303	1.288	0.95394	0.94778	0.95507	0.95423	1.288	0.93806	0.95589	0.95328	0.95357	1.288	0.93786	0.94562	0.94299
1.292	0.95978	0.9549	0.96624	0.96836	1.292	0.96906	0.96487	0.96985	0.96936	1.292	0.9587	0.97095	0.96815	0.96904	1.292	0.95816	0.9634	0.96199
1.296	0.97989	0.97714	0.983	0.98394	1.296	0.98465	0.9822	0.98463	0.98448	1.296	0.97935	0.98548	0.98354	0.98401	1.296	0.97887	0.98153	0.981
1.3	1	1	1	1	1.3	1	1	1	1	1.3	1	1	1	1	1.3	1	1	1

Ethanol-PBS (Spring)					Ethanol-PBS (Summer)					Ethanol-PBS (Autumn)					Ethanol-PBS (Winter)			
0.004	0.02761	0.0374	0.02817	0.04883	0.004	0.03875	0.08522	0.03992	0.07913	0.004	0.05208	0.04861	0.04559	0.1454	0.004	0.07537	0.037	0.039
0.008	0.02284	0.03227	0.02326	0.0427	0.008	0.03382	0.07709	0.03475	0.07184	0.008	0.04509	0.04386	0.03926	0.131	0.008	0.06594	0.03244	0.03298
0.012	0.01851	0.02792	0.01836	0.03681	0.012	0.02987	0.06946	0.02963	0.06556	0.012	0.03888	0.03934	0.03363	0.11797	0.012	0.0578	0.02818	0.02777
0.016	0.01489	0.02371	0.01467	0.03148	0.016	0.02586	0.06234	0.02519	0.05964	0.016	0.0339	0.03527	0.02858	0.107	0.016	0.05065	0.0243	0.02325
0.02	0.01166	0.01985	0.01071	0.02705	0.02	0.0225	0.05624	0.02075	0.05444	0.02	0.02924	0.03127	0.0243	0.09671	0.02	0.04425	0.02114	0.01907
0.024	0.00872	0.01642	0.00756	0.02303	0.024	0.01931	0.05014	0.0169	0.04924	0.024	0.02419	0.02764	0.02052	0.08711	0.024	0.03828	0.01818	0.0154
0.028	0.00642	0.01323	0.0049	0.01934	0.028	0.01625	0.04454	0.01368	0.04498	0.028	0.01992	0.0243	0.01701	0.07888	0.028	0.03297	0.01517	0.01237
0.032	0.00421	0.01047	0.00292	0.01571	0.032	0.01349	0.03895	0.0103	0.04051	0.032	0.01676	0.02119	0.01407	0.07064	0.032	0.0283	0.01236	0.00945
0.036	0.00255	0.00761	0.00121	0.01231	0.036	0.01102	0.03387	0.00745	0.03646	0.036	0.01346	0.01793	0.01087	0.06241	0.036	0.02375	0.00996	0.0067
0.04	0.00136	0.00573	2.70E-04	0.00964	0.04	0.00868	0.02924	0.00502	0.03256	0.04	0.01074	0.01541	0.00838	0.05556	0.04	0.01974	0.00776	0.00475
0.044	3.83E-04	0.00379	0	0.00726	0.044	0.00671	0.02492	0.00312	0.02888	0.044	0.00802	0.01282	0.00614	0.0487	0.044	0.01648	0.0057	0.00269
0.048	0	0.00248	2.25E-04	0.00493	0.048	0.00493	0.02054	0.00169	0.02592	0.048	0.00576	0.01023	0.00435	0.04252	0.048	0.01301	0.00407	0.00115
0.052	4.25E-05	0.00106	0.0013	0.00346	0.052	0.00332	0.01637	5.81E-04	0.02274	0.052	0.00375	0.00808	0.00275	0.03635	0.052	0.01019	0.00259	5.15E-04
0.056	4.25E-04	1.42E-04	0.00337	0.00153	0.056	0.00204	0.01276	0	0.01993	0.056	0.00226	0.00615	0.00147	0.03018	0.056	0.00759	0.00148	0
0.06	0.00217	1.06E-04	0.00675	8.51E-04	0.06	9.21E-04	0.01002	4.22E-04	0.01755	0.06	0.00123	0.00452	5.75E-04	0.02606	0.06	0.00618	8.75E-04	4.58E-04
0.064	0.00387	0	0.01048	1.13E-04	0.064	3.95E-04	0.00732	0.001	0.0148	0.064	5.18E-04	0.00311	6.39E-05	0.02112	0.064	0.00423	1.52E-04	0.00109
0.068	0.00651	6.37E-04	0.0157	0	0.068	1.64E-04	0.00483	0.00201	0.01213	0.068	0	0.00156	0	0.0166	0.068	0.00249	0	0.00229
0.072	0.00983	0.00173	0.02196	7.37E-04	0.072	0	0.0032	0.00349	0.01011	0.072	7.12E-04	9.63E-04	4.48E-04	0.01283	0.072	0.00141	1.14E-04	0.00452
0.076	0.01404	0.0035	0.02951	0.00164	0.076	4.93E-04	0.00173	0.00576	0.00794	0.076	0.00175	2.96E-04	0.00166	0.00953	0.076	4.34E-04	8.75E-04	0.00744
0.08	0.01893	0.00573	0.03851	0.00335	0.08	0.00115	5.08E-04	0.00861	0.00635	0.08	0.00291	0	0.00294	0.00658	0.08	1.08E-04	0.00164	0.01082
0.084	0.02489	0.00856	0.04886	0.00578	0.084	0.00227	5.08E-05	0.01199	0.00455	0.084	0.00498	7.41E-05	0.00486	0.00425	0.084	0	0.00304	0.01523
0.088	0.03152	0.01189	0.06101	0.00885	0.088	0.00382	0	0.01574	0.00332	0.088	0.00737	2.22E-04	0.00742	0.0024	0.088	2.17E-04	0.00506	0.0201
0.092	0.03901	0.01603	0.07451	0.01242	0.092	0.00589	2.03E-04	0.02038	0.00245	0.092	0.01106	0.00104	0.01125	0.00123	0.092	8.68E-04	0.00726	0.02617
0.096	0.04726	0.02095	0.09025	0.01645	0.096	0.00832	0.00153	0.02519	0.00159	0.096	0.01481	0.00222	0.01515	3.43E-04	0.096	0.00217	0.01012	0.03264
0.1	0.05581	0.02604	0.1069	0.02121	0.1	0.01095	0.0027	0.0301	7.22E-04	0.1	0.01973	0.00385	0.01956	0	0.1	0.00358	0.01339	0.04008
0.104	0.06534	0.03199	0.12535	0.0262	0.104	0.01418	0.00468	0.03591	4.33E-04	0.104	0.02484	0.00578	0.02487	6.86E-04	0.104	0.00521	0.01704	0.04799
0.108	0.07513	0.03839	0.14515	0.03182	0.108	0.01763	0.00702	0.04151	0	0.108	0.0306	0.00778	0.03062	0.00178	0.108	0.00716	0.02118	0.05646
0.112	0.08517	0.04529	0.16629	0.03754	0.112	0.02125	0.00971	0.04753	0	0.112	0.03694	0.01052	0.03715	0.00377	0.112	0.00976	0.02574	0.0658
0.116	0.09559	0.05237	0.18789	0.04344	0.116	0.02533	0.01241	0.0535	7.22E-05	0.116	0.04367	0.01393	0.04411	0.00604	0.116	0.01291	0.03042	0.07507
0.12	0.1058	0.06016	0.21128	0.04996	0.12	0.02954	0.01586	0.05952	2.17E-04	0.12	0.05143	0.01712	0.05191	0.00885	0.12	0.01638	0.03567	0.08469
0.124	0.11601	0.06829	0.23513	0.05631	0.124	0.03382	0.01932	0.06559	0.00108	0.124	0.05932	0.02067	0.05991	0.01255	0.124	0.02039	0.04099	0.09471
0.128	0.12622	0.07608	0.25988	0.06255	0.128	0.03875	0.02324	0.0714	0.00152	0.128	0.0678	0.02482	0.06854	0.01701	0.128	0.02559	0.04685	0.10519
0.132	0.13558	0.08457	0.28462	0.06936	0.132	0.04336	0.02695	0.07742	0.00195	0.132	0.07588	0.0289	0.07717	0.02133	0.132	0.0308	0.05248	0.11504
0.136	0.14494	0.09306	0.31027	0.07616	0.136	0.04829	0.03132	0.08323	0.00282	0.136	0.0841	0.03342	0.08625	0.02675	0.136	0.03687	0.05849	0.12552
0.14	0.15387	0.10156	0.33546	0.0824	0.14	0.05356	0.03539	0.08956	0.00361	0.14	0.09277	0.03786	0.09526	0.03224	0.14	0.04414	0.06453	0.13572
0.144	0.16195	0.11005	0.36066	0.08921	0.144	0.05816	0.03946	0.0959	0.00426	0.144	0.10131	0.04238	0.10447	0.03841	0.144	0.05195	0.07103	0.14597
0.148	0.17003	0.1189	0.38585	0.09545	0.148	0.06375	0.04404	0.10224	0.00527	0.148	0.10991	0.04675	0.11355	0.04595	0.148	0.06095	0.07746	0.15633
0.152	0.17769	0.12774	0.41105	0.10225	0.152	0.06902	0.04861	0.1091	0.00628	0.152	0.11813	0.0512	0.12275	0.0535	0.152	0.07027	0.08423	0.16692
0.156	0.18535	0.13694	0.43625	0.10962	0.156	0.07527	0.05421	0.11703	0.00722	0.156	0.12628	0.05609	0.13209	0.06241	0.156	0.08144	0.09134	0.17769
0.16	0.19343	0.1465	0.46099	0.117	0.16	0.08185	0.05929	0.12548	0.00816	0.16	0.13462	0.06091	0.14136	0.07202	0.16	0.09283	0.09879	0.18903
0.164	0.20066	0.1557	0.48484	0.12494	0.164	0.08876	0.06539	0.13392	0.00931	0.164	0.14245	0.06572	0.15082	0.08299	0.164	0.10454	0.10648	0.20025
0.168	0.20875	0.16525	0.50868	0.13288	0.168	0.09665	0.07149	0.1429	0.01032	0.168	0.1506	0.07039	0.16041	0.09534	0.168	0.11734	0.11473	0.21205
0.172	0.21725	0.17551	0.53163	0.14195	0.172	0.1052	0.07861	0.15294	0.01148	0.172	0.15901	0.0758	0.17071	0.10837	0.172	0.13003	0.12302	0.22465
0.176	0.22534	0.18542	0.55323	0.15102	0.176	0.11442	0.08675	0.16297	0.01256	0.176	0.16736	0.08047	0.18087	0.12346	0.176	0.14304	0.13184	0.23782
0.18	0.23427	0.19533	0.57347	0.1601	0.18	0.12396	0.09438	0.17353	0.01401	0.18	0.17531	0.08514	0.19161	0.13992	0.18	0.15541	0.14021	0.25099
0.184	0.24235	0.20559	0.59237	0.17031	0.184	0.13448	0.10302	0.18409	0.01523	0.184	0.18314	0.0898	0.20274	0.15706	0.184	0.16723	0.14895	0.26359
0.188	0.25086	0.2155	0.60947	0.17995	0.188	0.14534	0.11166	0.19466	0.01675	0.188	0.19097	0.09477	0.21463	0.17558	0.188	0.17829	0.1577	0.27733
0.192	0.25894	0.22505	0.62476	0.18902	0.192	0.15619	0.12031	0.20469	0.01827	0.192	0.19821	0.09944	0.22614	0.19547	0.192	0.18827	0.16568	0.28993

0.196	0.26618	0.23355	0.63781	0.19753	0.196	0.16705	0.12895	0.21367	0.01942	0.196	0.20527	0.10373	0.23701	0.21399	0.196	0.19672	0.17253	0.30195
0.2	0.27256	0.24168	0.64951	0.20547	0.2	0.17758	0.13709	0.22212	0.02108	0.2	0.21174	0.10788	0.24839	0.23388	0.2	0.20399	0.17937	0.31341
0.204	0.27766	0.24912	0.65941	0.21284	0.204	0.18712	0.14421	0.22898	0.02282	0.204	0.21652	0.11159	0.25874	0.2524	0.204	0.2092	0.1847	0.32371
0.208	0.28107	0.25513	0.66751	0.21908	0.208	0.19567	0.15031	0.23479	0.02404	0.208	0.2206	0.11463	0.26776	0.26955	0.208	0.21256	0.18812	0.33173
0.212	0.28319	0.26044	0.67291	0.22248	0.212	0.20291	0.1554	0.23902	0.02505	0.212	0.22312	0.11722	0.27575	0.28464	0.212	0.21419	0.19002	0.33803
0.216	0.28277	0.26362	0.67695	0.22475	0.216	0.20784	0.15895	0.24113	0.02585	0.216	0.22435	0.11929	0.28221	0.2963	0.216	0.21419	0.19078	0.34261
0.22	0.28149	0.2661	0.6801	0.22532	0.22	0.21179	0.16099	0.24166	0.02643	0.22	0.22364	0.12115	0.28713	0.3059	0.22	0.21245	0.18964	0.3449
0.224	0.27809	0.26681	0.681	0.22418	0.224	0.21344	0.1615	0.2406	0.02708	0.224	0.22222	0.12218	0.29033	0.31276	0.224	0.2092	0.18698	0.34433
0.228	0.27341	0.26575	0.67965	0.22135	0.228	0.21311	0.15997	0.23796	0.02765	0.228	0.21846	0.12307	0.29161	0.31481	0.228	0.20442	0.18242	0.34146
0.232	0.26745	0.26327	0.6765	0.21738	0.232	0.2108	0.15692	0.23426	0.02758	0.232	0.21361	0.12344	0.29097	0.31413	0.232	0.19879	0.17633	0.33631
0.236	0.26065	0.25973	0.67201	0.21227	0.236	0.20653	0.15336	0.22845	0.02729	0.236	0.20779	0.12337	0.28905	0.30933	0.236	0.1926	0.16987	0.32944
0.24	0.25299	0.25442	0.66481	0.20603	0.24	0.20093	0.14828	0.22264	0.02736	0.24	0.20087	0.12307	0.2856	0.30178	0.24	0.18523	0.16188	0.32085
0.244	0.24405	0.24805	0.65671	0.19923	0.244	0.19435	0.14217	0.21631	0.02708	0.244	0.19336	0.12159	0.28099	0.29081	0.244	0.17796	0.15352	0.31054
0.248	0.23555	0.24098	0.64681	0.19299	0.248	0.18679	0.13506	0.20891	0.02635	0.248	0.18541	0.121	0.27517	0.27778	0.248	0.17016	0.14477	0.29909
0.252	0.22704	0.23319	0.63691	0.18618	0.252	0.17856	0.12895	0.20205	0.02585	0.252	0.17719	0.11989	0.26904	0.26337	0.252	0.16246	0.13602	0.28764
0.256	0.21896	0.2247	0.62656	0.17938	0.256	0.16968	0.12183	0.19571	0.0252	0.256	0.16897	0.11907	0.26264	0.2476	0.256	0.15497	0.12766	0.27561
0.26	0.21087	0.2155	0.61487	0.17257	0.26	0.16113	0.11522	0.18885	0.02462	0.26	0.16056	0.11774	0.25663	0.23182	0.26	0.14727	0.1188	0.26301
0.264	0.20449	0.20665	0.60362	0.16747	0.264	0.1529	0.10912	0.18357	0.0244	0.264	0.15325	0.11685	0.25075	0.21536	0.264	0.14044	0.11134	0.25156
0.268	0.19811	0.19816	0.59327	0.16237	0.268	0.14534	0.10302	0.17881	0.02448	0.268	0.14601	0.11611	0.24493	0.19959	0.268	0.13393	0.10419	0.24011
0.272	0.19258	0.19038	0.58337	0.15783	0.272	0.13777	0.09794	0.17512	0.02426	0.272	0.13935	0.11515	0.23975	0.18519	0.272	0.12775	0.09739	0.22923
0.276	0.18833	0.18259	0.57392	0.15329	0.276	0.13152	0.09387	0.17195	0.02448	0.276	0.13333	0.11529	0.23502	0.17147	0.276	0.1219	0.0913	0.21949
0.28	0.18492	0.17551	0.56627	0.14989	0.28	0.1256	0.0898	0.16984	0.02477	0.28	0.12796	0.11507	0.23125	0.15912	0.28	0.11712	0.08606	0.21033
0.284	0.18237	0.1695	0.55907	0.14705	0.284	0.12067	0.08675	0.16878	0.02534	0.284	0.12311	0.11581	0.22844	0.14815	0.284	0.11289	0.08161	0.20231
0.288	0.18109	0.16419	0.55368	0.14479	0.288	0.11639	0.08471	0.16825	0.02643	0.288	0.11929	0.11648	0.22601	0.13923	0.288	0.10942	0.07754	0.19527
0.292	0.18067	0.15994	0.54918	0.14308	0.292	0.1131	0.08319	0.16878	0.02736	0.292	0.11554	0.11766	0.22473	0.131	0.292	0.10639	0.07434	0.18937
0.296	0.18067	0.1564	0.54603	0.14308	0.296	0.11113	0.08217	0.17036	0.02852	0.296	0.11282	0.11981	0.22422	0.12414	0.296	0.10422	0.07172	0.18405
0.3	0.18152	0.15393	0.54423	0.14308	0.3	0.10948	0.08217	0.173	0.03011	0.3	0.11069	0.12204	0.22435	0.11934	0.3	0.10303	0.06974	0.17969
0.304	0.18322	0.15287	0.54288	0.14422	0.304	0.10882	0.08268	0.17617	0.03184	0.304	0.10939	0.125	0.22518	0.11523	0.304	0.10303	0.06856	0.17637
0.308	0.18577	0.15216	0.54288	0.14592	0.308	0.10882	0.0837	0.17987	0.03408	0.308	0.10765	0.12893	0.22671	0.1118	0.308	0.10346	0.06776	0.17374
0.312	0.18875	0.15251	0.54333	0.14762	0.312	0.10915	0.08573	0.18462	0.03646	0.312	0.10687	0.13308	0.22818	0.10974	0.312	0.10433	0.06757	0.17151
0.316	0.19258	0.15393	0.54558	0.15046	0.316	0.11047	0.08827	0.19043	0.03913	0.316	0.10732	0.13856	0.23074	0.10905	0.316	0.10715	0.0678	0.17082
0.32	0.19726	0.15605	0.54873	0.15386	0.32	0.11277	0.09133	0.19677	0.04202	0.32	0.10732	0.14434	0.23349	0.10905	0.32	0.11062	0.06868	0.17059
0.324	0.20237	0.15853	0.55233	0.15726	0.324	0.1154	0.09488	0.20363	0.04505	0.324	0.10816	0.15116	0.23675	0.10905	0.324	0.11528	0.06978	0.17082
0.328	0.20747	0.16207	0.55683	0.16237	0.328	0.11869	0.09946	0.21208	0.04874	0.328	0.10965	0.15871	0.24052	0.1118	0.328	0.12059	0.07157	0.17225
0.332	0.21385	0.16667	0.56267	0.16747	0.332	0.12264	0.10404	0.22106	0.05249	0.332	0.11146	0.16709	0.24429	0.11385	0.332	0.12732	0.07392	0.1742
0.336	0.22066	0.17162	0.56807	0.17314	0.336	0.12692	0.11014	0.23057	0.05625	0.336	0.11353	0.17657	0.24909	0.11728	0.336	0.13556	0.07659	0.1762
0.34	0.22789	0.17728	0.57527	0.17995	0.34	0.13152	0.11624	0.2406	0.06094	0.34	0.1156	0.18702	0.25376	0.1214	0.34	0.14456	0.07978	0.17918
0.344	0.23555	0.1833	0.58292	0.18618	0.344	0.13679	0.12285	0.25222	0.06556	0.344	0.11884	0.1988	0.25893	0.1262	0.344	0.15541	0.08332	0.18273
0.348	0.24405	0.19038	0.59102	0.19412	0.348	0.14271	0.13048	0.26436	0.07054	0.348	0.12214	0.21132	0.26475	0.13237	0.348	0.16679	0.08731	0.18679
0.352	0.25341	0.19816	0.60047	0.20206	0.352	0.14863	0.13811	0.27704	0.0761	0.352	0.12654	0.22533	0.27089	0.13855	0.352	0.18035	0.09165	0.19166
0.356	0.26235	0.20594	0.60947	0.21	0.356	0.15521	0.14675	0.29024	0.08209	0.356	0.13055	0.24029	0.27722	0.1454	0.356	0.1951	0.09636	0.19699
0.36	0.27298	0.21479	0.61981	0.21908	0.36	0.16244	0.1559	0.30397	0.08859	0.36	0.13527	0.25652	0.28355	0.15364	0.36	0.21147	0.10172	0.20289
0.364	0.28447	0.2247	0.63151	0.22985	0.364	0.17067	0.16658	0.31929	0.09596	0.364	0.1409	0.27438	0.29161	0.16324	0.364	0.23002	0.10746	0.20976
0.368	0.29553	0.23496	0.64321	0.24006	0.368	0.17856	0.17675	0.3346	0.10332	0.368	0.14646	0.29268	0.29992	0.17284	0.368	0.24965	0.11359	0.21663
0.372	0.30744	0.24558	0.65536	0.25027	0.372	0.18679	0.18743	0.35044	0.1109	0.372	0.15287	0.31194	0.30823	0.18244	0.372	0.27058	0.11998	0.22465
0.376	0.3202	0.2569	0.66796	0.26161	0.376	0.19567	0.19862	0.36629	0.11935	0.376	0.15888	0.33158	0.31654	0.19342	0.376	0.29292	0.1269	0.23209
0.38	0.33296	0.26893	0.68145	0.27239	0.38	0.20488	0.21031	0.38266	0.12838	0.38	0.16574	0.35277	0.32677	0.20508	0.38	0.31754	0.13488	0.24068
0.384	0.34658	0.28132	0.6954	0.2843	0.384	0.21442	0.22201	0.39956	0.13747	0.384	0.17337	0.37456	0.33636	0.21674	0.384	0.34367	0.14249	0.24984
0.388	0.36019	0.29406	0.7098	0.29564	0.388	0.22396	0.23421	0.4154	0.147	0.388	0.18088	0.3959	0.34723	0.2284	0.388	0.37024	0.15085	0.259
0.392	0.37465	0.3075	0.72465	0.30698	0.392	0.23383	0.24642	0.43177	0.15747	0.392	0.18916	0.41835	0.35874	0.24143	0.392	0.39931	0.1596	0.26931
0.396	0.38912	0.32095	0.74039	0.31889	0.396	0.24337	0.25862	0.44814	0.16823	0.396	0.1975	0.44057	0.37024	0.25446	0.396	0.42945	0.16873	0.27905
0.4	0.40401	0.3351	0.75614	0.3308	0.4	0.25324	0.27082	0.46293	0.17935	0.4	0.20637	0.4628	0.38303	0.2668	0.4	0.46058	0.17785	0.28935
0.404	0.41847	0.34926	0.77144	0.34214	0.404	0.26278	0.28252	0.47824	0.19076	0.404	0.21536	0.48429	0.39582	0.27984	0.404	0.4929	0.18736	0.29909
0.408	0.43293	0.3627	0.78719	0.35292	0.408	0.27232	0.29472	0.49303	0.20217	0.408	0.22493	0.50504	0.40861	0.29218	0.408	0.52521	0.19687	0.3094
0.412	0.44697	0.3765	0.80293	0.36369	0.412	0.2812	0.30591	0.50676	0.21386	0.412	0.23431	0.52504	0.42267	0.30453	0.412	0.55786	0.20637	0.31913
0.416	0.46059	0.3903	0.81823	0.37333	0.416	0.28976	0.31659	0.51996	0.22542	0.416	0.24337	0.54431	0.43674	0.31619	0.416	0.59039	0.21512	0.32829
0.42	0.47377	0.40269	0.83308	0.38241	0.42	0.29765	0.32676	0.53264	0.23668	0.42	0.25243	0.56209	0.4508	0.32716	0.42	0.62108	0.22387	0.33688
0.424	0.48654	0.41472	0.84883	0.39148	0.424	0.30555	0.33642	0.54425	0.24816	0.424	0.26109	0.57913	0.46487	0.33813	0.424	0.65112	0.23223	0.34547
0.428	0.49845	0.42675	0.86322	0.39999	0.428	0.31311	0.34608	0.55587	0.25942	0.428	0.27035	0.59469	0.47957	0.34842	0.428	0.67899	0.23984	0.35349
0.432	0.50993	0.43808	0.87672	0.40736	0.432	0.32002	0.35422	0.56591	0.26996	0.432	0.27817	0.60803	0.49364	0.35802	0.432	0.70502	0.24744	0.36093
0.436	0.52057	0.44798	0.89022	0.4136	0.436	0.32594	0.36184	0.57541	0.27978	0.436	0.28574	0.61989	0.50706	0.36694	0.436	0.72888	0.25391	0.36781
0.44	0.52993	0.45754	0.90027	0.41984	0.44	0.33186	0.36896	0.58386	0.2896	0.44	0.29357	0.63026	0.51985	0.37517	0.44	0.74948	0.25999	0.37411
0.444	0.53929	0.46638	0.91541	0.42494	0.444	0.33713	0.37506	0.59178	0.29856	0.444	0.29939	0.63915	0.532	0.38272	0.444	0.76575	0.26608	0.37983
0.448	0.54737	0.47381	0.92711	0.42948	0.448	0.34173	0.38066	0.59918	0.30715	0.448	0.30521	0.6473	0.54351	0.38957	0.448	0.77768	0.2714	0.38441
0.452	0.5546	0.48018	0.93656	0.43231	0.452	0.34535	0.38523	0.60551	0.31437	0.452	0.31039	0.65249	0.5531	0.39438	0.452	0.78636	0.27558	0.38785
0.456	0.56098	0.48655	0.94556	0.43515	0.456	0.34897	0.38981	0.61079	0.32159	0.456	0.31492	0.65842	0.56205	0.39986	0.456	0.79286	0.28053	0.39128
0.46	0.56651	0.49186	0.95321	0.43799	0.46	0.35226	0.39337	0.61608	0.32823	0.46	0.31815	0.66286	0.57036	0.40466	0.46	0.79612	0.28433	0.39357
0.464	0.57119	0.49575	0.95906	0.43969	0.464	0.35456	0.39642	0.6203	0.33357	0.464	0.32139	0.66657	0.57675	0.40741	0.464	0.79612	0.28775	0.39529
0.468	0.57545	0.49929	0.96401	0.44082	0.468	0.35654	0.39896	0.624	0.33935	0.468	0.32397	0.66953	0.58123	0.41015	0.468	0.79395	0.29117	0.39587
0.472	0.578	0.50142	0.96761	0.44139	0.472	0.35752	0.40049	0.62611	0.34368	0.472	0.32592	0.67175	0.58506	0.41152	0.472	0.78961	0.29422	0.39644
0.476	0.58055	0.50283	0.96986	0.44196	0.476	0.35884	0.40201	0.62822	0.34729	0.476	0.32786	0.6725	0.58698	0.41152	0.476	0.78419	0.29688	0.39587
0.48	0.5814	0.50318	0.97031	0.44252	0.48	0.3595	0.40303	0.62875	0.3509	0.48	0.32915	0.67472	0.5889	0.41152	0.48	0.77768	0.29954	0.39415
0.484	0.5814	0.50248	0.96941	0.44196	0.484	0.35917	0.40354	0.62875	0.35379	0.484	0.3298	0.67472	0.58826	0.41084	0.484	0.76901	0.30144	0.39243
0.488	0.58055	0.50142	0.96806	0.44139	0.488	0.35917	0.40405	0.62875	0.35596	0.488	0.33044	0.67546	0.58698	0.40878	0.488	0.76033	0.30296	0.39071
0.492	0.57927	0.49929	0.96536	0.44025	0.492	0.35851	0.40405	0.62822	0.3574	0.492	0.33044	0.67546	0.58506	0.40741	0.492	0.75057	0.30448	0.38785
0.496	0.57715	0.49682	0.96131	0.43912	0.496	0.35752	0.40405	0.62664	0.35884	0.496	0.3298	0.67546	0.58251	0.40466	0.496	0.74081	0.30562	0.38556
0.5	0.57374	0.49328	0.95681	0.43799	0.5	0.35654	0.40354	0.62505	0.35957	0.5	0.3298	0.67472	0.57867	0.40192	0.5	0.73105	0.30638	0.38212
0.504	0.57034	0.48903	0.95096	0.43628	0.504	0.35522	0.40303	0.62241	0.35957	0.504	0.32915	0.67324	0.57484	0.39849	0.504	0.7202	0.30677	0.37926
0.508	0.56609	0.48443	0.94466	0.43572	0.508	0.3539	0.40252	0.61977	0.35957	0.508	0.3285	0.67324	0.56972	0.39575	0.508	0.71044	0.30715	0.37582
0.512	0.56183	0.47983	0.93836	0.43402	0.512	0.35259	0.40201	0.61713	0.35957	0.512	0.32721	0.67175	0.56525	0.39232	0.512	0.6996	0.30753	0.37296
0.516	0.55673	0.47417	0.93026	0.43231	0.516	0.35062	0.401	0.61396	0.35884	0.516	0.32592	0.66953	0.55949	0.38957	0.516	0.68875	0.30753	0.36952
0.52	0.55162	0.46921	0.92261	0.43118	0.52	0.3493	0.401	0.61079	0.35812	0.52	0.32462	0.66805	0.55374	0.38683	0.52	0.67899	0.30715	0.36609
0.524	0.54652	0.46355	0.91451	0.43005	0.524	0.34765	0.40049	0.60763	0.3574	0.524	0.32333	0.66583	0.54862	0.38409	0.524	0.66923	0.30677	0.3638
0.528	0.54141	0.45789	0.90597	0.42891	0.528	0.34634	0.39998	0.60446	0.35668	0.528	0.32139	0.66434	0.54287	0.38134	0.528	0.65947	0.30638	0.36093
0.532	0.53673	0.45223	0.89742	0.42834	0.532	0.34469	0.39998	0.60182	0.35523	0.532	0.32009	0.66286	0.53647	0.37997	0.532	0.65101	0.306	0.35807
0.536	0.53163	0.44728	0.88887	0.42778	0.536	0.34371	0.39947	0.59865	0.35451	0.536	0.31945	0.66064	0.53136	0.37791	0.536	0.64299	0.30524	0.35578
0.54	0.52695	0.44232	0.88077	0.42721	0.54	0.34272	0.39998	0.59601	0.35379	0.54	0.31815	0.65916	0.52561	0.37723	0.54	0.63572	0.30486	0.35406
0.544	0.5227	0.43737	0.87267	0.42721	0.544	0.34206	0.401	0.5939	0.35307	0.544	0.31751	0.65768	0.52049	0.37654	0.544	0.62889	0.30448	0.35177
0.548	0.51844	0.43312	0.86412	0.42721	0.548	0.3414	0.401	0.59125	0.35235	0.548	0.31621	0.65545	0.51538	0.37586	0.548	0.62271	0.30372	0.35063
0.552	0.51461	0.42887	0.85647	0.42778	0.552	0.34108	0.40201	0.58967	0.35162	0.552	0.31556	0.65397	0.51026	0.37654	0.552	0.6175	0.30334	0.34948
0.556	0.51121	0.42534	0.84973	0.42778	0.556	0.34075	0.40303	0.58861	0.35162	0.556	0.31556	0.65249	0.50643	0.37723	0.556	0.61327	0.30334	0.34834
0.56	0.50781	0.4218	0.84253	0.42834	0.56	0.34075	0.40456	0.58703	0.3509	0.56	0.31556	0.65101	0.50195	0.3786	0.56	0.60959	0.30296	0.34719
0.564	0.5044	0.41861	0.83578	0.42891	0.564	0.34075	0.40506	0.58597	0.3509	0.564	0.31621	0.65027	0.49811	0.37997	0.564	0.60601	0.30296	0.34662
0.568	0.50185	0.41543	0.82903	0.42948	0.568	0.34075	0.40608	0.58439	0.3509	0.568	0.31621	0.64878	0.49492	0.38203	0.568	0.6033	0.30296	0.34547
0.572	0.4993	0.4126	0.82318	0.43005	0.572	0.34108	0.4071	0.58386	0.3509	0.572	0.31686	0.64878	0.49236	0.38409	0.572	0.60134	0.30334	0.34547
0.576	0.49632	0.41047	0.81733	0.43061	0.576	0.3414	0.40862	0.58228	0.3509	0.576	0.31751	0.64804	0.4898	0.38615	0.576	0.5995	0.30334	0.34433
0.58	0.49334	0.40764	0.81148	0.43061	0.58	0.34108	0.40964	0.58069	0.35162	0.58	0.31815	0.64804	0.48725	0.3882	0.58	0.59722	0.30334	0.34376
0.584	0.49036	0.40481	0.80563	0.43061	0.584	0.3414	0.41015	0.57911	0.35162	0.584	0.3188	0.64804	0.48597	0.39026	0.584	0.59549	0.30334	0.34261
0.588	0.48739	0.40269	0.79978	0.43005	0.588	0.34075	0.41066	0.577	0.35162	0.588	0.32009	0.64804	0.48405	0.393	0.588	0.59386	0.30372	0.34204
0.592	0.48398	0.39986	0.79394	0.43005	0.592	0.34042	0.41117	0.57488	0.35235	0.592	0.32074	0.64804	0.48213	0.39506	0.592	0.59202	0.3041	0.34089
0.596	0.48058	0.39738	0.78854	0.42948	0.596	0.34009	0.41066	0.57172	0.35235	0.596	0.32139	0.64804	0.48085	0.39712	0.596	0.58974	0.30372	0.33917
0.6	0.47718	0.3942	0.78224	0.42834	0.6	0.3391	0.41015	0.56855	0.35235	0.6	0.32268	0.64804	0.47957	0.39849	0.6	0.58725	0.30372	0.33803
0.604	0.47335	0.39101	0.77594	0.42721	0.604	0.33779	0.41015	0.56485	0.35235	0.604	0.32333	0.64804	0.47829	0.40055	0.604	0.58464	0.30372	0.33631

0.608	0.46952	0.38818	0.77009	0.42608	0.608	0.3368	0.40913	0.56115	0.35235	0.608	0.32397	0.64804	0.47702	0.40192	0.608	0.58172	0.30334	0.33459
0.612	0.46527	0.38393	0.76334	0.42381	0.612	0.33515	0.40761	0.5564	0.35162	0.612	0.32462	0.64804	0.4751	0.40261	0.612	0.5777	0.30258	0.33288
0.616	0.46101	0.3804	0.75659	0.42211	0.616	0.33318	0.40608	0.55112	0.35162	0.616	0.32462	0.6473	0.47382	0.40329	0.616	0.57412	0.3022	0.33058
0.62	0.45591	0.3765	0.74939	0.41984	0.62	0.33121	0.40405	0.54584	0.3509	0.62	0.32462	0.64656	0.47254	0.40329	0.62	0.56935	0.30144	0.32829
0.624	0.45123	0.37226	0.74219	0.417	0.624	0.3289	0.40151	0.54003	0.35018	0.624	0.32462	0.64508	0.47062	0.40329	0.624	0.56436	0.30068	0.326
0.628	0.44655	0.36801	0.7341	0.4136	0.628	0.32627	0.39845	0.53316	0.34946	0.628	0.32462	0.64286	0.4687	0.40261	0.628	0.55872	0.29954	0.32314
0.632	0.44144	0.36377	0.726	0.4102	0.632	0.32298	0.39591	0.5263	0.34801	0.632	0.32397	0.64063	0.46615	0.40192	0.632	0.55243	0.29802	0.32028
0.636	0.43634	0.35916	0.7179	0.40679	0.636	0.32035	0.39235	0.51943	0.34657	0.636	0.32333	0.63841	0.46359	0.40055	0.636	0.5456	0.2965	0.31799
0.64	0.43081	0.35456	0.7098	0.40282	0.64	0.31739	0.3893	0.51204	0.34513	0.64	0.32268	0.63545	0.46167	0.39918	0.64	0.53866	0.2946	0.31455
0.644	0.42528	0.34961	0.7008	0.39829	0.644	0.3141	0.38523	0.50412	0.34296	0.644	0.32139	0.631	0.45847	0.39712	0.644	0.53053	0.29269	0.31169
0.648	0.42017	0.34501	0.6918	0.39432	0.648	0.31048	0.38167	0.49673	0.34079	0.648	0.32009	0.62656	0.45592	0.39506	0.648	0.52294	0.29041	0.30825
0.652	0.41464	0.34006	0.6828	0.38978	0.652	0.30719	0.37761	0.4888	0.33863	0.652	0.3188	0.62211	0.45272	0.39232	0.652	0.51437	0.28775	0.30539
0.656	0.40911	0.3351	0.67426	0.38581	0.656	0.30324	0.37354	0.48088	0.33646	0.656	0.31751	0.61618	0.44952	0.38957	0.656	0.5058	0.28509	0.30253
0.66	0.40358	0.33015	0.66526	0.38071	0.66	0.29962	0.36896	0.47296	0.33357	0.66	0.31556	0.60951	0.44569	0.38615	0.66	0.49691	0.28205	0.29909
0.664	0.39805	0.32519	0.65626	0.37617	0.664	0.29568	0.36439	0.46451	0.33018	0.664	0.31362	0.60285	0.44185	0.38272	0.664	0.48823	0.27862	0.29565
0.668	0.39252	0.32024	0.64726	0.3722	0.668	0.29206	0.36032	0.45712	0.327	0.668	0.31168	0.59618	0.43802	0.37929	0.668	0.47977	0.27558	0.29336
0.672	0.38742	0.31599	0.63871	0.36766	0.672	0.28877	0.35625	0.44973	0.3239	0.672	0.30974	0.58877	0.43482	0.37654	0.672	0.47121	0.27254	0.2905
0.676	0.38189	0.31104	0.62971	0.36313	0.676	0.28482	0.35167	0.4418	0.32014	0.676	0.3078	0.58136	0.43034	0.37243	0.676	0.46275	0.26874	0.28706
0.68	0.37678	0.30644	0.62116	0.35859	0.68	0.2812	0.34761	0.43441	0.31668	0.68	0.30521	0.57321	0.42651	0.369	0.68	0.45461	0.26532	0.2842
0.684	0.3721	0.30184	0.61262	0.35462	0.684	0.27791	0.34354	0.42755	0.31314	0.684	0.30327	0.56506	0.42267	0.36557	0.684	0.44691	0.26189	0.28077
0.688	0.367	0.29759	0.60362	0.35122	0.688	0.27462	0.33947	0.42121	0.30924	0.688	0.30198	0.55765	0.41884	0.36214	0.688	0.44008	0.25885	0.2779
0.692	0.36189	0.29335	0.59552	0.34725	0.692	0.271	0.3354	0.41434	0.30592	0.692	0.29939	0.5495	0.415	0.35802	0.692	0.43314	0.25505	0.27504
0.696	0.35764	0.28946	0.58742	0.34384	0.696	0.26771	0.33133	0.40853	0.30245	0.696	0.29745	0.54209	0.41116	0.3546	0.696	0.42674	0.25201	0.27218
0.7	0.35338	0.28556	0.57977	0.34044	0.7	0.26541	0.32777	0.40272	0.29935	0.7	0.29551	0.53468	0.40733	0.35117	0.7	0.421	0.24896	0.26988
0.704	0.34913	0.28167	0.57167	0.33704	0.704	0.26245	0.32472	0.39744	0.29617	0.704	0.29357	0.52727	0.40349	0.34774	0.704	0.41568	0.24592	0.26702
0.708	0.34488	0.27813	0.56402	0.3342	0.708	0.25949	0.32116	0.39216	0.293	0.708	0.29163	0.51986	0.40029	0.34431	0.708	0.41058	0.24288	0.26416
0.712	0.34105	0.27459	0.55683	0.33193	0.712	0.25719	0.31811	0.38741	0.29018	0.712	0.28969	0.51393	0.39646	0.34156	0.712	0.40614	0.23984	0.26187
0.716	0.33722	0.27141	0.54963	0.3291	0.716	0.25521	0.31557	0.38319	0.28765	0.716	0.28839	0.508	0.39326	0.33882	0.716	0.40223	0.23756	0.25958
0.72	0.33382	0.26822	0.54288	0.32683	0.72	0.25324	0.31252	0.37949	0.28542	0.72	0.28658	0.50207	0.39006	0.33608	0.72	0.39876	0.23527	0.25729
0.724	0.32999	0.26539	0.53613	0.32456	0.724	0.25094	0.30947	0.37579	0.28296	0.724	0.28503	0.49689	0.38687	0.33265	0.724	0.39529	0.23299	0.255
0.728	0.32701	0.26256	0.52938	0.32229	0.728	0.24929	0.30743	0.3721	0.28058	0.728	0.28367	0.49244	0.38367	0.3299	0.728	0.39215	0.23071	0.25271
0.732	0.32403	0.26008	0.52353	0.32059	0.732	0.24798	0.3054	0.36893	0.27899	0.732	0.28257	0.488	0.38111	0.32785	0.732	0.38987	0.22881	0.25099
0.736	0.32105	0.25761	0.51768	0.31946	0.736	0.24633	0.30337	0.36629	0.27726	0.736	0.28115	0.48429	0.37856	0.32579	0.736	0.38781	0.22691	0.2487
0.74	0.31808	0.25478	0.51183	0.31776	0.74	0.24502	0.30133	0.36312	0.27581	0.74	0.27998	0.48059	0.376	0.32305	0.74	0.38542	0.22539	0.24641
0.744	0.31552	0.25265	0.50643	0.31606	0.744	0.24403	0.2993	0.36101	0.27444	0.744	0.27908	0.47688	0.37344	0.32167	0.744	0.38401	0.22425	0.24526
0.748	0.31297	0.25088	0.50103	0.31549	0.748	0.24304	0.29777	0.35889	0.2735	0.748	0.27837	0.47466	0.37152	0.3203	0.748	0.38271	0.22311	0.24354
0.752	0.31084	0.24876	0.49609	0.31492	0.752	0.24206	0.29625	0.35678	0.27242	0.752	0.27759	0.47244	0.36897	0.31824	0.752	0.38174	0.22158	0.24183
0.756	0.30829	0.24699	0.49114	0.31379	0.756	0.2414	0.29523	0.3552	0.27134	0.756	0.27675	0.47021	0.36705	0.31687	0.756	0.38044	0.22082	0.24068
0.76	0.30616	0.24487	0.48619	0.31322	0.76	0.24074	0.2937	0.35361	0.27061	0.76	0.2761	0.46873	0.36449	0.3155	0.76	0.37957	0.21968	0.23896
0.764	0.30404	0.24345	0.48169	0.31379	0.764	0.24074	0.29269	0.35203	0.27018	0.764	0.27565	0.46725	0.36321	0.31481	0.764	0.37924	0.21892	0.23782
0.768	0.30191	0.24239	0.47764	0.31322	0.768	0.24041	0.29218	0.35044	0.2696	0.768	0.27552	0.46651	0.36129	0.31413	0.768	0.37892	0.21816	0.23667
0.772	0.30021	0.24062	0.47359	0.31322	0.772	0.24008	0.29116	0.34886	0.26903	0.772	0.275	0.46503	0.35938	0.31344	0.772	0.37816	0.2174	0.23553
0.776	0.29893	0.23992	0.47044	0.31379	0.776	0.24041	0.29116	0.34886	0.26946	0.776	0.27546	0.46577	0.35874	0.31413	0.776	0.37903	0.2174	0.23495
0.78	0.29723	0.23885	0.46729	0.31435	0.78	0.24074	0.29065	0.3478	0.26946	0.78	0.2752	0.46503	0.3581	0.31344	0.78	0.37892	0.21702	0.23438
0.784	0.29553	0.23815	0.46369	0.31492	0.784	0.24074	0.29015	0.34728	0.26939	0.784	0.27552	0.46503	0.35618	0.31344	0.784	0.37913	0.21626	0.23324
0.788	0.29383	0.23673	0.46054	0.31492	0.788	0.24074	0.28964	0.34569	0.26939	0.788	0.27539	0.46503	0.35554	0.31344	0.788	0.37935	0.21588	0.23266
0.792	0.29255	0.23602	0.45739	0.31549	0.792	0.24074	0.28913	0.34569	0.26924	0.792	0.27539	0.46577	0.35426	0.31344	0.792	0.37989	0.2155	0.23152
0.796	0.29085	0.23531	0.45469	0.31606	0.796	0.2414	0.28913	0.34516	0.26982	0.796	0.27552	0.46651	0.35362	0.31344	0.796	0.38033	0.21512	0.23152
0.8	0.29	0.23461	0.45244	0.31719	0.8	0.24173	0.28913	0.34463	0.26996	0.8	0.2763	0.46799	0.35298	0.31413	0.8	0.3812	0.21512	0.23095
0.804	0.28872	0.23425	0.44974	0.31776	0.804	0.24206	0.28862	0.34411	0.26996	0.804	0.2763	0.46873	0.35234	0.31413	0.804	0.38174	0.21474	0.23037
0.808	0.28745	0.2339	0.44749	0.31889	0.808	0.24271	0.28913	0.34358	0.27054	0.808	0.27694	0.47021	0.35234	0.31481	0.808	0.3825	0.21474	0.2298
0.812	0.2866	0.23319	0.44524	0.32002	0.812	0.24337	0.28913	0.34358	0.27134	0.812	0.27791	0.47095	0.3517	0.3155	0.812	0.38347	0.21474	0.22923
0.816	0.28574	0.23319	0.44344	0.32059	0.816	0.2437	0.28964	0.34358	0.2717	0.816	0.27837	0.47244	0.3517	0.31619	0.816	0.38488	0.21436	0.22923
0.82	0.28489	0.23284	0.44119	0.32116	0.82	0.24436	0.28964	0.34358	0.27227	0.82	0.27901	0.47466	0.3517	0.31687	0.82	0.38575	0.21474	0.22865
0.824	0.28362	0.23213	0.43895	0.32173	0.824	0.24469	0.28913	0.34305	0.27264	0.824	0.27927	0.4754	0.35106	0.31687	0.824	0.38651	0.21436	0.22808
0.828	0.28277	0.23248	0.4376	0.32229	0.828	0.24567	0.28964	0.34358	0.27372	0.828	0.28018	0.47762	0.35106	0.31824	0.828	0.38824	0.21436	0.22808
0.832	0.28234	0.23248	0.43625	0.32343	0.832	0.24633	0.29015	0.34358	0.27444	0.832	0.28115	0.47985	0.35106	0.31893	0.832	0.38987	0.21474	0.22808
0.836	0.28149	0.23213	0.4349	0.32456	0.836	0.24699	0.29015	0.34358	0.27516	0.836	0.28173	0.48133	0.3517	0.31962	0.836	0.39096	0.21474	0.22751
0.84	0.28149	0.23284	0.43355	0.3257	0.84	0.24798	0.29065	0.34411	0.27625	0.84	0.28251	0.48355	0.3517	0.32099	0.84	0.39215	0.21512	0.22751
0.844	0.28149	0.23319	0.43265	0.32626	0.844	0.24896	0.29116	0.34411	0.27762	0.844	0.28361	0.48577	0.35234	0.32236	0.844	0.39421	0.2155	0.22808
0.848	0.28107	0.23355	0.43175	0.3274	0.848	0.24995	0.29167	0.34463	0.27856	0.848	0.28464	0.48874	0.35298	0.32305	0.848	0.39584	0.21588	0.22808
0.852	0.28107	0.23319	0.43085	0.32796	0.852	0.25061	0.29218	0.34516	0.27986	0.852	0.28568	0.49096	0.35298	0.32442	0.852	0.39768	0.21626	0.22808
0.856	0.28107	0.23355	0.42995	0.3291	0.856	0.2516	0.29218	0.34516	0.28108	0.856	0.28684	0.49318	0.35426	0.32579	0.856	0.3992	0.21702	0.22808
0.86	0.28107	0.23425	0.4295	0.33023	0.86	0.25291	0.29269	0.34622	0.28231	0.86	0.28762	0.49689	0.35426	0.32647	0.86	0.40115	0.21778	0.22865
0.864	0.28107	0.23496	0.42905	0.3308	0.864	0.2539	0.2932	0.34675	0.28361	0.864	0.28839	0.49911	0.35554	0.32785	0.864	0.40332	0.21854	0.22923
0.868	0.28149	0.23531	0.4286	0.33137	0.868	0.25489	0.2937	0.34728	0.28491	0.868	0.28969	0.50207	0.35554	0.32922	0.868	0.40527	0.2193	0.22923
0.872	0.28192	0.23602	0.42815	0.3325	0.872	0.25587	0.29421	0.3478	0.28664	0.872	0.29098	0.50504	0.35682	0.33059	0.872	0.40744	0.22044	0.2298
0.876	0.28234	0.23673	0.42815	0.33364	0.876	0.25752	0.29523	0.34886	0.28852	0.876	0.29228	0.50874	0.35746	0.33196	0.876	0.41037	0.22158	0.23037
0.88	0.28277	0.23779	0.42815	0.33477	0.88	0.2585	0.29574	0.34992	0.2904	0.88	0.29357	0.51097	0.35874	0.33333	0.88	0.4121	0.22273	0.23095
0.884	0.28319	0.2385	0.4277	0.33477	0.884	0.25982	0.29625	0.35044	0.29184	0.884	0.29422	0.51467	0.35938	0.33471	0.884	0.41405	0.22387	0.23152
0.888	0.28404	0.23921	0.4277	0.3359	0.888	0.26081	0.29676	0.35097	0.29357	0.888	0.29551	0.51763	0.36002	0.33608	0.888	0.41633	0.22501	0.23209
0.892	0.28447	0.24027	0.4277	0.33647	0.892	0.26212	0.29777	0.3515	0.29552	0.892	0.2968	0.5206	0.36129	0.33745	0.892	0.41861	0.22615	0.23324
0.896	0.28532	0.24133	0.4277	0.33647	0.896	0.26344	0.29828	0.35256	0.29697	0.896	0.2981	0.52356	0.36193	0.33882	0.896	0.42132	0.22767	0.23381
0.9	0.28617	0.24204	0.4277	0.33704	0.9	0.26475	0.29879	0.35308	0.29884	0.9	0.29874	0.52653	0.36257	0.34019	0.9	0.42284	0.22881	0.23495
0.904	0.28702	0.24345	0.4277	0.33761	0.904	0.26607	0.29981	0.35414	0.30087	0.904	0.30004	0.52949	0.36385	0.34156	0.904	0.42512	0.23033	0.2361
0.908	0.28787	0.24487	0.4286	0.33761	0.908	0.26706	0.30032	0.35467	0.30274	0.908	0.30133	0.53245	0.36449	0.34294	0.908	0.42739	0.23223	0.23667
0.912	0.28872	0.24593	0.4286	0.33761	0.912	0.26837	0.30133	0.3552	0.30455	0.912	0.30263	0.53468	0.36577	0.34499	0.912	0.42967	0.23375	0.23782
0.916	0.29	0.24699	0.42905	0.33817	0.916	0.26969	0.30184	0.35572	0.30621	0.916	0.30392	0.5369	0.36641	0.34636	0.916	0.4313	0.23565	0.23896
0.92	0.29085	0.24805	0.4295	0.33817	0.92	0.271	0.30235	0.35625	0.30801	0.92	0.30521	0.53912	0.36705	0.34774	0.92	0.43303	0.23718	0.24011
0.924	0.29255	0.24947	0.4304	0.33874	0.924	0.27232	0.30337	0.35731	0.30996	0.924	0.30651	0.54135	0.36833	0.34979	0.924	0.43477	0.23946	0.24125
0.928	0.29383	0.25088	0.43085	0.33817	0.928	0.27364	0.30387	0.35784	0.31177	0.928	0.3078	0.54283	0.36961	0.35117	0.928	0.4364	0.24136	0.24297
0.932	0.2951	0.25195	0.4313	0.33874	0.932	0.27462	0.30438	0.35837	0.31357	0.932	0.3091	0.54431	0.37024	0.35254	0.932	0.4377	0.24326	0.24412
0.936	0.29638	0.25336	0.43175	0.33817	0.936	0.27594	0.3054	0.35889	0.31545	0.936	0.31039	0.54505	0.37088	0.3546	0.936	0.43932	0.24516	0.24526
0.94	0.29766	0.25478	0.4331	0.33874	0.94	0.27726	0.30642	0.35942	0.31755	0.94	0.31168	0.54579	0.37216	0.35665	0.94	0.44084	0.24744	0.24755
0.944	0.29936	0.25619	0.434	0.33874	0.944	0.27857	0.30743	0.36048	0.31935	0.944	0.31298	0.54653	0.37344	0.35802	0.944	0.44214	0.24972	0.24927
0.948	0.30106	0.25796	0.43445	0.33874	0.948	0.28022	0.30845	0.36101	0.32116	0.948	0.31427	0.54727	0.37408	0.36008	0.948	0.44366	0.25201	0.25042
0.952	0.30276	0.25938	0.4358	0.33874	0.952	0.28153	0.30947	0.36101	0.3231	0.952	0.31556	0.54801	0.37536	0.36214	0.952	0.44496	0.25429	0.25271
0.956	0.30446	0.26115	0.43625	0.33931	0.956	0.28285	0.31049	0.36153	0.32462	0.956	0.31751	0.54801	0.37664	0.3642	0.956	0.44583	0.25657	0.25442
0.96	0.30616	0.26292	0.43715	0.33931	0.96	0.28416	0.3115	0.36259	0.32664	0.96	0.31945	0.54876	0.37728	0.36626	0.96	0.44702	0.25923	0.25671
0.964	0.30829	0.26469	0.43805	0.33931	0.964	0.28548	0.31252	0.36312	0.32838	0.964	0.32074	0.54801	0.37856	0.36763	0.964	0.44735	0.26151	0.259
0.968	0.30999	0.26645	0.43895	0.33987	0.968	0.28712	0.31404	0.36365	0.33032	0.968	0.32268	0.54876	0.37984	0.37037	0.968	0.44876	0.26417	0.26072
0.972	0.31212	0.26822	0.4403	0.34044	0.972	0.28844	0.31506	0.36417	0.33206	0.972	0.32462	0.54876	0.38111	0.37243	0.972	0.45049	0.26684	0.26301
0.976	0.31425	0.26999	0.44164	0.34101	0.976	0.29008	0.31659	0.36523	0.3343	0.976	0.32656	0.54876	0.38239	0.37517	0.976	0.45158	0.2695	0.2653
0.98	0.31595	0.27212	0.44299	0.34158	0.98	0.29206	0.3176	0.36576	0.33646	0.98	0.3285	0.54876	0.38367	0.37723	0.98	0.45353	0.27254	0.26817
0.984	0.3185	0.27424	0.44389	0.34158	0.984	0.2937	0.31913	0.36681	0.33863	0.984	0.33044	0.54876	0.38559	0.37997	0.984	0.45461	0.2752	0.27046
0.988	0.32063	0.27636	0.44569	0.34271	0.988	0.29535	0.32116	0.36734	0.34079	0.988	0.33303	0.54876	0.38687	0.38272	0.988	0.45624	0.27824	0.27332
0.992	0.32318	0.27849	0.44704	0.34384	0.992	0.29732	0.32269	0.3684	0.34296	0.992	0.33497	0.54876	0.38815	0.38546	0.992	0.45798	0.28129	0.27618
0.996	0.32531	0.28061	0.44839	0.34441	0.996	0.29897	0.32421	0.36893	0.34513	0.996	0.33691	0.54876	0.39006	0.3882	0.996	0.45917	0.28395	0.27847
1	0.32786	0.28309	0.45019	0.34555	1	0.30127	0.32625	0.36998	0.34729	1	0.3395	0.54876	0.39198	0.39095	1	0.4608	0.28737	0.28191
1.004	0.33041	0.28521	0.45199	0.34668	1.004	0.30324	0.32828	0.37157	0.35018	1.004	0.34209	0.5495	0.3939	0.39369	1.004	0.46296	0.29041	0.28477
1.008	0.33296	0.28804	0.45334	0.34838	1.008	0.30555	0.33032	0.3721	0.35307	1.008	0.34468	0.55024	0.39582	0.39712	1.008	0.46481	0.29384	0.28764
1.012	0.33552	0.29016	0.45514	0.34895	1.012	0.30785	0.33235	0.37368	0.35523	1.012	0.34662	0.55024	0.3971	0.39986	1.012	0.46698	0.29688	0.29107
1.016	0.33807	0.29264	0.45739	0.35008	1.016	0.30982	0.33438	0.37474	0.35812	1.016	0.34985	0.55098	0.39902	0.40261	1.016	0.4685	0.3003	0.29451

1.02	0.34147	0.29547	0.45919	0.35178	1.02	0.31245	0.33642	0.37632	0.36101	1.02	0.35309	0.55098	0.40157	0.40604	1.02	0.47066	0.30372	0.29794
1.024	0.34445	0.2983	0.46189	0.35348	1.024	0.31476	0.33896	0.3779	0.36318	1.024	0.35567	0.55246	0.40413	0.40947	1.024	0.47294	0.30753	0.30138
1.028	0.347	0.30078	0.46369	0.35519	1.028	0.31739	0.3415	0.37949	0.36606	1.028	0.35826	0.55246	0.40605	0.41221	1.028	0.47468	0.31095	0.30482
1.032	0.35041	0.30396	0.46639	0.35745	1.032	0.32002	0.34405	0.38107	0.36895	1.032	0.36214	0.5532	0.40861	0.41632	1.032	0.47728	0.31437	0.30882
1.036	0.35338	0.30715	0.46909	0.35916	1.036	0.32298	0.3471	0.38319	0.37256	1.036	0.36538	0.55468	0.41116	0.41975	1.036	0.47977	0.31817	0.31283
1.04	0.35679	0.31033	0.47134	0.36142	1.04	0.32594	0.34964	0.3853	0.37545	1.04	0.36926	0.55616	0.41436	0.42318	1.04	0.48227	0.32198	0.31741
1.044	0.35977	0.31352	0.47449	0.36369	1.044	0.3289	0.35269	0.38741	0.37834	1.044	0.37249	0.55765	0.41756	0.4273	1.044	0.48465	0.32578	0.322
1.048	0.36359	0.3167	0.47719	0.36596	1.048	0.33219	0.35574	0.38952	0.38195	1.048	0.37637	0.55913	0.42011	0.43073	1.048	0.48704	0.32996	0.326
1.052	0.36742	0.32024	0.48034	0.3688	1.052	0.33548	0.35879	0.39216	0.38556	1.052	0.3809	0.56135	0.42395	0.43484	1.052	0.49073	0.33414	0.33058
1.056	0.37125	0.32413	0.48349	0.37163	1.056	0.3391	0.36286	0.3948	0.38917	1.056	0.38478	0.56283	0.42715	0.43896	1.056	0.49344	0.33833	0.33517
1.06	0.37465	0.32767	0.48664	0.37447	1.06	0.34239	0.36591	0.39744	0.39278	1.06	0.38931	0.56506	0.43034	0.44307	1.06	0.49615	0.34251	0.33975
1.064	0.37891	0.33156	0.49024	0.37787	1.064	0.34634	0.36947	0.40008	0.39711	1.064	0.39384	0.56728	0.43418	0.44719	1.064	0.4994	0.34669	0.3449
1.068	0.38274	0.33581	0.49429	0.38071	1.068	0.35029	0.37354	0.40325	0.40144	1.068	0.39837	0.57024	0.43802	0.45199	1.068	0.50277	0.35126	0.35005
1.072	0.38742	0.34006	0.49744	0.38354	1.072	0.35423	0.37761	0.40642	0.40578	1.072	0.4029	0.57247	0.44249	0.4561	1.072	0.50613	0.35582	0.35521
1.076	0.39125	0.3443	0.50148	0.38751	1.076	0.35818	0.38167	0.40959	0.41011	1.076	0.40807	0.57543	0.44633	0.46091	1.076	0.51003	0.36076	0.36036
1.08	0.3955	0.34855	0.50508	0.39091	1.08	0.36312	0.38574	0.41276	0.41444	1.08	0.41325	0.57839	0.4508	0.46571	1.08	0.51361	0.36533	0.36552
1.084	0.4006	0.3535	0.50958	0.39545	1.084	0.36805	0.39083	0.41698	0.41949	1.084	0.41842	0.5821	0.45592	0.47119	1.084	0.51806	0.37065	0.37124
1.088	0.40486	0.35846	0.51408	0.39942	1.088	0.37266	0.3954	0.42121	0.42455	1.088	0.42425	0.5858	0.46039	0.47668	1.088	0.52218	0.37597	0.37754
1.092	0.40954	0.36341	0.51768	0.40339	1.092	0.37726	0.39947	0.4249	0.42888	1.092	0.42942	0.58877	0.46487	0.48148	1.092	0.52673	0.38054	0.38269
1.096	0.41464	0.36837	0.52263	0.40793	1.096	0.38253	0.40456	0.42913	0.43466	1.096	0.43524	0.59247	0.46998	0.48697	1.096	0.53118	0.38586	0.38899
1.1	0.41975	0.37367	0.52758	0.41247	1.1	0.38779	0.40964	0.43335	0.43971	1.1	0.44171	0.59692	0.4751	0.49246	1.1	0.53573	0.39157	0.39529
1.104	0.42485	0.37933	0.53253	0.41757	1.104	0.39272	0.41523	0.43811	0.44549	1.104	0.44754	0.60136	0.48085	0.49863	1.104	0.54072	0.39727	0.40159
1.108	0.43038	0.385	0.53703	0.42211	1.108	0.39832	0.42032	0.44233	0.45054	1.108	0.45336	0.60581	0.48597	0.50412	1.108	0.54517	0.40259	0.40789
1.112	0.43634	0.39066	0.54243	0.42721	1.112	0.40424	0.42591	0.44708	0.45632	1.112	0.45983	0.61025	0.49172	0.51029	1.112	0.54994	0.4083	0.41476
1.116	0.44229	0.39703	0.54828	0.43288	1.116	0.41016	0.43252	0.45237	0.46282	1.116	0.4663	0.61544	0.49747	0.51646	1.116	0.55482	0.41476	0.42221
1.12	0.44825	0.4034	0.55368	0.43855	1.12	0.41674	0.43863	0.45817	0.46931	1.12	0.47341	0.62063	0.50387	0.52263	1.12	0.56057	0.42085	0.42965
1.124	0.45421	0.40941	0.55952	0.44366	1.124	0.42299	0.44473	0.46346	0.47581	1.124	0.48053	0.62582	0.50962	0.52881	1.124	0.56556	0.42731	0.43652
1.128	0.46059	0.41649	0.56582	0.4499	1.128	0.42957	0.45134	0.46979	0.48303	1.128	0.48764	0.631	0.51602	0.53567	1.128	0.57087	0.43378	0.44397
1.132	0.46739	0.42286	0.57212	0.45613	1.132	0.43615	0.45795	0.47613	0.48953	1.132	0.49541	0.63693	0.52305	0.54252	1.132	0.57651	0.44024	0.45141
1.136	0.47377	0.42994	0.57842	0.46237	1.136	0.44338	0.46507	0.48194	0.49675	1.136	0.50317	0.64286	0.52944	0.54938	1.136	0.58226	0.44747	0.45943
1.14	0.48058	0.43701	0.58427	0.46861	1.14	0.45029	0.47219	0.48828	0.50397	1.14	0.51029	0.64878	0.53647	0.55624	1.14	0.58833	0.45469	0.46687
1.144	0.48781	0.44444	0.59102	0.47542	1.144	0.45786	0.4793	0.49514	0.51119	1.144	0.51805	0.65545	0.54351	0.56379	1.144	0.59451	0.46192	0.47489
1.148	0.49504	0.45223	0.59777	0.48279	1.148	0.46575	0.48693	0.50201	0.51913	1.148	0.52711	0.66212	0.55118	0.57133	1.148	0.60124	0.46952	0.48348
1.152	0.5027	0.46072	0.60497	0.49016	1.152	0.47398	0.49507	0.5094	0.52708	1.152	0.53552	0.66879	0.55949	0.57888	1.152	0.60796	0.47751	0.4915
1.156	0.50993	0.46851	0.61172	0.4981	1.156	0.48187	0.5027	0.51679	0.53502	1.156	0.54393	0.67546	0.56716	0.58642	1.156	0.61468	0.48549	0.50009
1.16	0.51802	0.47735	0.61891	0.50604	1.16	0.49076	0.51134	0.52471	0.54368	1.16	0.55298	0.68287	0.57547	0.59465	1.16	0.62217	0.49424	0.50925
1.164	0.52652	0.48585	0.62701	0.51398	1.164	0.49931	0.51948	0.53316	0.55235	1.164	0.56204	0.69028	0.58315	0.60288	1.164	0.62932	0.50299	0.51841
1.168	0.53503	0.49469	0.63421	0.52305	1.168	0.50852	0.52812	0.54109	0.56101	1.168	0.5711	0.69843	0.5921	0.61111	1.168	0.63648	0.51211	0.52757
1.172	0.54311	0.50389	0.64186	0.53156	1.172	0.51773	0.53727	0.54954	0.5704	1.172	0.58015	0.70658	0.60041	0.62003	1.172	0.64407	0.52124	0.53731
1.176	0.55205	0.51345	0.64996	0.54063	1.176	0.52694	0.54643	0.55798	0.57978	1.176	0.58986	0.71473	0.60936	0.62894	1.176	0.65166	0.53074	0.54647
1.18	0.56141	0.52371	0.65851	0.54971	1.18	0.53681	0.55609	0.56696	0.58989	1.18	0.59956	0.72362	0.61831	0.63855	1.18	0.65947	0.54025	0.55678
1.184	0.57162	0.53432	0.66751	0.56048	1.184	0.548	0.56626	0.577	0.6	1.184	0.61056	0.73325	0.62854	0.64883	1.184	0.66815	0.55128	0.56823
1.188	0.58098	0.54459	0.67561	0.57012	1.188	0.55819	0.57592	0.5865	0.61011	1.188	0.62026	0.74215	0.63749	0.65775	1.188	0.67682	0.56117	0.57796
1.192	0.59161	0.55556	0.68505	0.58033	1.192	0.56938	0.5866	0.59601	0.62022	1.192	0.63126	0.75104	0.64772	0.66804	1.192	0.6855	0.57181	0.58884
1.196	0.60267	0.56653	0.6945	0.59111	1.196	0.58056	0.59727	0.60657	0.63105	1.196	0.64161	0.76067	0.65731	0.67764	1.196	0.69418	0.58322	0.59973
1.2	0.61331	0.57785	0.70395	0.60188	1.2	0.59208	0.60846	0.61713	0.64188	1.2	0.65325	0.76956	0.6669	0.68724	1.2	0.70394	0.59463	0.61118
1.204	0.62394	0.58988	0.71295	0.61323	1.204	0.60392	0.61965	0.62769	0.65199	1.204	0.66425	0.77845	0.67713	0.69753	1.204	0.71261	0.60566	0.62206
1.208	0.635	0.60156	0.7224	0.62457	1.208	0.61609	0.63134	0.63878	0.66354	1.208	0.67525	0.78809	0.688	0.70782	1.208	0.72237	0.61783	0.63408
1.212	0.64649	0.6143	0.7323	0.63591	1.212	0.62925	0.64304	0.64987	0.67509	1.212	0.68689	0.79772	0.69951	0.71811	1.212	0.73213	0.63	0.64611
1.216	0.65883	0.62703	0.74219	0.64839	1.216	0.64208	0.65524	0.66202	0.68664	1.216	0.69854	0.80735	0.71038	0.72908	1.216	0.74189	0.64293	0.65871
1.22	0.67074	0.64013	0.75209	0.66086	1.22	0.65491	0.66745	0.67417	0.69892	1.22	0.71018	0.81624	0.72125	0.74005	1.22	0.75165	0.65547	0.67073
1.224	0.6835	0.65428	0.76289	0.67391	1.224	0.66873	0.68067	0.68684	0.71119	1.224	0.72247	0.82587	0.73275	0.75171	1.224	0.7625	0.66916	0.6839
1.228	0.69669	0.66844	0.77324	0.68752	1.228	0.68254	0.69338	0.70004	0.72419	1.228	0.73477	0.83625	0.7449	0.76269	1.228	0.77334	0.68285	0.69822
1.232	0.70987	0.6833	0.78449	0.70113	1.232	0.69702	0.7066	0.71324	0.73718	1.232	0.74706	0.84588	0.75705	0.77435	1.232	0.78419	0.69692	0.71196
1.236	0.72306	0.69781	0.79483	0.71531	1.236	0.71149	0.71982	0.72592	0.74946	1.236	0.75935	0.85403	0.76856	0.78532	1.236	0.79395	0.71061	0.72571
1.24	0.73752	0.71302	0.80608	0.72948	1.24	0.7263	0.73406	0.74018	0.76245	1.24	0.77229	0.86366	0.78134	0.79767	1.24	0.80479	0.72544	0.74002
1.244	0.75241	0.72895	0.81778	0.7448	1.244	0.74209	0.74881	0.75391	0.77617	1.244	0.78652	0.8733	0.79477	0.81001	1.244	0.81672	0.74065	0.75434
1.248	0.7673	0.74558	0.82993	0.76011	1.248	0.75788	0.76457	0.76869	0.79061	1.248	0.8001	0.88219	0.80756	0.82236	1.248	0.82757	0.75663	0.7698
1.252	0.78219	0.76185	0.84118	0.77542	1.252	0.77334	0.77931	0.78348	0.80433	1.252	0.81369	0.89108	0.82034	0.83471	1.252	0.8395	0.77222	0.78469
1.256	0.79793	0.77919	0.85377	0.79187	1.256	0.79045	0.79659	0.7988	0.81877	1.256	0.82792	0.90071	0.83441	0.84636	1.256	0.85143	0.78895	0.80072
1.26	0.8141	0.79724	0.86682	0.80775	1.26	0.80755	0.81186	0.81517	0.83394	1.26	0.84215	0.91034	0.84784	0.8594	1.26	0.86444	0.80606	0.81733
1.264	0.83069	0.81529	0.87942	0.82533	1.264	0.82499	0.82864	0.83154	0.84838	1.264	0.85639	0.91924	0.86126	0.87311	1.264	0.87637	0.82355	0.83393
1.268	0.8477	0.83333	0.89202	0.84234	1.268	0.84275	0.84491	0.84844	0.86354	1.268	0.87191	0.92813	0.87533	0.88615	1.268	0.88938	0.84105	0.85054
1.272	0.86472	0.85209	0.90417	0.86049	1.272	0.86118	0.86271	0.86586	0.88014	1.272	0.88679	0.93702	0.89067	0.89986	1.272	0.90131	0.8593	0.86772
1.276	0.88301	0.8719	0.91766	0.8792	1.276	0.87993	0.88101	0.88329	0.89603	1.276	0.90232	0.94591	0.90538	0.91358	1.276	0.91541	0.87793	0.8849
1.28	0.90173	0.89243	0.93116	0.89849	1.28	0.89868	0.89983	0.90177	0.91191	1.28	0.91784	0.95554	0.92072	0.9273	1.28	0.92842	0.89733	0.90322
1.284	0.9196	0.9126	0.94376	0.9172	1.284	0.91776	0.91813	0.91973	0.92852	1.284	0.93337	0.96443	0.93479	0.94102	1.284	0.94252	0.91634	0.92155
1.288	0.94002	0.93454	0.95816	0.93762	1.288	0.93848	0.93847	0.93927	0.94657	1.288	0.95019	0.97407	0.95141	0.95679	1.288	0.95662	0.93726	0.94102
1.292	0.96001	0.95577	0.97255	0.95747	1.292	0.95888	0.9583	0.95934	0.96462	1.292	0.96701	0.98222	0.96739	0.97119	1.292	0.97072	0.95741	0.96049
1.296	0.97916	0.97771	0.98605	0.97845	1.296	0.9796	0.97915	0.9794	0.98267	1.296	0.98318	0.99111	0.98338	0.9856	1.296	0.98482	0.9787	0.98053
1.3	1	1	1	1	1.3	1	1	1	1	1.3	1	1	1	1	1.3	1	1	1
Water-ABS (Spring)					Water-ABS (Summer)					Water-ABS (Autumn)					Water-ABS (Winter)			
0.004	0.08527	0.07648	0.09363	0.06996	0.004	0.10645	0.12135	0.12517	0.04526	0.004	0.13778	0.051	0.08686	0.03787	0.004	0.17475	0.04878	0.10381
0.008	0.07976	0.0717	0.08742	0.06506	0.008	0.10156	0.11552	0.11767	0.04152	0.008	0.1284	0.04719	0.08138	0.03579	0.008	0.16505	0.04566	0.09719
0.012	0.0752	0.06774	0.08259	0.06147	0.012	0.09668	0.11085	0.11187	0.03836	0.012	0.12119	0.04452	0.07726	0.03419	0.012	0.15709	0.04286	0.0916
0.016	0.07138	0.06432	0.0789	0.05853	0.016	0.09277	0.10696	0.10709	0.03595	0.016	0.11524	0.04228	0.07349	0.03273	0.016	0.15043	0.04068	0.08718
0.02	0.06767	0.06184	0.07568	0.05625	0.02	0.08919	0.10346	0.10266	0.03386	0.02	0.11049	0.04089	0.07087	0.03134	0.02	0.14478	0.0385	0.08335
0.024	0.06494	0.05948	0.07292	0.05429	0.024	0.08594	0.10035	0.09925	0.03221	0.024	0.10643	0.03961	0.06838	0.03014	0.024	0.13957	0.0365	0.07992
0.028	0.06249	0.05754	0.07085	0.05233	0.028	0.08301	0.09763	0.09652	0.0306	0.028	0.10282	0.0384	0.06617	0.02923	0.028	0.13508	0.03488	0.07688
0.032	0.06019	0.0557	0.06878	0.0507	0.032	0.0804	0.09529	0.09413	0.02932	0.032	0.10002	0.03752	0.06468	0.02824	0.032	0.13103	0.03336	0.07438
0.036	0.05797	0.05406	0.06694	0.04972	0.036	0.0778	0.09257	0.09141	0.02803	0.036	0.09727	0.03679	0.06312	0.02751	0.036	0.12755	0.03174	0.07212
0.04	0.05604	0.05255	0.06533	0.04809	0.04	0.07552	0.09063	0.08936	0.02707	0.04	0.09527	0.03594	0.06184	0.02671	0.04	0.12408	0.03024	0.06971
0.044	0.05426	0.05113	0.06418	0.04711	0.044	0.07292	0.08868	0.08799	0.02643	0.044	0.09298	0.03547	0.06056	0.02609	0.044	0.12075	0.029	0.06765
0.048	0.05256	0.0498	0.0628	0.0458	0.048	0.07096	0.08674	0.08595	0.02552	0.048	0.09149	0.03496	0.05935	0.02529	0.048	0.11756	0.02766	0.06559
0.052	0.05087	0.04853	0.06188	0.04482	0.052	0.06836	0.08479	0.08424	0.02472	0.052	0.08955	0.03459	0.05836	0.02441	0.052	0.11496	0.02629	0.06358
0.056	0.04932	0.04723	0.0605	0.04352	0.056	0.06576	0.08324	0.08254	0.02407	0.056	0.08754	0.03393	0.05715	0.02368	0.056	0.11177	0.02514	0.06191
0.06	0.04795	0.04623	0.05958	0.04263	0.06	0.06413	0.08207	0.08151	0.02365	0.06	0.08623	0.03371	0.05629	0.02302	0.06	0.10945	0.02417	0.0603
0.064	0.04644	0.04502	0.05866	0.04165	0.064	0.06185	0.08051	0.07981	0.02295	0.064	0.08468	0.03342	0.05544	0.02215	0.064	0.10757	0.02305	0.05853
0.068	0.04508	0.04375	0.05797	0.04054	0.068	0.0599	0.07896	0.0781	0.02242	0.068	0.08337	0.03294	0.05445	0.02127	0.068	0.10439	0.02165	0.05671
0.072	0.04372	0.04254	0.05705	0.03947	0.072	0.05762	0.0774	0.07708	0.02199	0.072	0.08182	0.03272	0.05338	0.02047	0.072	0.10279	0.02056	0.05544
0.076	0.04259	0.04145	0.05636	0.03849	0.076	0.05566	0.07585	0.07538	0.0214	0.076	0.08056	0.03246	0.05238	0.01967	0.076	0.10062	0.0195	0.05387
0.08	0.04108	0.04027	0.05544	0.03718	0.08	0.05306	0.0739	0.07401	0.02103	0.08	0.07902	0.03195	0.0516	0.01901	0.08	0.09816	0.01828	0.05249
0.084	0.03981	0.03912	0.05475	0.03594	0.084	0.05111	0.07273	0.07231	0.02049	0.084	0.07759	0.03158	0.05039	0.0181	0.084	0.09613	0.01744	0.05117
0.088	0.03854	0.03794	0.05383	0.03499	0.088	0.04915	0.07079	0.07094	0.01985	0.088	0.07604	0.03078	0.04954	0.01726	0.088	0.09367	0.01623	0.0496
0.092	0.03732	0.03688	0.05268	0.03411	0.092	0.04688	0.06962	0.06992	0.01953	0.092	0.07473	0.03016	0.04855	0.01635	0.092	0.09165	0.01532	0.04832
0.096	0.03614	0.03576	0.05176	0.03278	0.096	0.04492	0.06768	0.06821	0.01894	0.096	0.07341	0.02957	0.04784	0.01569	0.096	0.08933	0.0142	0.04705
0.1	0.03482	0.03461	0.05061	0.0316	0.1	0.04264	0.06612	0.06685	0.01835	0.1	0.07209	0.02873	0.04663	0.01496	0.1	0.0873	0.01333	0.04553
0.104	0.03365	0.0334	0.04969	0.03059	0.104	0.04069	0.06418	0.06514	0.01792	0.104	0.07015	0.02799	0.0457	0.01408	0.104	0.0847	0.01237	0.04425
0.108	0.03237	0.03225	0.04854	0.02925	0.108	0.03841	0.06262	0.06344	0.01739	0.108	0.06889	0.02693	0.04478	0.01328	0.108	0.08267	0.01152	0.04278
0.112	0.03129	0.03104	0.04808	0.02824	0.112	0.03646	0.06068	0.06173	0.01685	0.112	0.0674	0.02612	0.04371	0.01255	0.112	0.07992	0.01059	0.04131
0.116	0.03002	0.02989	0.0467	0.0269	0.116	0.03418	0.05873	0.06003	0.01621	0.116	0.06586	0.02521	0.04293	0.01175	0.116	0.07746	0.00962	0.03994
0.12	0.0287	0.02874	0.04555	0.02585	0.12	0.03223	0.05679	0.05798	0.01568	0.12	0.06408	0.02429	0.04165	0.01098	0.12	0.075	0.00891	0.03846

0.124	0.02767	0.0275	0.04417	0.02481	0.124	0.03027	0.05484	0.05662	0.01525	0.124	0.06231	0.02367	0.04066	0.01025	0.124	0.07268	0.0081	0.03719
0.128	0.0264	0.02638	0.04371	0.02347	0.128	0.02832	0.05251	0.05491	0.01461	0.128	0.06065	0.02283	0.0398	0.0096	0.128	0.07051	0.00735	0.03562
0.132	0.02518	0.02517	0.0421	0.0222	0.132	0.02604	0.05056	0.05321	0.01396	0.132	0.05876	0.02198	0.0386	0.00883	0.132	0.06834	0.00645	0.0342
0.136	0.02409	0.02393	0.04141	0.02122	0.136	0.02409	0.04862	0.05116	0.01348	0.136	0.0571	0.02096	0.03753	0.00814	0.136	0.06602	0.00582	0.03267
0.14	0.02268	0.02272	0.04026	0.02001	0.14	0.02246	0.0459	0.04911	0.01295	0.14	0.05499	0.0199	0.03646	0.00755	0.14	0.06356	0.00529	0.0313
0.144	0.0215	0.02157	0.03911	0.0189	0.144	0.02051	0.04395	0.04707	0.01241	0.144	0.05304	0.01924	0.03533	0.00701	0.144	0.06095	0.00467	0.02983
0.148	0.02005	0.02042	0.0375	0.01773	0.148	0.01888	0.04162	0.04536	0.01161	0.148	0.05144	0.01839	0.03419	0.00646	0.148	0.05835	0.00411	0.02826
0.152	0.01863	0.01906	0.03635	0.01671	0.152	0.01725	0.03928	0.04366	0.01118	0.152	0.04949	0.01784	0.03298	0.0058	0.152	0.0556	0.00327	0.02674
0.156	0.01741	0.01779	0.03451	0.01573	0.156	0.01563	0.03695	0.04161	0.0107	0.156	0.04761	0.01704	0.03199	0.00525	0.156	0.05357	0.00293	0.02527
0.16	0.01605	0.01679	0.03359	0.01459	0.16	0.01432	0.03501	0.0399	0.01027	0.16	0.04566	0.01649	0.03085	0.00471	0.16	0.05096	0.00249	0.02394
0.164	0.01478	0.01549	0.03244	0.01338	0.164	0.0127	0.03267	0.03786	0.00952	0.164	0.04371	0.01594	0.02971	0.00412	0.164	0.04836	0.00184	0.02262
0.168	0.01332	0.01425	0.03129	0.01237	0.168	0.01139	0.02995	0.03581	0.00915	0.168	0.04171	0.01491	0.02836	0.00361	0.168	0.04575	0.00146	0.0211
0.172	0.012	0.01331	0.02991	0.01116	0.172	0.01042	0.02762	0.03411	0.00867	0.172	0.03971	0.01458	0.02715	0.0031	0.172	0.04329	0.00112	0.01987
0.176	0.01073	0.0121	0.0283	0.01012	0.176	0.00879	0.02567	0.0324	0.00808	0.176	0.03748	0.01403	0.02609	0.00263	0.176	0.04083	9.34E-04	0.01835
0.18	0.00946	0.01101	0.02669	0.00927	0.18	0.00749	0.02373	0.03035	0.0076	0.18	0.03576	0.01345	0.02474	0.00226	0.18	0.03808	4.36E-04	0.01693
0.184	0.00805	0.0098	0.02553	0.00829	0.184	0.00651	0.02178	0.02831	0.00738	0.184	0.03376	0.01282	0.02338	0.00182	0.184	0.03576	2.80E-04	0.0156
0.188	0.00701	0.0088	0.02369	0.00728	0.188	0.00553	0.01945	0.0266	0.0069	0.188	0.03193	0.01205	0.02239	0.00157	0.188	0.03344	1.87E-04	0.01457
0.192	0.00588	0.00778	0.02231	0.00646	0.192	0.00456	0.01789	0.0249	0.00647	0.192	0.02993	0.01158	0.02097	0.0012	0.192	0.03098	1.87E-04	0.0133
0.196	0.00475	0.00666	0.02093	0.00542	0.196	0.00358	0.01556	0.02319	0.0061	0.196	0.02809	0.01081	0.0199	9.85E-04	0.196	0.02838	0	0.01182
0.2	0.00386	0.00572	0.01932	0.0047	0.2	0.0026	0.014	0.02149	0.00567	0.2	0.02598	0.01026	0.01862	7.66E-04	0.2	0.02621	1.56E-04	0.0107
0.204	0.00315	0.00508	0.01771	0.00382	0.204	0.00195	0.01245	0.02012	0.00546	0.204	0.02443	0.0096	0.01734	6.57E-04	0.204	0.02403	5.92E-04	0.00947
0.208	0.00231	0.00402	0.01587	0.0032	0.208	0.00163	0.0105	0.01842	0.00498	0.208	0.02254	0.00879	0.01621	4.01E-04	0.208	0.02157	7.48E-04	0.00819
0.212	0.00184	0.00318	0.01426	0.00225	0.212	9.77E-04	0.00933	0.01637	0.0046	0.212	0.02048	0.00806	0.01486	2.55E-04	0.212	0.01911	0.00121	0.00702
0.216	0.00113	0.00236	0.01265	0.00176	0.216	6.51E-04	0.00778	0.01501	0.00412	0.216	0.01888	0.00751	0.0135	3.65E-05	0.216	0.01723	0.00178	0.00584
0.22	8.94E-04	0.00169	0.01081	0.00114	0.22	3.26E-04	0.00661	0.01296	0.0038	0.22	0.01722	0.00693	0.01237	0	0.22	0.01506	0.00249	0.00486
0.224	5.18E-04	0.00124	0.00943	7.18E-04	0.224	0	0.00545	0.01126	0.00353	0.224	0.01539	0.00623	0.01116	7.30E-05	0.224	0.01303	0.0034	0.00388
0.228	1.88E-04	7.56E-04	0.00736	1.63E-04	0.228	0	0.00428	0.00989	0.00294	0.228	0.01356	0.00546	0.00988	7.30E-05	0.228	0.01086	0.00445	0.00289
0.232	0	2.72E-04	0.00575	6.53E-05	0.232	3.26E-04	0.00311	0.00853	0.00262	0.232	0.01219	0.0048	0.00888	2.92E-04	0.232	0.00927	0.00582	0.00221
0.236	1.41E-04	6.05E-05	0.00437	0	0.236	9.77E-04	0.00272	0.00682	0.00219	0.236	0.0107	0.00414	0.00761	5.11E-04	0.236	0.00767	0.00732	0.00147
0.24	4.71E-05	0	0.00299	1.96E-04	0.24	0.00163	0.00194	0.0058	0.00182	0.24	0.00921	0.00333	0.00654	8.03E-04	0.24	0.00594	0.00894	8.34E-04
0.244	4.71E-04	9.08E-05	0.00184	5.88E-04	0.244	0.0026	7.78E-04	0.00409	0.00155	0.244	0.00755	0.00282	0.00547	0.00117	0.244	0.00434	0.01087	4.91E-04
0.248	6.12E-04	3.63E-04	9.20E-04	0.00108	0.248	0.00391	3.89E-04	0.00307	0.00102	0.248	0.00618	0.00216	0.00426	0.00161	0.248	0.00492	0.01314	2.45E-04
0.252	0.00118	9.98E-04	4.60E-04	0.00196	0.252	0.00586	0	0.00205	8.02E-04	0.252	0.00492	0.00172	0.00334	0.00237	0.252	0.00217	0.0157	0
0.256	0.0016	0.00203	0	0.00304	0.256	0.00781	0	0.00136	5.35E-04	0.256	0.00372	0.00128	0.0027	0.00314	0.256	0.00145	0.01859	2.94E-04
0.26	0.00259	0.00303	0	0.00424	0.26	0.01042	0	6.82E-04	1.60E-04	0.26	0.00269	6.60E-04	0.00185	0.00405	0.26	5.79E-04	0.02196	4.42E-04
0.264	0.00348	0.00472	4.60E-04	0.00604	0.264	0.01367	3.89E-04	0	0	0.264	0.00189	5.50E-04	0.00121	0.00533	0.264	2.90E-04	0.02566	0.00128
0.268	0.00489	0.00705	0.00138	0.00819	0.268	0.01758	3.89E-04	0	1.07E-04	0.268	9.73E-04	2.93E-04	6.40E-04	0.00675	0.268	0	0.02999	0.00226
0.272	0.0064	0.00968	0.00299	0.01084	0.272	0.02181	0.00117	0	5.35E-05	0.272	5.15E-04	1.10E-04	2.84E-04	0.00839	0.272	0	0.03479	0.00353
0.276	0.00856	0.01298	0.00575	0.01374	0.276	0.02669	0.00194	6.82E-04	2.14E-04	0.276	0	0	7.11E-05	0.01014	0.276	4.34E-04	0.04037	0.00535
0.28	0.01078	0.01697	0.0092	0.01737	0.28	0.03223	0.00311	0.00136	5.35E-04	0.28	5.72E-05	2.20E-04	0	0.01241	0.28	4.34E-04	0.0466	0.00731
0.284	0.01374	0.0219	0.0138	0.02164	0.284	0.03906	0.00467	0.00273	0.00112	0.284	5.15E-04	5.50E-04	2.13E-04	0.01496	0.284	0.00101	0.05376	0.0102
0.288	0.01694	0.02756	0.01978	0.02631	0.288	0.04622	0.007	0.00443	0.00187	0.288	0.00114	5.86E-04	7.82E-04	0.01795	0.288	0.00174	0.06217	0.01354
0.292	0.02113	0.03422	0.02738	0.03206	0.292	0.05436	0.00933	0.00682	0.00267	0.292	0.002	0.00114	0.00149	0.02127	0.292	0.0029	0.07151	0.01751
0.296	0.02579	0.04199	0.03681	0.03842	0.296	0.06315	0.01206	0.00955	0.00417	0.296	0.00349	0.00183	0.00249	0.02499	0.296	0.0042	0.08179	0.02203
0.3	0.0312	0.05098	0.04854	0.0458	0.3	0.07324	0.01556	0.0133	0.00572	0.3	0.00532	0.00326	0.0037	0.02937	0.3	0.00565	0.09363	0.02723
0.304	0.0375	0.06111	0.06188	0.05396	0.304	0.08398	0.01984	0.01739	0.00765	0.304	0.00801	0.00451	0.00533	0.03415	0.304	0.00724	0.10671	0.03331
0.308	0.04428	0.0724	0.07706	0.06343	0.308	0.0957	0.02412	0.02217	0.00984	0.308	0.01093	0.00634	0.00718	0.03952	0.308	0.00927	0.12073	0.03998
0.312	0.05186	0.0845	0.09478	0.07355	0.312	0.1084	0.02878	0.02763	0.01273	0.312	0.01453	0.0085	0.0096	0.04543	0.312	0.01129	0.13568	0.04739
0.316	0.06047	0.09812	0.11502	0.08497	0.316	0.12174	0.03462	0.03411	0.01594	0.316	0.01877	0.01114	0.01251	0.05214	0.316	0.01433	0.15187	0.05608
0.32	0.0695	0.11233	0.13757	0.09705	0.32	0.13607	0.04123	0.04093	0.01958	0.32	0.02375	0.01392	0.01535	0.05947	0.32	0.01752	0.16869	0.06481
0.324	0.07929	0.12716	0.16195	0.11011	0.324	0.15039	0.04784	0.04809	0.02349	0.324	0.02918	0.01733	0.01876	0.06739	0.324	0.02056	0.18582	0.07442
0.328	0.08978	0.14289	0.18841	0.12415	0.328	0.16602	0.05562	0.05593	0.02809	0.328	0.03548	0.02096	0.02267	0.07622	0.328	0.02447	0.20326	0.08453
0.332	0.10047	0.15832	0.21532	0.13884	0.332	0.18164	0.06301	0.06378	0.03312	0.332	0.04217	0.02514	0.02729	0.08564	0.332	0.02881	0.22071	0.09528
0.336	0.11138	0.17436	0.24262	0.15418	0.336	0.19759	0.07157	0.07196	0.03868	0.336	0.04938	0.02983	0.03191	0.09592	0.336	0.03301	0.23784	0.10636
0.34	0.1223	0.18979	0.27214	0.16952	0.34	0.21322	0.07974	0.08049	0.04478	0.34	0.05699	0.03514	0.0371	0.10705	0.34	0.03793	0.25434	0.11789
0.344	0.13322	0.20491	0.29952	0.18519	0.344	0.22884	0.08829	0.08902	0.05136	0.344	0.06511	0.04078	0.04265	0.11898	0.344	0.04242	0.27023	0.12913
0.348	0.14399	0.21944	0.3262	0.20086	0.348	0.24414	0.09724	0.09823	0.05815	0.348	0.07364	0.0473	0.04869	0.1319	0.348	0.04763	0.2858	0.14066
0.352	0.15477	0.23335	0.35059	0.21718	0.352	0.25911	0.10618	0.10744	0.06538	0.352	0.08239	0.0543	0.05494	0.14591	0.352	0.05256	0.30107	0.15209
0.356	0.16475	0.24666	0.37198	0.23351	0.356	0.27376	0.11552	0.11733	0.07329	0.356	0.09098	0.06156	0.06148	0.16109	0.356	0.05762	0.31539	0.16347
0.36	0.17477	0.25967	0.3913	0.25113	0.36	0.28809	0.12447	0.1279	0.08175	0.36	0.10025	0.07035	0.06866	0.17787	0.36	0.06269	0.33034	0.1748
0.364	0.18484	0.27329	0.40856	0.27039	0.364	0.30371	0.13497	0.14086	0.09105	0.364	0.10986	0.0812	0.07634	0.19688	0.364	0.06834	0.34623	0.18707
0.368	0.19434	0.2869	0.42282	0.29096	0.368	0.31868	0.14508	0.15484	0.1009	0.368	0.11924	0.09263	0.08416	0.21732	0.368	0.07326	0.36305	0.19884
0.372	0.20394	0.30173	0.43501	0.31414	0.372	0.33464	0.15636	0.17087	0.11128	0.372	0.12914	0.10597	0.09269	0.24034	0.372	0.07804	0.38111	0.2116
0.376	0.21397	0.31806	0.44582	0.34123	0.376	0.35189	0.16919	0.18963	0.12315	0.376	0.13933	0.12062	0.10178	0.26625	0.376	0.08296	0.40167	0.22484
0.38	0.22479	0.33652	0.45687	0.3729	0.38	0.37109	0.18359	0.21146	0.13599	0.38	0.1498	0.13784	0.11202	0.29617	0.38	0.08817	0.42503	0.24005
0.384	0.23646	0.3577	0.46837	0.40946	0.384	0.3929	0.19992	0.23636	0.15012	0.384	0.16135	0.15726	0.12318	0.32973	0.384	0.09353	0.45119	0.25673
0.388	0.24898	0.3819	0.48263	0.45125	0.388	0.41667	0.2182	0.26398	0.16536	0.388	0.17308	0.17778	0.1354	0.36659	0.388	0.09932	0.47954	0.27587
0.392	0.26309	0.40943	0.50081	0.49923	0.392	0.44368	0.2396	0.29434	0.18184	0.392	0.18624	0.2005	0.14905	0.40782	0.392	0.10583	0.51099	0.29696
0.396	0.27815	0.43969	0.52358	0.55244	0.396	0.47363	0.26371	0.32674	0.19923	0.396	0.19981	0.22431	0.16405	0.45196	0.396	0.11322	0.54401	0.321
0.4	0.29462	0.47267	0.55095	0.60957	0.4	0.50521	0.29016	0.36085	0.21726	0.4	0.21417	0.24886	0.18068	0.49867	0.4	0.12118	0.57765	0.3475
0.404	0.31203	0.50655	0.58385	0.66996	0.404	0.53906	0.31855	0.39529	0.23529	0.404	0.2295	0.27268	0.19909	0.5472	0.404	0.12958	0.61035	0.37595
0.408	0.32944	0.54165	0.62181	0.73101	0.408	0.57292	0.34811	0.42872	0.25251	0.408	0.24472	0.29576	0.21878	0.59536	0.408	0.13884	0.64181	0.40637
0.412	0.34732	0.57583	0.66368	0.79107	0.412	0.60677	0.37884	0.45975	0.26814	0.412	0.25988	0.31702	0.24003	0.64206	0.412	0.14898	0.66984	0.43777
0.416	0.36427	0.6073	0.70807	0.84657	0.416	0.63835	0.40879	0.48738	0.2814	0.416	0.27482	0.3346	0.26185	0.68512	0.416	0.15955	0.69258	0.46917
0.42	0.37979	0.63513	0.75408	0.89456	0.42	0.66634	0.43718	0.51023	0.29173	0.42	0.28861	0.34816	0.28438	0.72233	0.42	0.1707	0.70878	0.49958
0.424	0.39438	0.65843	0.80009	0.93341	0.424	0.68978	0.46247	0.52729	0.29842	0.424	0.30142	0.35695	0.30727	0.75225	0.424	0.18228	0.71843	0.52853
0.428	0.40568	0.67507	0.84403	0.95985	0.428	0.70703	0.48347	0.53752	0.3012	0.428	0.3123	0.36025	0.32938	0.77232	0.428	0.19357	0.71999	0.55404
0.432	0.41462	0.68475	0.88337	0.97258	0.432	0.7168	0.49942	0.54093	0.29991	0.432	0.32025	0.35842	0.34992	0.78144	0.432	0.20414	0.71376	0.57563
0.436	0.41979	0.68626	0.91672	0.96997	0.436	0.71842	0.50836	0.53683	0.29456	0.436	0.32597	0.35146	0.36854	0.77925	0.436	0.21413	0.69974	0.59182
0.44	0.42262	0.67991	0.94203	0.95267	0.44	0.71191	0.5107	0.52694	0.2859	0.44	0.32883	0.33937	0.38404	0.76575	0.44	0.22267	0.67919	0.60163
0.444	0.42167	0.6669	0.95905	0.92231	0.444	0.69792	0.50642	0.51126	0.27429	0.444	0.3294	0.32434	0.3964	0.74204	0.444	0.23049	0.65334	0.60555
0.448	0.41744	0.64663	0.96618	0.87954	0.448	0.67611	0.49514	0.49079	0.26033	0.448	0.32654	0.30639	0.40479	0.70993	0.448	0.23643	0.62312	0.60261
0.452	0.40991	0.62061	0.96342	0.82764	0.452	0.64811	0.47802	0.46623	0.24508	0.452	0.32139	0.2866	0.40913	0.67052	0.452	0.2412	0.5898	0.5928
0.456	0.40003	0.59096	0.95123	0.7692	0.456	0.61523	0.45663	0.43963	0.22924	0.456	0.31396	0.26609	0.40948	0.62674	0.456	0.24396	0.55585	0.57759
0.46	0.38826	0.55829	0.93122	0.70783	0.46	0.5791	0.43174	0.41132	0.21383	0.46	0.30457	0.24593	0.4065	0.58149	0.46	0.2454	0.52158	0.55747
0.464	0.37556	0.5241	0.90292	0.64581	0.464	0.54036	0.40412	0.38233	0.19875	0.464	0.29387	0.22615	0.4001	0.53661	0.464	0.24497	0.48763	0.53393
0.468	0.3605	0.48991	0.8698	0.58541	0.468	0.5013	0.37534	0.35402	0.18484	0.468	0.28168	0.20746	0.391	0.49392	0.468	0.24323	0.45618	0.50792
0.472	0.34544	0.45603	0.8323	0.52763	0.472	0.46224	0.34617	0.32572	0.17227	0.472	0.26881	0.18987	0.37963	0.45488	0.472	0.23947	0.42628	0.48045
0.476	0.32991	0.42396	0.79273	0.47508	0.476	0.4248	0.31816	0.29877	0.16108	0.476	0.25514	0.17412	0.36698	0.42059	0.476	0.23527	0.39855	0.45248
0.48	0.31391	0.394	0.75224	0.42709	0.48	0.38932	0.29133	0.27319	0.15113	0.48	0.24163	0.15983	0.35418	0.3903	0.48	0.22991	0.37395	0.42501
0.484	0.29839	0.36617	0.71245	0.384	0.484	0.35645	0.26643	0.24932	0.14241	0.484	0.22773	0.147	0.34068	0.36476	0.484	0.22412	0.35152	0.39901
0.488	0.2838	0.34106	0.67426	0.34678	0.488	0.32617	0.24349	0.22749	0.13503	0.488	0.21422	0.13564	0.32767	0.3436	0.488	0.21775	0.3319	0.37497
0.492	0.26921	0.31867	0.63883	0.31446	0.492	0.2998	0.22287	0.20771	0.12877	0.492	0.20129	0.12612	0.31537	0.32608	0.492	0.21225	0.31446	0.35289
0.496	0.25604	0.2987	0.60524	0.28704	0.496	0.27637	0.20498	0.18997	0.12353	0.496	0.18871	0.11732	0.30407	0.31149	0.496	0.20646	0.2992	0.33327
0.5	0.24342	0.28115	0.57488	0.26387	0.5	0.25553	0.18864	0.17428	0.11909	0.5	0.17703	0.11	0.29398	0.29981	0.5	0.2011	0.28611	0.3161
0.504	0.23166	0.26603	0.54796	0.24461	0.504	0.23796	0.17425	0.1603	0.11534	0.504	0.16616	0.1034	0.28495	0.29069	0.504	0.19618	0.2749	0.30089
0.508	0.22121	0.25302	0.5245	0.22926	0.508	0.22331	0.16219	0.14836	0.11262	0.508	0.15649	0.0979	0.27749	0.28376	0.508	0.19212	0.26587	0.28813
0.512	0.21194	0.24213	0.50403	0.21686	0.512	0.21061	0.15208	0.13813	0.11042	0.512	0.14745	0.09321	0.27131	0.27829	0.512	0.18821	0.25808	0.27734
0.516	0.20343	0.23305	0.48585	0.20706	0.516	0.2002	0.14352	0.1296	0.1085	0.516	0.1395	0.08911	0.26597	0.27391	0.516	0.18488	0.25123	0.26802
0.52	0.19637	0.22579	0.4709	0.19988	0.52	0.19206	0.13613	0.12244	0.10743	0.52	0.13246	0.08592	0.26178	0.27135	0.52	0.18271	0.24656	0.26115
0.524	0.19016	0.22004	0.45871	0.19466	0.524	0.18587	0.13069	0.11664	0.10673	0.524	0.12628	0.08336	0.25901	0.26989	0.524	0.18098	0.24282	0.25526
0.528	0.18493	0.2155	0.44836	0.19139	0.528	0.18132	0.12602	0.11255	0.1063	0.528	0.12119	0.08138	0.25709	0.26916	0.528	0.17996	0.24002	0.25134
0.532	0.18056	0.21248	0.44053	0.18976	0.532	0.17806	0.12252	0.10914	0.10636	0.532	0.11701	0.07984	0.25602	0.26916	0.532	0.17909	0.23784	0.24839

0.536	0.17731	0.21036	0.43432	0.18944	0.536	0.17611	0.12019	0.10675	0.10673	0.536	0.11358	0.07874	0.25581	0.26989	0.536	0.17895	0.2369	0.24643
0.54	0.17486	0.20945	0.42995	0.19042	0.54	0.17546	0.11902	0.10539	0.10737	0.54	0.11089	0.07823	0.25638	0.27135	0.54	0.17924	0.2369	0.24594
0.544	0.17317	0.20945	0.42719	0.19303	0.544	0.17578	0.11824	0.10505	0.10834	0.544	0.10877	0.07804	0.25766	0.27354	0.544	0.1804	0.23721	0.24594
0.548	0.17176	0.21006	0.42535	0.19597	0.548	0.17676	0.11824	0.10471	0.10962	0.548	0.1074	0.0779	0.25972	0.27573	0.548	0.18127	0.23815	0.24692
0.552	0.17143	0.21127	0.42512	0.19956	0.552	0.17839	0.11902	0.10539	0.11106	0.552	0.10654	0.07845	0.26228	0.27865	0.552	0.183	0.23971	0.24888
0.556	0.17162	0.21338	0.42581	0.20445	0.556	0.18099	0.12058	0.10607	0.11272	0.556	0.10631	0.07911	0.26576	0.28194	0.556	0.18517	0.24189	0.25134
0.56	0.17223	0.2158	0.42742	0.20935	0.56	0.18424	0.12213	0.10778	0.11459	0.56	0.10631	0.07991	0.26967	0.28558	0.56	0.18706	0.24407	0.25379
0.564	0.17331	0.21853	0.42949	0.2149	0.564	0.18783	0.12408	0.10914	0.11647	0.564	0.10671	0.0809	0.27422	0.2896	0.564	0.18966	0.24656	0.25673
0.568	0.17444	0.22186	0.43179	0.22045	0.568	0.19173	0.1268	0.11119	0.11845	0.568	0.10757	0.08204	0.2787	0.29361	0.568	0.19241	0.24936	0.26017
0.572	0.17594	0.22549	0.4357	0.22665	0.572	0.19629	0.12952	0.11357	0.12059	0.572	0.10894	0.08336	0.28382	0.29835	0.572	0.19589	0.25248	0.26409
0.576	0.17778	0.22912	0.43892	0.23285	0.576	0.20052	0.13224	0.11596	0.1231	0.576	0.11037	0.08468	0.28915	0.3031	0.576	0.19907	0.25559	0.26802
0.58	0.17971	0.23275	0.44214	0.23873	0.58	0.20508	0.13497	0.11801	0.12535	0.58	0.1118	0.08611	0.29448	0.30748	0.58	0.2024	0.25839	0.27194
0.584	0.18183	0.23668	0.44651	0.24461	0.584	0.20996	0.13847	0.12108	0.12781	0.584	0.11392	0.08768	0.30038	0.31258	0.584	0.20617	0.26182	0.27587
0.588	0.18413	0.24031	0.45043	0.25048	0.588	0.21484	0.14236	0.12347	0.13048	0.588	0.11575	0.089	0.30656	0.31733	0.588	0.21008	0.26493	0.28028
0.592	0.18653	0.24424	0.45411	0.2557	0.592	0.2194	0.14508	0.12585	0.13305	0.592	0.11781	0.09072	0.3126	0.32244	0.592	0.21428	0.26774	0.28421
0.596	0.18879	0.24818	0.45756	0.26125	0.596	0.22396	0.14858	0.12858	0.13573	0.596	0.11999	0.0923	0.319	0.32718	0.596	0.21847	0.27085	0.28813
0.6	0.19119	0.25181	0.46101	0.26582	0.6	0.22819	0.15169	0.13097	0.13851	0.6	0.12227	0.09391	0.32547	0.33192	0.6	0.22267	0.27366	0.29206
0.604	0.19354	0.25544	0.46446	0.27039	0.604	0.23275	0.15519	0.1340	0.14124	0.604	0.12462	0.0956	0.33201	0.33703	0.604	0.22687	0.27646	0.29549
0.608	0.19613	0.25877	0.46791	0.27464	0.608	0.23698	0.15869	0.13677	0.14413	0.608	0.12714	0.09717	0.33869	0.34177	0.608	0.23121	0.27895	0.29893
0.612	0.1983	0.26209	0.47044	0.27823	0.612	0.24056	0.1618	0.1395	0.1468	0.612	0.12931	0.099	0.34516	0.34615	0.612	0.23527	0.28144	0.30187
0.616	0.2007	0.26512	0.47343	0.28182	0.616	0.24414	0.16453	0.14188	0.1498	0.616	0.13154	0.10084	0.3517	0.35053	0.616	0.23976	0.28362	0.30481
0.62	0.20291	0.26845	0.47619	0.28508	0.62	0.2474	0.16764	0.14461	0.15269	0.62	0.13389	0.1023	0.35816	0.35454	0.62	0.2441	0.2858	0.30727
0.624	0.20512	0.27117	0.47826	0.2877	0.624	0.25065	0.17075	0.147	0.15574	0.624	0.13629	0.10413	0.3647	0.35856	0.624	0.24844	0.28798	0.30972
0.628	0.20728	0.27389	0.48056	0.29031	0.628	0.25326	0.17347	0.14973	0.15841	0.628	0.13858	0.1056	0.37096	0.36257	0.628	0.25264	0.28985	0.31168
0.632	0.20945	0.27662	0.4824	0.29227	0.632	0.25553	0.17581	0.15211	0.1613	0.632	0.14081	0.10743	0.37721	0.36622	0.632	0.25713	0.29172	0.31364
0.636	0.21152	0.27934	0.48401	0.29423	0.636	0.25814	0.17892	0.15484	0.16408	0.636	0.14333	0.10926	0.3834	0.3695	0.636	0.26162	0.29359	0.31561
0.64	0.21359	0.28176	0.48562	0.29618	0.64	0.26074	0.18164	0.15757	0.16697	0.64	0.14585	0.11109	0.38937	0.37315	0.64	0.26567	0.29546	0.31708
0.644	0.21561	0.28388	0.48677	0.29716	0.644	0.26237	0.18398	0.15996	0.16964	0.644	0.14808	0.11329	0.39512	0.37607	0.644	0.26944	0.2967	0.31806
0.648	0.21783	0.2863	0.48815	0.2988	0.648	0.26465	0.18631	0.16269	0.17221	0.648	0.15048	0.11549	0.40074	0.37899	0.648	0.2732	0.29857	0.31953
0.652	0.2199	0.28872	0.48838	0.29977	0.652	0.2666	0.18903	0.16542	0.17478	0.652	0.153	0.11696	0.4065	0.38191	0.652	0.27711	0.30013	0.32051
0.656	0.2222	0.29084	0.48953	0.30075	0.656	0.26823	0.19137	0.16814	0.17719	0.656	0.15546	0.11879	0.41183	0.38483	0.656	0.28073	0.302	0.32149
0.66	0.22432	0.29265	0.48976	0.30141	0.66	0.26953	0.1937	0.17053	0.17938	0.66	0.15815	0.12026	0.41695	0.38702	0.66	0.28406	0.30356	0.32247
0.664	0.22653	0.29477	0.49068	0.30239	0.664	0.27116	0.19564	0.17326	0.18147	0.664	0.16055	0.12172	0.42213	0.38957	0.664	0.28768	0.30511	0.32395
0.668	0.22884	0.29719	0.4916	0.30304	0.668	0.27279	0.19837	0.17633	0.18366	0.668	0.1633	0.12319	0.42675	0.39176	0.668	0.29115	0.30667	0.32493
0.672	0.23142	0.29961	0.49229	0.30402	0.672	0.27441	0.20109	0.1794	0.1858	0.672	0.16588	0.12502	0.4318	0.39432	0.672	0.29477	0.30885	0.3264
0.676	0.23401	0.30142	0.49229	0.30467	0.676	0.27604	0.20303	0.18179	0.18751	0.676	0.16839	0.12648	0.43663	0.3965	0.676	0.29752	0.31041	0.32738
0.68	0.23637	0.30354	0.49298	0.30565	0.68	0.27767	0.20576	0.18486	0.18949	0.68	0.17091	0.12795	0.44118	0.39869	0.68	0.30042	0.31259	0.32934
0.684	0.23909	0.30596	0.49344	0.3063	0.684	0.27897	0.20809	0.18793	0.1912	0.684	0.17366	0.12978	0.44538	0.40052	0.684	0.30361	0.31477	0.33032
0.688	0.24206	0.30838	0.49413	0.30761	0.688	0.28092	0.21081	0.19065	0.19286	0.688	0.17657	0.13125	0.44964	0.40271	0.688	0.30621	0.31695	0.33229
0.692	0.24521	0.3108	0.49413	0.30892	0.692	0.28255	0.21315	0.19372	0.19458	0.692	0.17932	0.13308	0.45348	0.40453	0.692	0.30896	0.31913	0.33425
0.696	0.24804	0.31322	0.49505	0.31022	0.696	0.28451	0.21626	0.19714	0.19629	0.696	0.18195	0.13491	0.45732	0.40672	0.696	0.31171	0.32162	0.3367
0.7	0.25133	0.31595	0.49597	0.31185	0.7	0.28678	0.21898	0.2002	0.19827	0.7	0.1847	0.13674	0.4613	0.40855	0.7	0.31432	0.32474	0.33965
0.704	0.25462	0.31897	0.49689	0.31381	0.704	0.28906	0.22209	0.20396	0.20003	0.704	0.18773	0.13894	0.46507	0.41073	0.704	0.31692	0.32785	0.34259
0.708	0.25839	0.3217	0.49781	0.31577	0.708	0.29167	0.22482	0.20771	0.20185	0.708	0.19048	0.14151	0.46841	0.41256	0.708	0.31895	0.33097	0.34553
0.712	0.26262	0.32502	0.49965	0.31838	0.712	0.2946	0.22832	0.21146	0.2041	0.712	0.19328	0.14407	0.4721	0.41511	0.712	0.32156	0.3347	0.34946
0.716	0.26639	0.32835	0.50127	0.32132	0.716	0.29785	0.23182	0.21555	0.20618	0.716	0.19603	0.147	0.47558	0.4173	0.716	0.32373	0.33875	0.35387
0.72	0.27062	0.33198	0.50311	0.32426	0.72	0.30111	0.23532	0.21999	0.20854	0.72	0.19912	0.1503	0.47885	0.41986	0.72	0.32634	0.3428	0.35829
0.724	0.27533	0.33531	0.50495	0.32752	0.724	0.30469	0.2396	0.22408	0.21089	0.724	0.20227	0.1536	0.48198	0.42241	0.724	0.32836	0.34747	0.36319
0.728	0.28003	0.33955	0.50725	0.33144	0.728	0.30859	0.24387	0.22885	0.21362	0.728	0.20558	0.15763	0.48525	0.42496	0.728	0.33068	0.35246	0.36859
0.732	0.28474	0.34348	0.50978	0.33568	0.732	0.31315	0.24854	0.23363	0.2164	0.732	0.2089	0.16203	0.48859	0.42825	0.732	0.33314	0.35775	0.37448
0.736	0.28992	0.34802	0.51277	0.34025	0.736	0.31803	0.2536	0.23874	0.21961	0.736	0.21245	0.16679	0.49207	0.43117	0.736	0.33546	0.36367	0.38086
0.74	0.29509	0.35255	0.5153	0.34482	0.74	0.32292	0.25865	0.24318	0.22266	0.74	0.21628	0.17155	0.4952	0.43445	0.74	0.33806	0.36959	0.38674
0.744	0.30074	0.35709	0.51852	0.35037	0.744	0.32845	0.2641	0.24829	0.22641	0.744	0.22	0.17741	0.49854	0.4381	0.744	0.34081	0.37582	0.39361
0.748	0.30592	0.36224	0.52174	0.35592	0.748	0.33398	0.27032	0.25341	0.23031	0.748	0.22424	0.18328	0.50195	0.44211	0.748	0.34342	0.38267	0.40097
0.752	0.31156	0.36738	0.52519	0.3618	0.752	0.34017	0.27577	0.25853	0.23454	0.752	0.22853	0.18951	0.50515	0.44613	0.752	0.34646	0.38983	0.40882
0.756	0.31721	0.37222	0.52841	0.36735	0.756	0.34635	0.2816	0.2633	0.23877	0.756	0.23265	0.1961	0.50871	0.45051	0.756	0.3495	0.39637	0.41618
0.76	0.32238	0.37736	0.53163	0.37322	0.76	0.35254	0.28744	0.26842	0.24342	0.76	0.23734	0.20306	0.51247	0.45561	0.76	0.35211	0.40323	0.42354
0.764	0.32756	0.3822	0.53508	0.3791	0.764	0.35872	0.29405	0.27319	0.24829	0.764	0.24175	0.21039	0.51624	0.46036	0.764	0.35558	0.41008	0.4309
0.768	0.33321	0.38735	0.53807	0.3853	0.768	0.36556	0.30027	0.27831	0.25337	0.768	0.24672	0.21809	0.52036	0.46583	0.768	0.35862	0.41724	0.43826
0.772	0.33791	0.39189	0.54083	0.3902	0.772	0.37142	0.30611	0.2824	0.25861	0.772	0.25124	0.22578	0.5242	0.47094	0.772	0.36181	0.42316	0.44513
0.776	0.34356	0.39673	0.54428	0.39673	0.776	0.37858	0.31311	0.28786	0.2645	0.776	0.25662	0.23384	0.52946	0.47714	0.776	0.36601	0.43032	0.45248
0.78	0.3478	0.40096	0.54658	0.4013	0.78	0.38444	0.31894	0.29195	0.26969	0.78	0.26143	0.2419	0.53373	0.48298	0.78	0.36948	0.43593	0.45886
0.784	0.35156	0.4049	0.54842	0.40587	0.784	0.3903	0.32478	0.29638	0.27525	0.784	0.26629	0.2496	0.53799	0.48845	0.784	0.3731	0.44123	0.46426
0.788	0.35532	0.40792	0.54957	0.40946	0.788	0.39518	0.32983	0.30014	0.28039	0.788	0.27087	0.25729	0.54155	0.49392	0.788	0.37672	0.44559	0.46917
0.792	0.35815	0.41064	0.55049	0.41272	0.792	0.39974	0.33489	0.30423	0.28547	0.792	0.27539	0.26499	0.54581	0.4994	0.792	0.38063	0.44995	0.47358
0.796	0.36097	0.41337	0.55118	0.41566	0.796	0.4043	0.33956	0.30798	0.29066	0.796	0.28037	0.27195	0.55007	0.50487	0.796	0.38439	0.45368	0.47702
0.8	0.36332	0.41548	0.55141	0.41795	0.8	0.40788	0.34384	0.31173	0.29553	0.8	0.28483	0.27891	0.55434	0.50998	0.8	0.38845	0.45711	0.47996
0.804	0.36521	0.41669	0.55072	0.41991	0.804	0.41081	0.34734	0.31514	0.29975	0.804	0.28901	0.28514	0.5586	0.51436	0.804	0.39236	0.45929	0.48192
0.808	0.36662	0.4179	0.55003	0.42154	0.808	0.41374	0.35123	0.31821	0.30377	0.808	0.2933	0.29137	0.56216	0.51874	0.808	0.39641	0.46147	0.48388
0.812	0.36756	0.41881	0.54865	0.42284	0.812	0.41634	0.35434	0.32128	0.30767	0.812	0.29753	0.2965	0.56571	0.52275	0.812	0.4009	0.46303	0.48437
0.816	0.36803	0.41911	0.54681	0.42382	0.816	0.41829	0.35706	0.32469	0.31115	0.816	0.30137	0.30126	0.56927	0.5264	0.816	0.40466	0.46427	0.48486
0.82	0.3685	0.41911	0.54451	0.42415	0.82	0.41927	0.35939	0.32708	0.31398	0.82	0.3048	0.30602	0.57211	0.52932	0.82	0.40843	0.4649	0.48388
0.824	0.36803	0.41881	0.54175	0.42382	0.824	0.42025	0.36134	0.32913	0.31629	0.824	0.30778	0.30932	0.57424	0.53151	0.824	0.41161	0.4649	0.48241
0.828	0.36756	0.41851	0.53899	0.42448	0.828	0.42122	0.36328	0.33186	0.31848	0.828	0.31098	0.31299	0.57708	0.53333	0.828	0.41624	0.46521	0.48143
0.832	0.36756	0.4179	0.536	0.42448	0.832	0.42188	0.36523	0.33424	0.32019	0.832	0.3139	0.31555	0.57922	0.53479	0.832	0.42044	0.46552	0.47996
0.836	0.36662	0.4173	0.53255	0.42448	0.836	0.42188	0.36639	0.33595	0.32137	0.836	0.31624	0.31812	0.58064	0.53552	0.836	0.42435	0.46521	0.47751
0.84	0.36615	0.41669	0.52956	0.42415	0.84	0.42253	0.36795	0.33868	0.32244	0.84	0.31911	0.32031	0.58206	0.53625	0.84	0.4287	0.46521	0.47505
0.844	0.36568	0.41609	0.52588	0.42448	0.844	0.42285	0.36912	0.34038	0.32281	0.844	0.32082	0.32178	0.58277	0.53661	0.844	0.43275	0.4649	0.4726
0.848	0.36474	0.41518	0.5222	0.42448	0.848	0.42318	0.37028	0.34175	0.32319	0.848	0.32254	0.32324	0.58348	0.53661	0.848	0.4368	0.46459	0.47015
0.852	0.36427	0.41458	0.51875	0.42448	0.852	0.42318	0.37106	0.34345	0.32303	0.852	0.32425	0.32434	0.58419	0.53588	0.852	0.44057	0.46396	0.46769
0.856	0.36379	0.41397	0.51507	0.42448	0.856	0.42318	0.37184	0.34482	0.32287	0.856	0.3254	0.32544	0.5849	0.53552	0.856	0.44433	0.46396	0.46524
0.86	0.36332	0.41367	0.51162	0.42513	0.86	0.42383	0.37262	0.34652	0.32222	0.86	0.32654	0.32618	0.5849	0.53443	0.86	0.44853	0.46427	0.46279
0.864	0.36332	0.41367	0.5084	0.42546	0.864	0.42415	0.3734	0.34789	0.32174	0.864	0.32769	0.32654	0.5849	0.5337	0.864	0.45273	0.46427	0.46033
0.868	0.36285	0.41367	0.50495	0.42578	0.868	0.42415	0.37417	0.34891	0.32083	0.868	0.32826	0.32691	0.5849	0.5326	0.868	0.45649	0.46459	0.45788
0.872	0.36332	0.41397	0.5015	0.42676	0.872	0.42448	0.37495	0.35027	0.32008	0.872	0.32883	0.32728	0.58419	0.53187	0.872	0.4604	0.4649	0.45641
0.876	0.36379	0.41458	0.49896	0.42774	0.876	0.42546	0.37612	0.35198	0.31928	0.876	0.3294	0.32801	0.58419	0.53151	0.876	0.46489	0.46614	0.45494
0.88	0.36474	0.41548	0.49574	0.42905	0.88	0.42611	0.37729	0.353	0.31869	0.88	0.32998	0.32837	0.58419	0.53078	0.88	0.46837	0.46708	0.45347
0.884	0.36568	0.41639	0.49298	0.43003	0.884	0.42676	0.37767	0.35437	0.31789	0.884	0.32998	0.32874	0.58348	0.53005	0.884	0.47184	0.46801	0.45199
0.888	0.36662	0.4173	0.49022	0.43133	0.888	0.42773	0.37884	0.35539	0.31725	0.888	0.33055	0.32911	0.58277	0.52932	0.888	0.47531	0.46957	0.4515
0.892	0.36897	0.41911	0.48792	0.43329	0.892	0.42904	0.38001	0.35675	0.31687	0.892	0.33055	0.33021	0.58206	0.52895	0.892	0.47908	0.47144	0.4515
0.896	0.37085	0.42123	0.48585	0.43492	0.896	0.43034	0.38156	0.35812	0.31671	0.896	0.33169	0.33131	0.58206	0.52895	0.896	0.48197	0.47331	0.45101
0.9	0.37274	0.42335	0.48378	0.43688	0.9	0.43164	0.38273	0.35948	0.31677	0.9	0.33169	0.33241	0.58135	0.52859	0.9	0.48473	0.47549	0.4515
0.904	0.37556	0.42577	0.48217	0.43949	0.904	0.43327	0.38429	0.36085	0.31719	0.904	0.33227	0.33387	0.58064	0.52895	0.904	0.48777	0.47829	0.45248
0.908	0.37838	0.4288	0.48079	0.44178	0.908	0.43522	0.38623	0.36255	0.3181	0.908	0.33284	0.3357	0.58064	0.52932	0.908	0.49095	0.48141	0.45347
0.912	0.38168	0.43182	0.47941	0.44472	0.912	0.43685	0.38779	0.36392	0.31891	0.912	0.33398	0.33717	0.58064	0.53005	0.912	0.49399	0.48452	0.45494
0.916	0.38497	0.43515	0.47803	0.44765	0.916	0.43913	0.38973	0.36562	0.32019	0.916	0.33513	0.33937	0.57993	0.53114	0.916	0.49645	0.48763	0.4569
0.92	0.38873	0.43848	0.47757	0.45059	0.92	0.44141	0.39207	0.36767	0.32196	0.92	0.33627	0.34193	0.58064	0.53224	0.92	0.49935	0.49137	0.45886
0.924	0.39203	0.44241	0.47734	0.45418	0.924	0.44401	0.3944	0.36971	0.3241	0.924	0.33741	0.3445	0.58064	0.53406	0.924	0.50195	0.49511	0.46181
0.928	0.39579	0.44574	0.47688	0.4581	0.928	0.44661	0.39712	0.3721	0.3264	0.928	0.33913	0.34743	0.58064	0.53588	0.928	0.50427	0.49916	0.46426
0.932	0.39862	0.44907	0.47688	0.46169	0.932	0.44922	0.39946	0.37449	0.32907	0.932	0.34085	0.35073	0.58064	0.53771	0.932	0.50615	0.50321	0.46671
0.936	0.40191	0.453	0.47734	0.46594	0.936	0.45247	0.40296	0.37722	0.3318	0.936	0.34314	0.35402	0.58064	0.54026	0.936	0.50876	0.50757	0.46966
0.94	0.4052	0.45603	0.4778	0.47083	0.94	0.45573	0.40646	0.38063	0.33522	0.94	0.34543	0.35842	0.58135	0.54318	0.94	0.51137	0.51224	0.4726
0.944	0.40803	0.45935	0.47895	0.47573	0.944	0.45931	0.40957	0.3837	0.33865	0.944	0.34771	0.36208	0.58206	0.5461	0.944	0.51354	0.5166	0.47603

0.948	0.41038	0.46208	0.4801	0.4803	0.948	0.46289	0.41346	0.38677	0.34229	0.948	0.35	0.36611	0.58277	0.54975	0.948	0.51585	0.52127	0.47849	
0.952	0.41226	0.4645	0.48148	0.48585	0.952	0.46712	0.41696	0.39052	0.34614	0.952	0.35286	0.37088	0.58348	0.5534	0.952	0.51759	0.52626	0.48143	
0.956	0.41415	0.46692	0.48332	0.4914	0.956	0.47103	0.42163	0.39427	0.35047	0.956	0.35572	0.37564	0.5849	0.55778	0.956	0.52005	0.53124	0.48437	
0.96	0.41556	0.46903	0.48562	0.49727	0.96	0.47559	0.4259	0.3987	0.35459	0.96	0.35916	0.38077	0.58632	0.56179	0.96	0.52251	0.53654	0.48683	
0.964	0.4165	0.47145	0.48746	0.50348	0.964	0.48014	0.43018	0.4028	0.35898	0.964	0.36202	0.3859	0.58775	0.56653	0.964	0.5244	0.54121	0.48977	
0.968	0.41744	0.47327	0.49045	0.50968	0.968	0.48535	0.43524	0.40791	0.36379	0.968	0.36545	0.39176	0.58917	0.57128	0.968	0.52686	0.54619	0.49222	
0.972	0.41791	0.47539	0.49321	0.51653	0.972	0.49056	0.4403	0.41269	0.36871	0.972	0.36888	0.39726	0.5913	0.57675	0.972	0.52903	0.55149	0.49517	
0.976	0.41885	0.47751	0.49643	0.52339	0.976	0.49609	0.44574	0.4178	0.37374	0.976	0.37289	0.40349	0.59343	0.58222	0.976	0.53192	0.55678	0.49762	
0.98	0.41932	0.47962	0.50012	0.5309	0.98	0.50163	0.45158	0.42394	0.37866	0.98	0.37632	0.40972	0.59556	0.58806	0.98	0.53525	0.56208	0.50056	
0.984	0.41979	0.48174	0.5038	0.53808	0.984	0.50716	0.45741	0.4294	0.3838	0.984	0.38033	0.41631	0.5977	0.5939	0.984	0.53815	0.56737	0.50302	
0.988	0.42026	0.48416	0.50817	0.54559	0.988	0.51367	0.46324	0.43554	0.38926	0.988	0.38491	0.42291	0.60054	0.6001	0.988	0.54177	0.57298	0.50596	
0.992	0.4212	0.48688	0.51231	0.55375	0.992	0.51986	0.46947	0.44202	0.39493	0.992	0.38948	0.42987	0.60409	0.60667	0.992	0.5451	0.57889	0.5089	
0.996	0.42167	0.48931	0.51668	0.56126	0.996	0.52604	0.47569	0.44816	0.40039	0.996	0.39349	0.4372	0.60694	0.61287	0.996	0.54857	0.58419	0.51185	
1	0.42309	0.49233	0.52174	0.56942	1	0.53255	0.4823	0.45464	0.40616	1	0.39864	0.44416	0.61049	0.61944	1	0.55248	0.5898	0.51528	
1.004	0.42403	0.49536	0.52657	0.57758	1.004	0.53939	0.48891	0.4618	0.41258	1.004	0.40379	0.45185	0.61405	0.62637	1.004	0.55683	0.5954	0.51872	
1.008	0.42544	0.49899	0.53163	0.58574	1.008	0.54622	0.49553	0.46896	0.41847	1.008	0.40951	0.45955	0.61831	0.63331	1.008	0.56088	0.60101	0.52215	
1.012	0.42685	0.50201	0.53692	0.5939	1.012	0.55273	0.50253	0.47613	0.42489	1.012	0.41466	0.46688	0.62257	0.63987	1.012	0.56464	0.6063	0.52559	
1.016	0.42826	0.50594	0.54221	0.60141	1.016	0.55957	0.50875	0.48329	0.43131	1.016	0.42038	0.47494	0.62684	0.64681	1.016	0.56942	0.6116	0.52951	
1.02	0.43014	0.50958	0.54773	0.60924	1.02	0.56641	0.51614	0.49045	0.43826	1.02	0.4261	0.483	0.63181	0.65374	1.02	0.57449	0.61689	0.53343	
1.024	0.4325	0.51321	0.55326	0.61643	1.024	0.57292	0.52314	0.49761	0.44468	1.024	0.4324	0.49069	0.63679	0.66031	1.024	0.5797	0.62219	0.53736	
1.028	0.43438	0.51653	0.55832	0.62296	1.028	0.57878	0.52898	0.50443	0.45057	1.028	0.43812	0.49802	0.64105	0.66651	1.028	0.58419	0.62686	0.54128	
1.032	0.43673	0.52047	0.56384	0.62948	1.032	0.58496	0.53559	0.51126	0.45806	1.032	0.44441	0.50608	0.64674	0.67344	1.032	0.58969	0.63184	0.54521	
1.036	0.43909	0.5241	0.56913	0.63536	1.036	0.59049	0.5422	0.51774	0.46501	1.036	0.45071	0.51378	0.65172	0.67964	1.036	0.5949	0.63652	0.54913	
1.04	0.44191	0.52773	0.57396	0.64091	1.04	0.59603	0.54842	0.52422	0.47197	1.04	0.457	0.52111	0.6574	0.68585	1.04	0.60012	0.64088	0.55306	
1.044	0.44426	0.53106	0.57902	0.64548	1.044	0.60091	0.55387	0.53001	0.47892	1.044	0.46272	0.52843	0.66238	0.69132	1.044	0.60518	0.64493	0.55698	
1.048	0.44708	0.53408	0.58362	0.64972	1.048	0.60514	0.55932	0.53581	0.48534	1.048	0.46844	0.53539	0.66735	0.69679	1.048	0.6104	0.64866	0.56091	
1.052	0.44991	0.53741	0.58822	0.65364	1.052	0.60905	0.56476	0.54127	0.4923	1.052	0.47474	0.54272	0.67233	0.70227	1.052	0.61561	0.6524	0.56483	
1.056	0.45273	0.54074	0.59282	0.65723	1.056	0.61296	0.56982	0.54638	0.49872	1.056	0.48046	0.54932	0.67802	0.70701	1.056	0.62082	0.65583	0.56876	
1.06	0.45508	0.54316	0.59673	0.66017	1.06	0.61589	0.57448	0.55048	0.50567	1.06	0.48561	0.55555	0.68228	0.71139	1.06	0.62603	0.65863	0.5717	
1.064	0.45791	0.54618	0.60041	0.66311	1.064	0.61849	0.57876	0.55491	0.51209	1.064	0.49076	0.56178	0.68726	0.7154	1.064	0.63095	0.66112	0.57514	
1.068	0.46073	0.5486	0.60478	0.66539	1.068	0.62109	0.58304	0.559	0.51798	1.068	0.49591	0.56727	0.69223	0.71941	1.068	0.63573	0.66393	0.57857	
1.072	0.46308	0.55102	0.60847	0.66735	1.072	0.62305	0.58732	0.56241	0.52386	1.072	0.50106	0.57277	0.6965	0.72306	1.072	0.64036	0.66642	0.58151	
1.076	0.46544	0.55344	0.61192	0.66898	1.076	0.625	0.59082	0.56617	0.52921	1.076	0.50506	0.57753	0.70005	0.72598	1.076	0.64485	0.6686	0.58397	
1.08	0.46826	0.55556	0.6156	0.67062	1.08	0.6263	0.59393	0.56924	0.53456	1.08	0.50907	0.5823	0.70431	0.7289	1.08	0.64891	0.67016	0.58593	
1.084	0.47108	0.55829	0.61928	0.67258	1.084	0.62826	0.59782	0.57333	0.53991	1.084	0.51307	0.58742	0.70858	0.73182	1.084	0.65354	0.67234	0.58936	
1.088	0.47344	0.5604	0.62273	0.67421	1.088	0.62988	0.60093	0.57606	0.54473	1.088	0.51708	0.59182	0.71213	0.73401	1.088	0.65745	0.6742	0.59182	
1.092	0.47579	0.56222	0.62572	0.67551	1.092	0.63086	0.60366	0.57913	0.54954	1.092	0.51994	0.59549	0.71498	0.7362	1.092	0.66107	0.67514	0.59378	
1.096	0.47861	0.56434	0.62894	0.67715	1.096	0.63249	0.60677	0.5822	0.55435	1.096	0.52337	0.59952	0.71853	0.73802	1.096	0.66483	0.67701	0.59623	
1.1	0.48097	0.56645	0.63262	0.6791	1.1	0.63411	0.60949	0.58527	0.55863	1.1	0.52681	0.60355	0.72208	0.73985	1.1	0.66845	0.67856	0.59819	
1.104	0.48332	0.56857	0.63584	0.68074	1.104	0.63542	0.6126	0.58799	0.56345	1.104	0.53024	0.60721	0.72493	0.74131	1.104	0.67193	0.67981	0.60016	
1.108	0.48567	0.57069	0.63906	0.6827	1.108	0.63672	0.61532	0.59106	0.56773	1.108	0.5331	0.61051	0.72777	0.74277	1.108	0.67555	0.68106	0.60212	
1.112	0.48849	0.57311	0.64251	0.68465	1.112	0.63867	0.61805	0.59413	0.57201	1.112	0.53596	0.61454	0.73061	0.74386	1.112	0.67873	0.68199	0.60359	
1.116	0.49132	0.57553	0.64619	0.68694	1.116	0.64062	0.62116	0.59754	0.57629	1.116	0.53939	0.6182	0.73275	0.74532	1.116	0.68177	0.68386	0.60604	
1.12	0.49414	0.57886	0.65033	0.68955	1.12	0.64355	0.62427	0.6013	0.5811	1.12	0.5434	0.62223	0.73559	0.74678	1.12	0.6851	0.68542	0.60801	
1.124	0.49696	0.58128	0.65378	0.69216	1.124	0.64551	0.62738	0.60402	0.58538	1.124	0.54683	0.6259	0.73772	0.74787	1.124	0.68829	0.68697	0.61046	
1.128	0.50073	0.5843	0.65769	0.6951	1.128	0.64844	0.63088	0.60812	0.5902	1.128	0.55027	0.63029	0.74056	0.74933	1.128	0.69205	0.68884	0.61291	
1.132	0.50402	0.58763	0.66184	0.69804	1.132	0.65169	0.63438	0.61221	0.59501	1.132	0.55427	0.63469	0.7427	0.75043	1.132	0.6948	0.69102	0.61537	
1.136	0.50779	0.59096	0.66598	0.70163	1.136	0.65527	0.63788	0.6163	0.59983	1.136	0.55885	0.63909	0.74554	0.75225	1.136	0.69755	0.69352	0.61831	
1.14	0.51155	0.59459	0.66989	0.70522	1.14	0.65885	0.64177	0.62074	0.60464	1.14	0.56343	0.64349	0.74767	0.75371	1.14	0.70074	0.6957	0.62076	
1.144	0.51579	0.59883	0.67449	0.70914	1.144	0.66243	0.64605	0.62551	0.60999	1.144	0.568	0.64861	0.75052	0.75517	1.144	0.70421	0.6985	0.62371	
1.148	0.52049	0.60306	0.67909	0.71371	1.148	0.66699	0.65033	0.63029	0.61534	1.148	0.57258	0.65411	0.75336	0.75736	1.148	0.7074	0.70192	0.62763	
1.152	0.5252	0.6079	0.68415	0.71828	1.152	0.67188	0.655	0.6354	0.62123	1.152	0.57773	0.65961	0.7562	0.75955	1.152	0.71116	0.70535	0.63107	
1.156	0.53038	0.61274	0.68875	0.72285	1.156	0.67643	0.65928	0.64052	0.62658	1.156	0.58345	0.6651	0.75904	0.7621	1.156	0.7142	0.70878	0.63499	
1.16	0.53602	0.61819	0.69404	0.72872	1.16	0.68229	0.66472	0.64632	0.633	1.16	0.58917	0.6717	0.76189	0.76502	1.16	0.71768	0.71314	0.63941	
1.164	0.54214	0.62363	0.69933	0.73395	1.164	0.68783	0.66978	0.65177	0.63942	1.164	0.59547	0.67793	0.76544	0.76794	1.164	0.72144	0.7175	0.64431	
1.168	0.54779	0.62969	0.70485	0.73982	1.168	0.69368	0.67561	0.65791	0.64637	1.168	0.60176	0.68452	0.76828	0.77123	1.168	0.72521	0.72217	0.64873	
1.172	0.55437	0.63543	0.71037	0.7457	1.172	0.69987	0.68145	0.66405	0.65279	1.172	0.60863	0.69112	0.77184	0.77451	1.172	0.72897	0.72684	0.65412	
1.176	0.56143	0.64209	0.7159	0.75158	1.176	0.70605	0.68728	0.67053	0.65975	1.176	0.61607	0.69808	0.77539	0.77816	1.176	0.73273	0.73214	0.65952	
1.18	0.56849	0.64875	0.72211	0.75778	1.18	0.71289	0.69389	0.67701	0.66724	1.18	0.62351	0.70577	0.77895	0.78217	1.18	0.73722	0.73743	0.66541	
1.184	0.57696	0.65601	0.72901	0.76529	1.184	0.7207	0.70167	0.68486	0.6758	1.184	0.63209	0.7142	0.78392	0.78728	1.184	0.74244	0.74366	0.67228	
1.188	0.58496	0.66266	0.73476	0.77116	1.188	0.72754	0.70828	0.69134	0.68329	1.188	0.6401	0.72153	0.78819	0.79129	1.188	0.74577	0.74896	0.67816	
1.192	0.59296	0.67023	0.74166	0.77769	1.192	0.7347	0.71529	0.69884	0.69078	1.192	0.64868	0.72959	0.79316	0.79677	1.192	0.75025	0.75487	0.68503	
1.196	0.6019	0.67749	0.7481	0.78422	1.196	0.74219	0.72268	0.70668	0.69934	1.196	0.65726	0.73765	0.79814	0.80188	1.196	0.75532	0.76079	0.69239	
1.2	0.61084	0.68505	0.75477	0.79075	1.2	0.74935	0.73046	0.71419	0.7079	1.2	0.66642	0.74571	0.8024	0.80735	1.2	0.7601	0.76702	0.69975	
1.204	0.62025	0.69261	0.76098	0.79662	1.204	0.75651	0.73785	0.72203	0.71646	1.204	0.67557	0.75377	0.80738	0.81282	1.204	0.76517	0.77263	0.70711	
1.208	0.62966	0.70018	0.76812	0.80315	1.208	0.764	0.74562	0.73022	0.72502	1.208	0.6853	0.7622	0.81306	0.81902	1.208	0.77023	0.77855	0.71447	
1.212	0.64002	0.70835	0.77548	0.81001	1.212	0.77116	0.75379	0.73909	0.73411	1.212	0.69503	0.77063	0.81875	0.82523	1.212	0.77617	0.78509	0.72232	
1.216	0.6499	0.71652	0.78261	0.81654	1.216	0.77897	0.76196	0.74761	0.74374	1.216	0.70533	0.77906	0.82444	0.83179	1.216	0.78239	0.79132	0.73066	
1.22	0.66025	0.72438	0.78997	0.82241	1.22	0.78613	0.76974	0.75614	0.75284	1.22	0.71563	0.78748	0.83012	0.838	1.22	0.78804	0.79755	0.73851	
1.224	0.67107	0.73285	0.79779	0.82927	1.224	0.79427	0.77869	0.76603	0.763	1.224	0.72593	0.79628	0.83723	0.84457	1.224	0.7947	0.80409	0.74783	
1.228	0.68237	0.74193	0.80561	0.83612	1.228	0.80273	0.78763	0.77524	0.77316	1.228	0.7368	0.80507	0.84363	0.8515	1.228	0.80136	0.81125	0.75715	
1.232	0.69366	0.75101	0.81366	0.84298	1.232	0.8112	0.79658	0.78547	0.78333	1.232	0.74824	0.81386	0.85074	0.85843	1.232	0.80846	0.81873	0.76647	
1.236	0.70496	0.76008	0.82149	0.84918	1.236	0.81934	0.80552	0.79468	0.79296	1.236	0.75911	0.82266	0.85713	0.865	1.236	0.81526	0.82558	0.7753	
1.24	0.71719	0.77007	0.83023	0.85636	1.24	0.82813	0.81486	0.80525	0.80366	1.24	0.77056	0.83145	0.86424	0.87157	1.24	0.8225	0.83305	0.78512	
1.244	0.7299	0.78066	0.83897	0.86387	1.244	0.83724	0.82458	0.81583	0.81436	1.244	0.78314	0.84061	0.87135	0.87886	1.244	0.8309	0.84084	0.79542	
1.248	0.74401	0.79185	0.84817	0.87171	1.248	0.84701	0.83508	0.82674	0.82559	1.248	0.79573	0.84977	0.87917	0.88616	1.248	0.83987	0.84956	0.8067	
1.252	0.75766	0.80304	0.8576	0.87954	1.252	0.85677	0.8452	0.83765	0.83629	1.252	0.80832	0.85857	0.88627	0.89346	1.252	0.84856	0.85797	0.817	
1.256	0.77225	0.81545	0.86726	0.88803	1.256	0.86686	0.8557	0.84959	0.84806	1.256	0.82148	0.86883	0.8948	0.90076	1.256	0.85797	0.867	0.82878	
1.26	0.78872	0.82815	0.87762	0.89684	1.26	0.87728	0.86698	0.86153	0.85983	1.26	0.83521	0.87835	0.90262	0.90878	1.26	0.86839	0.87697	0.84104	
1.264	0.80519	0.84177	0.88774	0.90566	1.264	0.88835	0.87865	0.87381	0.87214	1.264	0.84894	0.88861	0.91115	0.91608	1.264	0.87882	0.88663	0.8538	
1.268	0.82213	0.85538	0.89832	0.91447	1.268	0.89909	0.88993	0.88608	0.88444	1.268	0.86325	0.89924	0.91968	0.92411	1.268	0.88939	0.8969	0.86656	
1.272	0.84048	0.87021	0.90936	0.92426	1.272	0.91016	0.90198	0.8987	0.89728	1.272	0.87755	0.90986	0.92892	0.93213	1.272	0.90184	0.90781	0.88029	
1.276	0.85977	0.88624	0.9211	0.93438	1.276	0.9222	0.91482	0.91235	0.91066	1.276	0.893	0.92159	0.93816	0.94053	1.276	0.91443	0.91964	0.89599	
1.28	0.88001	0.90319	0.93306	0.9445	1.28	0.93424	0.92765	0.92531	0.92457	1.28	0.9096	0.93368	0.9474	0.94928	1.28	0.92718	0.93179	0.91071	
1.284	0.90118	0.92013	0.94502	0.95462	1.284	0.94629	0.94088	0.93895	0.93848	1.284	0.92562	0.94541	0.95735	0.95804	1.284	0.94006	0.94394	0.92641	
1.288	0.92471	0.93919	0.95859	0.96605	1.288	0.95964	0.95566	0.95464	0.95346	1.288	0.94335	0.95896	0.96801	0.96826	1.288	0.95454	0.95764	0.94407	
1.292	0.94871	0.95885	0.97216	0.97715	1.292	0.97266	0.97005	0.9693	0.96844	1.292	0.96166	0.97215	0.97797	0.97847	1.292	0.96873	0.97134	0.96173	
1.296	0.97365	0.97912	0.98597	0.98825	1.296	0.98633	0.98483	0.98431	0.98449	1.296	0.98055	0.98608	0.98863	0.98905	1.296	0.98378	0.98536	0.98038	
1.3	1	1	1	1	1.3	1	1	1	1	1.3	1	1	1	1	1.3	1	1	1	

Water-PBS (Spring)					Water-PBS (Summer)					Water-PBS (Autumn)					Water-PBS (Winter)			
0.004	0.01569	0.01132	0.01499	0.05438	0.004	0.04137	0.04662	0.05022	0.01631	0.004	0.03519	0.05308	0.03003	0.07121	0.004	0.01839	0.03421	0.03515
0.008	0.01375	0.00954	0.01338	0.0485	0.008	0.03831	0.04327	0.04634	0.01441	0.008	0.03277	0.0492	0.02678	0.0653	0.008	0.0164	0.03163	0.03188
0.012	0.01209	0.00802	0.01161	0.0438	0.012	0.03549	0.03992	0.04282	0.0128	0.012	0.03003	0.04557	0.02396	0.05995	0.012	0.01452	0.02906	0.02896
0.016	0.01055	0.00657	0.00999	0.04027	0.016	0.0329	0.03685	0.03964	0.0114	0.016	0.02742	0.04169	0.02141	0.05524	0.016	0.01287	0.02672	0.02648
0.02	0.00915	0.00534	0.0087	0.03645	0.02	0.03007	0.03378	0.03629	0.01005	0.02	0.02506	0.03832	0.01925	0.05093	0.02	0.01143	0.0245	0.02396
0.024	0.00773	0.00423	0.00741	0.03292	0.024	0.02772	0.03127	0.03358	0.00905	0.024	0.0228	0.03521	0.01732	0.04687	0.024	0.00994	0.0223	0.0219
0.028	0.00669	0.00342	0.00661	0.02939	0.028	0.0256	0.02903	0.03068	0.00795	0.028	0.02076	0.03263	0.01558	0.04338	0.028	0.00872	0.02024	0.01969
0.032	0.00552	0.00258	0.00564	0.02675	0.032	0.02348	0.02652	0.0279	0.00715	0.032	0.01901	0.03004	0.01393	0.03979	0.032	0.00751	0.0182	0.01787
0.036	0.00457	0.00184	0.00451	0.0241	0.036	0.02148	0.02429	0.02525	0.00635	0.036	0.01701	0.02745	0.01266	0.03638	0.036	0.0064	0.01614	0.01622
0.04	0.0036	0.00133	0.00387	0.02146	0.04	0.01933	0.02233	0.02271	0.0056	0.04	0.01503	0.02486	0.01134	0.03336	0.04	0.00536	0.01444	0.01436
0.044	0.00268	7.38E-04	0.00306	0.0194	0.044	0.01738	0.02038	0.02028	0.0049	0.044	0.0135	0.02279	0.01017	0.03038	0.044	0.00442	0.01273	0.01267
0.048	0.00194	4.67E-04	0.00226	0.01675	0.048	0.01559	0.01843	0.01809	0.004	0.048	0.01172	0.02046	0.00904	0.02754	0.048	0.00359	0.0109	0.01088

0.052	0.00117	2.21E-04	0.00177	0.01499
0.056	8.01E-04	0	0.00113	0.01323
0.06	4.29E-04	2.21E-04	9.67E-04	0.01117
0.064	1.68E-04	6.15E-04	4.84E-04	0.00911
0.068	0	0.00108	1.61E-04	0.00794
0.072	3.17E-04	0.00192	0	0.00676
0.076	8.20E-04	0.0031	1.61E-04	0.00558
0.08	0.00158	0.00453	6.45E-04	0.00382
0.084	0.00283	0.0064	0.00129	0.00323
0.088	0.00458	0.0089	0.00226	0.00206
0.092	0.00701	0.01188	0.00355	0.00118
0.096	0.01004	0.01557	0.00548	5.88E-04
0.1	0.01398	0.02	0.00806	0
0.104	0.01871	0.02536	0.01112	0
0.108	0.02449	0.03168	0.01499	2.94E-04
0.112	0.03138	0.03892	0.01966	5.88E-04
0.116	0.03939	0.04757	0.02531	0.00176
0.12	0.04871	0.05739	0.03175	0.00323
0.124	0.05933	0.06868	0.03981	0.00558
0.128	0.07107	0.08145	0.04884	0.00823
0.132	0.08393	0.09554	0.05916	0.01146
0.136	0.09809	0.11102	0.07108	0.01558
0.14	0.113	0.12799	0.08414	0.02028
0.144	0.12884	0.14641	0.09848	0.02587
0.148	0.14524	0.16585	0.11428	0.03204
0.152	0.16201	0.18626	0.13137	0.0388
0.156	0.17934	0.20767	0.14958	0.04615
0.16	0.19667	0.22956	0.16876	0.05438
0.164	0.21381	0.2517	0.18875	0.0632
0.168	0.23096	0.27384	0.20954	0.0729
0.172	0.2481	0.29598	0.23082	0.08289
0.176	0.26543	0.31762	0.25193	0.09406
0.18	0.28295	0.33853	0.27369	0.10611
0.184	0.30121	0.35895	0.29545	0.11905
0.188	0.32059	0.37863	0.31738	0.13374
0.192	0.34164	0.39806	0.33962	0.14991
0.196	0.36438	0.41626	0.3617	0.16755
0.2	0.38916	0.43447	0.38507	0.18783
0.204	0.41581	0.45169	0.40941	0.21076
0.208	0.44432	0.46841	0.43504	0.23692
0.212	0.47395	0.4844	0.46212	0.26573
0.216	0.50376	0.49966	0.49113	0.29777
0.22	0.53265	0.51392	0.52208	0.33216
0.224	0.55967	0.52696	0.55529	0.3689
0.228	0.58277	0.53778	0.59026	0.40623
0.232	0.60122	0.54713	0.62669	0.44356
0.236	0.61352	0.55353	0.66425	0.47942
0.24	0.61893	0.55673	0.70181	0.51205
0.244	0.61669	0.55673	0.73823	0.53909
0.248	0.60719	0.55328	0.77257	0.56055
0.252	0.59079	0.54639	0.80464	0.57437
0.256	0.56787	0.53631	0.83253	0.57995
0.26	0.53954	0.52229	0.85493	0.57643
0.264	0.50749	0.50581	0.87202	0.56437
0.268	0.47264	0.48662	0.88282	0.5438
0.272	0.43649	0.46472	0.88685	0.51617
0.276	0.40016	0.44185	0.88443	0.48236
0.28	0.36475	0.41823	0.87556	0.44503
0.284	0.33158	0.39413	0.86138	0.40535
0.288	0.30046	0.37002	0.84172	0.36508
0.292	0.27214	0.3469	0.81802	0.3254
0.296	0.24661	0.32426	0.79175	0.28748
0.3	0.22406	0.30336	0.76354	0.25162
0.304	0.20394	0.28392	0.7342	0.21928
0.308	0.18661	0.26596	0.70487	0.18959
0.312	0.17133	0.24998	0.67618	0.16314
0.316	0.15828	0.23522	0.6491	0.13992
0.32	0.1471	0.22242	0.62315	0.11964
0.324	0.1376	0.21111	0.59881	0.102
0.328	0.12958	0.20127	0.57689	0.08613
0.332	0.12288	0.19241	0.55674	0.07319
0.336	0.11729	0.18503	0.53836	0.06173
0.34	0.11263	0.17864	0.52176	0.05173
0.344	0.1089	0.17298	0.50677	0.0435
0.348	0.10555	0.16831	0.49339	0.03645
0.352	0.10312	0.16437	0.48146	0.03057
0.356	0.10126	0.16117	0.47034	0.02528
0.36	0.09996	0.15822	0.46051	0.02058
0.364	0.09921	0.1565	0.45229	0.01764
0.368	0.09865	0.15478	0.44439	0.0147
0.372	0.09846	0.1533	0.43714	0.01235
0.376	0.09865	0.15256	0.43053	0.01058
0.38	0.09921	0.15207	0.42473	0.00882
0.384	0.10014	0.15183	0.41957	0.00823
0.388	0.10107	0.15158	0.41473	0.00735
0.392	0.10238	0.15207	0.41054	0.00735
0.396	0.10387	0.15256	0.40667	0.00735
0.4	0.10573	0.1533	0.40297	0.00764
0.404	0.1076	0.15453	0.40006	0.00852
0.408	0.10965	0.15552	0.39732	0.00941
0.412	0.11169	0.15675	0.39507	0.01088
0.416	0.11393	0.15822	0.39281	0.01205
0.42	0.11617	0.15945	0.39104	0.01352
0.424	0.1184	0.16117	0.38959	0.01499
0.428	0.12083	0.16289	0.38846	0.01705
0.432	0.12325	0.16462	0.38749	0.01911
0.436	0.12567	0.16634	0.38652	0.02087
0.44	0.12791	0.16831	0.38604	0.02293
0.444	0.13033	0.17003	0.38556	0.02469
0.448	0.13257	0.172	0.3854	0.02675
0.452	0.1348	0.17372	0.38507	0.02851
0.456	0.13704	0.17544	0.38507	0.03028
0.46	0.13909	0.17716	0.38524	0.03204

0.052	0.01401	0.01675	0.01598	0.00355
0.056	0.01215	0.0148	0.01393	0.00295
0.06	0.01046	0.01312	0.01227	0.0025
0.064	0.00892	0.01145	0.01051	0.0019
0.068	0.00735	0.00977	0.00882	0.00145
0.072	0.0061	0.0081	0.00723	0.0011
0.076	0.0048	0.0067	0.00578	7.00E-04
0.08	0.00353	0.00503	0.00444	3.00E-04
0.084	0.00238	0.00391	0.00317	2.50E-04
0.088	0.00139	0.00279	0.00226	5.00E-05
0.092	8.71E-04	0.00195	0.00127	0
0.096	3.53E-04	8.38E-04	8.11E-04	2.50E-04
0.1	0	2.79E-04	1.41E-04	3.50E-04
0.104	1.18E-04	0	2.12E-04	9.00E-04
0.108	5.65E-04	0	0	0.00165
0.112	0.00134	0	6.70E-04	0.00225
0.116	0.00273	8.38E-04	0.00194	0.0034
0.12	0.00454	0.00195	0.00328	0.00485
0.124	0.00695	0.00335	0.00529	0.0066
0.128	0.01013	0.00558	0.00783	0.0087
0.132	0.01368	0.0081	0.01083	0.0111
0.136	0.01808	0.01117	0.01478	0.01415
0.14	0.02324	0.0148	0.01904	0.01761
0.144	0.02889	0.01926	0.02398	0.02156
0.148	0.03549	0.02429	0.02977	0.02606
0.152	0.04279	0.02987	0.03597	0.03111
0.156	0.05103	0.03629	0.04246	0.03661
0.16	0.05998	0.04327	0.04987	0.04266
0.164	0.06963	0.05081	0.05763	0.04932
0.168	0.07999	0.05918	0.06609	0.05652
0.172	0.09106	0.06784	0.07491	0.06422
0.176	0.10307	0.07705	0.08443	0.07248
0.18	0.11578	0.08654	0.09501	0.08138
0.184	0.12921	0.09659	0.1063	0.09103
0.188	0.14404	0.1072	0.11829	0.10149
0.192	0.16029	0.11809	0.13204	0.11279
0.196	0.17771	0.12954	0.14686	0.12459
0.2	0.19726	0.14182	0.16308	0.1372
0.204	0.21916	0.15438	0.18177	0.15045
0.208	0.24341	0.16806	0.20223	0.16421
0.212	0.27049	0.18258	0.22515	0.17821
0.216	0.3011	0.19821	0.24984	0.19217
0.22	0.33431	0.21496	0.27629	0.20582
0.224	0.37081	0.23339	0.30451	0.21838
0.228	0.40872	0.25209	0.33307	0.22923
0.232	0.44898	0.27219	0.36235	0.23813
0.236	0.48972	0.29257	0.39091	0.24449
0.24	0.52999	0.31295	0.41701	0.24794
0.244	0.56837	0.33305	0.44064	0.24794
0.248	0.60369	0.35204	0.46004	0.24454
0.252	0.63478	0.36907	0.47521	0.23838
0.256	0.66021	0.38358	0.48543	0.22913
0.26	0.67787	0.39447	0.48931	0.21758
0.264	0.68799	0.40229	0.4879	0.20457
0.268	0.68917	0.40536	0.48085	0.19057
0.272	0.68187	0.4048	0.46886	0.17591
0.276	0.66609	0.39922	0.45228	0.16166
0.28	0.64255	0.38973	0.43183	0.1481
0.284	0.61288	0.37633	0.40961	0.1355
0.288	0.57803	0.35985	0.38562	0.12384
0.292	0.53917	0.34059	0.36094	0.11334
0.296	0.49891	0.31993	0.33625	0.10409
0.3	0.45817	0.29844	0.31227	0.09603
0.304	0.41743	0.27638	0.28934	0.08908
0.308	0.37858	0.25433	0.26748	0.08288
0.312	0.34184	0.23339	0.24702	0.07758
0.316	0.3084	0.21357	0.22868	0.07323
0.32	0.27756	0.19458	0.21175	0.06937
0.324	0.24977	0.17755	0.19588	0.06602
0.328	0.22481	0.16192	0.18177	0.06347
0.332	0.20291	0.14796	0.16872	0.06102
0.336	0.18384	0.13568	0.15744	0.05922
0.34	0.16688	0.12451	0.1465	0.05767
0.344	0.15181	0.11474	0.13734	0.05637
0.348	0.1391	0.10637	0.12958	0.05552
0.352	0.1285	0.09911	0.12217	0.05492
0.356	0.11908	0.09296	0.11582	0.05427
0.36	0.11131	0.08766	0.11018	0.05407
0.364	0.10472	0.08347	0.1063	0.05427
0.368	0.0993	0.07956	0.10207	0.05417
0.372	0.09436	0.07621	0.09889	0.05457
0.376	0.09035	0.07342	0.09607	0.05507
0.38	0.08753	0.07147	0.09395	0.05572
0.384	0.08494	0.06979	0.09219	0.05637
0.388	0.08305	0.0684	0.09113	0.05717
0.392	0.08188	0.06728	0.09043	0.05822
0.396	0.08093	0.06644	0.09008	0.05927
0.4	0.08046	0.06616	0.08972	0.06042
0.404	0.08023	0.06616	0.09008	0.06172
0.408	0.0807	0.06616	0.09043	0.06302
0.412	0.08117	0.06644	0.09149	0.06467
0.416	0.08188	0.067	0.09219	0.06622
0.42	0.08282	0.06756	0.0936	0.06787
0.424	0.08399	0.0684	0.09466	0.06972
0.428	0.08517	0.06951	0.09642	0.07168
0.432	0.08682	0.07091	0.09783	0.07358
0.436	0.08847	0.07203	0.0996	0.07553
0.44	0.09012	0.07314	0.10136	0.07733
0.444	0.092	0.07482	0.10312	0.07943
0.448	0.09388	0.07649	0.10524	0.08138
0.452	0.09553	0.07817	0.107	0.08323
0.456	0.09742	0.07956	0.10912	0.08523
0.46	0.0993	0.08124	0.11088	0.08708

0.052	0.01016	0.01813	0.00795	0.02469
0.056	0.00853	0.01605	0.00682	0.02205
0.06	0.0072	0.01424	0.00593	0.01955
0.064	0.00576	0.01217	0.00513	0.01705
0.068	0.00433	0.0101	0.00424	0.01454
0.072	0.00306	0.00829	0.00344	0.01217
0.076	0.00213	0.00647	0.00278	0.00997
0.08	0.00127	0.00518	0.00212	0.00807
0.084	4.46E-04	0.00363	0.00151	0.0063
0.088	6.37E-05	0.00259	9.88E-04	0.00462
0.092	0	0.00155	6.59E-04	0.00324
0.096	1.91E-04	5.18E-04	2.82E-04	0.00194
0.1	6.05E-04	2.59E-04	0	9.93E-04
0.104	0.00131	0	0	3.88E-04
0.108	0.00277	2.59E-04	1.41E-04	0
0.112	0.00481	7.77E-04	5.65E-04	3.45E-04
0.116	0.00732	0.00207	0.00104	0.00104
0.12	0.0107	0.00363	0.00174	0.00216
0.124	0.01446	0.00621	0.00287	0.00406
0.128	0.01927	0.00932	0.00419	0.0066
0.132	0.02487	0.01269	0.00588	0.00971
0.136	0.0313	0.01735	0.00791	0.01381
0.14	0.03888	0.02279	0.01017	0.01864
0.144	0.04719	0.02874	0.01294	0.02413
0.148	0.05646	0.03547	0.016	0.03082
0.152	0.06687	0.04324	0.01963	0.03833
0.156	0.07808	0.05205	0.02353	0.047
0.16	0.0905	0.06189	0.02791	0.05684
0.164	0.10324	0.0725	0.03247	0.0675
0.168	0.11725	0.08441	0.0376	0.07941
0.172	0.13222	0.09762	0.04278	0.09279
0.176	0.14782	0.11186	0.04843	0.10729
0.18	0.16438	0.12765	0.05436	0.12352
0.184	0.1819	0.14526	0.06052	0.14113
0.188	0.20069	0.16468	0.06707	0.16142
0.192	0.22043	0.18643	0.07384	0.18343
0.196	0.24145	0.20974	0.081	0.2076
0.2	0.26342	0.23563	0.08871	0.23479
0.204	0.28699	0.26308	0.09667	0.265
0.208	0.31119	0.29208	0.10519	0.29823
0.212	0.33571	0.32134	0.11422	0.33448
0.216	0.36087	0.3506	0.12387	0.3729
0.22	0.38475	0.3783	0.13413	0.41433
0.224	0.40768	0.40368	0.1451	0.45749
0.228	0.42774	0.42465	0.15663	0.50022
0.232	0.44526	0.44122	0.16863	0.54381
0.236	0.45831	0.4521	0.18115	0.58481
0.24	0.46691	0.45702	0.19376	0.62236
0.244	0.46946	0.4552	0.20642	0.65473
0.248	0.46691	0.44692	0.21842	0.68062
0.252	0.45927	0.4332	0.23	0.69832
0.256	0.44653	0.41455	0.24054	0.70695
0.26	0.4287	0.39125	0.24939	0.70609
0.264	0.40832	0.36561	0.25668	0.69659
0.268	0.38475	0.33817	0.26191	0.6776
0.272	0.35959	0.30968	0.26511	0.65084
0.276	0.33348	0.28094	0.26586	0.61804
0.28	0.30737	0.25298	0.26468	0.58049
0.284	0.28189	0.22657	0.26153	0.54079
0.288	0.25769	0.20171	0.25617	0.50065
0.292	0.23476	0.17815	0.24944	0.46094
0.296	0.21343	0.15691	0.24148	0.42296
0.3	0.19432	0.13801	0.23254	0.38757
0.304	0.17617	0.12066	0.22308	0.3552
0.308	0.16056	0.10539	0.21291	0.32585
0.312	0.14623	0.0914	0.20308	0.29909
0.316	0.13349	0.08001	0.19343	0.27579
0.32	0.12267	0.06965	0.18416	0.25507
0.324	0.1128	0.06033	0.17531	0.23651
0.328	0.1042	0.05256	0.16707	0.22054
0.332	0.09687	0.04635	0.15973	0.2063
0.336	0.0905	0.04039	0.15272	0.19379
0.34	0.08509	0.03573	0.14637	0.183
0.344	0.08063	0.03185	0.14072	0.1735
0.348	0.07681	0.02874	0.13578	0.1653
0.352	0.07363	0.02615	0.1314	0.15796
0.356	0.07073	0.02382	0.12731	0.15149
0.36	0.06863	0.02201	0.12396	0.14631
0.364	0.06703	0.02071	0.12109	0.14199
0.368	0.06576	0.01994	0.11846	0.13854
0.372	0.06477	0.0189	0.1162	0.13509
0.376	0.06401	0.01838	0.11427	0.13207
0.38	0.06363	0.01813	0.11272	0.13034
0.384	0.06356	0.02046	0.1114	0.12905
0.388	0.06347	0.01994	0.11027	0.12818
0.392	0.06388	0.01994	0.10923	0.12732
0.396	0.06423	0.0202	0.10843	0.12689
0.4	0.065	0.02046	0.10763	0.12732
0.404	0.06573	0.02097	0.10716	0.12732
0.408	0.06665	0.02149	0.10679	0.12818
0.412	0.06786	0.02201	0.1065	0.12905
0.416	0.06894	0.02253	0.10632	0.13034
0.42	0.07015	0.02305	0.10632	0.13164
0.424	0.07172	0.02356	0.10646	0.13336
0.428	0.07331	0.02408	0.10674	0.13552
0.432	0.0749	0.02486	0.10693	0.13725
0.436	0.07649	0.02512	0.1074	0.1394
0.44	0.07777	0.02563	0.10773	0.14156
0.444	0.07968	0.02589	0.10843	0.14415
0.448	0.08159	0.02615	0.10895	0.14631
0.452	0.08286	0.02615	0.10975	0.1489
0.456	0.08477	0.02615	0.11065	0.15149
0.46	0.08668	0.02615	0.11145	0.15408

0.052	0.00276	0.00931	0.0094
0.056	0.00199	0.00758	0.00792
0.06	0.00155	0.00629	0.00647
0.064	0.0011	0.00501	0.00523
0.068	4.97E-04	0.0037	0.00403
0.072	3.31E-04	0.00257	0.00306
0.076	1.10E-04	0.00164	0.002
0.08	0	9.36E-04	0.00117
0.084	0	4.68E-04	5.51E-04
0.088	5.52E-05	9.36E-05	2.07E-04
0.092	1.66E-04	0	0
0.096	1.66E-04	3.74E-04	0
0.1	1.66E-04	8.66E-04	4.48E-04
0.104	3.87E-04	0.00201	0.00134
0.108	6.07E-04	0.00349	0.00244
0.112	8.28E-04	0.00538	0.0042
0.116	9.94E-04	0.00795	0.00644
0.12	0.00127	0.011	0.00912
0.124	0.00155	0.01455	0.01233
0.128	0.00215	0.01883	0.01629
0.132	0.00271	0.02365	0.02066
0.136	0.00348	0.02906	0.02575
0.14	0.00442	0.03491	0.0315
0.144	0.00541	0.04146	0.03784
0.148	0.0069	0.04824	0.04459
0.152	0.00817	0.0555	0.05192
0.156	0.00999	0.06298	0.0597
0.16	0.01193	0.07094	0.06796
0.164	0.01408	0.07866	0.07619
0.168	0.01651	0.08685	0.08497
0.172	0.01921	0.09504	0.09389
0.176	0.02214	0.10369	0.10301
0.18	0.02562	0.11235	0.11255
0.184	0.02937	0.12147	0.12226
0.188	0.03374	0.13107	0.13259
0.192	0.03876	0.14159	0.14329
0.196	0.04412	0.15282	0.15493
0.2	0.05036	0.16476	0.16788
0.204	0.05753	0.17739	0.18196
0.208	0.06526	0.19073	0.1978
0.212	0.07399	0.20406	0.21467
0.216	0.08376	0.21763	0.23326
0.22	0.09453	0.23073	0.25323
0.224	0.10645	0.2429	0.27389
0.228	0.11882	0.25366	0.29558
0.232	0.1323	0.26279	0.31761
0.236	0.14593	0.26957	0.3393
0.24	0.15963	0.27378	0.35927
0.244	0.17315	0.27542	0.37786
0.248	0.18652	0.27378	0.39404
0.252	0.19905	0.27004	0.40713
0.256	0.21059	0.26349	0.41642
0.26	0.22058	0.2546	0.42193
0.264	0.22903	0.24407	0.42365
0.268	0.23555	0.23214	0.42124
0.272	0.24002	0.21904	0.41505
0.276	0.24261	0.2057	0.40575
0.28	0.24311	0.1919	0.39301
0.284	0.24184	0.17856	0.37821
0.288	0.23908	0.16546	0.36168
0.292	0.23488	0.15306	0.34378
0.296	0.22975	0.14159	0.32587
0.3	0.22384	0.13083	0.30763
0.304	0.21733	0.12101	0.29007
0.308	0.21054	0.11211	0.27285
0.312	0.20363	0.10416	0.25701
0.316	0.19717	0.09714	0.24187
0.32	0.19055	0.09106	0.22844
0.324	0.18442	0.08544	0.2157
0.328	0.17862	0.08076	0.20468
0.332	0.17315	0.07702	0.1947
0.336	0.16819	0.07351	0.1854
0.34	0.1636	0.07094	0.17748
0.344	0.15941	0.06883	0.17025
0.348	0.15571	0.06696	0.16406
0.352	0.15239	0.06579	0.15875
0.356	0.14952	0.06485	0.15407
0.36	0.14715	0.06415	0.15001
0.364	0.14549	0.06415	0.1467
0.368	0.14389	0.06415	0.14378
0.372	0.14257	0.06439	0.14123
0.376	0.14141	0.06485	0.13903
0.38	0.14074	0.06556	0.13744
0.384	0.14014	0.06626	0.13596
0.388	0.14003	0.06719	0.13476
0.392	0.14008	0.06836	0.13403
0.396	0.14047	0.06953	0.13359
0.4	0.14113	0.07094	0.13331
0.404	0.1419	0.07234	0.13317
0.408	0.14306	0.07398	0.13321
0.412	0.14461	0.07562	0.13355
0.416	0.1461	0.07725	0.13386
0.42	0.14781	0.07913	0.13424
0.424	0.14996	0.08076	0.13486
0.428	0.15206	0.08264	0.13582
0.432	0.15466	0.08474	0.13675
0.436	0.15709	0.08638	0.13775
0.44	0.16001	0.08825	0.13882
0.444	0.16283	0.09012	0.13999
0.448	0.16576	0.09199	0.14126
0.452	0.16857	0.09387	0.1423
0.456	0.17161	0.09574	0.14371
0.46	0.17459	0.09737	0.14491

0.464	0.14132	0.17888	0.38524	0.0338	0.464	0.10071	0.08291	0.11265	0.08903	0.464	0.08827	0.02589	0.11229	0.15667	0.464	0.17752	0.09925	0.14629
0.468	0.14337	0.18061	0.38556	0.03557	0.468	0.1026	0.08459	0.11476	0.09098	0.468	0.08987	0.02563	0.11338	0.15926	0.468	0.18044	0.10112	0.1477
0.472	0.14542	0.18233	0.38588	0.03704	0.472	0.10425	0.08626	0.11653	0.09283	0.472	0.09146	0.02538	0.11418	0.16185	0.472	0.18315	0.10252	0.1488
0.476	0.14747	0.1838	0.3862	0.03851	0.476	0.10566	0.08794	0.11864	0.09463	0.476	0.09337	0.02512	0.11526	0.16444	0.476	0.1858	0.10439	0.15025
0.48	0.14952	0.18553	0.38669	0.03998	0.48	0.10731	0.08961	0.12041	0.09628	0.48	0.09528	0.02486	0.11625	0.16659	0.48	0.18823	0.1058	0.15145
0.484	0.15157	0.18676	0.38685	0.04145	0.484	0.10872	0.09101	0.12252	0.09788	0.484	0.09655	0.02434	0.11723	0.16918	0.484	0.19049	0.10744	0.15266
0.488	0.15362	0.18848	0.38733	0.04292	0.488	0.11013	0.09296	0.12429	0.09948	0.488	0.09847	0.02382	0.11836	0.17134	0.488	0.19242	0.10931	0.15404
0.492	0.15586	0.18995	0.38781	0.04439	0.492	0.11155	0.09464	0.1264	0.10119	0.492	0.10069	0.02382	0.11949	0.17436	0.492	0.19441	0.11094	0.15545
0.496	0.15809	0.19143	0.3883	0.04556	0.496	0.11296	0.09631	0.12817	0.10279	0.496	0.10229	0.0233	0.12058	0.17652	0.496	0.19596	0.11258	0.15693
0.5	0.16033	0.19315	0.38862	0.04674	0.5	0.11437	0.09799	0.13028	0.10429	0.5	0.1042	0.0233	0.12175	0.17911	0.5	0.19712	0.11422	0.15834
0.504	0.16257	0.19438	0.38943	0.04791	0.504	0.11578	0.09939	0.1324	0.10589	0.504	0.10611	0.02305	0.12284	0.18127	0.504	0.19839	0.11609	0.15992
0.508	0.16518	0.1961	0.39007	0.04909	0.508	0.11743	0.10134	0.13451	0.10764	0.508	0.10834	0.0233	0.12401	0.18429	0.508	0.19922	0.11773	0.16158
0.512	0.16778	0.19783	0.39088	0.05056	0.512	0.11908	0.10329	0.13663	0.10939	0.512	0.11057	0.02356	0.12524	0.18688	0.512	0.19999	0.11984	0.16347
0.516	0.17021	0.1993	0.39152	0.05203	0.516	0.12026	0.10497	0.13839	0.11104	0.516	0.11248	0.02382	0.12651	0.18947	0.516	0.20038	0.12147	0.16509
0.52	0.17319	0.20127	0.39249	0.0535	0.52	0.12191	0.1072	0.14121	0.11304	0.52	0.11502	0.02434	0.12773	0.19206	0.52	0.20082	0.12358	0.16688
0.524	0.17617	0.20299	0.39346	0.05526	0.524	0.12379	0.10916	0.14368	0.11474	0.524	0.11725	0.02538	0.12919	0.19465	0.524	0.20126	0.12592	0.16891
0.528	0.17952	0.20496	0.39458	0.05703	0.528	0.12591	0.11139	0.14615	0.11689	0.528	0.11948	0.02641	0.13055	0.1981	0.528	0.20142	0.12826	0.17094
0.532	0.18288	0.20693	0.39571	0.0585	0.532	0.12779	0.11362	0.14862	0.11899	0.532	0.12203	0.02771	0.13211	0.20069	0.532	0.2017	0.13083	0.17301
0.536	0.18642	0.2089	0.397	0.06055	0.536	0.12991	0.11614	0.15109	0.12104	0.536	0.12458	0.02926	0.13342	0.20371	0.536	0.20192	0.13317	0.17542
0.54	0.19033	0.21111	0.39877	0.0629	0.54	0.13203	0.11865	0.15426	0.12324	0.54	0.12744	0.03133	0.13512	0.20716	0.54	0.2022	0.13621	0.17817
0.544	0.19425	0.21332	0.40039	0.06496	0.544	0.13462	0.12144	0.15709	0.12579	0.544	0.13031	0.03366	0.13677	0.21062	0.544	0.20242	0.13925	0.18058
0.548	0.19835	0.21578	0.402	0.06761	0.548	0.13721	0.12423	0.15991	0.12829	0.548	0.13286	0.03599	0.13832	0.21407	0.548	0.20258	0.1423	0.18334
0.552	0.20282	0.21849	0.40409	0.07025	0.552	0.1398	0.1273	0.16308	0.1308	0.552	0.13604	0.03936	0.14006	0.21795	0.552	0.20325	0.14581	0.18643
0.556	0.20766	0.22095	0.40619	0.07319	0.556	0.14286	0.13037	0.16626	0.1335	0.556	0.13923	0.04247	0.1419	0.22184	0.556	0.20358	0.14931	0.18919
0.56	0.21251	0.2239	0.40845	0.07613	0.56	0.14616	0.134	0.16978	0.13645	0.56	0.14273	0.04635	0.14392	0.22572	0.56	0.20424	0.15282	0.19263
0.564	0.21735	0.22661	0.4107	0.07966	0.564	0.14922	0.13735	0.17331	0.13935	0.564	0.14591	0.05023	0.1459	0.23004	0.564	0.20485	0.1568	0.19573
0.568	0.22238	0.22956	0.41328	0.08319	0.568	0.15275	0.14098	0.17684	0.1424	0.568	0.14942	0.05463	0.14806	0.23479	0.568	0.20584	0.16078	0.19952
0.572	0.22779	0.23276	0.41618	0.08701	0.572	0.15652	0.14517	0.18107	0.14575	0.572	0.15324	0.05955	0.15041	0.23997	0.572	0.20717	0.16522	0.20331
0.576	0.23338	0.23595	0.41908	0.09112	0.576	0.16076	0.14908	0.18495	0.1491	0.576	0.15674	0.06499	0.15277	0.24471	0.576	0.20849	0.16944	0.20675
0.58	0.2386	0.23915	0.42182	0.09524	0.58	0.16476	0.15355	0.18883	0.1525	0.58	0.16024	0.07043	0.15526	0.24989	0.58	0.20982	0.17365	0.21088
0.584	0.24419	0.2426	0.42489	0.09965	0.584	0.169	0.15801	0.19306	0.1562	0.584	0.16438	0.07639	0.1578	0.2555	0.584	0.21147	0.17833	0.21467
0.588	0.24978	0.24604	0.42795	0.10464	0.588	0.17371	0.16276	0.19764	0.16001	0.588	0.16852	0.08234	0.16058	0.26111	0.588	0.2133	0.18301	0.2188
0.592	0.25537	0.24973	0.43101	0.10905	0.592	0.17819	0.16778	0.20188	0.16381	0.592	0.17235	0.08881	0.16354	0.26716	0.592	0.21534	0.18768	0.22259
0.596	0.26077	0.25317	0.43424	0.11376	0.596	0.18289	0.17281	0.20611	0.16791	0.596	0.17617	0.09503	0.16651	0.27363	0.596	0.21744	0.19236	0.22672
0.6	0.26599	0.25686	0.4373	0.11846	0.6	0.1876	0.17811	0.21069	0.17191	0.6	0.17999	0.10357	0.16962	0.2801	0.6	0.21987	0.19728	0.2305
0.604	0.27139	0.26031	0.44052	0.12316	0.604	0.19231	0.18342	0.21598	0.17601	0.604	0.18413	0.11031	0.17286	0.28658	0.604	0.22235	0.20219	0.23464
0.608	0.27642	0.26375	0.44375	0.12787	0.608	0.19726	0.18872	0.22057	0.18021	0.608	0.18795	0.11652	0.1762	0.29391	0.608	0.22506	0.20687	0.23842
0.612	0.28108	0.26719	0.44649	0.13228	0.612	0.20197	0.1943	0.22515	0.18411	0.612	0.19177	0.12222	0.17955	0.30082	0.612	0.22787	0.21131	0.24221
0.616	0.28537	0.27064	0.44955	0.13668	0.616	0.20668	0.19989	0.22974	0.18827	0.616	0.19527	0.12765	0.18293	0.30816	0.616	0.2308	0.21599	0.246
0.62	0.28965	0.27384	0.45229	0.1408	0.62	0.21139	0.20547	0.23432	0.19247	0.62	0.19846	0.13283	0.18651	0.31549	0.62	0.23406	0.22044	0.24978
0.624	0.29338	0.27703	0.45487	0.14462	0.624	0.2161	0.21078	0.23891	0.19642	0.624	0.20196	0.13698	0.19009	0.32283	0.624	0.23737	0.22488	0.25288
0.628	0.29674	0.27999	0.45712	0.14785	0.628	0.22034	0.21636	0.24314	0.20037	0.628	0.20483	0.14112	0.19362	0.33017	0.628	0.2409	0.22886	0.25598
0.632	0.29953	0.28269	0.45922	0.15138	0.632	0.22457	0.22138	0.24737	0.20412	0.632	0.20769	0.14448	0.19729	0.33751	0.632	0.24427	0.2326	0.25874
0.636	0.30214	0.2854	0.46115	0.15403	0.636	0.22881	0.22669	0.25125	0.20807	0.636	0.21024	0.14733	0.20105	0.34441	0.636	0.24803	0.23635	0.26149
0.64	0.30456	0.28761	0.46277	0.15667	0.64	0.23282	0.23116	0.25478	0.21132	0.64	0.21279	0.1494	0.20468	0.35175	0.64	0.25162	0.23986	0.2639
0.644	0.30624	0.28958	0.46389	0.15873	0.644	0.23635	0.23562	0.25831	0.21427	0.644	0.2147	0.15096	0.20807	0.35822	0.644	0.2552	0.24313	0.26597
0.648	0.30754	0.29155	0.46518	0.16049	0.648	0.23964	0.23981	0.26148	0.21693	0.648	0.21661	0.15225	0.21164	0.3647	0.648	0.25896	0.24594	0.26769
0.652	0.30847	0.29278	0.46583	0.16226	0.652	0.24271	0.24344	0.2643	0.21908	0.652	0.2182	0.15277	0.21498	0.37074	0.652	0.2626	0.24851	0.26941
0.656	0.30903	0.29425	0.46615	0.16343	0.656	0.24577	0.24679	0.26677	0.22103	0.656	0.21948	0.15303	0.21842	0.37635	0.656	0.26619	0.25085	0.27044
0.66	0.30941	0.29524	0.46631	0.16432	0.66	0.24812	0.24958	0.26889	0.22253	0.66	0.22075	0.15303	0.22148	0.38153	0.66	0.26989	0.25296	0.27182
0.664	0.30941	0.29573	0.46599	0.1652	0.664	0.25048	0.25181	0.27065	0.22373	0.664	0.22139	0.15277	0.22449	0.38628	0.664	0.27331	0.2546	0.27251
0.668	0.30922	0.29622	0.46583	0.16549	0.668	0.25236	0.25377	0.27241	0.22458	0.668	0.22234	0.15225	0.22732	0.39059	0.668	0.27685	0.25624	0.27285
0.672	0.30885	0.29671	0.46534	0.16608	0.672	0.25424	0.25572	0.27382	0.22543	0.672	0.22298	0.15173	0.23023	0.39491	0.672	0.28038	0.25764	0.27354
0.676	0.3081	0.29647	0.46406	0.16608	0.676	0.25542	0.25684	0.27453	0.22563	0.676	0.22298	0.15096	0.23263	0.39793	0.676	0.28342	0.25857	0.27354
0.68	0.30736	0.29622	0.46309	0.16608	0.68	0.25636	0.2574	0.27523	0.22563	0.68	0.22298	0.15018	0.23503	0.40052	0.68	0.28651	0.25928	0.2732
0.684	0.30624	0.29573	0.46164	0.16608	0.684	0.25707	0.25768	0.27629	0.22533	0.684	0.22298	0.14966	0.23734	0.40268	0.684	0.28955	0.25998	0.2732
0.688	0.30512	0.29499	0.46003	0.16608	0.688	0.25754	0.25768	0.27629	0.22488	0.688	0.22266	0.14889	0.23936	0.40397	0.688	0.29231	0.26045	0.27285
0.692	0.30382	0.29425	0.45809	0.16578	0.692	0.25778	0.25712	0.27629	0.22423	0.692	0.22202	0.14837	0.24129	0.40483	0.692	0.29507	0.26068	0.27216
0.696	0.30251	0.29302	0.45616	0.1652	0.696	0.25778	0.25628	0.27629	0.22348	0.696	0.22171	0.14785	0.24299	0.4057	0.696	0.29766	0.26091	0.27148
0.7	0.30139	0.29204	0.45406	0.1652	0.7	0.25778	0.25544	0.27629	0.22263	0.7	0.22107	0.14785	0.24459	0.4057	0.7	0.30009	0.26138	0.27079
0.704	0.30009	0.29106	0.45197	0.1649	0.704	0.25754	0.25433	0.27594	0.22143	0.704	0.22075	0.14759	0.24605	0.4057	0.704	0.30241	0.26138	0.2701
0.708	0.29841	0.28958	0.44955	0.16461	0.708	0.25707	0.25321	0.27559	0.22033	0.708	0.22011	0.14733	0.24722	0.40483	0.708	0.30418	0.26115	0.26907
0.712	0.29729	0.2881	0.44745	0.16432	0.712	0.2566	0.25181	0.27523	0.21958	0.712	0.21916	0.14759	0.24845	0.40397	0.712	0.30611	0.26138	0.26838
0.716	0.29599	0.28663	0.44504	0.16432	0.716	0.25613	0.25042	0.27453	0.21873	0.716	0.21852	0.14811	0.24953	0.40268	0.716	0.30788	0.26138	0.26769
0.72	0.29487	0.2854	0.44246	0.16461	0.72	0.25566	0.2493	0.27382	0.21768	0.72	0.2182	0.14837	0.25052	0.40181	0.72	0.30943	0.26138	0.26666
0.724	0.29338	0.28368	0.43988	0.16432	0.724	0.25472	0.24791	0.27312	0.21673	0.724	0.21725	0.14915	0.25136	0.40009	0.724	0.31086	0.26138	0.26562
0.728	0.29226	0.2822	0.43714	0.16461	0.728	0.25424	0.24651	0.27241	0.21588	0.728	0.21693	0.14966	0.25202	0.39836	0.728	0.31208	0.26138	0.26459
0.732	0.29133	0.28072	0.43456	0.1649	0.732	0.25377	0.24539	0.27206	0.21513	0.732	0.21629	0.1507	0.25268	0.3962	0.732	0.31324	0.26162	0.2639
0.736	0.2904	0.27925	0.43198	0.1652	0.736	0.2533	0.24428	0.27171	0.21478	0.736	0.21597	0.15173	0.25334	0.39491	0.736	0.31428	0.26185	0.26321
0.74	0.28947	0.27753	0.42924	0.16578	0.74	0.25283	0.24316	0.271	0.21412	0.74	0.21566	0.15277	0.25372	0.39275	0.74	0.31517	0.26185	0.26218
0.744	0.28854	0.27605	0.42682	0.16637	0.744	0.2526	0.24204	0.27065	0.21392	0.744	0.21534	0.15407	0.25433	0.39102	0.744	0.316	0.26208	0.26115
0.748	0.28798	0.27482	0.4244	0.16725	0.748	0.25236	0.24149	0.27065	0.21377	0.748	0.21534	0.15562	0.25494	0.38973	0.748	0.31666	0.26255	0.2608
0.752	0.28742	0.27334	0.42182	0.16784	0.752	0.25236	0.24093	0.2703	0.21367	0.752	0.21534	0.15743	0.25522	0.388	0.752	0.31727	0.26302	0.25977
0.756	0.28686	0.27187	0.41941	0.16872	0.756	0.25236	0.24065	0.2703	0.21357	0.756	0.21534	0.15898	0.25574	0.38628	0.756	0.31776	0.26349	0.25943
0.76	0.2863	0.27039	0.41683	0.16961	0.76	0.2526	0.24037	0.2703	0.21402	0.76	0.21534	0.16054	0.25598	0.38498	0.76	0.31826	0.26396	0.25839
0.764	0.28611	0.26916	0.41457	0.17078	0.764	0.25307	0.24037	0.27065	0.21438	0.764	0.21566	0.16235	0.25664	0.38412	0.764	0.31898	0.26489	0.25805
0.768	0.28611	0.26793	0.41248	0.17196	0.768	0.25354	0.24037	0.271	0.21498	0.768	0.21597	0.16442	0.25715	0.38325	0.768	0.31981	0.26559	0.2577
0.772	0.28555	0.2667	0.41022	0.17313	0.772	0.25401	0.24065	0.27136	0.21548	0.772	0.21629	0.16624	0.25744	0.38196	0.772	0.3203	0.2663	0.25736
0.776	0.2863	0.26621	0.40845	0.17519	0.776	0.25542	0.24176	0.27241	0.21668	0.776	0.21725	0.16908	0.25828	0.38196	0.776	0.32141	0.2677	0.25736
0.78	0.28649	0.26523	0.40651	0.17666	0.78	0.25613	0.2426	0.27347	0.21763	0.78	0.21788	0.17115	0.2588	0.38153	0.78	0.32235	0.26864	0.25701
0.784	0.28649	0.26424	0.40442	0.17813	0.784	0.25754	0.24316	0.27418	0.21853	0.784	0.21852	0.17349	0.25946	0.3811	0.784	0.32306	0.2698	0.25701
0.788	0.28667	0.26326	0.40264	0.1796	0.788	0.25825	0.244	0.27523	0.21948	0.788	0.21948	0.17582	0.26012	0.38066	0.788	0.32384	0.27074	0.25667
0.792	0.28705	0.26252	0.40087	0.18136	0.792	0.25966	0.24511	0.27629	0.22043	0.792	0.22011	0.17789	0.26097	0.38066	0.792	0.32472	0.27191	0.25667
0.796	0.2876	0.26178	0.3991	0.18342	0.796	0.26131	0.24623	0.2777	0.22163	0.796	0.22139	0.18022	0.26177	0.3811	0.796	0.32577	0.27331	0.25667
0.8	0.28816	0.26129	0.39765	0.18548	0.8	0.26272	0.24791	0.27911	0.22303	0.8	0.22234	0.18281	0.26275	0.38153	0.8	0.32682	0.27472	0.25701
0.804	0.28872	0.2608	0.39587	0.18724	0.804	0.26413	0.24902	0.28052	0.22433	0.804	0.2233	0.18488	0.2636	0.38153	0.804	0.32787	0.27589	0.25701
0.808	0.28928	0.26031	0.39458	0.1893	0.808	0.26602	0.2507	0.28229	0.22543	0.808	0.22457	0.18747	0.26464	0.38239	0.808	0.32919	0.27729	0.25736
0.812	0.29021	0.26006	0.39346	0.19165	0.812	0.2679	0.25265	0.28405	0.22693	0.812	0.22616	0.1898	0.266	0.38325	0.812	0.33052	0.27893	0.2577
0.816	0.29114	0.25982	0.39217	0.19371	0.816	0.26979	0.25433	0.28617	0.22848	0.816	0.22744	0.19213	0.26704	0.38369	0.816	0.33195	0.2801	0.25839
0.82	0.29189	0.25982	0.3912	0.19606	0.82	0.27191	0.25628	0.28758	0.22973	0.82	0.22871	0.1942	0.26831	0.38455	0.82	0.33344	0.28174	0.25874
0.824	0.29245	0.25932	0.38975	0.19812	0.824	0.27355	0.25796	0.28899	0.23078	0.824	0.22999	0.19601	0.2693	0.38498	0.824	0.33477	0.28291	0.25908
0.828	0.29338	0.25932	0.38894	0.20018	0.828	0.27567	0.25991	0.29111	0.23233	0.828	0.23158	0.19834	0.27066	0.38628	0.828	0.33665	0.28454	0.25977
0.832	0.29469	0.25957	0.3883	0.20282	0.832	0.27803	0.26242	0.29357	0.23363	0.832	0.23317	0.20041	0.27226	0.38714	0.832	0.33847	0.28618	0.26011
0.836	0.29562	0.25957	0.38749	0.20517	0.836	0.27991	0.2641	0.29534	0.23483	0.836	0.23444	0.20223	0.27353	0.388	0.836	0.34012	0.28759	0.2608
0.84	0.29674	0.25982	0.38685	0.20723	0.84	0.28203	0.26661	0.29745	0.23623	0.84	0.23604	0.20404	0.27546	0.38973	0.84	0.34206	0.28922	0.26184
0.844	0.29767	0.26031	0.38636	0.20958	0.844	0.28439	0.26884	0.29992	0.23773	0.844	0.23763	0.20559	0.27734	0.39059	0.844	0.34382	0.29063	0.26287
0.848	0.29879	0.26055	0.38604	0.21223	0.848	0.2865	0.27108	0.30169	0.23913	0.848	0.23922	0.20741	0.27909	0.39189	0.848	0.34587	0.29227	0.26321
0.852	0.29972	0.26104	0.38556	0.21429	0.852	0.28862	0.27303	0.30415	0.24043	0.852	0.24081	0.20896	0.28083	0.39275	0.852	0.34769	0.29367	0.26425
0.856	0.30065	0.26129	0.3854	0.21634	0.856	0.29074	0.27527	0.30627	0.24183	0.856	0.2424	0.21025	0.28252	0.39404	0.856	0.34968	0.29531	0.26493
0.86	0.30177	0.26178	0.3854	0.21869	0.86	0.29286	0.27778	0.30803	0.24299	0.86	0.244	0.21181	0.28445	0.39534	0.86	0.35172	0.29671	0.26562
0.864	0.30288	0.26227	0.38524	0.22075	0.864	0.29475	0.27973	0.31015	0.24434	0.864	0.24559	0.2131	0.28629	0.39663	0.864	0.35376	0.29811	0.26631
0.868	0.30382	0.26301	0.38491	0.22252	0.868	0.29663	0.28169	0.31191	0.24564	0.868	0.24686	0.21414	0.28807	0.3975	0.868	0.3557	0.29952	0.267
0.872	0.30493	0.2635	0.38507	0.22428	0.872	0.29875	0.28364	0.31403	0.24699	0.872	0.24846	0.21543	0.29	0.39922	0.872	0.35757	0.30092	0.26803

0.876	0.30605	0.26449	0.38524	0.22634
0.88	0.30736	0.26498	0.3854	0.2284
0.884	0.30829	0.26572	0.38556	0.22957
0.888	0.30941	0.26646	0.38556	0.23133
0.892	0.31071	0.26719	0.38572	0.2328
0.896	0.31202	0.26818	0.38604	0.23427
0.9	0.31313	0.26916	0.38636	0.23516
0.904	0.31462	0.2699	0.38669	0.23692
0.908	0.31612	0.27088	0.38717	0.23839
0.912	0.31742	0.27211	0.38749	0.23956
0.916	0.31872	0.2731	0.38798	0.24074
0.92	0.32021	0.27433	0.38846	0.24192
0.924	0.32208	0.2758	0.3891	0.24339
0.928	0.32394	0.27703	0.38959	0.24486
0.932	0.32562	0.27826	0.39007	0.24603
0.936	0.32748	0.27974	0.39104	0.2478
0.94	0.32953	0.28146	0.39201	0.24927
0.944	0.3314	0.28318	0.39281	0.25132
0.948	0.33363	0.28491	0.39362	0.25279
0.952	0.33605	0.28687	0.39475	0.25485
0.956	0.33866	0.28909	0.39587	0.25691
0.96	0.34127	0.29106	0.39732	0.25897
0.964	0.34369	0.29327	0.39845	0.26132
0.968	0.34649	0.29548	0.40006	0.26396
0.972	0.34966	0.29819	0.40152	0.26661
0.976	0.35264	0.30065	0.40329	0.26925
0.98	0.35581	0.3036	0.40506	0.27219
0.984	0.35879	0.30631	0.40683	0.27513
0.988	0.36233	0.30951	0.40877	0.27837
0.992	0.3655	0.31246	0.41119	0.28189
0.996	0.36866	0.31565	0.41328	0.28513
1	0.3722	0.31885	0.4157	0.28865
1.004	0.37556	0.3223	0.41812	0.29248
1.008	0.37891	0.32549	0.42086	0.296
1.012	0.38208	0.32894	0.42344	0.29982
1.016	0.38525	0.33238	0.42618	0.30335
1.02	0.3886	0.33607	0.42908	0.30747
1.024	0.39196	0.33952	0.43214	0.31158
1.028	0.39513	0.34296	0.43488	0.3154
1.032	0.39867	0.3469	0.43827	0.31952
1.036	0.40221	0.35059	0.44165	0.32363
1.04	0.40556	0.35403	0.44504	0.32775
1.044	0.40929	0.35797	0.44842	0.33186
1.048	0.41301	0.36166	0.45181	0.33598
1.052	0.41674	0.36584	0.45567	0.34039
1.056	0.42084	0.37002	0.45954	0.34509
1.06	0.42475	0.37395	0.46325	0.3495
1.064	0.42885	0.37863	0.4676	0.3542
1.068	0.43351	0.3833	0.47179	0.3592
1.072	0.4378	0.38798	0.47614	0.3639
1.076	0.44246	0.39265	0.48066	0.3689
1.08	0.4473	0.39782	0.48549	0.3739
1.084	0.45271	0.40347	0.49065	0.37948
1.088	0.45792	0.40913	0.49581	0.38477
1.092	0.46314	0.41479	0.50081	0.39006
1.096	0.46892	0.42094	0.50661	0.39594
1.1	0.47488	0.42709	0.51225	0.40182
1.104	0.48122	0.43348	0.51821	0.4077
1.108	0.48737	0.44013	0.52434	0.41358
1.112	0.49389	0.44751	0.53095	0.41975
1.116	0.5006	0.45464	0.53772	0.42622
1.12	0.50786	0.46227	0.54497	0.43269
1.124	0.51494	0.46989	0.5519	0.43945
1.128	0.52221	0.47801	0.55948	0.4465
1.132	0.52985	0.48613	0.56738	0.45356
1.136	0.53749	0.49498	0.57544	0.46091
1.14	0.54569	0.50384	0.58349	0.46884
1.144	0.55389	0.51294	0.59188	0.47678
1.148	0.56265	0.52253	0.6009	0.48471
1.152	0.57122	0.53237	0.60993	0.49324
1.156	0.57998	0.54197	0.61896	0.50176
1.16	0.5893	0.5523	0.62863	0.51117
1.164	0.59861	0.56312	0.6383	0.52028
1.168	0.6083	0.57394	0.64813	0.52998
1.172	0.61818	0.58501	0.65845	0.53909
1.176	0.62843	0.59658	0.66908	0.54968
1.18	0.63868	0.60838	0.67972	0.56026
1.184	0.64986	0.62093	0.69117	0.57143
1.188	0.66029	0.63298	0.70181	0.58201
1.192	0.67129	0.64553	0.71293	0.59347
1.196	0.68266	0.65807	0.72405	0.60494
1.2	0.69365	0.67111	0.73517	0.6167
1.204	0.70502	0.68366	0.74629	0.62875
1.208	0.7162	0.69719	0.75758	0.6408
1.212	0.72794	0.71072	0.76902	0.65373
1.216	0.73968	0.72424	0.78046	0.66637
1.22	0.75142	0.73704	0.79159	0.6796
1.224	0.76334	0.75081	0.80319	0.69312
1.228	0.77527	0.7641	0.81447	0.70694
1.232	0.78738	0.77787	0.8256	0.72105
1.236	0.79912	0.7914	0.8364	0.73516
1.24	0.81123	0.80493	0.84736	0.74927
1.244	0.82335	0.81846	0.85799	0.76396
1.248	0.83564	0.83248	0.86928	0.77895
1.252	0.84738	0.84552	0.87959	0.79424
1.256	0.85987	0.85905	0.89039	0.80982
1.26	0.87217	0.87258	0.90071	0.82569
1.264	0.88465	0.88586	0.91103	0.84127
1.268	0.89714	0.89841	0.92102	0.85744
1.272	0.90962	0.9112	0.93101	0.8736
1.276	0.92211	0.92399	0.94101	0.89095
1.28	0.93478	0.93703	0.951	0.908
1.284	0.94689	0.94908	0.96019	0.92475

0.876	0.30087	0.28615	0.31615	0.24849
0.88	0.30252	0.28783	0.31791	0.24979
0.884	0.30417	0.2895	0.31967	0.25119
0.888	0.30581	0.2909	0.32108	0.25274
0.892	0.30723	0.29257	0.3232	0.25429
0.896	0.30888	0.29425	0.32461	0.25544
0.9	0.31005	0.29564	0.32602	0.25694
0.904	0.3117	0.29704	0.32743	0.25874
0.908	0.31335	0.29844	0.32884	0.26034
0.912	0.31453	0.29955	0.3299	0.26219
0.916	0.31594	0.30095	0.33096	0.26384
0.92	0.31735	0.30207	0.33237	0.26564
0.924	0.31877	0.30346	0.33378	0.26769
0.928	0.32018	0.30486	0.33519	0.26969
0.932	0.32183	0.30597	0.33625	0.2715
0.936	0.32324	0.30737	0.33766	0.2738
0.94	0.32489	0.30877	0.33907	0.27625
0.944	0.32654	0.31016	0.34048	0.27845
0.948	0.32842	0.31156	0.34154	0.28085
0.952	0.3303	0.31323	0.3433	0.2833
0.956	0.33242	0.31519	0.34471	0.28595
0.96	0.33454	0.31714	0.34648	0.2888
0.964	0.33643	0.31854	0.34824	0.29105
0.968	0.33878	0.32077	0.35036	0.29405
0.972	0.34114	0.323	0.35247	0.29715
0.976	0.34373	0.32524	0.35459	0.30026
0.98	0.34655	0.32775	0.3567	0.30341
0.984	0.34914	0.33054	0.35953	0.30666
0.988	0.3522	0.33333	0.36235	0.31016
0.992	0.3555	0.33668	0.36517	0.31381
0.996	0.35856	0.33948	0.36799	0.31736
1	0.36186	0.34283	0.37116	0.32121
1.004	0.36539	0.34645	0.37434	0.32516
1.008	0.36892	0.35008	0.37787	0.32922
1.012	0.37245	0.35343	0.38139	0.33332
1.016	0.37646	0.35734	0.38492	0.33762
1.02	0.38046	0.36153	0.38845	0.34212
1.024	0.38423	0.36572	0.39233	0.34672
1.028	0.388	0.36991	0.39621	0.35102
1.032	0.39223	0.37437	0.40044	0.35617
1.036	0.39624	0.37884	0.40467	0.36133
1.04	0.40048	0.38358	0.4089	0.36633
1.044	0.40471	0.38833	0.41384	0.37173
1.048	0.40895	0.39308	0.41807	0.37708
1.052	0.41343	0.39838	0.42266	0.38268
1.056	0.41814	0.40341	0.42759	0.38829
1.06	0.42238	0.40843	0.43218	0.39429
1.064	0.42708	0.41346	0.43676	0.40029
1.068	0.43203	0.41904	0.44241	0.40679
1.072	0.4365	0.42434	0.44734	0.41279
1.076	0.44145	0.42965	0.45263	0.4188
1.08	0.44663	0.43551	0.45792	0.4253
1.084	0.45181	0.44165	0.46357	0.4318
1.088	0.45723	0.44724	0.46956	0.4383
1.092	0.46217	0.4531	0.47485	0.44431
1.096	0.46759	0.45952	0.4805	0.45081
1.1	0.47324	0.46566	0.48649	0.45781
1.104	0.47889	0.47208	0.49249	0.46431
1.108	0.48478	0.47878	0.49848	0.47081
1.112	0.49043	0.4852	0.50483	0.47732
1.116	0.49655	0.49218	0.51118	0.48432
1.12	0.50291	0.49916	0.51788	0.49182
1.124	0.50903	0.50586	0.52458	0.49832
1.128	0.51562	0.51312	0.53164	0.50533
1.132	0.52245	0.52066	0.53834	0.51283
1.136	0.52905	0.5282	0.54539	0.51983
1.14	0.53635	0.53573	0.55244	0.52733
1.144	0.54341	0.54355	0.55985	0.53534
1.148	0.55118	0.55165	0.56796	0.54334
1.152	0.55872	0.56002	0.57572	0.55184
1.156	0.56625	0.5684	0.58348	0.56035
1.16	0.57473	0.57733	0.59194	0.56935
1.164	0.58344	0.58626	0.60076	0.57835
1.168	0.59215	0.59548	0.60958	0.58786
1.172	0.6011	0.60469	0.61875	0.59786
1.176	0.61005	0.6139	0.62757	0.60736
1.18	0.6197	0.62395	0.63744	0.61787
1.184	0.6303	0.63484	0.64767	0.62887
1.188	0.63996	0.64461	0.65684	0.63987
1.192	0.65008	0.65522	0.66707	0.65138
1.196	0.66068	0.66583	0.67729	0.66288
1.2	0.67127	0.67672	0.68787	0.67489
1.204	0.68234	0.6876	0.69846	0.68689
1.208	0.69341	0.69849	0.70939	0.69989
1.212	0.70495	0.70994	0.72032	0.7129
1.216	0.71625	0.72138	0.73161	0.7259
1.22	0.72779	0.73283	0.74325	0.73941
1.224	0.74003	0.74511	0.75488	0.75391
1.228	0.75251	0.75684	0.76652	0.76792
1.232	0.76499	0.76912	0.77816	0.78142
1.236	0.777	0.78057	0.78945	0.79593
1.24	0.78948	0.79285	0.80179	0.81043
1.244	0.80267	0.80542	0.81414	0.82444
1.248	0.81586	0.81854	0.82648	0.83944
1.252	0.82881	0.83082	0.83882	0.85345
1.256	0.8427	0.84394	0.85152	0.86795
1.26	0.85612	0.85734	0.86457	0.88246
1.264	0.86955	0.87074	0.87727	0.89546
1.268	0.88297	0.88358	0.89032	0.90847
1.272	0.89615	0.89782	0.90301	0.92097
1.276	0.91075	0.91178	0.91677	0.93398
1.28	0.92512	0.92602	0.93017	0.94598
1.284	0.93948	0.9397	0.94357	0.95749

0.876	0.25037	0.21699	0.29189	0.40052
0.88	0.25196	0.21802	0.29363	0.40181
0.884	0.25355	0.21906	0.29537	0.40311
0.888	0.25482	0.22035	0.29716	0.4044
0.892	0.25642	0.22139	0.29895	0.4057
0.896	0.25833	0.22268	0.30059	0.40699
0.9	0.2596	0.22372	0.30196	0.40785
0.904	0.26119	0.22527	0.30379	0.40958
0.908	0.2631	0.22657	0.3053	0.41088
0.912	0.26438	0.22812	0.30666	0.41217
0.916	0.26629	0.22941	0.30822	0.4139
0.92	0.26788	0.23123	0.30944	0.41519
0.924	0.26979	0.2333	0.31114	0.41692
0.928	0.2717	0.23537	0.3125	0.41864
0.932	0.27361	0.23718	0.31382	0.41994
0.936	0.27552	0.23951	0.31523	0.42167
0.94	0.27775	0.2421	0.31664	0.42382
0.944	0.27998	0.24469	0.31824	0.42598
0.948	0.28221	0.24728	0.31961	0.42771
0.952	0.28412	0.25039	0.32125	0.42987
0.956	0.28699	0.25324	0.32285	0.43246
0.96	0.28954	0.2566	0.32464	0.43461
0.964	0.29176	0.25971	0.32615	0.43677
0.968	0.29463	0.26359	0.32808	0.43979
0.972	0.2975	0.26748	0.3301	0.44238
0.976	0.30036	0.2711	0.33203	0.44497
0.98	0.30355	0.2755	0.33415	0.44799
0.984	0.30673	0.27965	0.33627	0.45101
0.988	0.30992	0.28405	0.33862	0.45447
0.992	0.31342	0.28871	0.34102	0.45792
0.996	0.3166	0.29311	0.34337	0.46094
1	0.32011	0.29777	0.34596	0.46439
1.004	0.32361	0.30295	0.34864	0.46828
1.008	0.32743	0.30787	0.35137	0.47173
1.012	0.33125	0.31279	0.35425	0.47562
1.016	0.33507	0.31797	0.35721	0.4795
1.02	0.33921	0.32341	0.36041	0.48382
1.024	0.34335	0.32859	0.36418	0.4877
1.028	0.34718	0.33402	0.36747	0.49202
1.032	0.35163	0.33946	0.37123	0.49633
1.036	0.35577	0.34516	0.375	0.50065
1.04	0.36023	0.3506	0.37877	0.50539
1.044	0.36469	0.35629	0.383	0.50971
1.048	0.36947	0.36199	0.38677	0.51446
1.052	0.37424	0.36794	0.39147	0.51921
1.056	0.37934	0.37364	0.39618	0.52438
1.06	0.3838	0.3796	0.40041	0.52913
1.064	0.38921	0.38581	0.40512	0.53388
1.068	0.39462	0.39177	0.4103	0.53949
1.072	0.39972	0.39772	0.41547	0.54467
1.076	0.40513	0.40394	0.42065	0.54985
1.08	0.41087	0.40989	0.4263	0.55546
1.084	0.41692	0.41688	0.43195	0.5615
1.088	0.42297	0.42361	0.43806	0.56754
1.092	0.42902	0.42957	0.44371	0.57315
1.096	0.43507	0.4363	0.44983	0.57877
1.1	0.44176	0.44329	0.45642	0.58481
1.104	0.44812	0.45054	0.46254	0.59085
1.108	0.45481	0.45753	0.46913	0.59689
1.112	0.4615	0.46479	0.47619	0.60293
1.116	0.46882	0.47229	0.48325	0.60941
1.12	0.47615	0.47954	0.49078	0.61588
1.124	0.48347	0.48705	0.49831	0.62236
1.128	0.49112	0.49508	0.50584	0.62969
1.132	0.49876	0.50311	0.51431	0.6366
1.136	0.50704	0.51113	0.52278	0.64394
1.14	0.515	0.51968	0.53125	0.65084
1.144	0.5236	0.52822	0.54019	0.65818
1.148	0.5322	0.53755	0.5496	0.66595
1.152	0.54079	0.54661	0.55949	0.67372
1.156	0.55003	0.55593	0.5689	0.68148
1.16	0.55926	0.56577	0.57925	0.68968
1.164	0.56882	0.57561	0.58961	0.69789
1.168	0.57837	0.58545	0.60043	0.70652
1.172	0.58824	0.59555	0.61126	0.71515
1.176	0.59843	0.6059	0.62255	0.72378
1.18	0.60894	0.61652	0.63432	0.73284
1.184	0.62041	0.62791	0.64703	0.74277
1.188	0.63123	0.63827	0.65879	0.7514
1.192	0.64238	0.64966	0.6715	0.7609
1.196	0.65353	0.66106	0.68421	0.76996
1.2	0.66499	0.67245	0.69691	0.77946
1.204	0.67677	0.6841	0.70962	0.78852
1.208	0.68887	0.69601	0.7228	0.79801
1.212	0.70097	0.70844	0.73598	0.80751
1.216	0.71308	0.72061	0.74962	0.81744
1.22	0.72518	0.73278	0.7628	0.8265
1.224	0.73791	0.74573	0.77645	0.83599
1.228	0.75033	0.75867	0.78963	0.84592
1.232	0.76339	0.77136	0.80328	0.85542
1.236	0.77613	0.78353	0.81645	0.86405
1.24	0.78887	0.797	0.8301	0.87311
1.244	0.80192	0.8102	0.84375	0.88261
1.248	0.8153	0.82341	0.85646	0.89167
1.252	0.82867	0.83635	0.86869	0.90073
1.256	0.84237	0.84982	0.8814	0.90937
1.26	0.85638	0.86354	0.89411	0.91843
1.264	0.87007	0.87675	0.90587	0.92706
1.268	0.88345	0.89021	0.91764	0.93483
1.272	0.89746	0.90342	0.92893	0.94303
1.276	0.91179	0.91688	0.94023	0.95123
1.28	0.92612	0.93086	0.95058	0.95986
1.284	0.94013	0.94433	0.96094	0.96763

0.876	0.35978	0.30256	0.26872
0.88	0.36171	0.30373	0.26941
0.884	0.36354	0.3049	0.2701
0.888	0.36525	0.30607	0.27079
0.892	0.36707	0.30747	0.27148
0.896	0.36884	0.30864	0.27216
0.9	0.37044	0.31005	0.27251
0.904	0.3722	0.31145	0.2732
0.908	0.37375	0.31285	0.27389
0.912	0.37546	0.31402	0.27457
0.916	0.37695	0.31543	0.27526
0.92	0.37839	0.31683	0.27561
0.924	0.37993	0.31847	0.2763
0.928	0.38137	0.31987	0.27698
0.932	0.38281	0.32128	0.27767
0.936	0.38419	0.32291	0.27836
0.94	0.38573	0.32479	0.27939
0.944	0.38717	0.32666	0.28043
0.948	0.3886	0.32853	0.28112
0.952	0.39009	0.33017	0.28215
0.956	0.39181	0.33227	0.28318
0.96	0.3933	0.33461	0.28456
0.964	0.39462	0.33672	0.28594
0.968	0.39644	0.33906	0.28731
0.972	0.39821	0.34163	0.28869
0.976	0.39998	0.34444	0.29041
0.98	0.40186	0.34725	0.29248
0.984	0.40362	0.35005	0.2942
0.988	0.40567	0.35286	0.29626
0.992	0.40754	0.3559	0.29833
0.996	0.40947	0.35871	0.3004
1	0.41174	0.36199	0.30246
1.004	0.41389	0.36526	0.30487
1.008	0.41599	0.3683	0.30728
1.012	0.41836	0.37181	0.30969
1.016	0.42057	0.37532	0.31245
1.02	0.42344	0.37883	0.3152
1.024	0.42609	0.38281	0.3183
1.028	0.42852	0.38608	0.32105
1.032	0.43184	0.39006	0.32415
1.036	0.4346	0.3938	0.32725
1.04	0.43736	0.39778	0.33035
1.044	0.44067	0.40176	0.33379
1.048	0.44398	0.40574	0.33758
1.052	0.4473	0.40995	0.34102
1.056	0.45116	0.41439	0.34481
1.06	0.45448	0.4186	0.34894
1.064	0.45834	0.42305	0.35273
1.068	0.46276	0.42773	0.3572
1.072	0.46662	0.43241	0.36202
1.076	0.47104	0.43732	0.3665
1.08	0.47546	0.44247	0.37132
1.084	0.48043	0.44808	0.37683
1.088	0.4854	0.45323	0.38234
1.092	0.49036	0.45861	0.38785
1.096	0.49533	0.46446	0.3937
1.1	0.50086	0.47054	0.3999
1.104	0.50693	0.47639	0.40644
1.108	0.513	0.48248	0.41298
1.112	0.51908	0.48903	0.41952
1.116	0.5257	0.49581	0.4271
1.12	0.53233	0.50283	0.43467
1.124	0.53895	0.50962	0.44224
1.128	0.54668	0.5171	0.45051
1.132	0.55441	0.52459	0.45912
1.136	0.56214	0.53254	0.46772
1.14	0.57043	0.54026	0.47667
1.144	0.57871	0.54845	0.48597
1.148	0.58754	0.55711	0.4963
1.152	0.59693	0.566	0.50663
1.156	0.60687	0.57466	0.51696
1.16	0.61681	0.58402	0.52797
1.164	0.6273	0.59337	0.53934
1.168	0.63834	0.6032	0.55104
1.172	0.64883	0.61303	0.56309
1.176	0.66043	0.62379	0.57514
1.18	0.67202	0.63455	0.58754
1.184	0.68472	0.64602	0.60096
1.188	0.69632	0.65678	0.61405
1.192	0.70846	0.66848	0.62747
1.196	0.72116	0.67971	0.6409
1.2	0.73386	0.6914	0.65467
1.204	0.74712	0.7031	0.66879
1.208	0.75981	0.71503	0.68291
1.212	0.77362	0.72767	0.69668
1.216	0.78687	0.74007	0.71148
1.22	0.79957	0.752	0.72525
1.224	0.81282	0.76487	0.73971
1.228	0.82607	0.7775	0.75452
1.232	0.83932	0.79037	0.76898
1.236	0.85202	0.80277	0.7831
1.24	0.86417	0.81564	0.7979
1.244	0.87632	0.82851	0.81202
1.248	0.88847	0.84114	0.82648
1.252	0.90006	0.85354	0.84025
1.256	0.91166	0.86641	0.85436
1.26	0.92215	0.87904	0.86848
1.264	0.93264	0.89144	0.88225
1.268	0.94202	0.90384	0.89568
1.272	0.95141	0.91577	0.90911
1.276	0.96025	0.92794	0.92253
1.28	0.96853	0.94034	0.93562
1.284	0.97626	0.95204	0.94836

1.288	0.96049	0.96261	0.9705	0.94386	1.288	0.95479	0.95477	0.95803	0.96949	1.288	0.95542	0.95857	0.97129	0.97669	1.288	0.98344	0.96444	0.96178
1.292	0.97354	0.97491	0.9805	0.96208	1.292	0.96962	0.96957	0.97143	0.97999	1.292	0.97007	0.97229	0.98117	0.98446	1.292	0.98951	0.97637	0.97418
1.296	0.98658	0.9877	0.99017	0.98031	1.296	0.98493	0.98492	0.98554	0.9905	1.296	0.98503	0.98628	0.99059	0.99223	1.296	0.99503	0.98807	0.98726
1.3	1	1	1	1	1.3	1	1	1	1	1.3	1	1	1	1	1.3	1	1	1

Raw table S2. Electrochemical fingerprints of *Acer sinopurpurascens*

Acer sinopurpurascens

**Ethanol-ABS (Spring)**					**Ethanol-ABS (Summer)**					**Ethanol-ABS (Autumn)**				
0.004	0.10116	0.13569	0.18921		0.004	0.12129	0.12074	0.1304	0.05296	0.004	0.1019	0.07714	0.06822	0.12051
0.008	0.0966	0.12854	0.18326		0.008	0.116	0.11522	0.12476	0.04965	0.008	0.09711	0.07315	0.06399	0.1111
0.012	0.09229	0.12264	0.17751		0.012	0.11198	0.11031	0.11977	0.047	0.012	0.09292	0.0698	0.06053	0.10292
0.016	0.08849	0.11767	0.17295		0.016	0.10838	0.10632	0.11564	0.0448	0.016	0.08924	0.067	0.05782	0.09638
0.02	0.08494	0.11302	0.16858		0.02	0.10499	0.10263	0.11174	0.04275	0.02	0.08593	0.06456	0.05522	0.09024
0.024	0.0819	0.10898	0.16442		0.024	0.10203	0.09925	0.10827	0.04117	0.024	0.08301	0.06226	0.05305	0.08574
0.028	0.07912	0.10525	0.16085		0.028	0.09949	0.09649	0.10501	0.03966	0.028	0.08074	0.06026	0.05128	0.08124
0.032	0.07582	0.10215	0.15728		0.032	0.09716	0.09373	0.10197	0.03836	0.032	0.07842	0.05852	0.04944	0.07756
0.036	0.07304	0.09873	0.15371		0.036	0.09483	0.09096	0.09915	0.03701	0.036	0.07607	0.05688	0.04782	0.07388
0.04	0.07076	0.09593	0.15054		0.04	0.09251	0.08851	0.09655	0.03575	0.04	0.07423	0.05524	0.04626	0.07142
0.044	0.06822	0.09314	0.14697		0.044	0.0906	0.08605	0.09395	0.03464	0.044	0.07215	0.05378	0.04496	0.06856
0.048	0.06569	0.09065	0.14359		0.048	0.08848	0.0839	0.09134	0.03357	0.048	0.07028	0.05231	0.04366	0.0661
0.052	0.06366	0.08786	0.14022		0.052	0.08637	0.08145	0.08874	0.03247	0.052	0.068	0.05075	0.04218	0.06283
0.056	0.06138	0.08538	0.13705		0.056	0.08446	0.0793	0.08614	0.0314	0.056	0.06592	0.04934	0.04103	0.06038
0.06	0.05961	0.0832	0.13407		0.06	0.08277	0.07746	0.08397	0.03045	0.06	0.06437	0.0482	0.03965	0.05833
0.064	0.05733	0.08103	0.1307		0.064	0.08044	0.075	0.08158	0.02954	0.064	0.06217	0.04693	0.03839	0.05588
0.068	0.0553	0.07854	0.12753		0.068	0.07854	0.07285	0.07919	0.02859	0.068	0.06001	0.0456	0.0372	0.05383
0.072	0.05353	0.07662	0.12436		0.072	0.07663	0.0707	0.07681	0.02758	0.072	0.05834	0.04435	0.03604	0.05179
0.076	0.05176	0.07454	0.12158		0.076	0.07473	0.06886	0.0742	0.0267	0.076	0.05654	0.04308	0.03492	0.05015
0.08	0.04998	0.07264	0.11841		0.08	0.07304	0.0664	0.07203	0.02579	0.08	0.0543	0.04186	0.03377	0.04852
0.084	0.04846	0.07056	0.11543		0.084	0.07092	0.06456	0.06943	0.02487	0.084	0.05247	0.04072	0.03239	0.04647
0.088	0.04669	0.0686	0.11206		0.088	0.06901	0.06241	0.06726	0.02402	0.088	0.05035	0.03958	0.03127	0.04442
0.092	0.04517	0.0669	0.10928		0.092	0.06732	0.06057	0.06509	0.02323	0.092	0.04843	0.03842	0.03026	0.04279
0.096	0.0439	0.065	0.10631		0.096	0.0652	0.05873	0.0627	0.02244	0.096	0.04644	0.03737	0.02907	0.04115
0.1	0.04213	0.06317	0.10313		0.1	0.0633	0.05627	0.06032	0.02159	0.1	0.04428	0.0362	0.02788	0.03952
0.104	0.04086	0.06155	0.10036		0.104	0.0614	0.05443	0.05815	0.02081	0.104	0.04249	0.03509	0.02676	0.03829
0.108	0.03934	0.05972	0.09718		0.108	0.05949	0.05259	0.05576	0.01999	0.108	0.04029	0.03393	0.02557	0.03706
0.112	0.03808	0.05808	0.09421		0.112	0.05737	0.05044	0.05337	0.01932	0.112	0.03821	0.0329	0.02434	0.03543
0.116	0.0363	0.05634	0.09123		0.116	0.05547	0.0486	0.0512	0.0185	0.116	0.03614	0.03185	0.02326	0.03379
0.12	0.03504	0.05454	0.08826		0.12	0.05357	0.04645	0.04903	0.01768	0.12	0.03434	0.03085	0.02207	0.03256
0.124	0.03352	0.05283	0.08528		0.124	0.05166	0.04461	0.04686	0.01715	0.124	0.0323	0.02983	0.02098	0.03093
0.128	0.03225	0.05093	0.08231		0.128	0.04976	0.04277	0.04448	0.01658	0.128	0.03027	0.02869	0.01986	0.02929
0.132	0.03073	0.04904	0.07933		0.132	0.04785	0.04062	0.04231	0.01582	0.132	0.02859	0.02764	0.01882	0.02765
0.136	0.02921	0.04711	0.07636		0.136	0.04595	0.03877	0.04014	0.01507	0.136	0.02659	0.02678	0.01773	0.02602
0.14	0.02769	0.04534	0.07358		0.14	0.04383	0.03724	0.03797	0.01447	0.14	0.02484	0.02564	0.01676	0.02438
0.144	0.02642	0.04342	0.07061		0.144	0.04193	0.03509	0.03602	0.01387	0.144	0.02296	0.02467	0.01571	0.02315
0.148	0.02465	0.04149	0.06783		0.148	0.03981	0.03325	0.03363	0.01318	0.148	0.02116	0.02364	0.01477	0.02152
0.152	0.02338	0.0395	0.06466		0.152	0.0379	0.03141	0.03168	0.01248	0.152	0.01949	0.02248	0.01372	0.02029
0.156	0.02136	0.03752	0.06168		0.156	0.03579	0.02926	0.02951	0.01179	0.156	0.01785	0.02145	0.01289	0.01865
0.16	0.02009	0.03559	0.05871		0.16	0.03388	0.02763	0.02755	0.01116	0.16	0.01633	0.02035	0.01192	0.01743
0.164	0.01857	0.03332	0.05553		0.164	0.03177	0.0257	0.02539	0.01037	0.164	0.01469	0.01921	0.01094	0.01579
0.168	0.01705	0.03118	0.05256		0.168	0.02986	0.02398	0.02343	0.00974	0.168	0.01306	0.01813	0.01004	0.01456
0.172	0.01553	0.02919	0.04958		0.172	0.02796	0.02217	0.02148	0.00898	0.172	0.0117	0.01696	0.00914	0.01334
0.176	0.01401	0.02711	0.04641		0.176	0.02584	0.02029	0.01953	0.00823	0.176	0.01026	0.01586	0.00816	0.01203
0.18	0.01249	0.02494	0.04324		0.18	0.02372	0.01854	0.01757	0.00738	0.18	0.00886	0.01455	0.00726	0.01047
0.184	0.01072	0.02276	0.04026		0.184	0.02182	0.01679	0.01562	0.00656	0.184	0.00759	0.01331	0.00636	0.00888
0.188	0.0097	0.02062	0.03709		0.188	0.01991	0.0151	0.01389	0.00589	0.188	0.00631	0.01225	0.00556	0.00757
0.192	0.00844	0.01857	0.03411		0.192	0.01801	0.01357	0.01215	0.00514	0.192	0.00519	0.011	0.00462	0.00634
0.196	0.00692	0.0164	0.03114		0.196	0.01611	0.0117	0.01041	0.00422	0.196	0.00395	0.00981	0.00383	0.00503
0.2	0.00565	0.01441	0.02816		0.2	0.0142	0.01035	0.0089	0.0035	0.2	0.00303	0.00862	0.00307	0.00389
0.204	0.00476	0.01252	0.02539		0.204	0.01251	0.00881	0.00738	0.0029	0.204	0.0022	0.00751	0.00246	0.00319
0.208	0.0037	0.0105	0.02281		0.208	0.01071	0.00737	0.00586	0.00224	0.208	0.00132	0.00624	0.00173	0.002
0.212	0.00276	0.00879	0.02003		0.212	0.0091	0.00589	0.00456	0.00151	0.212	7.19E-04	0.00513	0.00119	0.00115
0.216	0.00195	0.00711	0.01765		0.216	0.0076	0.00476	0.00347	0.00107	0.216	2.80E-04	0.00413	6.50E-04	7.77E-04
0.22	0.00137	0.00568	0.01527		0.22	0.00616	0.00365	0.0026	7.25E-04	0.22	0	0.0031	3.25E-04	1.23E-04
0.224	8.11E-04	0.00435	0.01289		0.224	0.00502	0.00273	0.00174	3.15E-04	0.224	1.20E-04	0.00233	1.08E-04	0
0.228	3.55E-04	0.00311	0.01071		0.228	0.00372	0.0019	8.68E-04	3.15E-05	0.228	7.99E-05	0.00152	0	0
0.232	7.60E-05	0.00217	0.00893		0.232	0.00281	0.00126	4.34E-04	0	0.232	5.99E-04	9.42E-04	1.08E-04	4.91E-04
0.236	0	0.00127	0.00714		0.236	0.00186	6.14E-04	0	1.26E-04	0.236	0.00144	4.71E-04	2.53E-04	0.00123
0.24	0	7.14E-04	0.00555		0.24	0.00119	2.46E-04	0	3.78E-04	0.24	0.00252	5.54E-05	5.78E-04	0.00213
0.244	2.03E-04	3.42E-04	0.00397		0.244	6.56E-04	9.21E-05	0	8.83E-04	0.244	0.00391	0	0.00134	0.00356
0.248	3.55E-04	0	0.00278		0.248	2.33E-04	0	2.17E-04	0.00148	0.248	0.00583	2.77E-05	0.00228	0.00536
0.252	0.00111	2.17E-04	0.00179		0.252	4.23E-05	3.68E-04	8.68E-04	0.00255	0.252	0.00815	2.77E-04	0.00339	0.00757
0.256	0.00182	4.97E-04	9.92E-04		0.256	0	7.37E-04	0.0013	0.00381	0.256	0.01094	7.76E-04	0.00509	0.01092
0.26	0.00271	8.70E-04	3.97E-04		0.26	2.12E-05	0.00126	0.00195	0.00536	0.26	0.01421	0.00147	0.00683	0.01456
0.264	0.004	0.0018	1.98E-04		0.264	3.39E-04	0.00218	0.00282	0.00731	0.264	0.01813	0.00247	0.00921	0.01947
0.268	0.00529	0.0028	0		0.268	8.47E-04	0.00335	0.00412	0.00943	0.268	0.02252	0.00385	0.01195	0.0252
0.272	0.00692	0.00407	0		0.272	0.00159	0.00439	0.00499	0.01192	0.272	0.02751	0.00541	0.01506	0.03215
0.276	0.00869	0.00537	1.98E-04		0.276	0.00229	0.00593	0.00651	0.01485	0.276	0.03298	0.00721	0.01878	0.04074
0.28	0.01072	0.00677	5.95E-04		0.28	0.00315	0.0074	0.00781	0.01797	0.28	0.03889	0.00909	0.02311	0.05015
0.284	0.013	0.00863	0.00159		0.284	0.00417	0.00924	0.00955	0.02128	0.284	0.04536	0.0115	0.0281	0.0612
0.288	0.01553	0.01056	0.00238		0.288	0.00535	0.01105	0.01107	0.02506	0.288	0.05215	0.01422	0.03377	0.07388
0.292	0.01857	0.01239	0.00357		0.292	0.00658	0.01314	0.0128	0.02881	0.292	0.05906	0.01702	0.04034	0.08697
0.296	0.02212	0.01463	0.00456		0.296	0.00811	0.0155	0.01475	0.03285	0.296	0.06616	0.02001	0.04756	0.10088
0.3	0.02617	0.01686	0.00575		0.3	0.00976	0.01771	0.01671	0.03685	0.3	0.07331	0.02334	0.05558	0.11478
0.304	0.03073	0.01929	0.00734		0.304	0.01136	0.02017	0.01909	0.04082	0.304	0.0801	0.0268	0.06417	0.12787
0.308	0.03605	0.02177	0.00893		0.308	0.01335	0.02266	0.02126	0.04483	0.308	0.08621	0.0303	0.07342	0.13892
0.312	0.04238	0.02429	0.01091		0.312	0.01547	0.0252	0.02408	0.04861	0.312	0.09184	0.03401	0.08303	0.14669
0.316	0.04998	0.02742	0.01289		0.316	0.01801	0.02797	0.02712	0.05264	0.316	0.09707	0.03806	0.0926	0.15119
0.32	0.0586	0.03068	0.01527		0.32	0.02076	0.03079	0.03081	0.05674	0.32	0.10158	0.04224	0.10173	0.15078
0.324	0.06873	0.03426	0.01805		0.324	0.02394	0.03386	0.03471	0.06116	0.324	0.10549	0.04649	0.10925	0.14587
0.328	0.08064	0.03848	0.02142		0.328	0.02775	0.03755	0.0397	0.06614	0.328	0.10913	0.05111	0.11585	0.1381
0.332	0.09381	0.04295	0.02519		0.332	0.03198	0.04123	0.04513	0.07169	0.332	0.11284	0.05619	0.12037	0.12869
0.336	0.1085	0.04808	0.02955		0.336	0.03727	0.04584	0.0512	0.07821	0.336	0.11663	0.06143	0.123	0.11928
0.34	0.12472	0.05367	0.03451		0.34	0.04319	0.05075	0.05858	0.0859	0.34	0.12087	0.06727	0.12358	0.11274
0.344	0.14195	0.05994	0.04026		0.344	0.05018	0.05627	0.06639	0.09486	0.344	0.12546	0.07373	0.1229	0.10947
0.348	0.16019	0.06659	0.04661		0.348	0.05801	0.06272	0.07485	0.10516	0.348	0.13117	0.08086	0.1217	0.10987
0.352	0.17893	0.0737	0.05375		0.352	0.06669	0.07009	0.0844	0.11705	0.352	0.1376	0.08859	0.12051	0.11397
0.356	0.19717	0.08103	0.06129		0.356	0.07621	0.07807	0.09373	0.1301	0.356	0.14487	0.09688	0.12026	0.12092
0.36	0.21465	0.08848	0.06962		0.36	0.08658	0.08697	0.10393	0.14457	0.36	0.15309	0.10594	0.12102	0.13115
0.364	0.23137	0.09593	0.07834		0.364	0.09759	0.0971	0.11434	0.16017	0.364	0.16227	0.11576	0.12333	0.14465

0.368	0.24556	0.10308	0.08707	0.368	0.1088	0.10754	0.12411	0.17597	0.368	0.17146	0.12573	0.12694	0.15978
0.372	0.25721	0.1096	0.0958	0.372	0.11981	0.11767	0.13343	0.19154	0.372	0.18064	0.13571	0.13192	0.17614
0.376	0.26583	0.11519	0.10393	0.376	0.13039	0.12842	0.14168	0.20686	0.376	0.18983	0.14569	0.13832	0.19373
0.38	0.2714	0.12016	0.11166	0.38	0.14055	0.13886	0.14927	0.22073	0.38	0.19781	0.15512	0.1459	0.21214
0.384	0.27317	0.12358	0.11821	0.384	0.14922	0.14868	0.15535	0.23302	0.384	0.205	0.16399	0.1545	0.23014
0.388	0.27089	0.12606	0.12376	0.388	0.15663	0.15758	0.15969	0.24248	0.388	0.21019	0.17147	0.16338	0.24691
0.392	0.26583	0.1273	0.12773	0.392	0.16235	0.16557	0.16273	0.24973	0.392	0.21378	0.17757	0.17255	0.26205
0.396	0.25772	0.12792	0.13031	0.396	0.16594	0.17232	0.16424	0.2532	0.396	0.21498	0.18173	0.18187	0.27473
0.4	0.24733	0.12699	0.1315	0.4	0.16764	0.17723	0.16403	0.25383	0.4	0.21378	0.1845	0.19043	0.28373
0.404	0.23492	0.12544	0.1307	0.404	0.16764	0.18092	0.16273	0.25068	0.404	0.21019	0.18505	0.19845	0.28986
0.408	0.22175	0.12358	0.12892	0.408	0.16552	0.18307	0.16034	0.245	0.408	0.205	0.18395	0.20527	0.29191
0.412	0.20807	0.12109	0.12555	0.412	0.16213	0.18399	0.15687	0.23681	0.412	0.19781	0.1809	0.21091	0.29068
0.416	0.19413	0.1183	0.12098	0.416	0.15705	0.18337	0.15274	0.2264	0.416	0.18943	0.17646	0.21524	0.28577
0.42	0.18147	0.1155	0.11563	0.42	0.15134	0.18184	0.14841	0.21537	0.42	0.18024	0.17064	0.21849	0.27841
0.424	0.16956	0.11333	0.11027	0.424	0.14478	0.18	0.14407	0.20368	0.424	0.17106	0.16426	0.2203	0.269
0.428	0.15917	0.11115	0.10432	0.428	0.13822	0.17723	0.13973	0.19207	0.428	0.16188	0.15733	0.22138	0.25877
0.432	0.1498	0.1096	0.09837	0.432	0.13145	0.17447	0.13539	0.18101	0.432	0.15269	0.14985	0.22138	0.24732
0.436	0.14195	0.10836	0.09242	0.436	0.12489	0.17171	0.13148	0.17089	0.436	0.14447	0.14264	0.22066	0.23587
0.44	0.13511	0.10743	0.08707	0.44	0.11896	0.16894	0.12823	0.16191	0.44	0.13696	0.13571	0.21993	0.22482
0.444	0.12978	0.1068	0.08211	0.444	0.11367	0.16649	0.12541	0.15447	0.444	0.13049	0.12906	0.21885	0.2146
0.448	0.12548	0.1068	0.07775	0.448	0.1088	0.16403	0.12302	0.14854	0.448	0.12482	0.12307	0.21777	0.20478
0.452	0.12168	0.10712	0.07358	0.452	0.10457	0.16219	0.12085	0.14387	0.452	0.12015	0.11759	0.21632	0.19619
0.456	0.11914	0.10805	0.07021	0.456	0.10097	0.16096	0.11955	0.14075	0.456	0.11655	0.11296	0.2156	0.18923
0.46	0.11762	0.10929	0.06743	0.46	0.09822	0.16004	0.11868	0.13861	0.46	0.11388	0.10885	0.21452	0.1831
0.464	0.11636	0.11053	0.06525	0.464	0.09589	0.15973	0.11825	0.13757	0.464	0.11176	0.1055	0.21343	0.17819
0.468	0.11585	0.11239	0.06327	0.468	0.0942	0.16004	0.11825	0.13754	0.468	0.11049	0.10262	0.21307	0.17451
0.472	0.11585	0.11426	0.06208	0.472	0.09314	0.16035	0.11868	0.13811	0.472	0.10981	0.10048	0.21235	0.17205
0.476	0.11636	0.11674	0.06109	0.476	0.09208	0.16096	0.11977	0.13943	0.476	0.10989	0.09885	0.21235	0.17042
0.48	0.11712	0.11954	0.06089	0.48	0.09187	0.1625	0.12107	0.1412	0.48	0.11033	0.09771	0.21307	0.1696
0.484	0.11864	0.12233	0.06049	0.484	0.09187	0.16372	0.1228	0.14321	0.484	0.11116	0.09691	0.2138	0.17001
0.488	0.12016	0.12544	0.06069	0.488	0.09229	0.16587	0.12454	0.14577	0.488	0.11248	0.09649	0.21524	0.17123
0.492	0.12218	0.12885	0.06109	0.492	0.09272	0.16802	0.12693	0.1487	0.492	0.11424	0.09652	0.21705	0.17287
0.496	0.12446	0.13196	0.06168	0.496	0.09356	0.17017	0.12953	0.15185	0.496	0.11636	0.09685	0.21957	0.17492
0.5	0.127	0.13569	0.06267	0.5	0.09441	0.17263	0.13235	0.15519	0.5	0.11911	0.09741	0.22246	0.17819
0.504	0.12978	0.13941	0.06367	0.504	0.09568	0.17539	0.13539	0.15888	0.504	0.12187	0.09824	0.22571	0.18146
0.508	0.13308	0.14314	0.06525	0.508	0.09695	0.17846	0.13842	0.16273	0.508	0.12534	0.09935	0.22969	0.18555
0.512	0.13637	0.14718	0.06664	0.512	0.09843	0.18122	0.14168	0.1667	0.512	0.12917	0.10079	0.23402	0.19005
0.516	0.13967	0.15091	0.06843	0.516	0.09991	0.18429	0.14472	0.17061	0.516	0.13321	0.10206	0.23871	0.19537
0.52	0.14347	0.15525	0.07041	0.52	0.10182	0.18767	0.14797	0.17499	0.52	0.13816	0.10384	0.24377	0.2011
0.524	0.14727	0.15929	0.07259	0.524	0.10372	0.19105	0.15144	0.17924	0.524	0.14331	0.10583	0.24883	0.20723
0.528	0.15157	0.16364	0.07517	0.528	0.10563	0.19504	0.15491	0.18391	0.528	0.1487	0.10808	0.2546	0.21419
0.532	0.15588	0.16799	0.07755	0.532	0.10774	0.19872	0.1586	0.18854	0.532	0.15469	0.11027	0.26002	0.22114
0.536	0.16019	0.17233	0.08013	0.536	0.11007	0.20241	0.16229	0.1933	0.536	0.16068	0.1126	0.2658	0.2285
0.54	0.165	0.1773	0.0833	0.54	0.11261	0.2067	0.1662	0.19822	0.54	0.16747	0.11528	0.27194	0.23628
0.544	0.17007	0.18165	0.08647	0.544	0.11536	0.2107	0.17032	0.20333	0.544	0.17385	0.11822	0.2788	0.24446
0.548	0.17488	0.18631	0.08965	0.548	0.11811	0.21499	0.17422	0.20844	0.548	0.18064	0.12105	0.28494	0.25264
0.552	0.17995	0.19128	0.09302	0.552	0.12108	0.21929	0.17856	0.21348	0.552	0.18743	0.12415	0.2918	0.26123
0.556	0.18527	0.19625	0.09659	0.556	0.12404	0.2239	0.1829	0.21884	0.556	0.19422	0.1274	0.29866	0.26982
0.56	0.19084	0.20153	0.10016	0.56	0.12743	0.22881	0.18746	0.2242	0.56	0.20101	0.13072	0.30589	0.27964
0.564	0.19616	0.2065	0.10413	0.564	0.13081	0.23372	0.1918	0.22924	0.564	0.20739	0.13433	0.31275	0.28864
0.568	0.20199	0.21209	0.1077	0.568	0.13441	0.23863	0.19657	0.2346	0.568	0.21338	0.13765	0.31997	0.29804
0.572	0.20781	0.21737	0.11186	0.572	0.13822	0.24385	0.20135	0.23996	0.572	0.21937	0.14181	0.32756	0.30745
0.576	0.21339	0.22296	0.11603	0.576	0.14182	0.24938	0.20634	0.245	0.576	0.22417	0.14514	0.33478	0.31727
0.58	0.21896	0.22824	0.11999	0.58	0.14563	0.25429	0.21111	0.25036	0.58	0.22816	0.14874	0.342	0.32709
0.584	0.22479	0.23383	0.12416	0.584	0.14965	0.25982	0.2161	0.25541	0.584	0.23175	0.15235	0.34922	0.33691
0.588	0.23036	0.23973	0.12832	0.588	0.15367	0.26504	0.22109	0.26045	0.588	0.23455	0.15595	0.35645	0.34713
0.592	0.23593	0.24501	0.13249	0.592	0.15769	0.27056	0.22608	0.26518	0.592	0.23614	0.15983	0.36367	0.35654
0.596	0.241	0.2506	0.13645	0.596	0.16171	0.27578	0.23064	0.26991	0.596	0.23734	0.16343	0.37053	0.36636
0.6	0.24632	0.25619	0.14042	0.6	0.16552	0.28131	0.23563	0.27464	0.6	0.23734	0.16704	0.37739	0.37618
0.604	0.25164	0.26147	0.14439	0.604	0.16954	0.28683	0.2404	0.27936	0.604	0.23734	0.17092	0.38389	0.3864
0.608	0.25671	0.26675	0.14796	0.608	0.17356	0.29205	0.24539	0.28378	0.608	0.23614	0.17452	0.39039	0.39581
0.612	0.26101	0.27172	0.15153	0.612	0.17737	0.29727	0.24973	0.28819	0.612	0.23415	0.17812	0.39689	0.40563
0.616	0.26583	0.27669	0.1549	0.616	0.18118	0.30218	0.25429	0.29292	0.616	0.23215	0.18173	0.40303	0.41586
0.62	0.27039	0.28134	0.15827	0.62	0.18499	0.30709	0.25884	0.29733	0.62	0.22976	0.18561	0.40917	0.42526
0.624	0.27495	0.28569	0.16125	0.624	0.18859	0.31201	0.26296	0.30175	0.624	0.22696	0.18921	0.41531	0.43508
0.628	0.279	0.28973	0.16442	0.628	0.19197	0.31661	0.26687	0.30648	0.628	0.22417	0.19282	0.42073	0.4449
0.632	0.28305	0.29346	0.1672	0.632	0.19536	0.32091	0.27056	0.3112	0.632	0.22097	0.19642	0.42651	0.45513
0.636	0.28711	0.29718	0.17017	0.636	0.19854	0.32521	0.27446	0.31625	0.636	0.21858	0.20002	0.43192	0.46535
0.64	0.29091	0.30029	0.17295	0.64	0.2015	0.32889	0.27793	0.32129	0.64	0.21578	0.20363	0.43698	0.47517
0.644	0.29445	0.30308	0.17553	0.644	0.20446	0.33258	0.28097	0.32602	0.644	0.21338	0.20723	0.44204	0.48499
0.648	0.29775	0.30588	0.1781	0.648	0.207	0.33626	0.28401	0.33138	0.648	0.21139	0.21083	0.44637	0.4948
0.652	0.30079	0.30836	0.18048	0.652	0.20954	0.33964	0.28683	0.33642	0.652	0.20939	0.21444	0.45107	0.50462
0.656	0.30357	0.31054	0.18306	0.656	0.21187	0.3424	0.28943	0.34178	0.656	0.20739	0.21776	0.4554	0.51444
0.66	0.30585	0.31209	0.18524	0.66	0.21398	0.34516	0.29139	0.34651	0.66	0.2062	0.22081	0.45937	0.52344
0.664	0.30737	0.31364	0.18782	0.664	0.2161	0.34793	0.29356	0.35124	0.664	0.2054	0.22386	0.46371	0.53244
0.668	0.30889	0.31489	0.19	0.668	0.21801	0.35038	0.29551	0.35565	0.668	0.2046	0.22719	0.46768	0.54103
0.672	0.30991	0.31613	0.19258	0.672	0.22033	0.35315	0.29746	0.36038	0.672	0.2042	0.22996	0.47129	0.55003
0.676	0.31016	0.31675	0.19457	0.676	0.22203	0.35499	0.29876	0.36385	0.676	0.2038	0.23245	0.47454	0.55739
0.68	0.30991	0.31706	0.19695	0.68	0.22372	0.35714	0.3005	0.36732	0.68	0.2038	0.23467	0.47779	0.56475
0.684	0.3094	0.31706	0.19913	0.684	0.22541	0.35929	0.30158	0.37047	0.684	0.2038	0.23661	0.48068	0.5713
0.688	0.30839	0.31706	0.20171	0.688	0.22711	0.36113	0.30267	0.37299	0.688	0.2042	0.23827	0.48321	0.57744
0.692	0.30712	0.31675	0.20389	0.692	0.22901	0.36266	0.30332	0.3752	0.692	0.2046	0.23994	0.48537	0.58275
0.696	0.3056	0.31644	0.20627	0.696	0.2307	0.36481	0.30419	0.37677	0.696	0.205	0.24105	0.48682	0.58644
0.7	0.30433	0.31613	0.20865	0.7	0.23261	0.36635	0.30506	0.37835	0.7	0.2058	0.24243	0.48862	0.59053
0.704	0.30281	0.31551	0.21123	0.704	0.23473	0.36819	0.30571	0.37993	0.704	0.207	0.24354	0.48935	0.59298
0.708	0.30104	0.31489	0.21341	0.708	0.23663	0.37003	0.30636	0.38087	0.708	0.20779	0.24437	0.48971	0.59462
0.712	0.29977	0.31426	0.21618	0.712	0.23896	0.37187	0.30701	0.38213	0.712	0.20899	0.24548	0.49007	0.59625
0.716	0.29876	0.31395	0.21896	0.716	0.24129	0.37371	0.30766	0.38308	0.716	0.21059	0.24659	0.49007	0.59707
0.72	0.29775	0.31364	0.22174	0.72	0.24383	0.37586	0.30853	0.38402	0.72	0.21219	0.24742	0.48971	0.59707
0.724	0.29673	0.31333	0.22432	0.724	0.24658	0.3774	0.30896	0.38528	0.724	0.21378	0.24853	0.48862	0.59625
0.728	0.29648	0.31302	0.22729	0.728	0.24912	0.37985	0.30983	0.38655	0.728	0.21618	0.24964	0.4879	0.59584
0.732	0.29648	0.31333	0.23007	0.732	0.25208	0.382	0.31091	0.38781	0.732	0.21858	0.2513	0.48682	0.59543
0.736	0.29749	0.31426	0.23344	0.736	0.25504	0.38415	0.31222	0.38938	0.736	0.22097	0.25297	0.48573	0.59503
0.74	0.29851	0.31489	0.23641	0.74	0.25779	0.3863	0.31308	0.39064	0.74	0.22337	0.25491	0.48429	0.5938
0.744	0.30028	0.31582	0.23959	0.744	0.26097	0.38876	0.31438	0.39254	0.744	0.22616	0.25712	0.48321	0.59339
0.748	0.30281	0.31706	0.24296	0.748	0.26414	0.39121	0.31612	0.39411	0.748	0.22936	0.25962	0.48212	0.59339
0.752	0.30611	0.31892	0.24633	0.752	0.26732	0.39336	0.31764	0.396	0.752	0.23215	0.26184	0.48104	0.59298
0.756	0.30965	0.32048	0.2495	0.756	0.27049	0.39582	0.31938	0.39758	0.756	0.23575	0.26461	0.47996	0.59298
0.76	0.31421	0.32265	0.25268	0.76	0.27345	0.39766	0.32111	0.39916	0.76	0.23934	0.26766	0.47923	0.59298
0.764	0.31928	0.32513	0.25605	0.764	0.27663	0.40012	0.32306	0.40105	0.764	0.24293	0.27126	0.47851	0.5938
0.768	0.3246	0.32793	0.25962	0.768	0.2798	0.40227	0.32502	0.40325	0.768	0.24693	0.27459	0.47815	0.59462
0.772	0.33068	0.3301	0.26279	0.772	0.28213	0.40411	0.32697	0.40483	0.772	0.25092	0.27819	0.47815	0.59543
0.776	0.33778	0.33383	0.26676	0.776	0.28531	0.40656	0.32979	0.40735	0.776	0.25571	0.28263	0.47887	0.59748

0.78	0.34462	0.33663	0.27013	0.78	0.28785	0.40779	0.33153	0.40956	0.78	0.2601	0.28678	0.47923	0.59912
0.784	0.35146	0.34004	0.2735	0.784	0.28996	0.40902	0.33326	0.41176	0.784	0.26449	0.29094	0.4796	0.60075
0.788	0.3588	0.34315	0.27668	0.788	0.29187	0.40963	0.33521	0.41397	0.788	0.26849	0.2951	0.47996	0.60239
0.792	0.3664	0.34625	0.27965	0.792	0.29377	0.41025	0.33673	0.41586	0.792	0.27288	0.29898	0.48104	0.60403
0.796	0.374	0.34967	0.28243	0.796	0.29525	0.41086	0.33803	0.41807	0.796	0.27767	0.30314	0.48212	0.60607
0.8	0.3811	0.35309	0.2852	0.8	0.29652	0.41086	0.33955	0.42059	0.8	0.28206	0.30757	0.48357	0.60852
0.804	0.38794	0.35588	0.28758	0.804	0.29737	0.41055	0.34042	0.42248	0.804	0.28606	0.31118	0.48501	0.61057
0.808	0.39452	0.3593	0.28996	0.808	0.29822	0.41055	0.34129	0.42437	0.808	0.29085	0.31506	0.48646	0.61302
0.812	0.40086	0.3624	0.29195	0.812	0.29864	0.40994	0.34216	0.42627	0.812	0.29484	0.31894	0.48826	0.61548
0.816	0.40668	0.36551	0.29353	0.816	0.29885	0.40902	0.34259	0.42816	0.816	0.29883	0.32254	0.49007	0.61793
0.82	0.412	0.3683	0.29512	0.82	0.29885	0.4081	0.34281	0.42942	0.82	0.30283	0.32587	0.49187	0.61998
0.824	0.41631	0.37079	0.29611	0.824	0.29822	0.40687	0.34259	0.43068	0.824	0.30602	0.32864	0.49332	0.62161
0.828	0.42138	0.37358	0.2971	0.828	0.29822	0.40564	0.34259	0.43194	0.828	0.31001	0.33141	0.49549	0.62448
0.832	0.42518	0.37638	0.29829	0.832	0.29758	0.40472	0.34259	0.43289	0.832	0.31361	0.33391	0.49729	0.62693
0.836	0.42847	0.37855	0.29869	0.836	0.29716	0.40288	0.34216	0.43352	0.836	0.3168	0.33585	0.4991	0.62898
0.84	0.43202	0.38104	0.29968	0.84	0.29673	0.40165	0.34194	0.43415	0.84	0.32	0.33806	0.5009	0.63143
0.844	0.43531	0.38352	0.30028	0.844	0.29631	0.40012	0.34172	0.43446	0.844	0.32319	0.33973	0.50307	0.63389
0.848	0.43835	0.3857	0.30087	0.848	0.29589	0.39889	0.34151	0.43478	0.848	0.32639	0.34084	0.50451	0.63552
0.852	0.44114	0.38787	0.30167	0.852	0.29568	0.39735	0.34129	0.43478	0.852	0.32878	0.34222	0.50596	0.63716
0.856	0.44418	0.39035	0.30226	0.856	0.29525	0.39613	0.34085	0.43478	0.856	0.33118	0.34333	0.50776	0.63839
0.86	0.44722	0.39284	0.30345	0.86	0.29525	0.3949	0.34085	0.43509	0.86	0.33397	0.34416	0.50885	0.64043
0.864	0.45051	0.39563	0.30444	0.864	0.29546	0.39367	0.34107	0.43509	0.864	0.33597	0.34499	0.51065	0.64166
0.868	0.45355	0.39812	0.30563	0.868	0.29589	0.39275	0.34107	0.43509	0.868	0.33836	0.34555	0.51138	0.64248
0.872	0.45735	0.40122	0.30722	0.872	0.29652	0.39213	0.34151	0.43541	0.872	0.34036	0.34638	0.51282	0.6433
0.876	0.46191	0.40433	0.3094	0.876	0.29779	0.39183	0.34259	0.43635	0.876	0.34316	0.34749	0.5139	0.64493
0.88	0.46622	0.40744	0.31138	0.88	0.29885	0.39183	0.34324	0.43667	0.88	0.34515	0.34832	0.51499	0.64534
0.884	0.47078	0.41116	0.31396	0.884	0.30033	0.39152	0.34411	0.43761	0.884	0.34715	0.34943	0.51571	0.64575
0.888	0.47559	0.41489	0.31654	0.888	0.30203	0.39183	0.34563	0.43856	0.888	0.34954	0.35026	0.51679	0.64575
0.892	0.48142	0.41893	0.31952	0.892	0.30414	0.39275	0.34715	0.44014	0.892	0.35194	0.35165	0.51752	0.64657
0.896	0.48724	0.42327	0.32289	0.896	0.30647	0.39367	0.34888	0.44171	0.896	0.35434	0.35303	0.5186	0.64698
0.9	0.49332	0.42793	0.32646	0.9	0.30901	0.39459	0.35084	0.44297	0.9	0.35673	0.35442	0.51968	0.64657
0.904	0.49991	0.43259	0.33042	0.904	0.31197	0.39613	0.35344	0.44518	0.904	0.35953	0.35608	0.52077	0.64657
0.908	0.50675	0.43818	0.33479	0.908	0.31515	0.39797	0.35604	0.44739	0.908	0.36192	0.3583	0.52185	0.64657
0.912	0.51384	0.44346	0.33895	0.912	0.31874	0.40012	0.35908	0.44991	0.912	0.36472	0.36024	0.52329	0.64616
0.916	0.52119	0.44905	0.34371	0.916	0.32255	0.40227	0.3619	0.45275	0.916	0.36791	0.36273	0.52438	0.64575
0.92	0.52879	0.45526	0.34847	0.92	0.32658	0.40472	0.36537	0.45558	0.92	0.37071	0.36523	0.52582	0.64493
0.924	0.53665	0.46116	0.35363	0.924	0.33081	0.40779	0.36906	0.45905	0.924	0.3739	0.368	0.52763	0.64411
0.928	0.5445	0.46769	0.35879	0.928	0.33504	0.41117	0.37297	0.46283	0.928	0.3771	0.37105	0.52943	0.6433
0.932	0.55235	0.4739	0.36394	0.932	0.33948	0.41455	0.37665	0.4663	0.932	0.38029	0.37382	0.53124	0.64166
0.936	0.56046	0.48042	0.3695	0.936	0.34435	0.41823	0.38121	0.4704	0.936	0.38349	0.37743	0.53377	0.64084
0.94	0.56907	0.48694	0.37545	0.94	0.34922	0.42222	0.38577	0.47481	0.94	0.38708	0.38103	0.53629	0.63961
0.944	0.57718	0.49346	0.3812	0.944	0.3543	0.42621	0.39032	0.47923	0.944	0.39107	0.38463	0.53846	0.63839
0.948	0.58529	0.49998	0.38695	0.948	0.35959	0.43082	0.39488	0.48395	0.948	0.39467	0.38824	0.54099	0.63675
0.952	0.59339	0.50651	0.3931	0.952	0.36488	0.43511	0.40009	0.48868	0.952	0.39866	0.39239	0.54388	0.63511
0.956	0.6015	0.51303	0.39905	0.956	0.37038	0.43972	0.40508	0.49373	0.956	0.40265	0.39683	0.54713	0.63389
0.96	0.61011	0.51955	0.4052	0.96	0.37589	0.44463	0.41028	0.49909	0.96	0.40664	0.40126	0.55038	0.63225
0.964	0.61797	0.52545	0.41134	0.964	0.38139	0.44954	0.41527	0.50444	0.964	0.41064	0.4057	0.55327	0.63102
0.968	0.62607	0.53135	0.41769	0.968	0.3871	0.45476	0.4207	0.5098	0.968	0.41503	0.41041	0.55688	0.6298
0.972	0.63418	0.53725	0.42404	0.972	0.39282	0.45998	0.42612	0.51548	0.972	0.41942	0.4154	0.56085	0.62857
0.976	0.64153	0.54284	0.43019	0.976	0.39853	0.4652	0.43155	0.52147	0.976	0.42381	0.42011	0.56446	0.62734
0.98	0.64913	0.54812	0.43653	0.98	0.40446	0.47011	0.43697	0.52714	0.98	0.42821	0.42538	0.56844	0.62652
0.984	0.65622	0.5534	0.44248	0.984	0.40975	0.47564	0.44218	0.53313	0.984	0.433	0.43037	0.57205	0.62571
0.988	0.66357	0.55806	0.44863	0.988	0.41525	0.48055	0.44739	0.53944	0.988	0.43699	0.43536	0.57602	0.6253
0.992	0.67066	0.56241	0.45458	0.992	0.42075	0.48546	0.45238	0.54543	0.992	0.44138	0.44063	0.57999	0.62489
0.996	0.67674	0.56645	0.45994	0.996	0.42562	0.49007	0.45693	0.5511	0.996	0.44538	0.44561	0.58397	0.62407
1	0.68333	0.57017	0.46549	1	0.4307	0.49467	0.46149	0.5574	1	0.44977	0.4506	0.58794	0.62366
1.004	0.68941	0.5739	0.47104	1.004	0.43536	0.49928	0.46604	0.56339	1.004	0.45416	0.45559	0.59191	0.62325
1.008	0.69523	0.57701	0.4762	1.008	0.4398	0.50327	0.47017	0.56907	1.008	0.45815	0.46058	0.59588	0.62325
1.012	0.7003	0.5798	0.48076	1.012	0.44382	0.50726	0.47407	0.57474	1.012	0.46215	0.4653	0.59986	0.62325
1.016	0.70511	0.58229	0.48592	1.016	0.44763	0.51125	0.47776	0.58042	1.016	0.46574	0.46945	0.60347	0.62284
1.02	0.70967	0.58477	0.49008	1.02	0.45144	0.51494	0.48145	0.58609	1.02	0.46973	0.47417	0.60744	0.62325
1.024	0.71373	0.58694	0.49425	1.024	0.45483	0.51831	0.48492	0.59177	1.024	0.47333	0.4786	0.61105	0.62325
1.028	0.71702	0.5885	0.49782	1.028	0.45779	0.52138	0.48774	0.59649	1.028	0.47692	0.4822	0.61394	0.62284
1.032	0.72006	0.59036	0.50159	1.032	0.46075	0.52476	0.49078	0.60185	1.032	0.48011	0.48608	0.61755	0.62284
1.036	0.7231	0.59191	0.50496	1.036	0.46372	0.52752	0.4936	0.6069	1.036	0.48371	0.48997	0.6208	0.62284
1.04	0.72513	0.59347	0.50813	1.04	0.46647	0.53059	0.49642	0.61163	1.04	0.4869	0.49329	0.62333	0.62284
1.044	0.7269	0.59471	0.51111	1.044	0.46901	0.53336	0.49902	0.61667	1.044	0.4901	0.49662	0.62622	0.62243
1.048	0.72817	0.59626	0.51388	1.048	0.47133	0.53612	0.50141	0.62108	1.048	0.49329	0.49994	0.62875	0.62243
1.052	0.72944	0.5975	0.51666	1.052	0.47387	0.53888	0.50423	0.62581	1.052	0.49649	0.50299	0.63127	0.62243
1.056	0.73045	0.59906	0.51944	1.056	0.47641	0.54164	0.50705	0.63023	1.056	0.49968	0.50604	0.6338	0.62243
1.06	0.7307	0.6003	0.52182	1.06	0.47874	0.5441	0.50966	0.63432	1.06	0.50287	0.50854	0.63561	0.62202
1.064	0.73146	0.60185	0.52459	1.064	0.48149	0.54686	0.51248	0.63874	1.064	0.50607	0.51159	0.63741	0.62202
1.068	0.73197	0.60371	0.52737	1.068	0.48446	0.54993	0.51551	0.64347	1.068	0.50966	0.51436	0.63922	0.62202
1.072	0.73248	0.60527	0.52995	1.072	0.48721	0.553	0.51855	0.64756	1.072	0.51286	0.51741	0.64103	0.62202
1.076	0.73273	0.60713	0.53273	1.076	0.49017	0.55577	0.52181	0.65229	1.076	0.51645	0.52018	0.64247	0.62202
1.08	0.73324	0.60899	0.5357	1.08	0.49334	0.55884	0.52528	0.65671	1.08	0.51965	0.52323	0.64391	0.62202
1.084	0.7345	0.61148	0.53927	1.084	0.49694	0.56283	0.52897	0.66143	1.084	0.52404	0.52656	0.64608	0.62284
1.088	0.73526	0.61427	0.54264	1.088	0.50054	0.56621	0.53287	0.66648	1.088	0.52803	0.5296	0.64789	0.62366
1.092	0.73602	0.61676	0.54601	1.092	0.50414	0.56989	0.53656	0.67121	1.092	0.53202	0.53265	0.64897	0.62407
1.096	0.73729	0.61986	0.54978	1.096	0.50816	0.57388	0.5409	0.67657	1.096	0.53642	0.53626	0.65114	0.62489
1.1	0.73881	0.62297	0.55375	1.1	0.51239	0.57787	0.54502	0.68161	1.1	0.54121	0.53986	0.65294	0.62611
1.104	0.74058	0.62608	0.55772	1.104	0.51684	0.58186	0.54936	0.68697	1.104	0.546	0.54402	0.65475	0.62693
1.108	0.7421	0.62949	0.56208	1.108	0.52149	0.58616	0.55413	0.69233	1.108	0.55079	0.5479	0.65655	0.62816
1.112	0.74438	0.63322	0.56664	1.112	0.52636	0.59077	0.55891	0.698	1.112	0.55598	0.55206	0.65872	0.6298
1.116	0.74666	0.63726	0.5714	1.116	0.53144	0.59537	0.5639	0.70399	1.116	0.56157	0.55677	0.66125	0.63184
1.12	0.7492	0.64129	0.57656	1.12	0.53715	0.60028	0.56954	0.70998	1.12	0.56716	0.5612	0.66378	0.63389
1.124	0.75148	0.64564	0.58171	1.124	0.54244	0.60519	0.57475	0.71597	1.124	0.57315	0.56564	0.66594	0.63593
1.128	0.75426	0.64999	0.58727	1.128	0.54837	0.61072	0.5806	0.72227	1.128	0.57914	0.57063	0.66883	0.63839
1.132	0.75705	0.65496	0.59282	1.132	0.55451	0.61594	0.58646	0.72858	1.132	0.58553	0.5759	0.67208	0.64125
1.136	0.76034	0.65993	0.59857	1.136	0.56064	0.62208	0.59254	0.73488	1.136	0.59232	0.58116	0.67497	0.64411
1.14	0.76313	0.6649	0.60452	1.14	0.5672	0.62791	0.59861	0.7415	1.14	0.59871	0.58671	0.67858	0.64739
1.144	0.76642	0.67018	0.61107	1.144	0.57355	0.63405	0.60512	0.74812	1.144	0.60549	0.59253	0.6822	0.65066
1.148	0.76972	0.67577	0.61781	1.148	0.58054	0.6405	0.61185	0.75506	1.148	0.61308	0.59863	0.68617	0.65475
1.152	0.77326	0.68198	0.62436	1.152	0.58773	0.64725	0.61901	0.76199	1.152	0.62027	0.60472	0.69014	0.65884
1.156	0.77681	0.68757	0.6313	1.156	0.59493	0.65339	0.62573	0.7683	1.156	0.62746	0.61082	0.69411	0.66293
1.16	0.78061	0.69378	0.63844	1.16	0.60234	0.66045	0.63311	0.77523	1.16	0.63544	0.61775	0.69881	0.66743
1.164	0.78441	0.69999	0.64597	1.164	0.61038	0.66721	0.6407	0.78217	1.164	0.64303	0.6244	0.7035	0.67275
1.168	0.78821	0.7062	0.65351	1.168	0.61821	0.67427	0.6483	0.78942	1.168	0.65101	0.63106	0.7082	0.67766
1.172	0.79226	0.71272	0.66125	1.172	0.62625	0.68164	0.65589	0.79604	1.172	0.659	0.63799	0.71289	0.68297
1.176	0.79632	0.71894	0.66898	1.176	0.63451	0.6887	0.66414	0.80298	1.176	0.66739	0.64547	0.71795	0.68829
1.18	0.80062	0.72577	0.67731	1.18	0.64318	0.69637	0.67238	0.81023	1.18	0.67577	0.65323	0.72337	0.69443
1.184	0.80544	0.73353	0.68643	1.184	0.65271	0.70497	0.68149	0.81779	1.184	0.68575	0.66183	0.73023	0.70138
1.188	0.80949	0.74036	0.69476	1.188	0.66138	0.71234	0.68974	0.8241	1.188	0.69414	0.66959	0.73564	0.70752

1.192	0.8143	0.7472	0.70309	1.192	0.67048	0.72063	0.69863	0.83103	1.192	0.70292	0.67818	0.74178	0.71406
1.196	0.81912	0.75465	0.71222	1.196	0.68001	0.72892	0.70731	0.83765	1.196	0.71211	0.68677	0.74828	0.72102
1.2	0.82368	0.7621	0.72134	1.2	0.68974	0.7369	0.71621	0.84459	1.2	0.72129	0.69537	0.75479	0.72838
1.204	0.82849	0.76987	0.73027	1.204	0.69927	0.74519	0.7251	0.85121	1.204	0.73047	0.70396	0.76129	0.73574
1.208	0.8333	0.77732	0.73959	1.208	0.70921	0.75409	0.73422	0.85783	1.208	0.73966	0.71311	0.76851	0.74311
1.212	0.83862	0.7854	0.74911	1.212	0.71979	0.76238	0.74376	0.86413	1.212	0.74964	0.72253	0.77573	0.75129
1.216	0.84394	0.79347	0.75902	1.216	0.73016	0.77128	0.75331	0.87075	1.216	0.75922	0.73251	0.78295	0.75988
1.22	0.84901	0.80093	0.76835	1.22	0.74032	0.77988	0.76286	0.87674	1.22	0.76881	0.74193	0.79018	0.76806
1.224	0.85484	0.80931	0.77846	1.224	0.75112	0.78909	0.77305	0.88273	1.224	0.77919	0.75219	0.79812	0.77706
1.228	0.86066	0.81739	0.78838	1.228	0.76212	0.7983	0.78325	0.88904	1.228	0.78917	0.76272	0.80607	0.78606
1.232	0.86649	0.82577	0.79849	1.232	0.77313	0.80782	0.79323	0.89534	1.232	0.79955	0.77326	0.81473	0.79547
1.236	0.87232	0.83385	0.80821	1.236	0.78413	0.81703	0.80321	0.9007	1.236	0.80954	0.78379	0.82268	0.80447
1.24	0.87865	0.84254	0.81872	1.24	0.79514	0.82685	0.81363	0.90669	1.24	0.81952	0.79515	0.83135	0.81388
1.244	0.88524	0.85124	0.82943	1.244	0.80699	0.83698	0.82447	0.91268	1.244	0.8303	0.80652	0.84038	0.82451
1.248	0.89208	0.86055	0.84034	1.248	0.81926	0.84711	0.83554	0.91867	1.248	0.84108	0.81844	0.85013	0.83556
1.252	0.89841	0.86987	0.85085	1.252	0.83111	0.85724	0.84617	0.92434	1.252	0.85186	0.8298	0.85952	0.84578
1.256	0.90576	0.87919	0.86236	1.256	0.8436	0.86799	0.85767	0.93065	1.256	0.86304	0.842	0.86999	0.85683
1.26	0.91311	0.88882	0.87346	1.26	0.85651	0.87904	0.86939	0.93664	1.26	0.87462	0.85475	0.8801	0.86869
1.264	0.92045	0.89907	0.88516	1.264	0.86963	0.88979	0.88132	0.94263	1.264	0.8862	0.8675	0.89094	0.88014
1.268	0.92805	0.90931	0.89667	1.268	0.88254	0.90115	0.89325	0.9483	1.268	0.89778	0.88053	0.90141	0.89201
1.272	0.93616	0.91956	0.90877	1.272	0.89609	0.9122	0.90562	0.95398	1.272	0.90976	0.89439	0.9126	0.90428
1.276	0.94427	0.93012	0.92106	1.276	0.91005	0.92417	0.91864	0.96028	1.276	0.92214	0.90853	0.92416	0.91655
1.28	0.95313	0.9413	0.93356	1.28	0.92402	0.93614	0.93166	0.96658	1.28	0.93491	0.92294	0.93608	0.93046
1.284	0.96149	0.95217	0.94605	1.284	0.9382	0.94781	0.94446	0.97226	1.284	0.94689	0.93708	0.94763	0.94273
1.288	0.97137	0.96428	0.95954	1.288	0.95365	0.96101	0.95834	0.97951	1.288	0.96007	0.95315	0.96064	0.95705
1.292	0.98049	0.97578	0.97243	1.292	0.96889	0.9736	0.97223	0.98613	1.292	0.97325	0.9684	0.97364	0.97137
1.296	0.99037	0.98789	0.98631	1.296	0.98392	0.98649	0.9859	0.99306	1.296	0.98642	0.9842	0.98664	0.98527
1.3	1	1	1	1.3	1	1	1	1	1.3	1	1	1	1

**Ethanol-PBS (Spring)**				**Ethanol-PBS (Summer)**					**Ethanol-PBS (Autumn)**				
0.004	0.10601	0.12211	0.05926	0.004	0.07563	0.05468	0.05885	0.01102	0.004	0.04043	0.03146	0.0344	0.02708
0.008	0.09781	0.11253	0.05342	0.008	0.0687	0.04977	0.05368	0.00912	0.008	0.03571	0.02729	0.02995	0.02391
0.012	0.09126	0.10348	0.04836	0.012	0.06225	0.04503	0.04851	0.00738	0.012	0.03164	0.02365	0.02572	0.02091
0.016	0.0847	0.09604	0.04369	0.016	0.05606	0.04058	0.04414	0.00568	0.016	0.02775	0.0203	0.02226	0.01821
0.02	0.07923	0.08859	0.03941	0.02	0.05075	0.03657	0.03976	0.00432	0.02	0.02444	0.01721	0.01907	0.01574
0.024	0.07377	0.08183	0.03512	0.024	0.0457	0.03255	0.03539	0.0032	0.024	0.02117	0.01446	0.01643	0.01344
0.028	0.06885	0.07576	0.03162	0.028	0.04139	0.02923	0.03141	0.00204	0.028	0.01843	0.01184	0.01385	0.01137
0.032	0.06393	0.06949	0.02772	0.032	0.03683	0.02568	0.02823	0.00121	0.032	0.01573	0.0097	0.01138	0.0094
0.036	0.05902	0.06363	0.02422	0.036	0.03276	0.02243	0.02425	4.37E-04	0.036	0.01295	0.00725	0.0094	0.0076
0.04	0.05464	0.05863	0.02071	0.04	0.0287	0.01951	0.02107	9.71E-05	0.04	0.01074	0.00541	0.00742	0.00592
0.044	0.05027	0.05347	0.01787	0.044	0.02498	0.01686	0.01789	0	0.044	0.00857	0.00403	0.00555	0.00449
0.048	0.04645	0.0489	0.01507	0.048	0.02141	0.01437	0.01511	9.71E-05	0.048	0.00672	0.00257	0.0039	0.00314
0.052	0.04208	0.04411	0.01223	0.052	0.01789	0.01191	0.01233	2.43E-04	0.052	0.00482	0.00154	0.00275	0.00205
0.056	0.03825	0.03943	0.01016	0.056	0.01477	0.00969	0.00994	0.00102	0.056	0.00314	6.44E-04	0.00159	0.00115
0.06	0.03497	0.03538	0.0081	0.06	0.01249	0.00786	0.00795	0.00204	0.06	0.0019	2.15E-04	9.34E-04	4.77E-04
0.064	0.03115	0.0316	0.00615	0.064	0.00966	0.00614	0.00596	0.00345	0.064	0.00102	0	3.30E-04	1.68E-04
0.068	0.02787	0.02729	0.00432	0.068	0.00739	0.00468	0.00398	0.00548	0.068	3.54E-04	4.72E-04	0	0
0.072	0.02459	0.024	0.00276	0.072	0.00515	0.00325	0.00278	0.00781	0.072	0	0.00112	1.10E-04	3.93E-04
0.076	0.02186	0.02059	0.00199	0.076	0.00357	0.00212	0.00159	0.01082	0.076	2.65E-04	0.00215	4.95E-04	0.00115
0.08	0.01858	0.0174	8.18E-04	0.08	0.00193	0.00149	7.95E-04	0.01403	0.08	5.74E-04	0.00382	0.00159	0.00236
0.084	0.01585	0.01453	2.34E-04	0.084	8.92E-04	8.30E-04	3.98E-04	0.01815	0.084	0.00141	0.00588	0.00302	0.0041
0.088	0.01311	0.01149	0	0.088	1.98E-04	2.32E-04	0	0.02266	0.088	0.00247	0.0082	0.00478	0.00631
0.092	0.01093	0.0092	3.89E-05	0.092	0	0	3.98E-04	0.02795	0.092	0.00424	0.01124	0.00736	0.00923
0.096	0.0082	0.0074	3.12E-04	0.096	1.49E-04	1.66E-04	7.95E-04	0.03383	0.096	0.0065	0.01519	0.01006	0.01291
0.1	0.00601	0.00537	7.01E-04	0.1	4.46E-04	3.32E-04	0.00119	0.04009	0.1	0.00897	0.01948	0.01352	0.01723
0.104	0.00437	0.00404	0.00156	0.104	0.00124	5.97E-04	0.00278	0.04736	0.104	0.01211	0.02437	0.01797	0.02256
0.108	0.00273	0.0025	0.00245	0.108	0.00228	9.95E-04	0.00398	0.05508	0.108	0.01586	0.03008	0.0227	0.02876
0.112	0.00164	0.00112	0.00362	0.112	0.00387	0.00189	0.00557	0.06333	0.112	0.01993	0.036	0.02819	0.03603
0.116	5.46E-04	5.85E-04	0.00487	0.116	0.00565	0.00265	0.00755	0.07211	0.116	0.02461	0.04257	0.03451	0.04439
0.12	0	1.60E-04	0.0065	0.12	0.00793	0.00348	0.00994	0.08134	0.12	0.02978	0.04952	0.04144	0.05413
0.124	0	0	0.0081	0.124	0.0108	0.00461	0.01233	0.09099	0.124	0.03544	0.05733	0.04935	0.06519
0.128	5.46E-04	3.72E-04	0.01016	0.128	0.01383	0.00594	0.01551	0.10084	0.128	0.04158	0.06518	0.05776	0.07787
0.132	0.00219	0.00101	0.01254	0.132	0.0169	0.00717	0.01869	0.11089	0.132	0.04812	0.0742	0.06732	0.09218
0.136	0.00437	0.00213	0.01491	0.136	0.02121	0.00889	0.02266	0.12123	0.136	0.05524	0.08338	0.0776	0.10846
0.14	0.0071	0.00356	0.01783	0.14	0.02587	0.01042	0.02704	0.13181	0.14	0.06266	0.09342	0.08931	0.12642
0.144	0.01148	0.00617	0.02149	0.144	0.03108	0.01224	0.03141	0.14253	0.144	0.07062	0.10424	0.10173	0.14634
0.148	0.01694	0.00899	0.02539	0.148	0.03688	0.01427	0.03698	0.15326	0.148	0.07897	0.11539	0.11514	0.16795
0.152	0.02404	0.01272	0.03006	0.152	0.04426	0.01679	0.04254	0.16427	0.152	0.08798	0.12745	0.1297	0.19124
0.156	0.03333	0.01761	0.03512	0.156	0.05229	0.01958	0.0497	0.17573	0.156	0.09757	0.14037	0.14531	0.21594
0.16	0.04426	0.02405	0.04135	0.16	0.06225	0.02289	0.05726	0.18762	0.16	0.10778	0.15457	0.16201	0.24176
0.164	0.05738	0.03027	0.04836	0.164	0.07316	0.02648	0.06561	0.19946	0.164	0.11843	0.16946	0.17911	0.26785
0.168	0.07268	0.03836	0.05615	0.168	0.08604	0.03066	0.07475	0.21203	0.168	0.12988	0.18508	0.19719	0.29423
0.172	0.09016	0.04794	0.0651	0.172	0.09992	0.0356	0.08469	0.22518	0.172	0.14194	0.20182	0.2156	0.32005
0.176	0.11093	0.05853	0.07484	0.176	0.11578	0.04085	0.09583	0.23833	0.176	0.15454	0.21898	0.23395	0.34503
0.18	0.13333	0.06949	0.08535	0.18	0.13313	0.04675	0.10736	0.25187	0.18	0.16744	0.23658	0.25181	0.36832
0.184	0.15738	0.08125	0.09664	0.184	0.15147	0.05335	0.11889	0.26531	0.184	0.1807	0.25332	0.26896	0.38993
0.188	0.18306	0.09444	0.10832	0.188	0.1708	0.05999	0.13121	0.27856	0.188	0.19351	0.27005	0.2849	0.40929
0.192	0.20929	0.10561	0.11962	0.192	0.19013	0.06663	0.14314	0.29084	0.192	0.20633	0.28507	0.29875	0.42585
0.196	0.23607	0.11785	0.13052	0.196	0.20847	0.07359	0.15467	0.30224	0.196	0.21826	0.29837	0.31029	0.4396
0.2	0.2612	0.12849	0.14064	0.2	0.22581	0.08023	0.16541	0.31248	0.2	0.22975	0.30996	0.31908	0.45026
0.204	0.28579	0.13807	0.1496	0.204	0.24118	0.0862	0.17535	0.32102	0.204	0.23947	0.31854	0.32568	0.45784
0.208	0.30765	0.14605	0.157	0.208	0.25357	0.09184	0.1833	0.32806	0.208	0.24831	0.32369	0.32952	0.46233
0.212	0.32568	0.15084	0.16284	0.212	0.26249	0.09616	0.18966	0.33272	0.212	0.25494	0.32627	0.33062	0.46373
0.216	0.33934	0.1535	0.16595	0.216	0.26745	0.09948	0.19404	0.33563	0.216	0.2598	0.32498	0.32897	0.46148
0.22	0.34918	0.15403	0.16751	0.22	0.26943	0.1018	0.19642	0.3366	0.22	0.26289	0.32112	0.32623	0.45643
0.224	0.3541	0.1519	0.16634	0.224	0.26646	0.10279	0.19682	0.33563	0.224	0.26422	0.31468	0.32128	0.44801
0.228	0.35301	0.14765	0.16284	0.228	0.25952	0.10213	0.19523	0.33262	0.228	0.26289	0.30567	0.31413	0.43763
0.232	0.34809	0.14073	0.15778	0.232	0.2496	0.10047	0.19165	0.32806	0.232	0.2598	0.29408	0.30699	0.42472
0.236	0.3377	0.13168	0.15116	0.236	0.23622	0.09782	0.18728	0.32204	0.236	0.25538	0.28164	0.29875	0.41041
0.24	0.32404	0.12157	0.14259	0.24	0.22036	0.09384	0.18091	0.31501	0.24	0.24919	0.26748	0.28907	0.39413
0.244	0.3071	0.10987	0.13325	0.244	0.20301	0.08985	0.17376	0.3071	0.244	0.24212	0.25289	0.27968	0.3773
0.248	0.28798	0.0987	0.12312	0.248	0.18418	0.08454	0.16541	0.29884	0.248	0.23328	0.23787	0.26989	0.36046
0.252	0.26831	0.0871	0.11261	0.252	0.16584	0.07957	0.15746	0.29055	0.252	0.22489	0.22285	0.26	0.3439
0.256	0.24754	0.07566	0.10248	0.256	0.14701	0.07426	0.14871	0.28196	0.256	0.21605	0.20869	0.25071	0.32763
0.26	0.22732	0.06475	0.09275	0.26	0.12916	0.06928	0.14036	0.27337	0.26	0.20677	0.19538	0.24126	0.31219
0.264	0.2082	0.05528	0.08302	0.264	0.11281	0.0643	0.1328	0.26609	0.264	0.19793	0.18294	0.23269	0.29788
0.268	0.19071	0.04634	0.07445	0.268	0.09744	0.05966	0.12485	0.2591	0.268	0.18909	0.17144	0.22455	0.28441
0.272	0.17377	0.03863	0.06627	0.272	0.08356	0.05535	0.11769	0.25274	0.272	0.18114	0.16058	0.21703	0.27234
0.276	0.15902	0.03155	0.05887	0.276	0.07117	0.05156	0.11173	0.24721	0.276	0.17407	0.15097	0.20983	0.26112
0.28	0.14536	0.02602	0.05187	0.28	0.06027	0.04798	0.10577	0.2427	0.28	0.167	0.14226	0.20301	0.2513
0.284	0.13333	0.0208	0.04602	0.284	0.0507	0.04529	0.10099	0.23915	0.284	0.16125	0.13462	0.19697	0.24232
0.288	0.1235	0.01676	0.04096	0.288	0.04218	0.0428	0.09662	0.23634	0.288	0.15617	0.12775	0.19147	0.23446
0.292	0.11475	0.01335	0.03629	0.292	0.03494	0.04088	0.09304	0.23445	0.292	0.15144	0.12183	0.18625	0.22716

0.296	0.1071	0.01075	0.0324	0.296	0.02855	0.03935	0.09026	0.23318	0.296	0.14747	0.11689	0.18202	0.22099
0.3	0.10109	0.00862	0.02928	0.3	0.02349	0.03852	0.08827	0.23265	0.3	0.14424	0.11278	0.17812	0.21566
0.304	0.09563	0.0074	0.02617	0.304	0.01938	0.03826	0.08708	0.2327	0.304	0.14168	0.10956	0.17482	0.21061
0.308	0.09126	0.00628	0.02422	0.308	0.01576	0.03803	0.08588	0.23328	0.308	0.13969	0.10651	0.17202	0.20668
0.312	0.08743	0.0058	0.02227	0.312	0.01269	0.03842	0.08549	0.2344	0.312	0.1377	0.10432	0.16943	0.20275
0.316	0.0847	0.00596	0.02071	0.316	0.01066	0.03945	0.08588	0.23648	0.316	0.13677	0.10282	0.16767	0.19994
0.32	0.08251	0.00638	0.02013	0.32	0.00902	0.04058	0.08708	0.23886	0.32	0.13589	0.10205	0.16641	0.19714
0.324	0.08033	0.00756	0.01935	0.324	0.00798	0.04231	0.08827	0.24163	0.324	0.13549	0.10179	0.16531	0.19489
0.328	0.07869	0.00873	0.01966	0.328	0.00773	0.04453	0.09026	0.24522	0.328	0.13593	0.10179	0.16504	0.19321
0.332	0.07814	0.01091	0.02013	0.332	0.00808	0.04728	0.09304	0.24959	0.332	0.13624	0.10239	0.16509	0.19181
0.336	0.0776	0.01335	0.0211	0.336	0.00862	0.05024	0.09622	0.2542	0.336	0.13708	0.10368	0.16548	0.19124
0.34	0.07814	0.01601	0.02266	0.34	0.00971	0.05402	0.0998	0.25939	0.34	0.1381	0.10565	0.16658	0.1904
0.344	0.07814	0.01921	0.02383	0.344	0.0111	0.058	0.10417	0.26512	0.344	0.13956	0.10775	0.16795	0.19012
0.348	0.07869	0.02341	0.02656	0.348	0.01328	0.06298	0.10895	0.27157	0.348	0.14146	0.11089	0.16982	0.1904
0.352	0.07978	0.02761	0.02889	0.352	0.01561	0.06829	0.11451	0.27856	0.352	0.1434	0.11415	0.17174	0.19096
0.356	0.08087	0.03246	0.03162	0.356	0.01809	0.07393	0.12048	0.28594	0.356	0.14574	0.1178	0.17421	0.19181
0.36	0.08251	0.03783	0.03512	0.36	0.02126	0.08056	0.12724	0.2937	0.36	0.14835	0.12209	0.17735	0.19293
0.364	0.08525	0.04427	0.03941	0.364	0.02528	0.08786	0.13519	0.30278	0.364	0.15184	0.12728	0.18108	0.19461
0.368	0.08798	0.05129	0.04408	0.368	0.02914	0.09549	0.14314	0.31209	0.368	0.15493	0.13239	0.18493	0.1963
0.372	0.09126	0.05831	0.04875	0.372	0.03301	0.10346	0.15149	0.3218	0.372	0.15856	0.13814	0.18894	0.19854
0.376	0.09454	0.06619	0.05381	0.376	0.03752	0.11208	0.16064	0.33218	0.376	0.1624	0.14423	0.19389	0.20107
0.38	0.09836	0.0747	0.05965	0.38	0.04228	0.12171	0.17018	0.3434	0.38	0.167	0.15097	0.199	0.20359
0.384	0.10328	0.08406	0.06588	0.384	0.04753	0.13166	0.18091	0.35504	0.384	0.17142	0.15809	0.20483	0.20668
0.388	0.10765	0.09284	0.07211	0.388	0.05284	0.14162	0.19125	0.36669	0.388	0.17672	0.16539	0.21065	0.20977
0.392	0.11311	0.10295	0.07873	0.392	0.05878	0.15256	0.20278	0.37882	0.392	0.18202	0.1735	0.21736	0.21341
0.396	0.11967	0.11359	0.08613	0.396	0.06423	0.16385	0.21471	0.39144	0.396	0.18777	0.18165	0.22444	0.21734
0.4	0.12623	0.1237	0.09353	0.4	0.07018	0.17513	0.22664	0.40454	0.4	0.19395	0.19023	0.23214	0.22127
0.404	0.13388	0.13434	0.10093	0.404	0.07663	0.18674	0.23897	0.41716	0.404	0.20058	0.19924	0.24	0.22548
0.408	0.14098	0.14552	0.10871	0.408	0.08307	0.19869	0.25129	0.43026	0.408	0.20721	0.20783	0.24824	0.22969
0.412	0.15027	0.15669	0.1165	0.412	0.08951	0.21063	0.26362	0.44337	0.412	0.21472	0.21684	0.25698	0.23418
0.416	0.15847	0.16786	0.12429	0.416	0.09596	0.22258	0.27515	0.45598	0.416	0.2218	0.22585	0.26566	0.23867
0.42	0.16776	0.1785	0.13247	0.42	0.1024	0.23419	0.28668	0.46812	0.42	0.22931	0.23443	0.27473	0.24316
0.424	0.1776	0.18968	0.14064	0.424	0.10884	0.2458	0.29861	0.47976	0.424	0.23682	0.24345	0.28385	0.24793
0.428	0.18798	0.20138	0.14921	0.428	0.11578	0.25742	0.30974	0.49141	0.428	0.24477	0.25203	0.2927	0.2527
0.432	0.19836	0.21202	0.15739	0.432	0.12222	0.2687	0.32048	0.50257	0.432	0.25273	0.26018	0.30149	0.25747
0.436	0.20874	0.22267	0.16556	0.436	0.12867	0.27965	0.33121	0.51228	0.436	0.26068	0.26833	0.30974	0.26224
0.44	0.21967	0.23331	0.17374	0.44	0.13462	0.29026	0.34115	0.52247	0.44	0.2682	0.27563	0.31798	0.26729
0.444	0.23115	0.24448	0.18192	0.444	0.14155	0.30088	0.3507	0.5312	0.444	0.27571	0.28335	0.32623	0.27263
0.448	0.24262	0.25406	0.19048	0.448	0.148	0.31084	0.35984	0.53994	0.448	0.28278	0.29022	0.33337	0.27768
0.452	0.25355	0.2647	0.19827	0.452	0.15395	0.32013	0.36819	0.5477	0.452	0.28941	0.29666	0.34051	0.28301
0.456	0.26448	0.27534	0.20684	0.456	0.16039	0.32975	0.37654	0.55498	0.456	0.29604	0.30352	0.34656	0.28862
0.46	0.27705	0.28492	0.21463	0.46	0.16683	0.33838	0.38449	0.56226	0.46	0.30311	0.30996	0.35315	0.29423
0.464	0.28798	0.29556	0.22241	0.464	0.17328	0.347	0.39165	0.56809	0.464	0.30885	0.31554	0.35865	0.29985
0.468	0.3	0.30513	0.23059	0.468	0.17922	0.3553	0.39841	0.57391	0.468	0.3146	0.32155	0.36415	0.30574
0.472	0.31148	0.31471	0.23799	0.472	0.18567	0.3626	0.40477	0.57925	0.472	0.31946	0.3267	0.36909	0.31163
0.476	0.32295	0.32376	0.24539	0.476	0.19161	0.36957	0.41074	0.58459	0.476	0.32388	0.33185	0.37349	0.31724
0.48	0.33497	0.3328	0.25239	0.48	0.19806	0.37587	0.4163	0.58847	0.48	0.3283	0.33657	0.37789	0.32314
0.484	0.3459	0.34025	0.25901	0.484	0.20351	0.38151	0.42068	0.59187	0.484	0.33139	0.34086	0.38118	0.32875
0.488	0.35738	0.3477	0.26563	0.488	0.20896	0.38682	0.42505	0.59526	0.488	0.33404	0.34472	0.38448	0.33464
0.492	0.36776	0.35462	0.27147	0.492	0.21491	0.39147	0.42903	0.59769	0.492	0.33669	0.34858	0.38778	0.33997
0.496	0.3776	0.361	0.27731	0.496	0.21987	0.39578	0.43221	0.60012	0.496	0.3389	0.35159	0.39053	0.34531
0.5	0.38689	0.36685	0.28277	0.5	0.22433	0.3991	0.43459	0.60109	0.5	0.34023	0.35459	0.39217	0.35008
0.504	0.39508	0.37164	0.28783	0.504	0.22879	0.40242	0.43658	0.60206	0.504	0.34111	0.35674	0.39437	0.35513
0.508	0.40273	0.37643	0.2925	0.508	0.23275	0.4054	0.43857	0.60206	0.508	0.34199	0.35888	0.39547	0.35962
0.512	0.40929	0.38015	0.29678	0.512	0.23672	0.40772	0.44016	0.60206	0.512	0.34244	0.36017	0.39657	0.36383
0.516	0.41421	0.38281	0.30029	0.516	0.2392	0.40938	0.44056	0.6006	0.516	0.34244	0.3606	0.39657	0.3672
0.52	0.41858	0.38601	0.30418	0.52	0.24266	0.41137	0.44215	0.59963	0.52	0.34288	0.36146	0.39712	0.37056
0.524	0.4224	0.38867	0.30769	0.524	0.24564	0.41303	0.44254	0.59818	0.524	0.34244	0.36146	0.39712	0.37281
0.528	0.42459	0.39026	0.3108	0.528	0.24812	0.41469	0.44334	0.59575	0.528	0.34199	0.36146	0.39657	0.37477
0.532	0.42568	0.39186	0.31431	0.532	0.25109	0.41569	0.44334	0.59332	0.532	0.34111	0.36103	0.39547	0.37646
0.536	0.42678	0.39346	0.31703	0.536	0.25307	0.41702	0.44374	0.5909	0.536	0.34067	0.3606	0.39492	0.3773
0.54	0.42678	0.39505	0.32015	0.54	0.25506	0.41834	0.44374	0.58798	0.54	0.34023	0.3606	0.39327	0.37786
0.544	0.42678	0.39612	0.32248	0.544	0.25753	0.41967	0.44414	0.58556	0.544	0.33934	0.36017	0.39217	0.37814
0.548	0.42568	0.39718	0.32482	0.548	0.25952	0.42066	0.44374	0.58265	0.548	0.3389	0.35974	0.39053	0.37758
0.552	0.42459	0.39878	0.32793	0.552	0.26249	0.42199	0.44374	0.58022	0.552	0.33846	0.35931	0.38888	0.37702
0.556	0.4235	0.40037	0.33066	0.556	0.26447	0.42365	0.44374	0.57779	0.556	0.33802	0.35888	0.38723	0.37646
0.56	0.4224	0.40144	0.33339	0.56	0.26695	0.42531	0.44414	0.57585	0.56	0.33758	0.35931	0.38558	0.37533
0.564	0.42077	0.40303	0.33611	0.564	0.26992	0.42697	0.44414	0.57391	0.564	0.33713	0.35931	0.38393	0.37421
0.568	0.41913	0.40463	0.33845	0.568	0.27191	0.42863	0.44414	0.57197	0.568	0.33713	0.35974	0.38228	0.37281
0.572	0.41858	0.40676	0.34195	0.572	0.27538	0.43062	0.44453	0.571	0.572	0.33802	0.3606	0.38063	0.37197
0.576	0.41749	0.40889	0.34468	0.576	0.27835	0.43261	0.44533	0.57003	0.576	0.33846	0.36146	0.37953	0.37112
0.58	0.41585	0.41101	0.3474	0.58	0.28083	0.43427	0.44493	0.56906	0.58	0.3389	0.36231	0.37789	0.37
0.584	0.4153	0.41314	0.35052	0.584	0.2838	0.43626	0.44533	0.56857	0.584	0.33979	0.3636	0.37734	0.36944
0.588	0.4153	0.4158	0.35363	0.588	0.28678	0.43792	0.44573	0.56809	0.588	0.34067	0.36489	0.37624	0.36916
0.592	0.41475	0.41846	0.35636	0.592	0.28975	0.43958	0.44573	0.56809	0.592	0.34199	0.36661	0.37569	0.36916
0.596	0.41475	0.42059	0.35947	0.596	0.29223	0.44091	0.44573	0.56809	0.596	0.34332	0.36789	0.37514	0.36916
0.6	0.4153	0.42378	0.3622	0.6	0.2957	0.4419	0.44573	0.56809	0.6	0.34465	0.36961	0.37459	0.36944
0.604	0.41639	0.42591	0.36492	0.604	0.29818	0.44323	0.44533	0.56809	0.604	0.34597	0.37133	0.37459	0.37028
0.608	0.41749	0.42857	0.36765	0.608	0.30115	0.44356	0.44493	0.56809	0.608	0.34774	0.37304	0.37404	0.3714
0.612	0.41858	0.43017	0.36999	0.612	0.30313	0.44356	0.44374	0.56809	0.612	0.34951	0.37433	0.37404	0.37253
0.616	0.42077	0.4323	0.37232	0.616	0.30561	0.4429	0.44254	0.5676	0.616	0.35127	0.37605	0.37404	0.37421
0.62	0.4224	0.43389	0.37427	0.62	0.30759	0.44223	0.44095	0.56712	0.62	0.3526	0.37733	0.37404	0.37589
0.624	0.42404	0.43549	0.37583	0.624	0.30908	0.44057	0.43897	0.56663	0.624	0.35437	0.37819	0.37404	0.37786
0.628	0.42568	0.43602	0.377	0.628	0.31007	0.43858	0.43618	0.56566	0.628	0.35569	0.37905	0.37404	0.37954
0.632	0.42787	0.43602	0.37816	0.632	0.31106	0.43593	0.4334	0.5642	0.632	0.35658	0.37948	0.37404	0.38151
0.636	0.43005	0.43655	0.37894	0.636	0.31156	0.43261	0.43022	0.56226	0.636	0.3579	0.38034	0.37404	0.38375
0.64	0.43169	0.43602	0.37894	0.64	0.31156	0.42863	0.42704	0.56032	0.64	0.35835	0.38034	0.37404	0.386
0.644	0.43333	0.43496	0.37855	0.644	0.31106	0.42431	0.42266	0.5579	0.644	0.35923	0.37991	0.37294	0.38796
0.648	0.43443	0.43389	0.37816	0.648	0.31106	0.41934	0.41829	0.55498	0.648	0.35967	0.37948	0.37294	0.38993
0.652	0.43552	0.4323	0.377	0.652	0.30958	0.41403	0.41392	0.55159	0.652	0.35967	0.37862	0.37184	0.39189
0.656	0.43607	0.42964	0.37505	0.656	0.30809	0.40806	0.40875	0.5477	0.656	0.35967	0.37776	0.37074	0.39413
0.66	0.43607	0.42698	0.37271	0.66	0.30611	0.40142	0.40358	0.54382	0.66	0.35923	0.37605	0.36964	0.39582
0.664	0.43552	0.42378	0.3696	0.664	0.30412	0.39512	0.39801	0.53945	0.664	0.35835	0.37433	0.36799	0.39722
0.668	0.43497	0.42059	0.36687	0.668	0.30214	0.38815	0.39284	0.5346	0.668	0.35746	0.37261	0.36634	0.39891
0.672	0.43443	0.41687	0.36376	0.672	0.29917	0.38118	0.38688	0.52975	0.672	0.35614	0.3709	0.3647	0.40031
0.676	0.43224	0.41261	0.35947	0.676	0.2957	0.37355	0.38091	0.52441	0.676	0.35481	0.36832	0.36195	0.40115
0.68	0.4306	0.40782	0.35519	0.68	0.29272	0.36625	0.37495	0.51907	0.68	0.35304	0.36618	0.35975	0.40227
0.684	0.42842	0.40356	0.35091	0.684	0.28876	0.35862	0.36899	0.51325	0.684	0.35127	0.3636	0.357	0.40283
0.688	0.42568	0.39931	0.34623	0.688	0.28529	0.35132	0.36342	0.50743	0.688	0.34951	0.3606	0.35425	0.4034
0.692	0.42295	0.39452	0.34117	0.692	0.28182	0.34369	0.35706	0.5016	0.692	0.3473	0.35802	0.35151	0.4034
0.696	0.41967	0.38973	0.33611	0.696	0.27785	0.33672	0.35149	0.49578	0.696	0.34509	0.35545	0.34821	0.4034
0.7	0.41639	0.38494	0.33105	0.7	0.27439	0.32975	0.34592	0.48947	0.7	0.34244	0.35287	0.34546	0.4034
0.704	0.41311	0.38069	0.32599	0.704	0.27092	0.32278	0.34036	0.48413	0.704	0.34023	0.3503	0.34271	0.4034

0.708	0.40984	0.3759	0.32093	0.708	0.26695	0.31648	0.33519	0.47782	0.708	0.33758	0.34729	0.33942	0.40283
0.712	0.40656	0.37217	0.31625	0.712	0.26398	0.31051	0.33042	0.47297	0.712	0.33537	0.34515	0.33667	0.40227
0.716	0.40383	0.36845	0.31197	0.716	0.261	0.30486	0.32565	0.46763	0.716	0.33316	0.343	0.33392	0.40171
0.72	0.40055	0.36472	0.30769	0.72	0.25803	0.29922	0.32087	0.46229	0.72	0.33095	0.34043	0.33117	0.40115
0.724	0.39672	0.361	0.30301	0.724	0.25555	0.29391	0.3165	0.45695	0.724	0.3283	0.33828	0.32842	0.40031
0.728	0.39399	0.35728	0.29912	0.728	0.25258	0.28927	0.31252	0.45259	0.728	0.32653	0.33614	0.32568	0.39947
0.732	0.39126	0.35462	0.296	0.732	0.25059	0.28496	0.30895	0.44822	0.732	0.32432	0.33442	0.32348	0.39834
0.736	0.38907	0.35196	0.2925	0.736	0.24861	0.28097	0.30577	0.44385	0.736	0.32255	0.33313	0.32128	0.3975
0.74	0.38634	0.34876	0.28939	0.74	0.24663	0.27732	0.30179	0.43997	0.74	0.32034	0.33142	0.31908	0.3961
0.744	0.3847	0.34663	0.28705	0.744	0.24514	0.27401	0.29901	0.43609	0.744	0.31902	0.33013	0.31743	0.39498
0.748	0.38251	0.34451	0.28432	0.748	0.24366	0.27102	0.29622	0.43269	0.748	0.31769	0.32927	0.31578	0.39385
0.752	0.38087	0.34238	0.28199	0.752	0.24266	0.26837	0.29384	0.42929	0.752	0.31636	0.32841	0.31413	0.39273
0.756	0.37923	0.34078	0.28043	0.756	0.24167	0.26571	0.29145	0.42638	0.756	0.31504	0.32713	0.31304	0.39133
0.76	0.37814	0.33865	0.27809	0.76	0.24118	0.26339	0.28907	0.42347	0.76	0.31371	0.32627	0.31194	0.38993
0.764	0.3776	0.33759	0.27693	0.764	0.24019	0.26173	0.28748	0.42104	0.764	0.31283	0.32627	0.31084	0.3888
0.768	0.37705	0.33653	0.27537	0.768	0.23969	0.25974	0.28588	0.4191	0.768	0.31194	0.32541	0.30974	0.38768
0.772	0.3765	0.33493	0.27381	0.772	0.23969	0.25808	0.2839	0.41667	0.772	0.31106	0.32498	0.30919	0.38628
0.776	0.3776	0.33546	0.27381	0.776	0.24019	0.25708	0.2831	0.4157	0.776	0.31106	0.32584	0.30919	0.38572
0.78	0.37814	0.33493	0.27264	0.78	0.23969	0.25609	0.28191	0.41376	0.78	0.31106	0.32541	0.30864	0.38487
0.784	0.37814	0.3344	0.27225	0.784	0.23969	0.25509	0.28072	0.41231	0.784	0.31062	0.32584	0.30864	0.38375
0.788	0.37814	0.33387	0.27147	0.788	0.24019	0.2541	0.27952	0.41085	0.788	0.31018	0.32584	0.30809	0.38291
0.792	0.37923	0.33387	0.27108	0.792	0.24019	0.25343	0.27833	0.40988	0.792	0.31018	0.32584	0.30809	0.38207
0.796	0.38033	0.33387	0.2707	0.796	0.24068	0.25277	0.27753	0.40891	0.796	0.31018	0.32627	0.30864	0.38123
0.8	0.38197	0.33387	0.2707	0.8	0.24167	0.25244	0.27674	0.40842	0.8	0.31018	0.3267	0.30864	0.38095
0.804	0.38306	0.33387	0.27031	0.804	0.24167	0.25211	0.27594	0.40745	0.804	0.31062	0.32713	0.30919	0.38038
0.808	0.3847	0.3344	0.2707	0.808	0.24217	0.25178	0.27555	0.40697	0.808	0.31106	0.32755	0.30974	0.3801
0.812	0.38634	0.33493	0.27108	0.812	0.24316	0.25178	0.27475	0.40697	0.812	0.31106	0.32841	0.31029	0.3801
0.816	0.38798	0.33546	0.27108	0.816	0.24366	0.25144	0.27435	0.40648	0.816	0.3115	0.32927	0.31084	0.37982
0.82	0.38962	0.33599	0.27147	0.82	0.24465	0.25178	0.27396	0.40648	0.82	0.31194	0.33013	0.31139	0.37982
0.824	0.39071	0.33599	0.27147	0.824	0.24514	0.25144	0.27316	0.40551	0.824	0.31194	0.33013	0.31194	0.37926
0.828	0.39344	0.33653	0.27225	0.828	0.24663	0.25178	0.27316	0.406	0.828	0.31283	0.33142	0.31304	0.37982
0.832	0.39563	0.33759	0.27303	0.832	0.24762	0.25211	0.27316	0.406	0.832	0.31371	0.33227	0.31359	0.3801
0.836	0.39727	0.33865	0.27381	0.836	0.24812	0.25211	0.27276	0.406	0.836	0.31415	0.33313	0.31468	0.3801
0.84	0.39945	0.33919	0.27459	0.84	0.2501	0.25277	0.27276	0.40648	0.84	0.31504	0.33485	0.31578	0.38067
0.844	0.40164	0.34025	0.27537	0.844	0.25159	0.25343	0.27276	0.40697	0.844	0.31592	0.33571	0.31688	0.38123
0.848	0.40328	0.34131	0.27654	0.848	0.25258	0.2541	0.27316	0.40745	0.848	0.31636	0.337	0.31798	0.38179
0.852	0.40546	0.34291	0.2777	0.852	0.25357	0.25476	0.27356	0.40794	0.852	0.31725	0.33785	0.31908	0.38235
0.856	0.40765	0.34344	0.27848	0.856	0.25506	0.25543	0.27356	0.40842	0.856	0.31857	0.33914	0.32018	0.38291
0.86	0.40929	0.34451	0.27965	0.86	0.25654	0.25609	0.27396	0.4094	0.86	0.31946	0.34086	0.32183	0.38347
0.864	0.41202	0.3461	0.28082	0.864	0.25852	0.25742	0.27435	0.41037	0.864	0.3199	0.34214	0.32293	0.38431
0.868	0.41311	0.34717	0.28199	0.868	0.25952	0.25841	0.27475	0.41085	0.868	0.32122	0.343	0.32458	0.38459
0.872	0.4153	0.34823	0.28354	0.872	0.2615	0.25941	0.27555	0.41182	0.872	0.32211	0.34472	0.32568	0.38572
0.876	0.41803	0.35036	0.2851	0.876	0.26348	0.26107	0.27634	0.41328	0.876	0.32299	0.34644	0.32787	0.38656
0.88	0.41967	0.35142	0.28627	0.88	0.26497	0.26239	0.27714	0.41425	0.88	0.32432	0.34815	0.32897	0.38712
0.884	0.42131	0.35302	0.28783	0.884	0.26646	0.26372	0.27793	0.41522	0.884	0.32564	0.34944	0.33062	0.38768
0.888	0.4235	0.35408	0.28939	0.888	0.26844	0.26472	0.27873	0.41667	0.888	0.32653	0.35073	0.33227	0.38852
0.892	0.42514	0.35568	0.29094	0.892	0.27042	0.26671	0.27952	0.41765	0.892	0.32785	0.35244	0.33392	0.38908
0.896	0.42678	0.35728	0.2925	0.896	0.2724	0.26837	0.28072	0.4191	0.896	0.32918	0.35416	0.33557	0.38993
0.9	0.42842	0.35887	0.29406	0.9	0.27439	0.27002	0.28151	0.42056	0.9	0.33006	0.35588	0.33722	0.39021
0.904	0.4306	0.36047	0.29562	0.904	0.27637	0.27135	0.2831	0.42201	0.904	0.33139	0.35759	0.33942	0.39133
0.908	0.43279	0.36206	0.29717	0.908	0.27835	0.27301	0.28429	0.42347	0.908	0.33271	0.35931	0.34106	0.39217
0.912	0.43388	0.36313	0.29873	0.912	0.28083	0.275	0.28549	0.42492	0.912	0.33404	0.36103	0.34326	0.39273
0.916	0.43607	0.36526	0.30068	0.916	0.28281	0.27666	0.28668	0.42687	0.916	0.33537	0.36274	0.34491	0.39329
0.92	0.43716	0.36685	0.30224	0.92	0.28529	0.27865	0.28787	0.42832	0.92	0.33669	0.36446	0.34711	0.39385
0.924	0.4388	0.36845	0.30418	0.924	0.28777	0.28064	0.28986	0.43026	0.924	0.33846	0.36661	0.34931	0.3947
0.928	0.44044	0.37005	0.30574	0.928	0.29025	0.28263	0.29105	0.4322	0.928	0.33979	0.36832	0.35151	0.39554
0.932	0.44208	0.37164	0.3073	0.932	0.29223	0.28429	0.29264	0.43415	0.932	0.34155	0.37047	0.3537	0.3961
0.936	0.44317	0.37324	0.30885	0.936	0.29471	0.28661	0.29423	0.43609	0.936	0.34332	0.37218	0.3559	0.39694
0.94	0.44426	0.37483	0.3108	0.94	0.29718	0.28861	0.29622	0.43851	0.94	0.34509	0.37476	0.35865	0.39778
0.944	0.4459	0.37643	0.31236	0.944	0.29966	0.29093	0.29781	0.44045	0.944	0.34686	0.3769	0.36085	0.39891
0.948	0.44699	0.37803	0.31392	0.948	0.30214	0.29325	0.2998	0.44288	0.948	0.34818	0.37905	0.36305	0.39975
0.952	0.44809	0.37962	0.31547	0.952	0.30462	0.29524	0.30179	0.44531	0.952	0.35039	0.3812	0.36579	0.40059
0.956	0.44918	0.38122	0.31742	0.956	0.3071	0.2979	0.30378	0.44773	0.956	0.3526	0.38377	0.36799	0.40199
0.96	0.45027	0.38335	0.31937	0.96	0.31007	0.29989	0.30577	0.45065	0.96	0.35481	0.38635	0.37074	0.40311
0.964	0.45082	0.38441	0.32093	0.964	0.31255	0.30221	0.30775	0.45307	0.964	0.35658	0.38849	0.37349	0.40424
0.968	0.45191	0.38654	0.32287	0.968	0.31503	0.30486	0.31014	0.4555	0.968	0.35923	0.39149	0.37624	0.40564
0.972	0.45246	0.38867	0.32443	0.972	0.318	0.30719	0.31252	0.4589	0.972	0.36188	0.39407	0.37898	0.40704
0.976	0.45301	0.39026	0.32677	0.976	0.32048	0.30984	0.31491	0.46132	0.976	0.36409	0.39664	0.38173	0.40873
0.98	0.45301	0.39239	0.32871	0.98	0.32395	0.3125	0.31769	0.46472	0.98	0.36674	0.39965	0.38503	0.41041
0.984	0.4541	0.39399	0.33066	0.984	0.32692	0.31482	0.32008	0.46812	0.984	0.36984	0.40308	0.38778	0.41209
0.988	0.45464	0.39558	0.33261	0.988	0.3299	0.31747	0.32286	0.47151	0.988	0.37249	0.40609	0.39107	0.41406
0.992	0.45519	0.39771	0.33494	0.992	0.33287	0.32046	0.32565	0.47491	0.992	0.37558	0.40952	0.39437	0.41602
0.996	0.45574	0.39984	0.33689	0.996	0.33584	0.32278	0.32803	0.47782	0.996	0.37867	0.41252	0.39767	0.41799
1	0.45683	0.40197	0.33923	1	0.33882	0.32577	0.33082	0.4817	1	0.38177	0.41638	0.40097	0.42023
1.004	0.45792	0.40356	0.34195	1.004	0.34278	0.32875	0.334	0.48559	1.004	0.3853	0.41982	0.40426	0.42276
1.008	0.45847	0.40623	0.34429	1.008	0.34625	0.33174	0.33718	0.48947	1.008	0.3884	0.42325	0.40811	0.425
1.012	0.45956	0.40835	0.34701	1.012	0.34972	0.3344	0.33996	0.49335	1.012	0.39193	0.42711	0.41141	0.42753
1.016	0.46066	0.41101	0.34935	1.016	0.35319	0.33738	0.34274	0.49772	1.016	0.39547	0.43097	0.41526	0.43034
1.02	0.46175	0.41314	0.35246	1.02	0.35716	0.34103	0.34632	0.50209	1.02	0.39944	0.43527	0.41965	0.43342
1.024	0.46339	0.4158	0.35558	1.024	0.36112	0.34435	0.3499	0.50597	1.024	0.40298	0.43956	0.42405	0.43651
1.028	0.46393	0.41846	0.35831	1.028	0.36459	0.34767	0.35308	0.50985	1.028	0.40696	0.44342	0.42735	0.43932
1.032	0.46557	0.42219	0.36142	1.032	0.36905	0.35099	0.35706	0.5147	1.032	0.41093	0.44814	0.43229	0.44268
1.036	0.46776	0.42431	0.36531	1.036	0.37351	0.35464	0.36103	0.51907	1.036	0.41535	0.45243	0.43669	0.44605
1.04	0.46995	0.42804	0.36882	1.04	0.37797	0.35862	0.36461	0.52393	1.04	0.41977	0.45715	0.44109	0.44942
1.044	0.47213	0.4307	0.37232	1.044	0.38243	0.36227	0.36859	0.52829	1.044	0.42419	0.46187	0.44603	0.45307
1.048	0.47432	0.43389	0.37622	1.048	0.3869	0.36592	0.37296	0.53315	1.048	0.42861	0.46659	0.45098	0.45671
1.052	0.4765	0.43815	0.3805	1.052	0.39235	0.3699	0.37734	0.53848	1.052	0.43347	0.47217	0.45592	0.46064
1.056	0.47978	0.44134	0.38478	1.056	0.3973	0.37388	0.38171	0.54382	1.056	0.43833	0.47732	0.46087	0.46485
1.06	0.48251	0.44507	0.38907	1.06	0.40226	0.37819	0.38569	0.54868	1.06	0.44319	0.48204	0.46637	0.46906
1.064	0.48579	0.44932	0.39374	1.064	0.40771	0.38251	0.39046	0.55401	1.064	0.44805	0.48719	0.47186	0.47327
1.068	0.48907	0.45358	0.39841	1.068	0.41316	0.38715	0.39563	0.55935	1.068	0.45336	0.4932	0.47736	0.47776
1.072	0.49235	0.4573	0.40308	1.072	0.41862	0.3918	0.4004	0.5642	1.072	0.4591	0.49878	0.4834	0.48253
1.076	0.49617	0.46156	0.40776	1.076	0.42407	0.39644	0.40517	0.56954	1.076	0.46485	0.50436	0.48945	0.48702
1.08	0.49891	0.46582	0.41282	1.08	0.43051	0.40109	0.41034	0.57488	1.08	0.47059	0.51036	0.49549	0.49235
1.084	0.50383	0.47114	0.41827	1.084	0.43695	0.40673	0.4163	0.5807	1.084	0.47678	0.5168	0.50209	0.4974
1.088	0.5082	0.47646	0.42372	1.088	0.4434	0.41171	0.42187	0.58653	1.088	0.48252	0.52324	0.50868	0.50274
1.092	0.51257	0.48071	0.42956	1.092	0.45034	0.41668	0.42744	0.59235	1.092	0.48871	0.52925	0.51528	0.50779
1.096	0.51749	0.48657	0.4354	1.096	0.45678	0.42199	0.4334	0.59818	1.096	0.4949	0.53611	0.52242	0.5134
1.1	0.5224	0.49242	0.44124	1.1	0.46372	0.42763	0.43936	0.604	1.1	0.50152	0.54255	0.52902	0.51873
1.104	0.52732	0.49774	0.44708	1.104	0.47115	0.43361	0.44533	0.61031	1.104	0.50771	0.54941	0.53616	0.52434
1.108	0.53279	0.50306	0.45331	1.108	0.47809	0.43925	0.45129	0.61613	1.108	0.51434	0.55628	0.54331	0.52996
1.112	0.53825	0.50891	0.45993	1.112	0.48553	0.44522	0.45765	0.62195	1.112	0.52141	0.56315	0.551	0.53557
1.116	0.54372	0.5153	0.46616	1.116	0.49346	0.45152	0.46402	0.62826	1.116	0.52848	0.57001	0.55814	0.54174

1.12	0.55027	0.52168	0.47278	1.12	0.50139	0.45816	0.47078	0.63506	1.12	0.53555	0.57774	0.56584	0.54792
1.124	0.55574	0.52807	0.4794	1.124	0.50882	0.4648	0.47674	0.64137	1.124	0.54262	0.5846	0.57353	0.55409
1.128	0.56175	0.53445	0.48641	1.128	0.51725	0.47143	0.4839	0.64768	1.128	0.55013	0.59233	0.58123	0.56054
1.132	0.56831	0.5419	0.49381	1.132	0.52567	0.4784	0.49066	0.65398	1.132	0.55721	0.60005	0.58947	0.567
1.136	0.57432	0.54935	0.50121	1.136	0.5336	0.4857	0.49781	0.66078	1.136	0.56516	0.60735	0.59771	0.57373
1.14	0.58087	0.55626	0.50861	1.14	0.54253	0.493	0.50537	0.66757	1.14	0.57223	0.6155	0.60541	0.58019
1.144	0.58798	0.56318	0.51639	1.144	0.55095	0.49997	0.51292	0.67388	1.144	0.57974	0.62322	0.6142	0.58692
1.148	0.59454	0.57116	0.52496	1.148	0.56037	0.50826	0.52087	0.68116	1.148	0.58814	0.63138	0.62299	0.59422
1.152	0.60219	0.57861	0.53314	1.152	0.56929	0.51589	0.52883	0.68844	1.152	0.59654	0.63953	0.63234	0.60152
1.156	0.60929	0.58659	0.54092	1.156	0.57821	0.52386	0.53678	0.69523	1.156	0.60493	0.64768	0.64113	0.60853
1.16	0.61639	0.59457	0.54949	1.16	0.58812	0.53215	0.54553	0.703	1.16	0.61333	0.65627	0.64992	0.61611
1.164	0.62404	0.60309	0.55845	1.164	0.59754	0.54078	0.55388	0.71028	1.164	0.62172	0.66528	0.65927	0.62397
1.168	0.63224	0.61213	0.56701	1.168	0.60745	0.54974	0.56223	0.71804	1.168	0.63056	0.67386	0.66861	0.63154
1.172	0.64044	0.62064	0.57636	1.172	0.61737	0.5587	0.57097	0.72532	1.172	0.63984	0.6833	0.6785	0.6394
1.176	0.64809	0.62969	0.58531	1.176	0.62778	0.56832	0.58012	0.73309	1.176	0.64912	0.69231	0.68839	0.64726
1.18	0.65628	0.63873	0.59505	1.18	0.63818	0.57827	0.58966	0.74085	1.18	0.65884	0.70133	0.69883	0.65511
1.184	0.66612	0.64884	0.60556	1.184	0.64958	0.58889	0.6004	0.74959	1.184	0.66901	0.7112	0.70983	0.66437
1.188	0.67432	0.65789	0.61529	1.188	0.6595	0.59885	0.60994	0.75687	1.188	0.67829	0.72021	0.71972	0.67251
1.192	0.68306	0.668	0.62542	1.192	0.6699	0.60913	0.61988	0.76512	1.192	0.68801	0.72965	0.73016	0.68121
1.196	0.69235	0.67811	0.63632	1.196	0.68081	0.61975	0.63022	0.77385	1.196	0.69817	0.73909	0.74005	0.69019
1.2	0.70164	0.68768	0.64683	1.2	0.69122	0.6307	0.64095	0.7821	1.2	0.70834	0.74853	0.74995	0.69945
1.204	0.71038	0.69779	0.65735	1.204	0.70212	0.64198	0.65209	0.78987	1.204	0.7185	0.75797	0.76039	0.70843
1.208	0.72022	0.70843	0.66825	1.208	0.71253	0.65293	0.66322	0.79812	1.208	0.72822	0.76741	0.77083	0.71769
1.212	0.7306	0.71907	0.67915	1.212	0.72343	0.66521	0.67435	0.80685	1.212	0.73927	0.77728	0.78127	0.72723
1.216	0.74044	0.72972	0.69083	1.216	0.73483	0.67715	0.68588	0.8151	1.216	0.75032	0.78715	0.79226	0.73706
1.22	0.75027	0.73982	0.70174	1.22	0.74574	0.68943	0.69702	0.82335	1.22	0.76048	0.79616	0.80215	0.74688
1.224	0.76066	0.751	0.7142	1.224	0.75714	0.70204	0.70895	0.83209	1.224	0.77153	0.80646	0.81315	0.75726
1.228	0.77158	0.76217	0.72627	1.228	0.76854	0.71531	0.72127	0.83985	1.228	0.78302	0.81633	0.82304	0.76764
1.232	0.78197	0.77334	0.73873	1.232	0.78043	0.72825	0.734	0.8481	1.232	0.79407	0.82577	0.83348	0.77831
1.236	0.7929	0.78399	0.75119	1.236	0.79084	0.74086	0.74632	0.85635	1.236	0.80468	0.83479	0.84282	0.78869
1.24	0.80383	0.79622	0.76404	1.24	0.80224	0.75446	0.75944	0.8646	1.24	0.81572	0.84466	0.85326	0.79964
1.244	0.81585	0.80793	0.77728	1.244	0.81414	0.76873	0.77336	0.87334	1.244	0.82721	0.85453	0.86371	0.81114
1.248	0.82787	0.82016	0.79207	1.248	0.82653	0.78333	0.78728	0.88159	1.248	0.8387	0.8644	0.87305	0.82265
1.252	0.83934	0.83293	0.80531	1.252	0.83793	0.7976	0.80119	0.89032	1.252	0.85019	0.87427	0.88294	0.83415
1.256	0.85191	0.84517	0.81894	1.256	0.85032	0.81253	0.8159	0.89906	1.256	0.86257	0.88414	0.89228	0.84622
1.26	0.86393	0.85794	0.83413	1.26	0.8632	0.82812	0.83101	0.90828	1.26	0.87406	0.89443	0.90218	0.85857
1.264	0.8765	0.87071	0.84892	1.264	0.87559	0.84372	0.84612	0.91701	1.264	0.88599	0.90388	0.91152	0.87119
1.268	0.88907	0.88348	0.86411	1.268	0.88799	0.85998	0.86163	0.92575	1.268	0.89792	0.91417	0.92141	0.88382
1.272	0.90109	0.89731	0.87968	1.272	0.90087	0.87624	0.87753	0.934	1.272	0.91073	0.9249	0.93075	0.89701
1.276	0.91475	0.91115	0.89565	1.276	0.91525	0.89283	0.89463	0.94322	1.276	0.92355	0.93563	0.9401	0.91076
1.28	0.92842	0.92551	0.912	1.28	0.92912	0.91008	0.91093	0.95293	1.28	0.93592	0.94593	0.94999	0.92479
1.284	0.94098	0.93935	0.92835	1.284	0.94251	0.927	0.92763	0.96166	1.284	0.94785	0.95623	0.95933	0.93882
1.288	0.95628	0.95424	0.94627	1.288	0.95688	0.94558	0.94553	0.97137	1.288	0.96155	0.96739	0.96922	0.95398
1.292	0.97104	0.96914	0.96379	1.292	0.97125	0.9635	0.96302	0.98107	1.292	0.97437	0.97811	0.97967	0.96913
1.296	0.98525	0.98404	0.9817	1.296	0.98563	0.98142	0.98131	0.99029	1.296	0.98674	0.98884	0.98956	0.98429
1.3	1	1	1	1.3	1	1	1	1	1.3	1	1	1	1

**Water-ABS (Spring)**				**Water-ABS (Summer)**					**Water-ABS (Autumn)**				
0.004	0.08234	0.10855	0.10996	0.004	0.05292	0.1002	0.08001	0.05384	0.004	0.11594	0.07728	0.04164	0.0627
0.008	0.07699	0.10222	0.10192	0.008	0.04998	0.09381	0.07556	0.0506	0.008	0.1106	0.07193	0.03945	0.05851
0.012	0.07232	0.09703	0.09561	0.012	0.04728	0.0889	0.07191	0.04799	0.012	0.10679	0.06756	0.03694	0.05528
0.016	0.06788	0.09272	0.09015	0.016	0.04483	0.08448	0.06851	0.04576	0.016	0.10221	0.06392	0.03475	0.05244
0.02	0.06441	0.08955	0.08613	0.02	0.04287	0.08128	0.06564	0.04372	0.02	0.09916	0.06123	0.03319	0.04971
0.024	0.06089	0.08667	0.0824	0.024	0.04084	0.07809	0.06302	0.04226	0.024	0.09687	0.05874	0.03131	0.04751
0.028	0.05855	0.08437	0.07896	0.028	0.03939	0.07588	0.06067	0.0408	0.028	0.09535	0.05665	0.03068	0.04536
0.032	0.05599	0.08206	0.0758	0.032	0.0378	0.07367	0.05858	0.03952	0.032	0.09344	0.05477	0.02974	0.04356
0.036	0.05372	0.08033	0.07351	0.036	0.03643	0.07195	0.05649	0.03838	0.036	0.09153	0.05298	0.0288	0.04194
0.04	0.05178	0.07889	0.0715	0.04	0.03518	0.07023	0.05492	0.03742	0.04	0.09001	0.05143	0.02786	0.04036
0.044	0.05024	0.07745	0.06949	0.044	0.03395	0.06876	0.05309	0.03634	0.044	0.08848	0.05024	0.02724	0.04025
0.048	0.04854	0.07601	0.06748	0.048	0.03273	0.06753	0.05153	0.03551	0.048	0.08734	0.04877	0.02661	0.03852
0.052	0.04655	0.07478	0.06576	0.052	0.03175	0.0663	0.0497	0.03462	0.052	0.08619	0.04771	0.02599	0.03694
0.056	0.0449	0.0738	0.06432	0.056	0.03062	0.06532	0.04816	0.0338	0.056	0.08429	0.04648	0.02536	0.03564
0.06	0.04285	0.07276	0.06317	0.06	0.02972	0.06458	0.04682	0.03316	0.06	0.08352	0.04542	0.02536	0.03444
0.064	0.04114	0.07187	0.06202	0.064	0.02886	0.0636	0.04552	0.0324	0.064	0.082	0.04432	0.02473	0.03344
0.068	0.03949	0.07103	0.06088	0.068	0.02788	0.06262	0.04411	0.0315	0.068	0.08085	0.0433	0.02442	0.03241
0.072	0.03818	0.07011	0.06002	0.072	0.02705	0.06188	0.04262	0.0308	0.072	0.07933	0.04228	0.02411	0.03129
0.076	0.03687	0.06939	0.05887	0.076	0.02621	0.06114	0.04131	0.02991	0.076	0.07857	0.04146	0.02348	0.03018
0.08	0.03539	0.06856	0.05801	0.08	0.02536	0.0604	0.03992	0.02934	0.08	0.07704	0.04036	0.02348	0.02925
0.084	0.03414	0.06752	0.05686	0.084	0.0245	0.05942	0.03851	0.02845	0.084	0.0759	0.03954	0.02317	0.02818
0.088	0.03249	0.06686	0.05611	0.088	0.02362	0.05868	0.03713	0.02769	0.088	0.07475	0.0384	0.02254	0.02776
0.092	0.03124	0.06599	0.05482	0.092	0.02286	0.05819	0.03598	0.02673	0.092	0.07399	0.0377	0.02254	0.02656
0.096	0.02987	0.06502	0.05373	0.096	0.02188	0.05721	0.03452	0.02603	0.096	0.07246	0.03676	0.02223	0.02556
0.1	0.02845	0.06424	0.05281	0.1	0.02112	0.05647	0.03329	0.02527	0.1	0.07094	0.03566	0.02192	0.02437
0.104	0.02714	0.06355	0.05175	0.104	0.02031	0.05549	0.03203	0.02457	0.104	0.06979	0.0348	0.02129	0.02337
0.108	0.02589	0.06271	0.05112	0.108	0.0194	0.05475	0.03062	0.02349	0.108	0.06827	0.0339	0.02098	0.0223
0.112	0.02441	0.06176	0.05006	0.112	0.01872	0.05377	0.02929	0.02259	0.112	0.06674	0.03284	0.02035	0.02114
0.116	0.0231	0.06093	0.04942	0.116	0.01788	0.05303	0.02804	0.0217	0.116	0.06522	0.03186	0.01972	0.02018
0.12	0.02162	0.05989	0.04865	0.12	0.017	0.05205	0.02668	0.02094	0.12	0.06369	0.03096	0.0191	0.01911
0.124	0.02048	0.059	0.04756	0.124	0.01632	0.05107	0.02555	0.01986	0.124	0.06217	0.02994	0.0191	0.01811
0.128	0.01906	0.05816	0.04687	0.128	0.01546	0.05009	0.02425	0.01903	0.128	0.06064	0.02892	0.01847	0.01711
0.132	0.01792	0.05721	0.04572	0.132	0.01472	0.0491	0.02302	0.01801	0.132	0.05912	0.02794	0.01785	0.01622
0.136	0.01673	0.05621	0.04498	0.136	0.01401	0.04812	0.02174	0.01725	0.136	0.05721	0.02692	0.01753	0.0153
0.14	0.01565	0.05523	0.04426	0.14	0.01325	0.04689	0.02059	0.01623	0.14	0.05568	0.02594	0.01722	0.01422
0.144	0.01457	0.05405	0.04374	0.144	0.01254	0.04591	0.01923	0.01534	0.144	0.05378	0.02496	0.01659	0.01334
0.148	0.01343	0.05298	0.04271	0.148	0.01171	0.04468	0.01798	0.01445	0.148	0.05225	0.02381	0.01597	0.01226
0.152	0.01235	0.05189	0.04188	0.152	0.011	0.04345	0.01662	0.01343	0.152	0.04996	0.02296	0.01534	0.01138
0.156	0.01132	0.05073	0.04064	0.156	0.01046	0.04223	0.01568	0.0126	0.156	0.04882	0.02185	0.01503	0.01053
0.16	0.01019	0.04978	0.0399	0.16	0.00985	0.041	0.01474	0.01184	0.16	0.04729	0.02095	0.01472	0.00973
0.164	0.00916	0.04852	0.03889	0.164	0.00924	0.03977	0.01377	0.01101	0.164	0.045	0.01997	0.0144	0.00876
0.168	0.00825	0.04728	0.03783	0.168	0.00848	0.03854	0.01262	0.01006	0.168	0.0431	0.01912	0.01378	0.00796
0.172	0.00745	0.04593	0.03694	0.172	0.00796	0.03731	0.01176	0.00923	0.172	0.04195	0.01801	0.01346	0.00719
0.176	0.00677	0.04463	0.03582	0.176	0.00745	0.03591	0.01084	0.00834	0.176	0.04005	0.01707	0.01315	0.00638
0.18	0.00563	0.04336	0.03464	0.18	0.00691	0.03449	0.0099	0.00757	0.18	0.03776	0.01622	0.01284	0.00569
0.184	0.00512	0.04201	0.03312	0.184	0.00634	0.03326	0.00907	0.00681	0.184	0.03585	0.01515	0.01252	0.00504
0.188	0.00444	0.04063	0.0324	0.188	0.00598	0.03203	0.00831	0.00605	0.188	0.03356	0.01426	0.01221	0.00434
0.192	0.00398	0.0393	0.0314	0.192	0.00551	0.03029	0.00747	0.00541	0.192	0.03204	0.01348	0.01221	0.00377
0.196	0.00341	0.03758	0.03031	0.196	0.00492	0.02886	0.00669	0.00465	0.196	0.03013	0.01254	0.01158	0.00304
0.2	0.00307	0.03631	0.02913	0.2	0.00439	0.02761	0.00609	0.00401	0.2	0.02822	0.01156	0.01127	0.0025
0.204	0.0029	0.0347	0.02827	0.204	0.00404	0.02606	0.00543	0.00344	0.204	0.02632	0.01082	0.01127	0.00211
0.208	0.00239	0.03317	0.02715	0.208	0.0037	0.02454	0.00478	0.00293	0.208	0.02441	0.00997	0.01096	0.00165
0.212	0.00222	0.03153	0.02592	0.212	0.00309	0.02292	0.00408	0.00235	0.212	0.0225	0.00911	0.01064	0.00127
0.216	0.00199	0.02983	0.02471	0.216	0.00272	0.02144	0.00345	0.00172	0.216	0.02059	0.00825	0.01002	7.69E-04
0.22	0.00188	0.02839	0.02359	0.22	0.00243	0.01987	0.00298	0.00127	0.22	0.01869	0.00747	0.01002	6.15E-04

0.224	0.00176	0.02663	0.02224	0.224	0.00203	0.01837	0.00246	8.91E-04	0.224	0.01678	0.00662	0.00939	3.08E-04
0.228	0.00131	0.02491	0.02101	0.228	0.00167	0.01675	0.00191	5.09E-04	0.228	0.01526	0.00576	0.00877	0
0.232	0.00114	0.02335	0.0198	0.232	0.00127	0.01528	0.00146	3.18E-04	0.232	0.01335	0.00482	0.00845	1.15E-04
0.236	9.67E-04	0.02165	0.01854	0.236	9.80E-04	0.01376	0.0011	1.27E-04	0.236	0.01182	0.00413	0.00814	7.69E-05
0.24	7.97E-04	0.0199	0.01716	0.24	6.86E-04	0.01223	6.27E-04	0	0.24	0.00992	0.00331	0.00751	1.54E-04
0.244	5.12E-04	0.01823	0.01573	0.244	3.67E-04	0.01056	5.23E-04	1.27E-04	0.244	0.00839	0.00282	0.00689	5.38E-04
0.248	2.28E-04	0.0165	0.01452	0.248	7.35E-05	0.00916	5.23E-05	6.36E-05	0.248	0.00686	0.00204	0.00626	9.61E-04
0.252	2.28E-04	0.01497	0.01338	0.252	7.35E-05	0.00776	0	3.82E-04	0.252	0.00572	0.00151	0.00564	0.00154
0.256	1.14E-04	0.0133	0.01205	0.256	0	0.00636	0	7.00E-04	0.256	0.0042	0.0011	0.00501	0.00242
0.26	0	0.01143	0.01062	0.26	0.00115	0.00489	2.61E-05	0.00121	0.26	0.00267	4.49E-04	0.00407	0.00346
0.264	5.69E-05	0.01014	0.0093	0.264	0.00223	0.00359	4.18E-04	0.00204	0.264	0.00191	3.27E-04	0.00376	0.005
0.268	2.28E-04	0.00852	0.00824	0.268	0.00225	0.00258	6.01E-04	0.00286	0.268	0.00114	1.63E-04	0.00282	0.0068
0.272	0	0.00711	0.007	0.272	0.00108	0.00182	0.00115	0.00407	0.272	3.81E-04	0	0.00219	0.00896
0.276	0	0.00576	0.00574	0.276	0.00164	9.09E-04	0.00183	0.00547	0.276	0	2.86E-04	0.00157	0.01192
0.28	2.28E-04	0.00449	0.00479	0.28	0.00294	3.19E-04	0.0028	0.00726	0.28	0	5.72E-04	9.39E-04	0.01503
0.284	5.12E-04	0.0034	0.00373	0.284	0.00382	0	0.004	0.00929	0.284	3.81E-04	0.00114	6.26E-04	0.01872
0.288	9.10E-04	0.00245	0.00273	0.288	0.00505	1.23E-04	0.00554	0.01197	0.288	0.00153	0.00208	3.13E-04	0.02314
0.292	0.00131	0.00138	0.00178	0.292	0.00691	2.46E-04	0.00732	0.01502	0.292	0.00229	0.00327	0	0.02841
0.296	0.00165	7.77E-04	0.00121	0.296	0.00855	0.00103	0.00938	0.01846	0.296	0.0042	0.00482	0	0.03441
0.3	0.0025	1.73E-04	5.45E-04	0.3	0.01154	0.00226	0.01189	0.02279	0.3	0.00686	0.00686	3.13E-04	0.04148
0.304	0.00324	0	5.74E-05	0.304	0.01453	0.00381	0.01482	0.02756	0.304	0.00992	0.00939	9.39E-04	0.04948
0.308	0.00421	2.88E-05	0	0.308	0.01788	0.0059	0.01805	0.0331	0.308	0.01411	0.01221	0.00188	0.05878
0.312	0.00541	2.30E-04	1.72E-04	0.312	0.02207	0.00845	0.02192	0.03946	0.312	0.01869	0.01585	0.00313	0.06897
0.316	0.00694	8.64E-04	7.75E-04	0.316	0.02692	0.01174	0.02626	0.04652	0.316	0.02479	0.0201	0.00532	0.08042
0.32	0.00871	0.00204	0.00152	0.32	0.03197	0.01562	0.03125	0.05467	0.32	0.03127	0.02492	0.00783	0.09299
0.324	0.01058	0.00343	0.00247	0.324	0.03854	0.02007	0.03682	0.06365	0.324	0.03928	0.03023	0.01064	0.10672
0.328	0.01292	0.00544	0.00385	0.328	0.04483	0.02518	0.04293	0.07351	0.328	0.04805	0.03656	0.01472	0.12152
0.332	0.01536	0.00795	0.00586	0.332	0.05218	0.03115	0.04996	0.08439	0.332	0.05797	0.0433	0.01941	0.13762
0.336	0.01827	0.01117	0.00798	0.336	0.06051	0.03756	0.05727	0.09617	0.336	0.06903	0.05085	0.02505	0.15469
0.34	0.0214	0.01538	0.01065	0.34	0.07031	0.04468	0.0659	0.10909	0.34	0.08124	0.05886	0.03131	0.17272
0.344	0.02464	0.02047	0.01412	0.344	0.08011	0.0523	0.07504	0.12322	0.344	0.09497	0.06776	0.03882	0.19156
0.348	0.02862	0.02678	0.01825	0.348	0.09138	0.0609	0.08549	0.13868	0.348	0.11022	0.07732	0.04728	0.2127
0.352	0.03312	0.03444	0.02354	0.352	0.1051	0.07023	0.09725	0.15574	0.352	0.12662	0.08762	0.05698	0.23231
0.356	0.03801	0.04391	0.02994	0.356	0.11857	0.08055	0.11005	0.17426	0.356	0.14455	0.0986	0.06731	0.25383
0.36	0.0437	0.0554	0.03794	0.36	0.13621	0.09209	0.12495	0.19456	0.36	0.16476	0.11069	0.0789	0.27652
0.364	0.05081	0.06985	0.04785	0.364	0.1563	0.10511	0.14193	0.21773	0.364	0.18841	0.12421	0.09236	0.30112
0.368	0.05838	0.08667	0.06002	0.368	0.18006	0.1201	0.16074	0.24319	0.368	0.21358	0.13867	0.10645	0.32649
0.372	0.06749	0.10711	0.07408	0.372	0.20799	0.1368	0.18217	0.27126	0.372	0.24218	0.15477	0.12116	0.35378
0.376	0.07813	0.1313	0.09159	0.376	0.24106	0.15694	0.20621	0.30327	0.376	0.27498	0.17274	0.13713	0.38377
0.38	0.09093	0.15952	0.11197	0.38	0.2805	0.18028	0.23338	0.3391	0.38	0.31159	0.19316	0.15435	0.41645
0.384	0.10567	0.19205	0.13608	0.384	0.32558	0.20755	0.26343	0.37825	0.384	0.35355	0.21604	0.17314	0.45181
0.388	0.12274	0.22862	0.16392	0.388	0.37776	0.23874	0.29583	0.42076	0.388	0.40008	0.24148	0.19317	0.49064
0.392	0.14243	0.26922	0.19578	0.392	0.43435	0.27461	0.33084	0.4669	0.392	0.45118	0.27008	0.21572	0.53293
0.396	0.16416	0.3127	0.23079	0.396	0.49804	0.31416	0.36664	0.51502	0.396	0.50763	0.30112	0.24014	0.57829
0.4	0.18812	0.35819	0.26868	0.4	0.56614	0.3569	0.40348	0.56466	0.4	0.56827	0.3342	0.26738	0.62673
0.404	0.21344	0.40426	0.30857	0.404	0.62959	0.4021	0.43954	0.61303	0.404	0.6312	0.36933	0.29806	0.6767
0.408	0.23944	0.44976	0.3499	0.408	0.69353	0.44828	0.47324	0.65886	0.408	0.69603	0.40446	0.33281	0.72706
0.412	0.26499	0.49237	0.39095	0.412	0.75233	0.49299	0.50303	0.69959	0.412	0.76011	0.43959	0.37226	0.77703
0.416	0.28929	0.53038	0.43056	0.416	0.80304	0.53499	0.52785	0.73332	0.416	0.82189	0.47227	0.41578	0.82432
0.42	0.31034	0.56176	0.46615	0.42	0.84199	0.57184	0.54588	0.75878	0.42	0.87719	0.50167	0.46493	0.86776
0.424	0.32798	0.58508	0.49743	0.424	0.86796	0.60181	0.55659	0.77342	0.424	0.92525	0.52659	0.51879	0.90543
0.428	0.34107	0.59948	0.52154	0.428	0.87825	0.62367	0.55921	0.77788	0.428	0.96262	0.54538	0.57733	0.93657
0.432	0.34847	0.60351	0.53819	0.432	0.87261	0.63522	0.55372	0.77151	0.432	0.98856	0.55723	0.63932	0.95925
0.436	0.35075	0.59747	0.5468	0.436	0.85105	0.63595	0.5404	0.75496	0.436	1	0.56172	0.70288	0.97194
0.44	0.34733	0.58192	0.54594	0.44	0.81529	0.62711	0.52028	0.73014	0.44	0.99657	0.55804	0.76612	0.97501
0.444	0.33993	0.55831	0.53704	0.444	0.7685	0.60819	0.49519	0.69896	0.444	0.98093	0.54783	0.82718	0.96848
0.448	0.32855	0.5275	0.52011	0.448	0.71313	0.58093	0.46567	0.66268	0.448	0.95118	0.53149	0.88291	0.9531
0.452	0.31433	0.49122	0.49628	0.452	0.65238	0.54703	0.43379	0.62449	0.452	0.90999	0.50903	0.92987	0.92965
0.456	0.2984	0.45206	0.46729	0.456	0.58966	0.50895	0.40087	0.5863	0.456	0.86156	0.48329	0.9665	0.89967
0.46	0.28132	0.41146	0.43486	0.46	0.52768	0.46769	0.36795	0.54939	0.46	0.80625	0.45511	0.99061	0.86507
0.464	0.26414	0.37115	0.40013	0.464	0.46815	0.42543	0.33633	0.51566	0.464	0.74828	0.42529	1	0.82778
0.468	0.2469	0.33228	0.36483	0.468	0.41279	0.38367	0.30654	0.48479	0.468	0.69069	0.39547	0.99468	0.78895
0.472	0.22989	0.29542	0.32953	0.472	0.36232	0.34388	0.27911	0.45806	0.472	0.63463	0.36606	0.97464	0.75051
0.476	0.2135	0.26145	0.29566	0.476	0.317	0.3063	0.25429	0.43534	0.476	0.58124	0.33829	0.94145	0.71284
0.48	0.19791	0.23092	0.26351	0.48	0.27732	0.27166	0.23208	0.41662	0.48	0.53242	0.31256	0.89699	0.67785
0.484	0.18266	0.20328	0.23366	0.484	0.24253	0.24022	0.21222	0.40116	0.484	0.48818	0.28846	0.84346	0.64518
0.488	0.16815	0.17881	0.20639	0.488	0.21264	0.21197	0.19471	0.38856	0.488	0.44889	0.26722	0.7846	0.61596
0.492	0.15415	0.15721	0.18171	0.492	0.18716	0.18716	0.17956	0.37856	0.492	0.41495	0.24843	0.72323	0.5902
0.496	0.14066	0.13792	0.15932	0.496	0.16585	0.16505	0.16597	0.37042	0.496	0.3852	0.23168	0.66187	0.56752
0.5	0.1278	0.12122	0.13923	0.5	0.14748	0.14564	0.15395	0.36424	0.5	0.35965	0.21702	0.60238	0.54753
0.504	0.11545	0.10682	0.12172	0.504	0.13229	0.12869	0.1435	0.35928	0.504	0.33791	0.20407	0.54634	0.53062
0.508	0.10413	0.09415	0.10651	0.508	0.11955	0.11445	0.13462	0.35603	0.508	0.31922	0.19312	0.4953	0.51678
0.512	0.09326	0.0835	0.09274	0.512	0.10877	0.10192	0.12678	0.35349	0.512	0.30397	0.18389	0.44959	0.50525
0.516	0.08291	0.07377	0.08097	0.516	0.1002	0.09111	0.11998	0.35171	0.516	0.291	0.1758	0.40889	0.49564
0.52	0.0738	0.06582	0.07092	0.52	0.09309	0.08251	0.11423	0.35126	0.52	0.28032	0.16947	0.37351	0.48872
0.524	0.06527	0.05906	0.06202	0.524	0.0877	0.0749	0.10953	0.35132	0.524	0.27155	0.16408	0.34283	0.48334
0.528	0.0577	0.05327	0.05462	0.528	0.08329	0.069	0.10535	0.35196	0.528	0.26506	0.15991	0.31653	0.47988
0.532	0.05042	0.04866	0.04836	0.532	0.07986	0.06409	0.10222	0.35323	0.532	0.25973	0.15656	0.29399	0.47757
0.536	0.04455	0.0448	0.04302	0.536	0.07741	0.0604	0.09934	0.35521	0.536	0.25553	0.15424	0.27458	0.4768
0.54	0.03915	0.04169	0.03855	0.54	0.07594	0.05746	0.09751	0.35762	0.54	0.25362	0.15264	0.25861	0.47718
0.544	0.03454	0.03953	0.03507	0.544	0.07521	0.05549	0.09621	0.36036	0.544	0.25248	0.15183	0.24483	0.47911
0.548	0.03044	0.03778	0.03203	0.548	0.07472	0.05426	0.09516	0.36354	0.548	0.2521	0.1515	0.23325	0.48141
0.552	0.02726	0.0366	0.02999	0.552	0.07521	0.05328	0.09464	0.36736	0.552	0.25248	0.15183	0.22417	0.48526
0.556	0.02464	0.03605	0.02841	0.556	0.0757	0.05303	0.09464	0.3715	0.556	0.254	0.15277	0.21697	0.48987
0.56	0.02231	0.03585	0.02692	0.56	0.07692	0.05328	0.0949	0.37589	0.56	0.25629	0.15395	0.21102	0.49525
0.564	0.02054	0.03622	0.02635	0.564	0.07839	0.05377	0.09542	0.38035	0.564	0.25896	0.15567	0.20632	0.50102
0.568	0.01929	0.03691	0.02589	0.568	0.08035	0.05451	0.09647	0.38537	0.568	0.26278	0.15759	0.20288	0.50755
0.572	0.01866	0.03801	0.02598	0.572	0.08256	0.05574	0.09751	0.39053	0.572	0.26659	0.16016	0.201	0.51486
0.576	0.01838	0.03953	0.02626	0.576	0.08501	0.05672	0.09908	0.39581	0.576	0.27079	0.16269	0.20006	0.52255
0.58	0.01821	0.04115	0.02666	0.58	0.08746	0.05819	0.10039	0.40097	0.58	0.27574	0.16531	0.19944	0.53023
0.584	0.01855	0.04342	0.02755	0.584	0.09015	0.05967	0.10248	0.40663	0.584	0.28032	0.16816	0.19975	0.53869
0.588	0.01935	0.04598	0.02882	0.588	0.09334	0.06139	0.10431	0.41242	0.588	0.28566	0.17102	0.201	0.54715
0.592	0.02014	0.04881	0.03019	0.592	0.09652	0.0636	0.10666	0.41802	0.592	0.29138	0.17401	0.20257	0.55561
0.596	0.02122	0.05212	0.03189	0.596	0.1002	0.06556	0.10901	0.42388	0.596	0.29672	0.17744	0.20445	0.56445
0.6	0.02236	0.05569	0.03381	0.6	0.10387	0.06777	0.11162	0.42974	0.6	0.30282	0.18066	0.20664	0.57291
0.604	0.02384	0.05986	0.03619	0.604	0.10804	0.07048	0.11476	0.43591	0.604	0.30892	0.1843	0.20914	0.58175
0.608	0.02532	0.06458	0.03886	0.608	0.11244	0.07318	0.11815	0.44196	0.608	0.31541	0.18781	0.21165	0.59059
0.612	0.02691	0.06948	0.04176	0.612	0.11685	0.07637	0.12155	0.44807	0.612	0.32189	0.19149	0.21446	0.59905
0.616	0.02851	0.07481	0.04515	0.616	0.12175	0.07981	0.12573	0.45488	0.616	0.32876	0.19569	0.2176	0.6075
0.62	0.02987	0.08033	0.04868	0.62	0.1269	0.0835	0.12991	0.46175	0.62	0.33562	0.20002	0.22104	0.61596
0.624	0.03169	0.08638	0.05258	0.624	0.13204	0.08718	0.13462	0.46843	0.624	0.34249	0.20464	0.22386	0.62403
0.628	0.033	0.09243	0.05686	0.628	0.13719	0.09111	0.13958	0.47543	0.628	0.34973	0.2095	0.2273	0.63211
0.632	0.03471	0.09905	0.06145	0.632	0.14282	0.09529	0.14507	0.48262	0.632	0.35812	0.21461	0.23043	0.64018

0.636	0.03619	0.1051	0.06604	0.636	0.14821	0.09971	0.15082	0.48988	0.636	0.36651	0.21992	0.23388	0.64825
0.64	0.03767	0.11172	0.07092	0.64	0.1536	0.10437	0.15656	0.49726	0.64	0.3749	0.22576	0.23732	0.65594
0.644	0.03903	0.11777	0.0758	0.644	0.1585	0.10855	0.16257	0.50446	0.644	0.3833	0.23131	0.24108	0.66325
0.648	0.04091	0.12381	0.08039	0.648	0.16389	0.11322	0.16858	0.5119	0.648	0.39207	0.2374	0.24483	0.67055
0.652	0.04222	0.12957	0.08499	0.652	0.17099	0.11764	0.17433	0.51884	0.652	0.40084	0.24393	0.24922	0.67747
0.656	0.04325	0.13504	0.08958	0.656	0.17173	0.12182	0.18034	0.52584	0.656	0.40923	0.25006	0.25329	0.68439
0.66	0.04478	0.13994	0.09331	0.66	0.1754	0.12599	0.18557	0.5322	0.66	0.41724	0.25619	0.25798	0.69015
0.664	0.04638	0.14426	0.09733	0.664	0.17859	0.12992	0.19079	0.53857	0.664	0.42563	0.26191	0.26268	0.69592
0.668	0.04831	0.148	0.10049	0.668	0.18128	0.13361	0.19576	0.5443	0.668	0.43326	0.26763	0.26769	0.7013
0.672	0.05059	0.15117	0.10336	0.672	0.18349	0.13729	0.2002	0.55003	0.672	0.44088	0.27334	0.27301	0.70707
0.676	0.05263	0.15347	0.10565	0.676	0.1852	0.14024	0.20386	0.55448	0.676	0.44737	0.27784	0.27802	0.71091
0.68	0.05514	0.1552	0.10795	0.68	0.18594	0.14319	0.20725	0.55894	0.68	0.45271	0.28274	0.28334	0.71476
0.684	0.05804	0.15664	0.10938	0.684	0.18716	0.14613	0.21013	0.56275	0.684	0.45767	0.28682	0.28898	0.71783
0.688	0.06134	0.1575	0.11053	0.688	0.1879	0.14908	0.21248	0.56594	0.688	0.46186	0.29009	0.29399	0.71976
0.692	0.06487	0.15808	0.11168	0.692	0.18839	0.15203	0.21457	0.56848	0.692	0.46529	0.29295	0.299	0.72129
0.696	0.06879	0.15808	0.11283	0.696	0.18912	0.15522	0.21614	0.57103	0.696	0.46758	0.2954	0.30369	0.72245
0.7	0.0734	0.15865	0.11369	0.7	0.18986	0.15891	0.21797	0.57294	0.7	0.46949	0.29703	0.3087	0.72283
0.704	0.0787	0.15894	0.11484	0.704	0.1901	0.16259	0.21953	0.57421	0.704	0.47025	0.29908	0.31246	0.72283
0.708	0.08444	0.15923	0.11627	0.708	0.19206	0.16677	0.2211	0.57548	0.708	0.47063	0.29989	0.31622	0.72129
0.712	0.09093	0.16009	0.11828	0.712	0.19402	0.17193	0.22293	0.57676	0.712	0.47025	0.30153	0.31935	0.72052
0.716	0.09827	0.16153	0.12058	0.716	0.19647	0.17782	0.22476	0.57803	0.716	0.46987	0.30234	0.32185	0.7186
0.72	0.10646	0.16326	0.12345	0.72	0.19966	0.18421	0.22711	0.57867	0.72	0.46873	0.30357	0.32373	0.71668
0.724	0.11511	0.16585	0.12689	0.724	0.20333	0.19133	0.22946	0.57994	0.724	0.46758	0.3048	0.32467	0.71399
0.728	0.12473	0.16902	0.1312	0.728	0.20799	0.19944	0.23234	0.58121	0.728	0.46644	0.30602	0.3253	0.71168
0.732	0.13492	0.17276	0.13636	0.732	0.21338	0.20853	0.23599	0.58248	0.732	0.46568	0.30806	0.32561	0.70899
0.736	0.14618	0.17766	0.14239	0.736	0.21975	0.21835	0.24044	0.58439	0.736	0.46491	0.31011	0.32561	0.70669
0.74	0.15768	0.18341	0.14899	0.74	0.22636	0.22892	0.24488	0.58567	0.74	0.46453	0.31256	0.32467	0.70399
0.744	0.17014	0.19004	0.15703	0.744	0.2342	0.24022	0.25037	0.58758	0.744	0.46453	0.31582	0.32405	0.70207
0.748	0.18283	0.19752	0.16564	0.748	0.24277	0.2525	0.25638	0.59012	0.748	0.46453	0.3195	0.32342	0.70053
0.752	0.1958	0.20616	0.1754	0.752	0.25208	0.26527	0.26265	0.59267	0.752	0.46568	0.32358	0.32217	0.699
0.756	0.20934	0.21509	0.18573	0.756	0.26164	0.27854	0.26944	0.59521	0.756	0.46644	0.32808	0.32154	0.69784
0.76	0.223	0.22517	0.19721	0.76	0.27217	0.29254	0.27702	0.59776	0.76	0.46834	0.33339	0.32091	0.69707
0.764	0.23688	0.2364	0.20955	0.764	0.28319	0.30728	0.28486	0.60094	0.764	0.47063	0.33951	0.3206	0.69707
0.768	0.25088	0.24791	0.22275	0.768	0.29446	0.32177	0.29269	0.60412	0.768	0.47368	0.34564	0.3206	0.69784
0.772	0.26494	0.26001	0.23624	0.772	0.30598	0.33626	0.30106	0.60667	0.772	0.47597	0.35259	0.32029	0.69823
0.776	0.27962	0.27354	0.25146	0.776	0.31823	0.35149	0.3102	0.61113	0.776	0.48131	0.36035	0.32185	0.70092
0.78	0.29384	0.28678	0.26638	0.78	0.33023	0.36599	0.31908	0.61494	0.78	0.48589	0.36852	0.32279	0.70284
0.784	0.30693	0.30003	0.28131	0.784	0.3415	0.37999	0.32797	0.61876	0.784	0.4897	0.37668	0.32405	0.70553
0.788	0.32116	0.31385	0.29652	0.788	0.35277	0.39325	0.33659	0.62195	0.788	0.49504	0.38567	0.32561	0.70784
0.792	0.33538	0.32796	0.31144	0.792	0.36379	0.40603	0.34521	0.62513	0.792	0.50038	0.39466	0.32749	0.7113
0.796	0.34961	0.34178	0.32637	0.796	0.37433	0.41757	0.35357	0.62895	0.796	0.50572	0.40405	0.32968	0.71476
0.8	0.36383	0.3556	0.34043	0.8	0.38437	0.42863	0.36194	0.63213	0.8	0.51144	0.41386	0.3325	0.71899
0.804	0.37749	0.36856	0.35392	0.804	0.39319	0.43796	0.36951	0.63531	0.804	0.51678	0.42366	0.33532	0.72245
0.808	0.39115	0.38123	0.36684	0.808	0.40176	0.44656	0.37709	0.63786	0.808	0.52365	0.43387	0.33876	0.72629
0.812	0.40423	0.39361	0.37861	0.812	0.40936	0.45368	0.38441	0.64104	0.812	0.52937	0.44408	0.3422	0.7309
0.816	0.41732	0.40541	0.38923	0.816	0.41597	0.45982	0.39094	0.64358	0.816	0.53547	0.4547	0.34596	0.73475
0.82	0.4287	0.41635	0.39812	0.82	0.42185	0.46425	0.39721	0.64549	0.82	0.54081	0.4645	0.34941	0.73859
0.824	0.44008	0.42614	0.40616	0.824	0.42626	0.46744	0.4027	0.6474	0.824	0.54615	0.4739	0.35254	0.74205
0.828	0.45089	0.43565	0.41305	0.828	0.43092	0.46965	0.40844	0.64995	0.828	0.55187	0.4837	0.35661	0.7459
0.832	0.4617	0.44428	0.41879	0.832	0.43435	0.47088	0.41367	0.65122	0.832	0.55721	0.49269	0.36068	0.74974
0.836	0.47081	0.45148	0.42281	0.836	0.43704	0.47088	0.41811	0.65249	0.836	0.56217	0.50127	0.36475	0.75282
0.84	0.47991	0.45839	0.42625	0.84	0.439	0.47014	0.42255	0.65377	0.84	0.56751	0.50984	0.36882	0.75628
0.844	0.48845	0.46415	0.42797	0.844	0.43998	0.46818	0.42673	0.65504	0.844	0.57208	0.5176	0.37289	0.75935
0.848	0.49642	0.46876	0.42883	0.848	0.44096	0.46572	0.43013	0.65568	0.848	0.57628	0.52455	0.37696	0.76204
0.852	0.50324	0.4725	0.42855	0.852	0.44096	0.46228	0.43353	0.65568	0.852	0.58047	0.53108	0.3804	0.76473
0.856	0.5095	0.47538	0.42769	0.856	0.44072	0.45835	0.4364	0.65631	0.856	0.58352	0.5368	0.38384	0.76666
0.86	0.51576	0.47768	0.42625	0.86	0.44023	0.45442	0.43928	0.65631	0.86	0.58658	0.54211	0.3876	0.76896
0.864	0.52088	0.47941	0.42395	0.864	0.43974	0.44975	0.44163	0.65631	0.864	0.58963	0.5462	0.39073	0.77088
0.868	0.52544	0.48028	0.4208	0.868	0.43851	0.44484	0.44346	0.65631	0.868	0.59153	0.54987	0.39355	0.77242
0.872	0.52942	0.48085	0.41764	0.872	0.43778	0.44042	0.44529	0.65568	0.872	0.5942	0.55273	0.39668	0.77396
0.876	0.5334	0.48114	0.41477	0.876	0.43729	0.436	0.44712	0.65568	0.876	0.59611	0.55559	0.3995	0.77588
0.88	0.53625	0.48085	0.41104	0.88	0.43631	0.43133	0.44842	0.65504	0.88	0.59764	0.55723	0.402	0.77665
0.884	0.53852	0.48028	0.40759	0.884	0.43557	0.42666	0.44947	0.65377	0.884	0.5984	0.55845	0.4042	0.77742
0.888	0.54023	0.47912	0.40415	0.888	0.43484	0.422	0.45025	0.65249	0.888	0.59916	0.55886	0.40639	0.77857
0.892	0.54194	0.47855	0.40099	0.892	0.43484	0.41856	0.45103	0.65186	0.892	0.59992	0.55927	0.40827	0.77896
0.896	0.54307	0.47768	0.39812	0.896	0.43484	0.41512	0.45208	0.65059	0.896	0.60031	0.55968	0.41014	0.77973
0.9	0.54364	0.47682	0.39554	0.9	0.43508	0.41217	0.45234	0.64931	0.9	0.60031	0.55927	0.4114	0.77934
0.904	0.54364	0.47625	0.39324	0.904	0.43582	0.40947	0.45313	0.64868	0.904	0.60069	0.55886	0.41296	0.77973
0.908	0.54364	0.47596	0.39181	0.908	0.43704	0.40775	0.45391	0.6474	0.908	0.60107	0.55845	0.41421	0.78011
0.912	0.54364	0.47567	0.39066	0.912	0.43827	0.40603	0.45469	0.64677	0.912	0.60107	0.55804	0.41515	0.78011
0.916	0.54307	0.47567	0.39009	0.916	0.44023	0.40529	0.45548	0.64549	0.916	0.60107	0.55763	0.41641	0.77973
0.92	0.54251	0.47625	0.3898	0.92	0.44243	0.40529	0.456	0.64486	0.92	0.60107	0.55682	0.41766	0.77934
0.924	0.54194	0.47711	0.39037	0.924	0.44512	0.40578	0.45704	0.64422	0.924	0.60145	0.55641	0.41828	0.77896
0.928	0.5408	0.47826	0.39123	0.928	0.44806	0.40652	0.45809	0.64358	0.928	0.60221	0.55641	0.41954	0.77896
0.932	0.53966	0.47941	0.39238	0.932	0.451	0.40775	0.45887	0.64295	0.932	0.60221	0.55559	0.42048	0.7778
0.936	0.53852	0.48114	0.39439	0.936	0.45468	0.40996	0.46044	0.64295	0.936	0.60297	0.55559	0.42173	0.77742
0.94	0.53795	0.48344	0.39726	0.94	0.45884	0.41241	0.46201	0.64295	0.94	0.60374	0.556	0.42298	0.77742
0.944	0.53682	0.48604	0.40013	0.944	0.46325	0.41561	0.46332	0.64295	0.944	0.6045	0.556	0.42455	0.77665
0.948	0.53568	0.48891	0.40358	0.948	0.46815	0.41905	0.46488	0.64358	0.948	0.60526	0.556	0.42611	0.77588
0.952	0.53511	0.49208	0.40759	0.952	0.47281	0.42322	0.46671	0.64422	0.952	0.60679	0.55682	0.42799	0.7755
0.956	0.53454	0.49554	0.41219	0.956	0.47844	0.42814	0.46906	0.64549	0.956	0.60793	0.55723	0.42987	0.7755
0.96	0.53454	0.49957	0.41707	0.96	0.48408	0.43305	0.47142	0.6474	0.96	0.60946	0.55845	0.43206	0.77511
0.964	0.53397	0.5036	0.42223	0.964	0.48996	0.43821	0.47403	0.64804	0.964	0.61098	0.55927	0.43394	0.77511
0.968	0.53454	0.50821	0.42826	0.968	0.49633	0.44435	0.4769	0.65059	0.968	0.61289	0.5609	0.43644	0.7755
0.972	0.53511	0.5131	0.43457	0.972	0.50318	0.45074	0.4803	0.65249	0.972	0.6148	0.56254	0.43926	0.7755
0.976	0.53568	0.51828	0.44118	0.976	0.51004	0.45761	0.48396	0.65504	0.976	0.61747	0.56417	0.44208	0.77627
0.98	0.53738	0.52375	0.44835	0.98	0.51739	0.46498	0.48788	0.65822	0.98	0.61937	0.56662	0.44521	0.77703
0.984	0.53909	0.52923	0.45581	0.984	0.52523	0.4726	0.49206	0.66077	0.984	0.62243	0.56866	0.44865	0.77819
0.988	0.5408	0.53527	0.46356	0.988	0.53307	0.48046	0.4965	0.66395	0.988	0.6251	0.57152	0.45241	0.77973
0.992	0.54307	0.54161	0.47189	0.992	0.5414	0.48857	0.5012	0.66777	0.992	0.62815	0.57479	0.45679	0.78126
0.996	0.54478	0.54765	0.48021	0.996	0.54949	0.49716	0.50591	0.67095	0.996	0.63082	0.57765	0.46055	0.7828
1	0.5482	0.55428	0.48939	1	0.55806	0.50601	0.51139	0.67541	1	0.63425	0.58133	0.46493	0.78472
1.004	0.55104	0.56119	0.49801	1.004	0.56712	0.51509	0.51714	0.67923	1.004	0.63768	0.58541	0.46994	0.78703
1.008	0.55446	0.5681	0.50748	1.008	0.57643	0.52443	0.52289	0.68368	1.008	0.64111	0.5899	0.47495	0.78934
1.012	0.55787	0.57472	0.51695	1.012	0.5855	0.53352	0.52864	0.68814	1.012	0.64493	0.59399	0.47996	0.79203
1.016	0.56128	0.58134	0.52671	1.016	0.59505	0.54261	0.53465	0.69259	1.016	0.64798	0.59848	0.4856	0.79472
1.02	0.56527	0.58854	0.53647	1.02	0.60461	0.55219	0.54066	0.69705	1.02	0.65256	0.60338	0.49155	0.79779
1.024	0.56925	0.59516	0.54622	1.024	0.61391	0.56152	0.54693	0.70214	1.024	0.65675	0.60869	0.49781	0.8001
1.028	0.5738	0.60179	0.5557	1.028	0.62298	0.57037	0.55241	0.70659	1.028	0.66056	0.61359	0.50407	0.80241
1.032	0.57779	0.60841	0.56545	1.032	0.63229	0.57995	0.55869	0.71169	1.032	0.66476	0.6189	0.51096	0.8051
1.036	0.58234	0.61474	0.57521	1.036	0.64111	0.58903	0.56469	0.71614	1.036	0.66857	0.62421	0.51785	0.8074
1.04	0.58689	0.62136	0.58411	1.04	0.64944	0.59788	0.57097	0.72123	1.04	0.67239	0.62952	0.52473	0.80933
1.044	0.59144	0.62712	0.59301	1.044	0.65728	0.60623	0.57645	0.72569	1.044	0.67582	0.63443	0.53225	0.81086

1.048	0.59599	0.63259	0.60162	1.048	0.66512	0.61458	0.5822	0.73014	1.048	0.67925	0.63974	0.53914	0.8124
1.052	0.60055	0.63807	0.60966	1.052	0.67222	0.62244	0.58743	0.7346	1.052	0.68345	0.64464	0.54665	0.81317
1.056	0.6051	0.64325	0.6174	1.056	0.67883	0.6303	0.59265	0.73905	1.056	0.68612	0.64954	0.55416	0.81432
1.06	0.60965	0.64757	0.62401	1.06	0.68447	0.63718	0.59736	0.74287	1.06	0.68879	0.65403	0.56074	0.81471
1.064	0.6142	0.65189	0.63061	1.064	0.6901	0.64406	0.60206	0.74669	1.064	0.69146	0.65893	0.56794	0.81548
1.068	0.61932	0.65621	0.63663	1.068	0.69451	0.65045	0.6065	0.75115	1.068	0.69413	0.66302	0.57483	0.81548
1.072	0.62331	0.65995	0.64209	1.072	0.69868	0.65634	0.61068	0.75496	1.072	0.69603	0.6671	0.58109	0.81548
1.076	0.62843	0.6634	0.64668	1.076	0.70186	0.66224	0.6146	0.75878	1.076	0.69832	0.67078	0.58704	0.81509
1.08	0.63298	0.66657	0.65099	1.08	0.7048	0.66764	0.61826	0.76197	1.08	0.69947	0.67445	0.59267	0.81471
1.084	0.6381	0.67003	0.65529	1.084	0.70774	0.67305	0.62218	0.76578	1.084	0.70175	0.67854	0.59894	0.81471
1.088	0.64265	0.67319	0.65874	1.088	0.70946	0.67796	0.62557	0.76897	1.088	0.70328	0.68181	0.60394	0.81394
1.092	0.64664	0.6755	0.66161	1.092	0.71093	0.68213	0.62845	0.77215	1.092	0.70404	0.68426	0.60802	0.81317
1.096	0.65119	0.67838	0.66476	1.096	0.7124	0.6868	0.63158	0.77533	1.096	0.70481	0.68712	0.61271	0.8124
1.1	0.65517	0.68068	0.66735	1.1	0.71362	0.69098	0.63472	0.77915	1.1	0.70595	0.68998	0.61647	0.81163
1.104	0.65972	0.68298	0.66993	1.104	0.7146	0.69515	0.63786	0.7817	1.104	0.70671	0.69243	0.61991	0.81125
1.108	0.66428	0.68557	0.67223	1.108	0.71558	0.69908	0.64047	0.78488	1.108	0.70671	0.69488	0.62304	0.81048
1.112	0.66883	0.68788	0.67423	1.112	0.71656	0.70277	0.64334	0.78806	1.112	0.70709	0.69733	0.62523	0.81009
1.116	0.67338	0.69076	0.67653	1.116	0.71779	0.70694	0.64648	0.79124	1.116	0.70824	0.69978	0.62774	0.80971
1.12	0.6785	0.69392	0.67911	1.12	0.71926	0.71112	0.64961	0.79442	1.12	0.70862	0.70223	0.63024	0.81009
1.124	0.68249	0.69652	0.68141	1.124	0.71999	0.71456	0.65249	0.79697	1.124	0.70786	0.70468	0.63181	0.80933
1.128	0.68761	0.69968	0.68399	1.128	0.72195	0.71874	0.65588	0.80079	1.128	0.709	0.70713	0.63338	0.80971
1.132	0.69273	0.70314	0.68715	1.132	0.72391	0.72291	0.65928	0.80334	1.132	0.70976	0.70999	0.63463	0.81009
1.136	0.69785	0.70659	0.69031	1.136	0.72611	0.72684	0.66268	0.80652	1.136	0.71053	0.71285	0.63619	0.81048
1.14	0.70297	0.71034	0.69346	1.14	0.72856	0.73126	0.6666	0.8097	1.14	0.71091	0.7153	0.63713	0.81125
1.144	0.70809	0.71466	0.6972	1.144	0.73126	0.73568	0.67026	0.81352	1.144	0.71167	0.71857	0.63838	0.8124
1.148	0.71378	0.71926	0.70121	1.148	0.73469	0.7406	0.6747	0.81734	1.148	0.7132	0.72184	0.63964	0.81355
1.152	0.71947	0.72387	0.70581	1.152	0.73836	0.74551	0.67914	0.82116	1.152	0.7151	0.7251	0.6412	0.81509
1.156	0.72459	0.72876	0.71011	1.156	0.74204	0.75018	0.68358	0.82434	1.156	0.71625	0.72837	0.64214	0.81625
1.16	0.73028	0.73424	0.71499	1.16	0.7462	0.75558	0.68828	0.82816	1.16	0.71854	0.73246	0.64371	0.81817
1.164	0.73597	0.73999	0.72044	1.164	0.75086	0.76074	0.69351	0.83198	1.164	0.72006	0.73695	0.64527	0.82047
1.168	0.74223	0.74604	0.72561	1.168	0.75551	0.7659	0.69874	0.83579	1.168	0.72235	0.74103	0.64715	0.82317
1.172	0.74792	0.75238	0.73135	1.172	0.76041	0.7713	0.70396	0.83961	1.172	0.72387	0.74553	0.6484	0.82547
1.176	0.75361	0.75871	0.73738	1.176	0.76556	0.77695	0.70945	0.84343	1.176	0.72578	0.75002	0.65028	0.82816
1.18	0.75987	0.76504	0.74369	1.18	0.77119	0.7826	0.71572	0.84725	1.18	0.72883	0.75533	0.65279	0.83124
1.184	0.76727	0.77282	0.75116	1.184	0.77732	0.78899	0.72251	0.85234	1.184	0.73265	0.76146	0.65623	0.83547
1.188	0.77296	0.77887	0.75718	1.188	0.7827	0.7939	0.72852	0.85616	1.188	0.73455	0.76636	0.65842	0.83816
1.192	0.77979	0.78578	0.76407	1.192	0.78858	0.80004	0.73505	0.86062	1.192	0.7376	0.77208	0.66093	0.842
1.196	0.78605	0.79297	0.77067	1.196	0.79397	0.80545	0.74159	0.86507	1.196	0.74142	0.7782	0.66406	0.84585
1.2	0.79288	0.79988	0.77727	1.2	0.79961	0.8111	0.74812	0.87016	1.2	0.74447	0.78433	0.66719	0.84969
1.204	0.79914	0.80651	0.78359	1.204	0.80524	0.81626	0.75491	0.87398	1.204	0.7479	0.79046	0.67032	0.85353
1.208	0.80596	0.81399	0.79019	1.208	0.81088	0.82191	0.76171	0.87844	1.208	0.75133	0.79699	0.67376	0.85776
1.212	0.81279	0.8209	0.79708	1.212	0.81676	0.82756	0.76902	0.88353	1.212	0.75515	0.80353	0.67752	0.86199
1.216	0.81962	0.82781	0.80397	1.216	0.82264	0.83321	0.77634	0.88798	1.216	0.75934	0.81088	0.68159	0.86661
1.22	0.82588	0.83473	0.81086	1.22	0.82827	0.83861	0.78339	0.89244	1.22	0.76316	0.81742	0.68535	0.87083
1.224	0.83328	0.84164	0.81803	1.224	0.83439	0.84451	0.79123	0.89753	1.224	0.76697	0.82477	0.68973	0.87583
1.228	0.8401	0.84826	0.82492	1.228	0.84076	0.85065	0.79907	0.90262	1.228	0.77155	0.83212	0.69411	0.88083
1.232	0.84807	0.85488	0.83209	1.232	0.84713	0.85654	0.80743	0.90771	1.232	0.77613	0.83947	0.6985	0.88621
1.236	0.8549	0.86122	0.83927	1.236	0.85301	0.86244	0.81527	0.91217	1.236	0.77994	0.84642	0.70257	0.89082
1.24	0.86173	0.86813	0.84673	1.24	0.85987	0.86858	0.82389	0.91662	1.24	0.78413	0.85418	0.70695	0.89621
1.244	0.86912	0.87504	0.85448	1.244	0.86698	0.87521	0.83251	0.92172	1.244	0.78833	0.86194	0.71165	0.90197
1.248	0.87652	0.88195	0.86281	1.248	0.87433	0.88209	0.84192	0.92681	1.248	0.79405	0.87011	0.71634	0.90774
1.252	0.88392	0.88914	0.87084	1.252	0.88143	0.88897	0.85107	0.9319	1.252	0.79825	0.87828	0.72104	0.91312
1.256	0.89189	0.89692	0.87974	1.256	0.88927	0.89634	0.86126	0.93699	1.256	0.80282	0.88686	0.72542	0.91927
1.26	0.90042	0.90469	0.88864	1.26	0.89784	0.9042	0.87171	0.94272	1.26	0.8074	0.89543	0.73043	0.92542
1.264	0.90896	0.91247	0.89811	1.264	0.90642	0.91181	0.88242	0.94781	1.264	0.81274	0.90442	0.73482	0.93196
1.268	0.91692	0.92082	0.90729	1.268	0.91499	0.91967	0.89313	0.9529	1.268	0.81732	0.913	0.7392	0.93849
1.272	0.92603	0.92917	0.91705	1.272	0.92406	0.92852	0.90463	0.95799	1.272	0.82304	0.9228	0.74389	0.94503
1.276	0.93513	0.93838	0.92796	1.276	0.93386	0.93761	0.91691	0.96372	1.276	0.82914	0.9326	0.74859	0.95272
1.28	0.9448	0.94731	0.93915	1.28	0.9439	0.94694	0.92919	0.96945	1.28	0.83486	0.94282	0.75329	0.96002
1.284	0.95391	0.9571	0.94977	1.284	0.9537	0.95628	0.94199	0.97454	1.284	0.8402	0.95303	0.75736	0.96732
1.288	0.96529	0.96775	0.96183	1.288	0.96521	0.96684	0.9561	0.98154	1.288	0.8463	0.96487	0.76268	0.97578
1.292	0.97667	0.97812	0.97446	1.292	0.97648	0.97765	0.97021	0.98727	1.292	0.8524	0.9759	0.76769	0.98347
1.296	0.98805	0.98906	0.9868	1.296	0.98824	0.98845	0.98485	0.99364	1.296	0.85889	0.98775	0.7727	0.99193
1.3	1	1	1	1.3	1	1	1	1	1.3	0.86499	1	0.77771	1

**Water-PBS (Spring)**				**Water-PBS (Summer)**					**Water-PBS (Autumn)**				
0.004	0.04462	0.07424	0.04248	0.004	0.01364	0.06043	0.02983	0.00758	0.004	0.02564	0.02413	0.01966	0.0147
0.008	0.04165	0.07053	0.0392	0.008	0.0123	0.05699	0.0277	0.0063	0.008	0.02361	0.02211	0.01812	0.01273
0.012	0.03916	0.06733	0.03625	0.012	0.01101	0.0538	0.02557	0.00534	0.012	0.02172	0.02017	0.01657	0.01121
0.016	0.03677	0.0643	0.03365	0.016	0.01001	0.05085	0.02344	0.00457	0.016	0.01989	0.01826	0.01538	0.00978
0.02	0.03485	0.06177	0.03149	0.02	0.009	0.0479	0.02131	0.0037	0.02	0.01834	0.01657	0.01433	0.00849
0.024	0.03288	0.05907	0.02933	0.024	0.00814	0.04544	0.01918	0.00295	0.024	0.01663	0.01482	0.01313	0.00734
0.028	0.0314	0.05705	0.02726	0.028	0.00734	0.04299	0.01731	0.00227	0.028	0.01525	0.01334	0.01222	0.00632
0.032	0.02989	0.05452	0.02557	0.032	0.0066	0.04078	0.01518	0.00171	0.032	0.01382	0.012	0.0112	0.00543
0.036	0.02835	0.0525	0.02378	0.036	0.00589	0.03832	0.01305	0.0014	0.036	0.01242	0.01038	0.01039	0.00448
0.04	0.027	0.05031	0.02206	0.04	0.00535	0.03586	0.01145	9.32E-04	0.04	0.01102	0.00909	0.00941	0.0037
0.044	0.02572	0.04845	0.02067	0.044	0.00475	0.0339	0.00986	5.90E-04	0.044	0.00973	0.00777	0.0085	0.00299
0.048	0.02446	0.04643	0.01914	0.048	0.00412	0.03169	0.00826	3.11E-04	0.048	0.00847	0.00659	0.00765	0.00228
0.052	0.02338	0.04475	0.01758	0.052	0.00361	0.02972	0.00693	1.24E-04	0.052	0.00744	0.00554	0.00685	0.00173
0.056	0.02209	0.04289	0.01628	0.056	0.00306	0.02751	0.00559	0	0.056	0.00632	0.0043	0.00583	0.00119
0.06	0.02107	0.04087	0.01496	0.06	0.00265	0.02579	0.00453	1.24E-04	0.06	0.00541	0.00347	0.00506	8.15E-04
0.064	0.01984	0.03902	0.01366	0.064	0.00211	0.02358	0.0032	2.17E-04	0.064	0.00426	0.00247	0.00414	4.08E-04
0.068	0.01873	0.03699	0.01242	0.068	0.00168	0.02162	0.0024	4.97E-04	0.068	0.00341	0.0018	0.00337	1.36E-04
0.072	0.01761	0.03531	0.01131	0.072	0.00129	0.0199	0.00133	9.32E-04	0.072	0.0026	0.0011	0.00263	0
0.076	0.01653	0.03345	0.0101	0.076	9.69E-04	0.01793	7.99E-04	0.00158	0.076	0.00195	6.19E-04	0.00193	1.02E-04
0.08	0.01544	0.0316	0.00876	0.08	8.01E-04	0.01621	2.66E-04	0.00245	0.08	0.00117	1.61E-04	0.00126	2.38E-04
0.084	0.01444	0.03008	0.00769	0.084	4.47E-04	0.01449	0	0.00354	0.084	5.72E-04	0	8.08E-04	5.77E-04
0.088	0.01327	0.02806	0.00643	0.088	3.17E-04	0.01277	0	0.00494	0.088	2.58E-04	8.07E-05	3.86E-04	0.00102
0.092	0.01233	0.02671	0.00532	0.092	1.49E-04	0.0113	0	0.00674	0.092	0	5.65E-04	0	0.00187
0.096	0.01125	0.02503	0.00423	0.096	0	0.00983	2.66E-04	0.00891	0.096	5.72E-05	0.0011	0	0.00285
0.1	0.01016	0.02334	0.00334	0.1	1.12E-04	0.00835	7.99E-04	0.01155	0.1	2.86E-04	0.00194	3.51E-05	0.00411
0.104	0.00902	0.02183	0.00248	0.104	3.73E-04	0.00712	0.0016	0.01466	0.104	6.30E-04	0.00309	3.51E-04	0.00591
0.108	0.00788	0.02048	0.00181	0.108	7.08E-04	0.00614	0.00293	0.01848	0.108	0.00155	0.00471	0.00102	0.00801
0.112	0.00677	0.01913	0.00107	0.112	0.00123	0.00516	0.00453	0.02298	0.112	0.0026	0.00675	0.00204	0.01056
0.116	0.00559	0.01812	4.88E-04	0.116	0.00194	0.00442	0.00639	0.02826	0.116	0.00409	0.00931	0.00344	0.01362
0.12	0.00451	0.01677	2.32E-05	0.12	0.00293	0.00393	0.00906	0.03432	0.12	0.00615	0.01232	0.00541	0.01732
0.124	0.00368	0.0161	0	0.124	0.00415	0.00418	0.01199	0.04143	0.124	0.0087	0.01603	0.00776	0.0216
0.128	0.00268	0.01525	4.64E-05	0.128	0.00576	0.00442	0.01545	0.04957	0.128	0.01168	0.02033	0.01078	0.02656
0.132	0.00194	0.01475	3.02E-04	0.132	0.00756	0.00491	0.01945	0.05888	0.132	0.0152	0.02531	0.01454	0.03202
0.136	0.0012	0.01441	7.43E-04	0.136	0.00971	0.0059	0.02451	0.06953	0.136	0.01943	0.03101	0.01893	0.03854
0.14	6.28E-04	0.0139	0.00158	0.14	0.01245	0.00737	0.02983	0.08165	0.14	0.02427	0.03752	0.02419	0.04571
0.144	1.71E-04	0.01407	0.00265	0.144	0.01545	0.00933	0.03596	0.09488	0.144	0.02999	0.0446	0.03034	0.05379
0.148	0	0.01424	0.00399	0.148	0.01887	0.01179	0.04289	0.10963	0.148	0.0364	0.05261	0.03729	0.06272

0.152	2.85E-05	0.01458	0.00548	0.152	0.02297	0.01474	0.05088	0.12593	0.152	0.04359	0.06149	0.04554	0.07267
0.156	6.28E-04	0.01508	0.00762	0.156	0.02754	0.01842	0.05967	0.14385	0.156	0.05157	0.0712	0.05495	0.08347
0.16	7.42E-04	0.0161	0.01006	0.16	0.03281	0.0226	0.06926	0.16357	0.16	0.06059	0.08198	0.06563	0.09522
0.164	0.0012	0.01744	0.01277	0.164	0.03859	0.02751	0.07991	0.18469	0.164	0.0704	0.09355	0.07732	0.10765
0.168	0.00166	0.01896	0.01633	0.168	0.04529	0.03267	0.0919	0.20742	0.168	0.08145	0.10619	0.09056	0.12134
0.172	0.00245	0.02098	0.02053	0.172	0.05275	0.03857	0.10522	0.23236	0.172	0.09358	0.12018	0.10534	0.13591
0.176	0.00363	0.02334	0.02531	0.176	0.06113	0.0452	0.11961	0.25932	0.176	0.10709	0.13497	0.12167	0.15166
0.18	0.00494	0.02654	0.03119	0.18	0.07045	0.05208	0.13639	0.28882	0.18	0.1224	0.15165	0.13979	0.16827
0.184	0.00662	0.03059	0.03806	0.184	0.08125	0.05994	0.15397	0.32112	0.184	0.13972	0.16994	0.16015	0.18623
0.188	0.00868	0.03548	0.04642	0.188	0.09374	0.06853	0.17448	0.3559	0.188	0.15918	0.19011	0.18259	0.20515
0.192	0.01122	0.04104	0.05641	0.192	0.10808	0.07811	0.19686	0.39472	0.192	0.18121	0.21244	0.20777	0.22563
0.196	0.01407	0.04812	0.06826	0.196	0.12429	0.08843	0.22163	0.43634	0.196	0.20554	0.23691	0.23568	0.2476
0.2	0.01756	0.05671	0.08242	0.2	0.1433	0.09998	0.24987	0.4823	0.2	0.23301	0.26381	0.26753	0.27157
0.204	0.02172	0.06666	0.09868	0.204	0.1651	0.11299	0.28103	0.53137	0.204	0.26335	0.29313	0.30335	0.29738
0.208	0.02632	0.07862	0.11795	0.208	0.18969	0.12675	0.31486	0.58416	0.208	0.29626	0.32406	0.34373	0.32523
0.212	0.03171	0.0921	0.13978	0.212	0.21708	0.14247	0.35162	0.63944	0.212	0.33089	0.35688	0.38902	0.35477
0.216	0.03779	0.10744	0.1644	0.216	0.24727	0.15893	0.39078	0.69627	0.216	0.36724	0.3905	0.43924	0.38669
0.22	0.04462	0.12413	0.19134	0.22	0.27987	0.17711	0.43154	0.75373	0.22	0.40416	0.42412	0.49436	0.41997
0.224	0.0519	0.14216	0.22014	0.224	0.31415	0.19627	0.47283	0.80963	0.224	0.44022	0.4564	0.55406	0.45461
0.228	0.05955	0.16036	0.2501	0.228	0.34844	0.21567	0.51305	0.86118	0.228	0.47399	0.48599	0.61691	0.48925
0.232	0.06734	0.17873	0.28075	0.232	0.38198	0.23532	0.55141	0.90745	0.232	0.50432	0.51208	0.68257	0.52389
0.236	0.07536	0.19643	0.31095	0.236	0.41365	0.25448	0.58551	0.94658	0.236	0.52979	0.53306	0.74788	0.55751
0.24	0.08301	0.21261	0.33905	0.24	0.44178	0.27241	0.61428	0.97578	0.24	0.54925	0.54785	0.81039	0.58875
0.244	0.09026	0.22643	0.36436	0.244	0.4647	0.28789	0.63559	0.99379	0.244	0.56185	0.55565	0.86762	0.61694
0.248	0.09654	0.23721	0.38526	0.248	0.48184	0.30115	0.64944	1	0.248	0.567	0.55619	0.91713	0.64105
0.252	0.10225	0.24497	0.40175	0.252	0.49228	0.31123	0.6537	0.99472	0.252	0.56413	0.55	0.95541	0.66041
0.256	0.10682	0.24884	0.41197	0.256	0.49544	0.31737	0.64917	0.97795	0.256	0.5544	0.53682	0.98034	0.67365
0.26	0.11024	0.24867	0.41592	0.26	0.49135	0.31982	0.63506	0.95062	0.26	0.53809	0.51773	0.99052	0.68044
0.264	0.11281	0.24497	0.4136	0.264	0.48054	0.31884	0.61375	0.91491	0.264	0.5172	0.49432	0.98595	0.6818
0.268	0.11395	0.23772	0.40523	0.268	0.46358	0.31417	0.58551	0.87236	0.268	0.49144	0.46716	0.96594	0.67671
0.272	0.11424	0.2271	0.3913	0.272	0.44123	0.30607	0.55168	0.82516	0.272	0.4634	0.43784	0.93223	0.66584
0.276	0.11395	0.21412	0.37272	0.276	0.41533	0.29501	0.51492	0.77609	0.276	0.43363	0.40691	0.88693	0.65022
0.28	0.11253	0.19912	0.35043	0.28	0.38645	0.28126	0.47629	0.72609	0.28	0.40301	0.37624	0.83356	0.63052
0.284	0.11053	0.18328	0.32604	0.284	0.35626	0.26603	0.4374	0.67795	0.284	0.37325	0.34666	0.77527	0.60845
0.288	0.10796	0.1666	0.2998	0.288	0.32571	0.24932	0.39984	0.6323	0.288	0.34406	0.31815	0.71347	0.58434
0.292	0.10453	0.15008	0.27332	0.292	0.29515	0.23139	0.36414	0.58975	0.292	0.31658	0.29125	0.65238	0.55853
0.296	0.10054	0.13373	0.24708	0.296	0.26608	0.21346	0.33111	0.55155	0.296	0.29082	0.26704	0.59338	0.53374
0.3	0.09625	0.11856	0.22177	0.3	0.2387	0.19528	0.30101	0.51739	0.3	0.26736	0.24471	0.53791	0.50929
0.304	0.0914	0.10424	0.19738	0.304	0.21317	0.1776	0.27357	0.48727	0.304	0.24561	0.22454	0.48664	0.48552
0.308	0.08626	0.09092	0.17485	0.308	0.18988	0.16016	0.24933	0.46087	0.308	0.22586	0.20652	0.44064	0.46344
0.312	0.08055	0.07862	0.15418	0.312	0.16864	0.14345	0.22749	0.43758	0.312	0.20812	0.19038	0.39956	0.44273
0.316	0.07485	0.06784	0.13537	0.316	0.14963	0.12773	0.20858	0.41739	0.316	0.19238	0.17613	0.36409	0.42405
0.32	0.06894	0.05823	0.11842	0.32	0.13287	0.11299	0.1918	0.39969	0.32	0.17864	0.16348	0.33284	0.40673
0.324	0.06291	0.0493	0.10309	0.324	0.11815	0.09924	0.17688	0.38447	0.324	0.16605	0.15246	0.30616	0.39111
0.328	0.05718	0.04154	0.08939	0.328	0.1051	0.08671	0.16383	0.37112	0.328	0.15517	0.1425	0.28263	0.37719
0.332	0.05141	0.03463	0.07731	0.332	0.09374	0.07517	0.15237	0.35963	0.332	0.14601	0.1339	0.26261	0.36428
0.336	0.04604	0.02874	0.06663	0.336	0.08386	0.06485	0.14225	0.34969	0.336	0.13771	0.12637	0.24541	0.35274
0.34	0.04079	0.02351	0.05688	0.34	0.07529	0.05551	0.13346	0.34068	0.34	0.13056	0.11991	0.2307	0.34255
0.344	0.03594	0.01896	0.04851	0.344	0.06784	0.04692	0.12573	0.33323	0.344	0.12438	0.11399	0.21813	0.33304
0.348	0.03137	0.01508	0.04178	0.348	0.0615	0.03955	0.11881	0.32671	0.348	0.11917	0.10942	0.20752	0.32489
0.352	0.02732	0.01154	0.03523	0.352	0.0561	0.03292	0.11295	0.3205	0.352	0.11485	0.10538	0.19843	0.31742
0.356	0.02346	0.00885	0.03003	0.356	0.05163	0.02677	0.10762	0.31553	0.356	0.11116	0.10189	0.1906	0.31063
0.36	0.02015	0.00632	0.02538	0.36	0.0479	0.02186	0.10309	0.31118	0.36	0.10815	0.0992	0.18431	0.30485
0.364	0.01718	0.00447	0.02164	0.364	0.04492	0.01769	0.09963	0.30807	0.364	0.106	0.09732	0.17961	0.3001
0.368	0.01467	0.00295	0.01842	0.368	0.04231	0.01376	0.0967	0.30466	0.368	0.10429	0.0957	0.17546	0.29568
0.372	0.0123	0.00177	0.01561	0.372	0.04026	0.01056	0.09403	0.30217	0.372	0.10303	0.09463	0.17223	0.29161
0.376	0.01016	6.74E-04	0.0134	0.376	0.03859	0.00786	0.0919	0.30031	0.376	0.10226	0.09382	0.16988	0.28821
0.38	0.00845	4.38E-04	0.01184	0.38	0.03747	0.00565	0.0903	0.29907	0.38	0.10183	0.09382	0.16848	0.2855
0.384	0.00714	1.01E-04	0.01052	0.384	0.03672	0.00393	0.08924	0.29814	0.384	0.10191	0.09409	0.16763	0.28312
0.388	0.00588	0	0.00952	0.388	0.03616	0.00246	0.08844	0.29752	0.388	0.10237	0.09463	0.16732	0.28142
0.392	0.00491	8.43E-05	0.00889	0.392	0.03598	0.00147	0.08817	0.29783	0.392	0.10331	0.0957	0.16781	0.28006
0.396	0.00414	6.40E-04	0.00864	0.396	0.03616	7.37E-04	0.08817	0.29845	0.396	0.10454	0.09678	0.16886	0.27904
0.4	0.00368	0.0013	0.00866	0.4	0.03654	4.91E-04	0.08871	0.29938	0.4	0.10595	0.09839	0.17044	0.2787
0.404	0.0034	0.00211	0.00889	0.404	0.0371	0	0.08924	0.30062	0.404	0.10775	0.10027	0.17234	0.27836
0.408	0.00317	0.00312	0.00936	0.408	0.03803	2.46E-04	0.0903	0.30248	0.408	0.1097	0.10243	0.1749	0.2787
0.412	0.00311	0.00463	0.01008	0.412	0.03915	4.91E-04	0.09164	0.30466	0.412	0.11196	0.10485	0.17767	0.27972
0.416	0.00323	0.00615	0.01105	0.416	0.04045	7.37E-04	0.09323	0.30714	0.416	0.11439	0.10727	0.18066	0.2804
0.42	0.00343	0.00767	0.01229	0.42	0.04175	0.00147	0.0951	0.31025	0.42	0.11708	0.10996	0.18424	0.28176
0.424	0.00388	0.00969	0.01356	0.424	0.04343	0.00246	0.09723	0.31335	0.424	0.12011	0.11292	0.18814	0.2838
0.428	0.00442	0.01171	0.01533	0.428	0.04511	0.00319	0.09989	0.31739	0.428	0.1234	0.11587	0.19214	0.2855
0.432	0.00505	0.01407	0.01709	0.432	0.04716	0.00442	0.10229	0.32112	0.432	0.12684	0.1191	0.19639	0.28821
0.436	0.00585	0.0166	0.01895	0.436	0.04921	0.00565	0.10522	0.32547	0.436	0.13056	0.1226	0.20088	0.29059
0.44	0.00645	0.0193	0.0212	0.44	0.05163	0.00688	0.10868	0.33012	0.44	0.13428	0.1261	0.20541	0.29365
0.444	0.00728	0.02216	0.02353	0.444	0.05405	0.0086	0.11215	0.33509	0.444	0.13857	0.13013	0.21015	0.2967
0.448	0.00819	0.02536	0.02603	0.448	0.05666	0.01007	0.11588	0.34068	0.448	0.14287	0.13417	0.21507	0.3001
0.452	0.00896	0.02823	0.02884	0.452	0.05927	0.01179	0.11987	0.34627	0.452	0.14744	0.1382	0.22002	0.30383
0.456	0.00988	0.03177	0.03175	0.456	0.06225	0.01351	0.1244	0.35217	0.456	0.15231	0.14277	0.22518	0.30791
0.46	0.01082	0.03531	0.03504	0.46	0.06523	0.01548	0.1292	0.3587	0.46	0.15746	0.14735	0.23052	0.31198
0.464	0.01165	0.03885	0.03832	0.464	0.0684	0.01769	0.13426	0.36491	0.464	0.16261	0.15219	0.23593	0.3164
0.468	0.01267	0.04255	0.04178	0.468	0.07175	0.0199	0.13958	0.37174	0.468	0.16834	0.1573	0.24162	0.32081
0.472	0.01344	0.04626	0.04573	0.472	0.07511	0.02235	0.14518	0.37888	0.472	0.17406	0.16241	0.24716	0.32591
0.476	0.01442	0.04997	0.04944	0.476	0.07827	0.02506	0.15077	0.38602	0.476	0.17978	0.16779	0.25278	0.33066
0.48	0.01536	0.05385	0.05362	0.48	0.08181	0.02751	0.1569	0.39317	0.48	0.18579	0.1729	0.2591	0.33576
0.484	0.01613	0.05739	0.0578	0.484	0.08517	0.03021	0.16303	0.40031	0.484	0.19152	0.17828	0.26472	0.34051
0.488	0.01701	0.06126	0.06198	0.488	0.08852	0.03341	0.16915	0.40745	0.488	0.19724	0.18366	0.27104	0.34594
0.492	0.01807	0.0648	0.06663	0.492	0.09187	0.0366	0.17555	0.41491	0.492	0.20296	0.18877	0.27701	0.35104
0.496	0.01898	0.06834	0.07104	0.496	0.09504	0.03979	0.18194	0.42205	0.496	0.20869	0.19388	0.28333	0.35647
0.5	0.02001	0.07188	0.07545	0.5	0.09821	0.04299	0.1878	0.42888	0.5	0.21413	0.19872	0.2893	0.36123
0.504	0.02098	0.07508	0.07987	0.504	0.10119	0.04667	0.19366	0.4354	0.504	0.21928	0.20329	0.29562	0.36632
0.508	0.02221	0.07828	0.08428	0.508	0.10417	0.05036	0.19952	0.44193	0.508	0.22414	0.2076	0.30124	0.37107
0.512	0.02338	0.08149	0.08846	0.512	0.10697	0.05429	0.20485	0.44814	0.512	0.22872	0.21163	0.30721	0.37583
0.516	0.02463	0.08418	0.09264	0.516	0.10958	0.05797	0.20964	0.45342	0.516	0.23244	0.21486	0.31248	0.3799
0.52	0.02603	0.08739	0.09682	0.52	0.11218	0.06239	0.21417	0.4587	0.52	0.23645	0.21835	0.31774	0.38432
0.524	0.02752	0.09025	0.10077	0.524	0.11479	0.06657	0.21843	0.46335	0.524	0.23988	0.22131	0.32231	0.38771
0.528	0.0292	0.09312	0.10495	0.528	0.1174	0.07099	0.22216	0.46801	0.528	0.24275	0.22373	0.32687	0.39111
0.532	0.03097	0.09598	0.1089	0.532	0.11982	0.07566	0.22589	0.47174	0.532	0.24561	0.22562	0.33038	0.39417
0.536	0.03283	0.09901	0.11285	0.536	0.12243	0.08057	0.22909	0.47516	0.536	0.24818	0.22777	0.3339	0.39688
0.54	0.03508	0.10205	0.11679	0.54	0.12523	0.08548	0.23202	0.47857	0.54	0.25019	0.22938	0.33706	0.39892
0.544	0.03748	0.10525	0.12097	0.544	0.12802	0.09113	0.23495	0.48137	0.544	0.25248	0.231	0.33951	0.40096
0.548	0.03999	0.10845	0.12515	0.548	0.13082	0.09654	0.23761	0.48385	0.548	0.25419	0.23261	0.34127	0.40232
0.552	0.04285	0.11199	0.1298	0.552	0.13417	0.10243	0.24054	0.48602	0.552	0.2562	0.23422	0.34302	0.40333
0.556	0.04613	0.11604	0.13444	0.556	0.13752	0.10906	0.24374	0.4882	0.556	0.25791	0.23584	0.34478	0.40401
0.56	0.04944	0.12025	0.13932	0.56	0.14144	0.1157	0.24667	0.49006	0.56	0.25992	0.23745	0.34583	0.40469

0.564	0.05335	0.1248	0.14443	0.564	0.14535	0.12257	0.24987	0.49193	0.564	0.26192	0.23907	0.34689	0.40503
0.568	0.0572	0.12986	0.15047	0.568	0.15001	0.13019	0.2536	0.49348	0.568	0.26421	0.24122	0.34794	0.40469
0.572	0.06172	0.13542	0.15697	0.572	0.15504	0.1383	0.25759	0.49565	0.572	0.26678	0.24364	0.34935	0.40503
0.576	0.0664	0.14132	0.16371	0.576	0.16044	0.14665	0.26212	0.49783	0.576	0.26965	0.24606	0.3504	0.40503
0.58	0.07116	0.14738	0.17091	0.58	0.16603	0.15549	0.26665	0.49938	0.58	0.27251	0.24875	0.35145	0.40469
0.584	0.0767	0.15412	0.17903	0.584	0.17218	0.16458	0.27198	0.50186	0.584	0.27566	0.25171	0.35321	0.40469
0.588	0.08255	0.1612	0.18763	0.588	0.1787	0.17391	0.27757	0.50435	0.588	0.27938	0.2552	0.35496	0.40469
0.592	0.08855	0.16862	0.19668	0.592	0.18559	0.18374	0.2837	0.50683	0.592	0.28338	0.25897	0.35707	0.40503
0.596	0.09511	0.17654	0.20597	0.596	0.19249	0.19356	0.29036	0.50994	0.596	0.28739	0.26301	0.35953	0.40537
0.6	0.10196	0.18446	0.21596	0.6	0.19975	0.20388	0.29728	0.51304	0.6	0.29197	0.26758	0.36269	0.40605
0.604	0.10939	0.19272	0.22618	0.604	0.20739	0.2142	0.30474	0.51646	0.604	0.29712	0.27215	0.36585	0.40707
0.608	0.11709	0.20131	0.23686	0.608	0.21503	0.22451	0.31247	0.52019	0.608	0.30227	0.27726	0.36971	0.40877
0.612	0.12509	0.20957	0.24708	0.612	0.22249	0.23459	0.32019	0.52422	0.612	0.30742	0.28237	0.37357	0.41047
0.616	0.13365	0.21817	0.25799	0.616	0.22994	0.24466	0.32845	0.52857	0.616	0.31343	0.28802	0.37814	0.4125
0.62	0.1425	0.22626	0.26868	0.62	0.23702	0.25424	0.33644	0.53323	0.62	0.31887	0.29394	0.3827	0.41488
0.624	0.15163	0.23401	0.2789	0.624	0.24391	0.26357	0.3447	0.53758	0.624	0.32488	0.29985	0.38797	0.41794
0.628	0.16134	0.24176	0.28865	0.628	0.25025	0.27217	0.35269	0.54224	0.628	0.33061	0.3055	0.39324	0.42099
0.632	0.17104	0.2485	0.29771	0.632	0.25621	0.28028	0.36068	0.54689	0.632	0.33633	0.31142	0.3985	0.42439
0.636	0.18075	0.25525	0.3063	0.636	0.26161	0.28789	0.36841	0.55186	0.636	0.34234	0.31734	0.40447	0.42846
0.64	0.19103	0.26114	0.3142	0.64	0.26646	0.29452	0.37587	0.55652	0.64	0.34778	0.32352	0.41044	0.43288
0.644	0.2013	0.2662	0.3207	0.644	0.27018	0.30017	0.38279	0.56087	0.644	0.35321	0.3289	0.41571	0.43695
0.648	0.21158	0.27058	0.32651	0.648	0.27335	0.30484	0.38945	0.56553	0.648	0.35808	0.33428	0.42168	0.44137
0.652	0.22157	0.27429	0.33115	0.652	0.27577	0.30877	0.39584	0.57019	0.652	0.36266	0.33966	0.42765	0.44612
0.656	0.23156	0.27716	0.33487	0.656	0.27764	0.31172	0.40144	0.57422	0.656	0.36695	0.3445	0.43327	0.45054
0.66	0.24127	0.27935	0.33742	0.66	0.27838	0.31393	0.40677	0.57826	0.66	0.37096	0.34881	0.43853	0.45529
0.664	0.25097	0.28069	0.33928	0.664	0.27857	0.31466	0.41129	0.58168	0.664	0.37411	0.35311	0.44345	0.45971
0.668	0.26011	0.28171	0.33998	0.668	0.27819	0.31491	0.41556	0.58509	0.668	0.37697	0.35688	0.44837	0.46446
0.672	0.26895	0.28187	0.33998	0.672	0.27745	0.31466	0.41955	0.5882	0.672	0.37954	0.36064	0.45293	0.46888
0.676	0.27723	0.2812	0.33881	0.676	0.27559	0.31319	0.42222	0.59037	0.676	0.38097	0.36333	0.45679	0.47261
0.68	0.28522	0.28002	0.33696	0.68	0.27372	0.31098	0.42461	0.59255	0.68	0.38241	0.36575	0.4603	0.47669
0.684	0.29208	0.27884	0.3344	0.684	0.27149	0.30828	0.42648	0.59441	0.684	0.38326	0.36791	0.46311	0.48042
0.688	0.29893	0.27665	0.33115	0.688	0.26888	0.30484	0.42781	0.59534	0.688	0.38326	0.36925	0.46557	0.48348
0.692	0.30464	0.27429	0.32767	0.692	0.2659	0.30115	0.42861	0.59596	0.692	0.38326	0.37033	0.46768	0.4862
0.696	0.31006	0.27159	0.32372	0.696	0.2631	0.29673	0.42888	0.59627	0.696	0.38241	0.37086	0.46908	0.48857
0.7	0.31434	0.2689	0.31931	0.7	0.26012	0.29256	0.42861	0.59596	0.7	0.38155	0.37113	0.47014	0.49095
0.704	0.31805	0.26603	0.31466	0.704	0.25714	0.28789	0.42808	0.59534	0.704	0.38012	0.37086	0.47084	0.49265
0.708	0.32091	0.26283	0.30978	0.708	0.25397	0.28273	0.42674	0.5941	0.708	0.3784	0.37033	0.47049	0.49401
0.712	0.32348	0.2598	0.30491	0.712	0.25118	0.27757	0.42541	0.59224	0.712	0.3764	0.36952	0.47049	0.49502
0.716	0.32519	0.25676	0.30003	0.716	0.24838	0.27266	0.42381	0.59037	0.716	0.37439	0.36844	0.46978	0.4957
0.72	0.32633	0.25373	0.29492	0.72	0.24578	0.26726	0.42195	0.5882	0.72	0.3721	0.3671	0.46873	0.49638
0.724	0.3269	0.2507	0.28981	0.724	0.24317	0.2621	0.41955	0.58509	0.724	0.36981	0.36548	0.46698	0.49638
0.728	0.32719	0.24783	0.28493	0.728	0.24093	0.25669	0.41716	0.58199	0.728	0.36752	0.36387	0.46522	0.49638
0.732	0.3269	0.2453	0.28052	0.732	0.23888	0.25178	0.41476	0.57857	0.732	0.36523	0.36199	0.46382	0.49604
0.736	0.32633	0.24277	0.27588	0.736	0.23702	0.24687	0.41236	0.57516	0.736	0.36294	0.36037	0.46171	0.49604
0.74	0.32519	0.24041	0.27146	0.74	0.23534	0.2422	0.4097	0.57112	0.74	0.36037	0.35849	0.4596	0.49536
0.744	0.32348	0.23839	0.26752	0.744	0.23404	0.23753	0.4073	0.56739	0.744	0.35837	0.35661	0.45785	0.49435
0.748	0.32176	0.23654	0.2638	0.748	0.23292	0.23311	0.4049	0.56335	0.748	0.35665	0.35499	0.45574	0.49401
0.752	0.32005	0.23468	0.26032	0.752	0.23199	0.22918	0.40277	0.55901	0.752	0.35493	0.35338	0.45398	0.49333
0.756	0.31777	0.23334	0.25707	0.756	0.23106	0.22525	0.40037	0.55497	0.756	0.35321	0.35177	0.45188	0.49265
0.76	0.31548	0.23199	0.25428	0.76	0.2305	0.22181	0.39851	0.55062	0.76	0.35178	0.35042	0.45012	0.49197
0.764	0.3132	0.23098	0.25172	0.764	0.23031	0.21837	0.39691	0.54658	0.764	0.35092	0.34908	0.44872	0.49163
0.768	0.31063	0.23047	0.24963	0.768	0.23012	0.21543	0.39531	0.54255	0.768	0.34978	0.34827	0.44731	0.49129
0.772	0.30806	0.22963	0.24801	0.772	0.23012	0.21272	0.39371	0.53851	0.772	0.34921	0.34719	0.44591	0.49061
0.776	0.30606	0.2298	0.24685	0.776	0.23087	0.21076	0.39345	0.5354	0.776	0.34921	0.34746	0.44556	0.49095
0.78	0.30321	0.22946	0.24545	0.78	0.23124	0.20879	0.39265	0.53168	0.78	0.34892	0.34719	0.4445	0.49095
0.784	0.30092	0.22946	0.24476	0.784	0.23199	0.20683	0.39212	0.52826	0.784	0.34863	0.34666	0.44415	0.49129
0.788	0.29836	0.22963	0.24406	0.788	0.23255	0.20511	0.39158	0.52484	0.788	0.34863	0.34666	0.44345	0.49129
0.792	0.29579	0.23013	0.2436	0.792	0.23348	0.20388	0.39132	0.52143	0.792	0.34892	0.34666	0.44345	0.49163
0.796	0.2935	0.23047	0.24336	0.796	0.2346	0.2029	0.39132	0.51863	0.796	0.34949	0.34719	0.44345	0.49231
0.8	0.2915	0.23148	0.2436	0.8	0.2359	0.20241	0.39158	0.51584	0.8	0.34978	0.34773	0.4438	0.49299
0.804	0.28922	0.23199	0.2436	0.804	0.23702	0.20142	0.39185	0.51304	0.804	0.35035	0.34827	0.44415	0.49367
0.808	0.28751	0.23317	0.24429	0.808	0.2387	0.20118	0.39238	0.51087	0.808	0.35092	0.34908	0.44485	0.49435
0.812	0.28608	0.23435	0.24522	0.812	0.24037	0.20142	0.39345	0.50839	0.812	0.35207	0.34988	0.44556	0.4957
0.816	0.28465	0.23586	0.24592	0.816	0.24205	0.20118	0.39425	0.50652	0.816	0.35264	0.35069	0.44661	0.49672
0.82	0.28351	0.23704	0.24708	0.82	0.24391	0.20167	0.39505	0.50435	0.82	0.3535	0.3515	0.44731	0.49774
0.824	0.28208	0.23822	0.24778	0.824	0.24559	0.20167	0.39558	0.50217	0.824	0.35379	0.35204	0.44801	0.49842
0.828	0.28151	0.23991	0.2494	0.828	0.24782	0.20216	0.39664	0.50062	0.828	0.35493	0.35311	0.44942	0.49944
0.832	0.28094	0.24159	0.2508	0.832	0.24987	0.2029	0.39771	0.49876	0.832	0.35579	0.35392	0.45082	0.5008
0.836	0.28094	0.24328	0.25242	0.836	0.25192	0.20364	0.39877	0.4972	0.836	0.35665	0.35499	0.45188	0.50148
0.84	0.28066	0.24497	0.25405	0.84	0.25416	0.20462	0.40011	0.49596	0.84	0.35751	0.3558	0.45328	0.5025
0.844	0.28123	0.24716	0.2559	0.844	0.25658	0.2056	0.40117	0.49472	0.844	0.35837	0.35688	0.45469	0.50351
0.848	0.2818	0.24867	0.25776	0.848	0.25882	0.20658	0.40224	0.49348	0.848	0.35922	0.35768	0.45609	0.50419
0.852	0.28237	0.25053	0.25939	0.852	0.26087	0.20757	0.4033	0.49224	0.852	0.3598	0.35849	0.45749	0.50487
0.856	0.28323	0.25238	0.26125	0.856	0.2631	0.20855	0.4041	0.49099	0.856	0.36037	0.3593	0.4589	0.50521
0.86	0.28437	0.2544	0.2631	0.86	0.26534	0.21002	0.40517	0.48975	0.86	0.36094	0.35984	0.45995	0.50589
0.864	0.2858	0.25609	0.26496	0.864	0.26739	0.21125	0.40597	0.48882	0.864	0.36151	0.36064	0.46136	0.50623
0.868	0.28722	0.25761	0.26659	0.868	0.26925	0.21248	0.4065	0.48758	0.868	0.3618	0.36118	0.46276	0.50623
0.872	0.28865	0.25929	0.26868	0.872	0.27111	0.21371	0.4073	0.48665	0.872	0.36237	0.36172	0.46382	0.50623
0.876	0.29065	0.26148	0.27054	0.876	0.27298	0.21518	0.4081	0.48571	0.876	0.36294	0.36226	0.46522	0.50657
0.88	0.29265	0.263	0.27216	0.88	0.27465	0.21641	0.40836	0.48447	0.88	0.36352	0.3628	0.46662	0.50623
0.884	0.29436	0.26435	0.27379	0.884	0.27596	0.21764	0.4089	0.48323	0.884	0.36352	0.36306	0.46733	0.50555
0.888	0.29636	0.26569	0.27541	0.888	0.27726	0.21887	0.4089	0.4823	0.888	0.36352	0.36333	0.46838	0.50487
0.892	0.29864	0.26738	0.27704	0.892	0.27857	0.22009	0.40943	0.48106	0.892	0.3638	0.3636	0.46943	0.50419
0.896	0.30064	0.26856	0.27843	0.896	0.27969	0.22132	0.40943	0.48012	0.896	0.36409	0.36387	0.47049	0.50385
0.9	0.30264	0.26991	0.27983	0.9	0.28062	0.22255	0.40943	0.47857	0.9	0.36409	0.36387	0.47084	0.5025
0.904	0.30492	0.27126	0.28145	0.904	0.28174	0.22353	0.4097	0.47764	0.904	0.36438	0.36414	0.47189	0.50182
0.908	0.3072	0.2726	0.28284	0.908	0.28267	0.22476	0.4097	0.4764	0.908	0.36438	0.36441	0.47259	0.5008
0.912	0.3092	0.27378	0.28424	0.912	0.2836	0.22599	0.4097	0.47516	0.912	0.36466	0.36468	0.4733	0.49978
0.916	0.31149	0.27513	0.2854	0.916	0.28416	0.22722	0.40943	0.47422	0.916	0.36495	0.36495	0.474	0.49876
0.92	0.31348	0.27648	0.28656	0.92	0.28509	0.2282	0.40916	0.47267	0.92	0.36495	0.36522	0.4747	0.49774
0.924	0.31577	0.27783	0.28795	0.924	0.28583	0.22943	0.40916	0.47174	0.924	0.36523	0.36548	0.4754	0.49672
0.928	0.31805	0.27918	0.28935	0.928	0.28677	0.23066	0.4089	0.4705	0.928	0.36581	0.36575	0.47646	0.4957
0.932	0.32034	0.28053	0.29028	0.932	0.28751	0.23164	0.40863	0.46925	0.932	0.36581	0.36602	0.47716	0.49469
0.936	0.32233	0.28204	0.29167	0.936	0.28844	0.23287	0.40863	0.46832	0.936	0.36666	0.36656	0.47786	0.49401
0.94	0.32462	0.28373	0.2933	0.94	0.28975	0.23459	0.40863	0.46739	0.94	0.36752	0.36737	0.47856	0.49299
0.944	0.32662	0.28541	0.29446	0.944	0.29068	0.23581	0.40863	0.46646	0.944	0.3681	0.36764	0.47962	0.49231
0.948	0.32861	0.2871	0.29608	0.948	0.2918	0.23753	0.40863	0.46553	0.948	0.36867	0.36871	0.48032	0.49197
0.952	0.3309	0.28912	0.29748	0.952	0.2931	0.23876	0.4089	0.4646	0.952	0.36981	0.36952	0.48137	0.49129
0.956	0.33318	0.29131	0.2991	0.956	0.29459	0.24073	0.40916	0.46398	0.956	0.37067	0.37033	0.48243	0.49095
0.96	0.33518	0.2935	0.30073	0.96	0.29608	0.24245	0.4097	0.46335	0.96	0.37182	0.3714	0.48348	0.49095
0.964	0.33718	0.29603	0.30258	0.964	0.29757	0.24392	0.40996	0.46273	0.964	0.37325	0.37248	0.48488	0.49027
0.968	0.33975	0.29856	0.30467	0.968	0.29925	0.24638	0.4105	0.46242	0.968	0.37468	0.37409	0.48629	0.49061
0.972	0.34174	0.30109	0.30677	0.972	0.3013	0.24883	0.41129	0.46211	0.972	0.37611	0.37571	0.48769	0.49095

0.976	0.34403	0.30446	0.30886	0.976	0.30335	0.25104	0.41183	0.4618	0.976	0.37783	0.37705	0.4891	0.49129
0.98	0.34631	0.30732	0.31118	0.98	0.30558	0.2535	0.41263	0.4618	0.98	0.38012	0.37866	0.49085	0.49197
0.984	0.34888	0.31052	0.31373	0.984	0.30801	0.25596	0.41369	0.46149	0.984	0.38212	0.38028	0.49226	0.49265
0.988	0.35116	0.31406	0.31652	0.988	0.31061	0.25841	0.41476	0.46149	0.988	0.38412	0.38243	0.49436	0.49333
0.992	0.35373	0.3176	0.31931	0.992	0.31341	0.26136	0.41582	0.4618	0.992	0.3867	0.38431	0.49647	0.49435
0.996	0.3563	0.32114	0.32186	0.996	0.31602	0.26406	0.41689	0.4618	0.996	0.38899	0.3862	0.49823	0.49536
1	0.35887	0.32519	0.32511	1	0.319	0.26701	0.41822	0.46242	1	0.39156	0.38835	0.50033	0.49672
1.004	0.36173	0.32906	0.32836	1.004	0.32217	0.2702	0.42009	0.46304	1.004	0.39443	0.39104	0.50279	0.49808
1.008	0.36458	0.33328	0.33185	1.008	0.32533	0.2734	0.42195	0.46366	1.008	0.39729	0.39346	0.50525	0.49978
1.012	0.36744	0.33715	0.3351	1.012	0.3285	0.27708	0.42355	0.46429	1.012	0.40015	0.39588	0.50736	0.50114
1.016	0.37058	0.3412	0.33858	1.016	0.33186	0.28052	0.42541	0.46522	1.016	0.4033	0.39884	0.51017	0.50318
1.02	0.374	0.34575	0.3423	1.02	0.3354	0.28445	0.42808	0.46615	1.02	0.40673	0.4018	0.51297	0.50521
1.024	0.37743	0.35013	0.34625	1.024	0.33912	0.28814	0.43021	0.46739	1.024	0.41017	0.40502	0.51578	0.50725
1.028	0.38085	0.35434	0.35019	1.028	0.34248	0.29182	0.43234	0.46832	1.028	0.41331	0.40798	0.51859	0.50929
1.032	0.38485	0.35889	0.35437	1.032	0.34639	0.29624	0.43527	0.46988	1.032	0.41732	0.41148	0.52175	0.512
1.036	0.38827	0.36311	0.35855	1.036	0.35011	0.30066	0.4382	0.47143	1.036	0.42104	0.41471	0.52491	0.51472
1.04	0.39227	0.36783	0.36297	1.04	0.35421	0.30484	0.44113	0.47298	1.04	0.42505	0.4182	0.52842	0.5171
1.044	0.39627	0.37204	0.36738	1.044	0.35813	0.30926	0.44433	0.47453	1.044	0.42877	0.42197	0.53194	0.52015
1.048	0.40026	0.37659	0.37179	1.048	0.36223	0.31393	0.44752	0.4764	1.048	0.43277	0.42574	0.53545	0.52321
1.052	0.40454	0.38097	0.37644	1.052	0.36651	0.31909	0.45099	0.47826	1.052	0.43735	0.42977	0.53931	0.52627
1.056	0.40883	0.38586	0.38131	1.056	0.37098	0.324	0.45471	0.48043	1.056	0.44165	0.43407	0.54317	0.53
1.06	0.41311	0.39041	0.38619	1.06	0.37508	0.3294	0.45844	0.48261	1.06	0.44594	0.43811	0.54703	0.53306
1.064	0.41739	0.39547	0.39107	1.064	0.37974	0.33456	0.46244	0.48478	1.064	0.45052	0.44268	0.55125	0.53679
1.068	0.42224	0.40052	0.39618	1.068	0.3844	0.34021	0.4667	0.48727	1.068	0.4551	0.44725	0.55511	0.54087
1.072	0.42681	0.40507	0.40129	1.072	0.38943	0.34611	0.47096	0.49006	1.072	0.45996	0.45183	0.55932	0.54461
1.076	0.43138	0.40979	0.40663	1.076	0.39427	0.35151	0.47549	0.49255	1.076	0.46454	0.45667	0.56354	0.54868
1.08	0.43623	0.41485	0.4122	1.08	0.39949	0.35741	0.48029	0.49534	1.08	0.46969	0.46178	0.5681	0.55276
1.084	0.44137	0.42024	0.41824	1.084	0.40527	0.36404	0.48562	0.49845	1.084	0.47513	0.46716	0.57267	0.55751
1.088	0.44651	0.4258	0.42405	1.088	0.41086	0.37042	0.49068	0.50155	1.088	0.48057	0.47254	0.57758	0.56226
1.092	0.45164	0.43086	0.42985	1.092	0.41644	0.37657	0.49574	0.50466	1.092	0.48601	0.47765	0.5818	0.56736
1.096	0.45707	0.43659	0.43612	1.096	0.42241	0.38344	0.50133	0.50807	1.096	0.49173	0.4833	0.58671	0.57245
1.1	0.46249	0.44282	0.44263	1.1	0.42856	0.39032	0.50693	0.51149	1.1	0.49745	0.48895	0.59163	0.57789
1.104	0.4682	0.44872	0.44936	1.104	0.43489	0.3972	0.51279	0.51522	1.104	0.50346	0.49513	0.59654	0.58298
1.108	0.47391	0.45513	0.45586	1.108	0.4416	0.40481	0.51865	0.51894	1.108	0.50947	0.50078	0.60146	0.58841
1.112	0.4799	0.46187	0.46306	1.112	0.44831	0.41218	0.52504	0.52298	1.112	0.51577	0.50724	0.60673	0.59385
1.116	0.48647	0.46861	0.47049	1.116	0.45557	0.42004	0.5317	0.52733	1.116	0.52207	0.51369	0.61199	0.59996
1.12	0.49332	0.47586	0.47793	1.12	0.46284	0.4284	0.53863	0.53168	1.12	0.52893	0.52042	0.61726	0.60607
1.124	0.49989	0.48294	0.48536	1.124	0.4701	0.4365	0.54529	0.53602	1.124	0.53552	0.52714	0.62253	0.61185
1.128	0.50731	0.49052	0.49372	1.128	0.47793	0.44535	0.55248	0.54068	1.128	0.54267	0.53467	0.62815	0.6183
1.132	0.51473	0.49844	0.50185	1.132	0.48594	0.45419	0.55994	0.54565	1.132	0.54983	0.54193	0.63377	0.62509
1.136	0.52244	0.50636	0.51021	1.136	0.49433	0.46352	0.56793	0.55062	1.136	0.55755	0.54946	0.63938	0.63188
1.14	0.53043	0.51462	0.5188	1.14	0.50308	0.47286	0.57539	0.55528	1.14	0.56499	0.557	0.64535	0.63901
1.144	0.53899	0.52288	0.52786	1.144	0.51184	0.48244	0.58364	0.56087	1.144	0.57301	0.56507	0.65132	0.64614
1.148	0.54784	0.53147	0.53738	1.148	0.52097	0.49275	0.59217	0.56646	1.148	0.58159	0.5734	0.65764	0.65361
1.152	0.55698	0.54041	0.5469	1.152	0.53066	0.50307	0.60123	0.57236	1.152	0.59018	0.58201	0.66431	0.66109
1.156	0.5664	0.54984	0.55642	1.156	0.54016	0.51339	0.60948	0.57795	1.156	0.59876	0.59035	0.67099	0.66856
1.16	0.57667	0.55911	0.56664	1.16	0.55004	0.52444	0.61907	0.58416	1.16	0.60764	0.59949	0.67801	0.67671
1.164	0.58695	0.56923	0.57686	1.164	0.56028	0.53549	0.62813	0.59037	1.164	0.61708	0.60864	0.68503	0.68486
1.168	0.5978	0.57934	0.58708	1.168	0.57072	0.54679	0.63799	0.59627	1.168	0.62624	0.61805	0.6924	0.69301
1.172	0.60836	0.58962	0.59799	1.172	0.58134	0.55809	0.64784	0.6028	1.172	0.63597	0.62774	0.69978	0.7015
1.176	0.61978	0.60007	0.60914	1.176	0.59233	0.56988	0.65796	0.60901	1.176	0.64541	0.63769	0.7075	0.71033
1.18	0.63148	0.61085	0.62075	1.18	0.60351	0.58192	0.66862	0.61584	1.18	0.65572	0.64791	0.71523	0.7195
1.184	0.64375	0.62282	0.63306	1.184	0.61599	0.59494	0.67981	0.62298	1.184	0.66659	0.65894	0.72401	0.72934
1.188	0.65574	0.63361	0.64444	1.188	0.62736	0.60747	0.69046	0.6295	1.188	0.67718	0.66916	0.73173	0.73817
1.192	0.66859	0.64523	0.65675	1.192	0.63947	0.62049	0.70139	0.63634	1.192	0.68805	0.68019	0.74016	0.74768
1.196	0.68143	0.6567	0.66883	1.196	0.65195	0.63375	0.71231	0.64348	1.196	0.69893	0.69095	0.74894	0.75719
1.2	0.69428	0.66866	0.68114	1.2	0.66444	0.64652	0.72376	0.65	1.2	0.7098	0.70224	0.75772	0.76704
1.204	0.70712	0.68029	0.69344	1.204	0.67692	0.66003	0.73495	0.65683	1.204	0.72097	0.71354	0.76614	0.77621
1.208	0.72026	0.69276	0.70598	1.208	0.68978	0.67354	0.7464	0.66398	1.208	0.73184	0.72484	0.77527	0.78606
1.212	0.73396	0.70506	0.71899	1.212	0.70301	0.6873	0.75812	0.67112	1.212	0.74358	0.73694	0.78475	0.79624
1.216	0.74766	0.71804	0.732	1.216	0.71642	0.70081	0.77011	0.67826	1.216	0.75502	0.74824	0.79423	0.80609
1.22	0.76079	0.72967	0.74454	1.22	0.72946	0.71457	0.78157	0.68509	1.22	0.76647	0.7598	0.80336	0.8156
1.224	0.77478	0.74265	0.75754	1.224	0.74325	0.72906	0.79355	0.69224	1.224	0.77849	0.77191	0.81355	0.82579
1.228	0.78819	0.75579	0.77032	1.228	0.75704	0.74331	0.80581	0.69969	1.228	0.78994	0.78401	0.82303	0.83598
1.232	0.80161	0.76894	0.78379	1.232	0.77101	0.75731	0.81753	0.70652	1.232	0.80167	0.79612	0.83286	0.84616
1.236	0.81474	0.78107	0.79656	1.236	0.78443	0.77106	0.82898	0.71304	1.236	0.81312	0.80795	0.84234	0.85533
1.24	0.82787	0.79388	0.80933	1.24	0.79822	0.78531	0.8407	0.71957	1.24	0.82485	0.81979	0.85217	0.86518
1.244	0.841	0.80753	0.82257	1.244	0.812	0.79956	0.85242	0.7264	1.244	0.83687	0.83189	0.862	0.87503
1.248	0.85442	0.82068	0.83581	1.248	0.82616	0.81405	0.86414	0.73323	1.248	0.84889	0.84399	0.87254	0.88488
1.252	0.86641	0.83416	0.84835	1.252	0.83995	0.82781	0.87507	0.73975	1.252	0.86034	0.85583	0.88202	0.89405
1.256	0.87897	0.8468	0.86159	1.256	0.85392	0.84205	0.88652	0.74658	1.256	0.87236	0.8682	0.8922	0.9039
1.26	0.89124	0.86096	0.87482	1.26	0.86771	0.85655	0.89798	0.75311	1.26	0.88409	0.88031	0.90238	0.91306
1.264	0.90323	0.87461	0.88736	1.264	0.88131	0.87104	0.90916	0.75963	1.264	0.89554	0.89241	0.91257	0.92223
1.268	0.91436	0.88759	0.90014	1.268	0.89473	0.88504	0.91955	0.76584	1.268	0.90699	0.90424	0.92275	0.93106
1.272	0.92578	0.90073	0.91221	1.272	0.90833	0.8988	0.93021	0.77205	1.272	0.91844	0.91635	0.93223	0.93989
1.276	0.9372	0.91506	0.92499	1.276	0.92193	0.91353	0.94086	0.77826	1.276	0.92988	0.92845	0.94241	0.94906
1.28	0.94805	0.92854	0.93799	1.28	0.93572	0.92803	0.95099	0.78509	1.28	0.94162	0.94029	0.9526	0.95789
1.284	0.95832	0.94219	0.94984	1.284	0.94858	0.94178	0.96084	0.79099	1.284	0.95278	0.95239	0.96173	0.96638
1.288	0.96946	0.95702	0.96307	1.288	0.96199	0.95677	0.97176	0.79783	1.288	0.96508	0.96476	0.97156	0.97555
1.292	0.97973	0.97101	0.97538	1.292	0.97466	0.97126	0.98109	0.80404	1.292	0.97653	0.97687	0.98104	0.9837
1.296	0.99001	0.985	0.98769	1.296	0.98752	0.98575	0.99094	0.80994	1.296	0.98855	0.98843	0.99087	0.99185
1.3	1	1	1	1.3	1	1	1	0.81615	1.3	1	1	1	1

Raw table S3. Electrochemical fingerprints of *Acer palmatum* 'Matsumurae'

Acer palmatum 'Matsumurae'

**Ethanol-ABS (Spring)**				**Ethanol-ABS (Summer)**					**Ethanol-ABS (Autumn)**				
0.004	0.12541	0.05472	0.12607	0.004	0.06268	0.1251	0.02589	0.04847	0.004	0.05231	0.06817	0.03944	0.03829
0.008	0.1197	0.05089	0.12	0.008	0.05888	0.11971	0.02618	0.04537	0.008	0.04915	0.06368	0.03665	0.03549
0.012	0.11473	0.04763	0.11494	0.012	0.05581	0.11494	0.02634	0.0428	0.012	0.04641	0.06014	0.03433	0.03289
0.016	0.11081	0.04475	0.11039	0.016	0.05306	0.11079	0.0264	0.04057	0.016	0.04415	0.0568	0.03227	0.03079
0.02	0.10706	0.04207	0.10659	0.02	0.05054	0.10726	0.02668	0.0388	0.02	0.04221	0.05402	0.03058	0.02888
0.024	0.10407	0.03977	0.1033	0.024	0.04837	0.10394	0.02662	0.03713	0.024	0.04045	0.05132	0.02865	0.02715
0.028	0.10135	0.03767	0.10002	0.028	0.04644	0.10124	0.02662	0.03551	0.028	0.03897	0.04905	0.02722	0.02553
0.032	0.09891	0.03575	0.09723	0.032	0.04452	0.09834	0.02662	0.03421	0.032	0.03751	0.04689	0.02579	0.02403
0.036	0.09644	0.03383	0.09445	0.036	0.04278	0.09585	0.02672	0.03291	0.036	0.03611	0.04492	0.02477	0.02259
0.04	0.09413	0.03211	0.09217	0.04	0.04114	0.09315	0.02665	0.03167	0.04	0.03493	0.04299	0.02347	0.02129
0.044	0.09208	0.03039	0.0899	0.044	0.03958	0.09087	0.02665	0.03053	0.044	0.0337	0.04122	0.02239	0.02014
0.048	0.09005	0.02885	0.08762	0.048	0.03804	0.0888	0.02656	0.02939	0.048	0.03252	0.03956	0.02125	0.01887
0.052	0.08813	0.02732	0.08534	0.052	0.03644	0.08631	0.02643	0.02838	0.052	0.03145	0.03785	0.02007	0.01777
0.056	0.08601	0.02579	0.08307	0.056	0.03508	0.08402	0.02611	0.02732	0.056	0.03024	0.03622	0.01896	0.01656

0.06	0.08425	0.02445	0.0813	0.06	0.03378	0.08216	0.02602	0.02639	0.06	0.02928	0.03476	0.01798	0.01567
0.064	0.08232	0.02291	0.07902	0.064	0.03233	0.07988	0.02576	0.02539	0.064	0.02824	0.03321	0.01693	0.01466
0.068	0.08056	0.02157	0.077	0.068	0.03079	0.0778	0.02545	0.02451	0.068	0.02717	0.0317	0.01582	0.01365
0.072	0.07879	0.02042	0.07523	0.072	0.02942	0.07552	0.02522	0.02353	0.072	0.02613	0.03058	0.0148	0.0127
0.076	0.07703	0.01927	0.0732	0.076	0.02828	0.07344	0.02468	0.02266	0.076	0.02516	0.029	0.01382	0.0118
0.08	0.0751	0.01793	0.07118	0.08	0.02695	0.07137	0.0243	0.02178	0.08	0.02412	0.02771	0.01277	0.01102
0.084	0.07328	0.01694	0.06916	0.084	0.02569	0.06929	0.02383	0.02091	0.084	0.02311	0.02642	0.01178	0.01013
0.088	0.07148	0.01571	0.06739	0.088	0.02441	0.06722	0.02325	0.02006	0.088	0.02209	0.02516	0.01083	0.00932
0.092	0.06955	0.01471	0.06536	0.092	0.02324	0.06515	0.02281	0.01913	0.092	0.02116	0.02395	0.00994	0.00863
0.096	0.06782	0.01368	0.06334	0.096	0.02212	0.06328	0.02224	0.01839	0.096	0.0202	0.02277	0.00905	0.00785
0.1	0.06583	0.0127	0.06157	0.1	0.02079	0.0612	0.02141	0.01752	0.1	0.01926	0.02168	0.00813	0.00716
0.104	0.06391	0.01167	0.05954	0.104	0.01977	0.05934	0.02097	0.01667	0.104	0.0183	0.02041	0.0074	0.00661
0.108	0.06201	0.01065	0.05777	0.108	0.01853	0.05726	0.02033	0.01587	0.108	0.01743	0.01934	0.00657	0.00594
0.112	0.05993	0.00985	0.0555	0.112	0.01748	0.05519	0.01979	0.01518	0.112	0.01649	0.01819	0.00578	0.0054
0.116	0.058	0.009	0.05347	0.116	0.01641	0.05332	0.01909	0.01436	0.116	0.01561	0.01713	0.00505	0.00488
0.12	0.05602	0.00816	0.0517	0.12	0.01541	0.05124	0.01842	0.01359	0.12	0.01463	0.0162	0.00422	0.00433
0.124	0.05399	0.0073	0.04968	0.124	0.01443	0.04938	0.01785	0.01293	0.124	0.01389	0.01513	0.00372	0.00381
0.128	0.05201	0.00646	0.04766	0.128	0.01343	0.0473	0.017	0.01216	0.128	0.01304	0.01432	0.00324	0.00349
0.132	0.04992	0.00584	0.04588	0.132	0.01252	0.04544	0.01639	0.01153	0.132	0.01218	0.01314	0.00264	0.00297
0.136	0.04806	0.00504	0.04361	0.136	0.01166	0.04336	0.01569	0.01079	0.136	0.01142	0.01238	0.00219	0.00254
0.14	0.04581	0.00435	0.04184	0.14	0.01068	0.04129	0.01509	0.01012	0.14	0.01059	0.01146	0.00175	0.00225
0.144	0.04363	0.00372	0.03956	0.144	0.00981	0.03942	0.01442	0.00943	0.144	0.0098	0.0105	0.00133	0.0019
0.148	0.04167	0.00312	0.03754	0.148	0.00888	0.03755	0.01366	0.00882	0.148	0.00903	0.0096	0.00105	0.00162
0.152	0.03946	0.00253	0.03526	0.152	0.00811	0.03548	0.01274	0.00811	0.152	0.00829	0.00862	6.67E-04	0.00121
0.156	0.03738	0.00216	0.03324	0.156	0.0072	0.03361	0.0121	0.00739	0.156	0.00768	0.00783	5.72E-04	0.00104
0.16	0.03532	0.00165	0.03121	0.16	0.0065	0.03174	0.01137	0.00673	0.16	0.00692	0.00682	2.86E-04	7.50E-04
0.164	0.03317	0.00126	0.02894	0.164	0.00569	0.02967	0.01058	0.00602	0.164	0.00628	0.00606	1.91E-04	4.62E-04
0.168	0.03099	8.81E-04	0.02717	0.168	0.0049	0.02759	0.00982	0.00535	0.168	0.0056	0.00517	3.18E-05	2.60E-04
0.172	0.02894	5.94E-04	0.02489	0.172	0.00415	0.02573	0.00899	0.00477	0.172	0.00494	0.00432	6.35E-05	1.15E-04
0.176	0.02682	2.49E-04	0.02287	0.176	0.00343	0.02365	0.00813	0.00411	0.176	0.00428	0.00359	0	0
0.18	0.02474	3.83E-05	0.02059	0.18	0.0028	0.02158	0.00731	0.00342	0.18	0.00368	0.00278	3.18E-04	2.89E-05
0.184	0.02252	3.83E-05	0.01831	0.184	0.00217	0.01971	0.00661	0.00278	0.184	0.00299	0.00208	4.13E-04	2.89E-05
0.188	0.0206	0	0.01637	0.188	0.00168	0.01784	0.00562	0.00223	0.188	0.00252	0.0014	7.62E-04	2.02E-04
0.192	0.01864	1.15E-04	0.01434	0.192	0.00107	0.01598	0.0048	0.00167	0.192	0.002	8.42E-04	0.00124	5.48E-04
0.196	0.01675	4.02E-04	0.01237	0.196	6.29E-04	0.01369	0.00384	0.00119	0.196	0.00143	4.49E-04	0.00181	9.23E-04
0.2	0.01495	8.05E-04	0.01052	0.2	2.80E-04	0.01203	0.00318	7.68E-04	0.2	0.0011	0	0.00267	0.0017
0.204	0.01322	0.00149	0.0088	0.204	9.32E-05	0.01017	0.00245	3.97E-04	0.204	6.59E-04	1.12E-04	0.00359	0.00242
0.208	0.01152	0.00241	0.00698	0.208	0	0.00851	0.00165	1.85E-04	0.208	3.84E-04	5.62E-05	0.00489	0.00349
0.212	0.00975	0.00356	0.00536	0.212	2.33E-05	0.00705	0.00105	2.65E-05	0.212	2.47E-04	2.53E-04	0.00616	0.00473
0.216	0.00821	0.00508	0.00382	0.216	4.66E-05	0.00539	5.72E-04	0	0.216	0	7.86E-04	0.00791	0.00632
0.22	0.00677	0.00682	0.00273	0.22	5.13E-04	0.00415	1.91E-04	7.95E-05	0.22	1.65E-04	0.0018	0.01	0.00834
0.224	0.00552	0.00889	0.00159	0.224	0.00107	0.00311	6.35E-05	5.30E-04	0.224	2.74E-04	0.00292	0.01248	0.01059
0.228	0.00423	0.01138	7.84E-04	0.228	0.00191	0.00187	0	0.00101	0.228	4.67E-04	0.00463	0.01537	0.0133
0.232	0.00327	0.01456	1.77E-04	0.232	0.00308	0.00104	1.27E-04	0.00172	0.232	9.88E-04	0.00682	0.01883	0.01659
0.236	0.00237	0.01812	7.59E-05	0.236	0.00455	6.22E-04	5.72E-04	0.00278	0.236	0.00173	0.00963	0.02287	0.02051
0.24	0.00141	0.02215	0	0.24	0.00627	2.07E-04	0.00149	0.00424	0.24	0.0025	0.01311	0.02766	0.02507
0.244	8.02E-04	0.02675	4.55E-04	0.244	0.00839	0	0.00257	0.00599	0.244	0.00357	0.01735	0.03332	0.0305
0.248	2.89E-04	0.03192	0.00132	0.248	0.01091	0	0.00397	0.00821	0.248	0.00502	0.02241	0.04005	0.03679
0.252	6.42E-05	0.03767	0.00283	0.252	0.01392	4.15E-04	0.00591	0.01105	0.252	0.00683	0.02881	0.04786	0.04417
0.256	0	0.04418	0.00488	0.256	0.01755	0.00124	0.00848	0.01442	0.256	0.00889	0.03627	0.05717	0.05283
0.26	9.62E-05	0.05089	0.00739	0.26	0.02156	0.00228	0.01166	0.01818	0.26	0.01147	0.04503	0.06803	0.06287
0.264	5.13E-04	0.05817	0.01067	0.264	0.02632	0.00373	0.01557	0.023	0.264	0.01446	0.05545	0.08064	0.07467
0.268	9.95E-04	0.06602	0.01472	0.268	0.03154	0.00539	0.02033	0.02854	0.268	0.01789	0.06736	0.09518	0.08832
0.272	0.00176	0.07368	0.01933	0.272	0.03725	0.00768	0.02608	0.03501	0.272	0.02179	0.08092	0.11211	0.1039
0.276	0.00311	0.08173	0.02464	0.276	0.04355	0.00996	0.03282	0.04216	0.276	0.02626	0.09588	0.13091	0.1215
0.28	0.00417	0.08978	0.03071	0.28	0.05038	0.01266	0.04076	0.05038	0.28	0.03131	0.11247	0.15178	0.14135
0.284	0.00565	0.09763	0.03779	0.284	0.05765	0.01598	0.04965	0.05957	0.284	0.03691	0.13019	0.17423	0.16325
0.288	0.00767	0.10491	0.04487	0.288	0.06513	0.01909	0.05963	0.06948	0.288	0.04297	0.14872	0.19776	0.18691
0.292	0.00966	0.11181	0.05246	0.292	0.07245	0.02241	0.07021	0.07987	0.292	0.04931	0.16669	0.22095	0.21175
0.296	0.012	0.11794	0.0603	0.296	0.07991	0.02552	0.0812	0.0905	0.296	0.05582	0.18354	0.24254	0.23685
0.3	0.01473	0.1233	0.06739	0.3	0.0869	0.02863	0.09181	0.10094	0.3	0.06246	0.19814	0.26096	0.26138
0.304	0.01742	0.1279	0.07396	0.304	0.09273	0.03112	0.10121	0.11029	0.304	0.0685	0.20937	0.2743	0.2833
0.308	0.02034	0.13173	0.07927	0.308	0.09763	0.0332	0.10852	0.11819	0.308	0.07387	0.2161	0.28129	0.30119
0.312	0.02339	0.13518	0.08332	0.312	0.10112	0.03465	0.11306	0.12378	0.312	0.07805	0.21779	0.28066	0.31302
0.316	0.02663	0.13863	0.08585	0.316	0.10345	0.03568	0.11423	0.1268	0.316	0.08112	0.21498	0.27272	0.31821
0.32	0.03009	0.14227	0.08686	0.32	0.10462	0.0361	0.11239	0.12693	0.32	0.0826	0.20796	0.25906	0.31533
0.324	0.03362	0.14649	0.08661	0.324	0.10462	0.0361	0.10801	0.12463	0.324	0.08285	0.19785	0.24096	0.30465
0.328	0.03744	0.15204	0.08585	0.328	0.10415	0.03672	0.10251	0.12026	0.328	0.08241	0.18634	0.2219	0.28821
0.332	0.04167	0.15875	0.08484	0.332	0.10345	0.03714	0.09711	0.11498	0.332	0.08159	0.17511	0.2038	0.26859
0.336	0.04633	0.16699	0.08383	0.336	0.10345	0.03797	0.09276	0.10952	0.336	0.08071	0.16444	0.18792	0.24781
0.34	0.05156	0.17676	0.08357	0.34	0.10392	0.03942	0.09009	0.10467	0.34	0.0803	0.15574	0.17544	0.22935
0.344	0.05739	0.18825	0.08383	0.344	0.10509	0.04129	0.08911	0.10099	0.344	0.08052	0.14956	0.1662	0.21463
0.348	0.06394	0.20147	0.08509	0.348	0.10765	0.04419	0.08996	0.09903	0.348	0.08156	0.14591	0.16035	0.20454
0.352	0.07145	0.21603	0.08737	0.352	0.11138	0.04772	0.09235	0.09874	0.352	0.08353	0.14451	0.15746	0.19877
0.356	0.07982	0.23117	0.09116	0.356	0.11628	0.05207	0.09629	0.09993	0.356	0.08622	0.14591	0.1573	0.19732
0.36	0.08916	0.24726	0.09597	0.36	0.12234	0.05726	0.10181	0.10301	0.36	0.09004	0.14928	0.15962	0.19963
0.364	0.0999	0.26393	0.10204	0.364	0.12956	0.06369	0.10874	0.1077	0.364	0.09503	0.15518	0.16445	0.20569
0.368	0.1112	0.27983	0.10912	0.368	0.13749	0.07033	0.11662	0.11334	0.368	0.10052	0.1622	0.17042	0.21348
0.372	0.1232	0.29497	0.11671	0.372	0.14611	0.07759	0.12539	0.11997	0.372	0.10694	0.17062	0.1775	0.223
0.376	0.13603	0.30895	0.12506	0.376	0.15497	0.08506	0.13472	0.12715	0.376	0.11386	0.17989	0.18506	0.23339
0.38	0.14886	0.32121	0.13391	0.38	0.16406	0.09295	0.14438	0.13486	0.38	0.12132	0.18915	0.193	0.24406
0.384	0.16234	0.33137	0.14302	0.384	0.17292	0.10041	0.15429	0.14257	0.384	0.12906	0.1987	0.20126	0.25416
0.388	0.17485	0.33903	0.15162	0.388	0.18085	0.10768	0.16408	0.14967	0.388	0.13669	0.20768	0.20856	0.26282
0.392	0.18704	0.3442	0.16047	0.392	0.18831	0.11411	0.17358	0.15651	0.392	0.14415	0.2161	0.21555	0.27003
0.396	0.19795	0.34688	0.16806	0.396	0.1946	0.11992	0.18241	0.16234	0.396	0.15121	0.22369	0.22127	0.27551
0.4	0.20725	0.34708	0.17514	0.4	0.19996	0.12469	0.19057	0.16711	0.4	0.15779	0.23014	0.22603	0.27869
0.404	0.21495	0.34516	0.18121	0.404	0.20369	0.12822	0.19788	0.17082	0.404	0.16356	0.2352	0.22984	0.27984
0.408	0.22073	0.34133	0.18627	0.408	0.20649	0.13029	0.20455	0.17373	0.408	0.16822	0.23913	0.23238	0.27984
0.412	0.22457	0.33596	0.19057	0.412	0.20789	0.13133	0.20995	0.17532	0.412	0.17206	0.24194	0.23429	0.27811
0.416	0.22586	0.32945	0.19335	0.416	0.20789	0.13091	0.2144	0.17638	0.416	0.17508	0.24334	0.23524	0.27522
0.42	0.22522	0.32236	0.19512	0.42	0.20719	0.12905	0.21789	0.17638	0.42	0.177	0.2439	0.23524	0.27205
0.424	0.22265	0.31489	0.19639	0.424	0.20602	0.12676	0.22043	0.17585	0.424	0.1781	0.24362	0.23492	0.26888
0.428	0.2188	0.30742	0.19689	0.428	0.20393	0.12303	0.22202	0.17532	0.428	0.17865	0.24334	0.2346	0.26599
0.432	0.21367	0.30014	0.19664	0.432	0.20113	0.11888	0.22297	0.17426	0.432	0.17783	0.24222	0.23333	0.26282
0.436	0.20757	0.29305	0.19563	0.436	0.1981	0.11411	0.22329	0.17294	0.436	0.17673	0.24053	0.23206	0.25993
0.44	0.20115	0.28634	0.19411	0.44	0.1953	0.10892	0.22266	0.17161	0.44	0.17481	0.23885	0.23016	0.25791
0.444	0.19442	0.2804	0.19209	0.444	0.19204	0.10394	0.22234	0.17002	0.444	0.17234	0.2366	0.22857	0.25618
0.448	0.18768	0.27466	0.18981	0.448	0.18924	0.09855	0.2217	0.16843	0.448	0.16959	0.23464	0.22698	0.25474
0.452	0.18126	0.26929	0.18728	0.452	0.18621	0.09357	0.22043	0.16684	0.452	0.16658	0.23267	0.22508	0.25358
0.456	0.17517	0.2645	0.18475	0.456	0.18341	0.08859	0.21948	0.16552	0.456	0.16356	0.2307	0.22381	0.25272
0.46	0.16971	0.2601	0.18248	0.46	0.18108	0.08423	0.21884	0.16393	0.46	0.16054	0.22874	0.22254	0.25243
0.464	0.1649	0.25607	0.17995	0.464	0.17875	0.07988	0.21821	0.1626	0.464	0.15752	0.22705	0.22158	0.25243
0.468	0.16073	0.25243	0.17792	0.468	0.17688	0.07614	0.21789	0.16128	0.468	0.1545	0.22593	0.22095	0.25272

0.472	0.15688	0.24918	0.17615	0.472	0.17502	0.07282	0.21789	0.16022	0.472	0.15176	0.22481	0.22063	0.2533
0.476	0.15399	0.24611	0.17438	0.476	0.17362	0.06971	0.21757	0.15916	0.476	0.14929	0.22397	0.22031	0.25445
0.48	0.15175	0.24305	0.17337	0.48	0.17269	0.06701	0.21821	0.15836	0.48	0.14715	0.2234	0.22063	0.2556
0.484	0.14982	0.24036	0.17211	0.484	0.17199	0.06452	0.21884	0.15757	0.484	0.14498	0.22284	0.22063	0.25705
0.488	0.14822	0.23787	0.17135	0.488	0.17129	0.06224	0.21948	0.15704	0.488	0.14328	0.22256	0.22127	0.25849
0.492	0.14726	0.23557	0.17109	0.492	0.17129	0.06037	0.22107	0.15651	0.492	0.14171	0.22256	0.22222	0.2608
0.496	0.14662	0.23308	0.17084	0.496	0.17129	0.05851	0.22202	0.15651	0.496	0.14031	0.22256	0.22285	0.26253
0.5	0.14597	0.23098	0.17084	0.5	0.17152	0.05685	0.22361	0.15624	0.5	0.13913	0.22256	0.22381	0.26484
0.504	0.14597	0.22887	0.17084	0.504	0.17222	0.05539	0.22488	0.15595	0.504	0.13834	0.22284	0.22444	0.26715
0.508	0.14629	0.22695	0.17135	0.508	0.17292	0.05415	0.22647	0.15598	0.508	0.13768	0.22369	0.22571	0.26945
0.512	0.14662	0.22504	0.1716	0.512	0.17385	0.05311	0.22806	0.15598	0.512	0.13713	0.22425	0.22698	0.27205
0.516	0.14694	0.22293	0.17185	0.516	0.17455	0.05207	0.22965	0.15598	0.516	0.1368	0.22481	0.22794	0.27407
0.52	0.1479	0.2212	0.17236	0.52	0.17572	0.05124	0.23155	0.15598	0.52	0.13683	0.22565	0.22952	0.27638
0.524	0.14854	0.21967	0.17286	0.524	0.17688	0.05062	0.23314	0.15624	0.524	0.13705	0.22649	0.23079	0.2784
0.528	0.1495	0.21814	0.17362	0.528	0.17828	0.04979	0.23505	0.15651	0.528	0.1374	0.22734	0.2327	0.28071
0.532	0.15047	0.21642	0.17413	0.532	0.17968	0.04938	0.23663	0.15677	0.532	0.13787	0.22874	0.23429	0.28301
0.536	0.15175	0.21488	0.17489	0.536	0.18108	0.04896	0.23854	0.15704	0.536	0.13864	0.23014	0.23619	0.28503
0.54	0.15303	0.21373	0.17565	0.54	0.18271	0.04855	0.24013	0.15757	0.54	0.1396	0.23155	0.2381	0.28734
0.544	0.15432	0.21258	0.17615	0.544	0.18434	0.04834	0.24172	0.1581	0.544	0.1407	0.23323	0.24032	0.28965
0.548	0.1556	0.21143	0.17716	0.548	0.18621	0.04813	0.24331	0.15863	0.548	0.1419	0.23492	0.24223	0.29167
0.552	0.1572	0.21067	0.17792	0.552	0.18784	0.04813	0.24521	0.15942	0.552	0.14317	0.23688	0.24445	0.29398
0.556	0.15881	0.2099	0.17893	0.556	0.18994	0.04834	0.24712	0.16022	0.556	0.14476	0.23913	0.24667	0.29658
0.56	0.16009	0.20933	0.17995	0.56	0.1918	0.04834	0.24871	0.16128	0.56	0.14638	0.24137	0.2489	0.29888
0.564	0.16169	0.20875	0.18071	0.564	0.1939	0.04855	0.24998	0.16234	0.564	0.14805	0.24334	0.25112	0.30119
0.568	0.1633	0.20837	0.18197	0.568	0.196	0.04876	0.25188	0.1634	0.568	0.14984	0.24587	0.25366	0.3035
0.572	0.1649	0.20837	0.18323	0.572	0.19833	0.04938	0.25347	0.16472	0.572	0.15176	0.24839	0.25652	0.30639
0.576	0.16651	0.20837	0.18399	0.576	0.20043	0.05	0.25506	0.16605	0.576	0.15368	0.25064	0.25906	0.30898
0.58	0.16811	0.20818	0.18526	0.58	0.20253	0.05041	0.25633	0.16737	0.58	0.1556	0.25317	0.2616	0.31158
0.584	0.17004	0.20837	0.18627	0.584	0.20486	0.05083	0.25824	0.16896	0.584	0.15752	0.25597	0.26414	0.31418
0.588	0.17164	0.20856	0.18779	0.588	0.20719	0.05166	0.25982	0.17055	0.588	0.15971	0.2585	0.267	0.31677
0.592	0.17356	0.20894	0.1888	0.592	0.20929	0.05228	0.26173	0.17214	0.592	0.16191	0.26131	0.26986	0.31937
0.596	0.17517	0.20913	0.19032	0.596	0.21162	0.05311	0.26332	0.17373	0.596	0.16411	0.26412	0.2724	0.32197
0.6	0.17709	0.20971	0.19158	0.6	0.21395	0.05394	0.26522	0.17559	0.6	0.1663	0.26692	0.27526	0.32427
0.604	0.17902	0.21028	0.19335	0.604	0.21628	0.05477	0.26713	0.17744	0.604	0.1685	0.26973	0.27811	0.32687
0.608	0.18094	0.21105	0.19462	0.608	0.21838	0.05581	0.26904	0.1793	0.608	0.17069	0.27254	0.28129	0.32947
0.612	0.18255	0.21163	0.19613	0.612	0.22048	0.05664	0.27094	0.18115	0.612	0.17289	0.27507	0.28415	0.33149
0.616	0.18415	0.21239	0.1974	0.616	0.22281	0.05768	0.27317	0.18301	0.616	0.17536	0.27815	0.28701	0.33379
0.62	0.18576	0.21316	0.19917	0.62	0.22491	0.05892	0.27507	0.18486	0.62	0.17783	0.28096	0.29018	0.3361
0.624	0.18736	0.21431	0.20094	0.624	0.227	0.05996	0.2773	0.18698	0.624	0.17975	0.28377	0.29336	0.33812
0.628	0.18864	0.21507	0.20246	0.628	0.2291	0.061	0.27952	0.18884	0.628	0.18194	0.28658	0.29622	0.34014
0.632	0.18993	0.21603	0.20398	0.632	0.2312	0.06224	0.28143	0.19096	0.632	0.18414	0.28938	0.29939	0.34187
0.636	0.19121	0.21718	0.206	0.636	0.2333	0.06349	0.28365	0.19308	0.636	0.18661	0.29219	0.30257	0.34389
0.64	0.19217	0.21833	0.20752	0.64	0.23493	0.06473	0.28587	0.1952	0.64	0.18853	0.295	0.30575	0.34562
0.644	0.19346	0.21929	0.20929	0.644	0.23679	0.06577	0.28778	0.19732	0.644	0.19045	0.29753	0.3086	0.34736
0.648	0.19442	0.22063	0.21131	0.648	0.23866	0.06722	0.28969	0.19944	0.648	0.19237	0.30005	0.31178	0.34938
0.652	0.19538	0.22197	0.21283	0.652	0.24052	0.06867	0.29159	0.20129	0.652	0.19429	0.30286	0.31496	0.35111
0.656	0.19634	0.22331	0.2146	0.656	0.24216	0.06992	0.2935	0.20341	0.656	0.19649	0.30539	0.31813	0.35313
0.66	0.19698	0.22484	0.21637	0.66	0.24355	0.07116	0.2954	0.20553	0.66	0.19813	0.30791	0.32099	0.35486
0.664	0.19763	0.22619	0.21814	0.664	0.24495	0.07282	0.29699	0.20739	0.664	0.19978	0.31016	0.32417	0.35659
0.668	0.19827	0.22772	0.22016	0.668	0.24635	0.07427	0.2989	0.20924	0.668	0.2017	0.31241	0.32734	0.35861
0.672	0.19923	0.22944	0.22219	0.672	0.24775	0.07573	0.30049	0.21163	0.672	0.20362	0.31465	0.3302	0.36063
0.676	0.19987	0.23098	0.22396	0.676	0.24868	0.07739	0.30176	0.21322	0.676	0.20527	0.31662	0.33306	0.36236
0.68	0.20051	0.2327	0.22573	0.68	0.24985	0.07905	0.30335	0.21534	0.68	0.20692	0.31886	0.3356	0.36438
0.684	0.20148	0.23442	0.2275	0.684	0.25101	0.08071	0.30462	0.21719	0.684	0.20856	0.32083	0.33846	0.36611
0.688	0.20212	0.23615	0.22927	0.688	0.25195	0.08237	0.30589	0.21905	0.688	0.21021	0.32251	0.341	0.36813
0.692	0.20308	0.23768	0.23129	0.692	0.25288	0.08423	0.30684	0.22064	0.692	0.21158	0.3242	0.34354	0.36986
0.696	0.20404	0.23941	0.23307	0.696	0.25381	0.08589	0.30811	0.22223	0.696	0.21323	0.3256	0.34576	0.37159
0.7	0.205	0.24113	0.23484	0.7	0.25474	0.08797	0.30938	0.22408	0.7	0.2146	0.32701	0.34767	0.37332
0.704	0.20597	0.24305	0.23661	0.704	0.25568	0.08983	0.31065	0.22567	0.704	0.21625	0.32869	0.34989	0.37477
0.708	0.20725	0.24477	0.23838	0.708	0.25638	0.09191	0.3116	0.22726	0.708	0.21762	0.32953	0.35148	0.3765
0.712	0.20853	0.24669	0.24015	0.712	0.25707	0.09378	0.31256	0.22885	0.712	0.21926	0.33094	0.35307	0.37794
0.716	0.20982	0.2486	0.24217	0.716	0.25801	0.09606	0.31383	0.23018	0.716	0.22064	0.33206	0.35465	0.37938
0.72	0.21142	0.25033	0.24369	0.72	0.25894	0.09834	0.3151	0.23177	0.72	0.22228	0.33318	0.35592	0.38054
0.724	0.21303	0.25224	0.24521	0.724	0.25964	0.10041	0.31605	0.23309	0.724	0.22366	0.33403	0.35688	0.3814
0.728	0.21463	0.25397	0.24698	0.728	0.26034	0.10249	0.31732	0.23442	0.728	0.2253	0.33487	0.35815	0.38227
0.732	0.21623	0.25607	0.24849	0.732	0.2615	0.10498	0.31859	0.23574	0.732	0.22695	0.33571	0.3591	0.38313
0.736	0.21816	0.25818	0.25052	0.736	0.26244	0.10768	0.31986	0.23707	0.736	0.22859	0.33711	0.36005	0.384
0.74	0.22008	0.2599	0.25153	0.74	0.26314	0.10996	0.32113	0.23839	0.74	0.23024	0.33768	0.36101	0.38458
0.744	0.22201	0.2622	0.2533	0.744	0.2643	0.11245	0.32241	0.23972	0.744	0.23189	0.3388	0.36164	0.38515
0.748	0.22425	0.2645	0.25507	0.748	0.26547	0.11494	0.32368	0.24104	0.748	0.23381	0.33964	0.36228	0.38573
0.752	0.2265	0.2668	0.25659	0.752	0.26663	0.11763	0.32495	0.24237	0.752	0.23546	0.34076	0.36323	0.38602
0.756	0.22875	0.2691	0.25811	0.756	0.26803	0.12012	0.32654	0.24369	0.756	0.23738	0.34189	0.36386	0.3866
0.76	0.23131	0.2714	0.25962	0.76	0.2692	0.12282	0.32781	0.24502	0.76	0.23902	0.34301	0.36418	0.38717
0.764	0.23388	0.27427	0.26114	0.764	0.2706	0.12531	0.32908	0.24661	0.764	0.24094	0.34441	0.36513	0.38775
0.768	0.23677	0.27676	0.26291	0.768	0.27199	0.12822	0.33035	0.24793	0.768	0.24286	0.34582	0.36577	0.38833
0.772	0.23901	0.27945	0.26443	0.772	0.27339	0.13091	0.33194	0.24926	0.772	0.24479	0.34694	0.36641	0.3889
0.776	0.24254	0.28309	0.26671	0.776	0.27549	0.13382	0.33352	0.25111	0.776	0.24726	0.34919	0.36768	0.39035
0.78	0.24511	0.28596	0.26848	0.78	0.27712	0.13651	0.33511	0.2527	0.78	0.24945	0.35087	0.36863	0.39121
0.784	0.24832	0.28922	0.2705	0.784	0.27875	0.13921	0.33638	0.25429	0.784	0.25137	0.35284	0.36958	0.39208
0.788	0.25152	0.29247	0.27227	0.788	0.28062	0.14191	0.33797	0.25588	0.788	0.25329	0.35452	0.37053	0.39323
0.792	0.25441	0.29573	0.27404	0.792	0.28248	0.14481	0.33956	0.25774	0.792	0.25549	0.35649	0.37149	0.39439
0.796	0.25762	0.29937	0.27632	0.796	0.28435	0.14772	0.34147	0.25959	0.796	0.25768	0.35873	0.37307	0.39583
0.8	0.26115	0.3032	0.2786	0.8	0.28668	0.15062	0.34305	0.26171	0.8	0.25988	0.36098	0.37434	0.39727
0.804	0.26404	0.30704	0.28062	0.804	0.28854	0.15353	0.34496	0.26357	0.804	0.26207	0.36323	0.37562	0.399
0.808	0.26724	0.31106	0.2829	0.808	0.29064	0.15664	0.34718	0.26595	0.808	0.26454	0.36575	0.37752	0.40073
0.812	0.27077	0.31527	0.28568	0.812	0.29344	0.15975	0.34941	0.26807	0.812	0.26701	0.36856	0.37911	0.40275
0.816	0.27366	0.31987	0.28821	0.816	0.29577	0.16307	0.35163	0.27046	0.816	0.26948	0.37137	0.38101	0.40506
0.82	0.27687	0.32428	0.29074	0.82	0.29834	0.16639	0.35385	0.27284	0.82	0.27195	0.37446	0.38292	0.40708
0.824	0.27944	0.32888	0.29327	0.824	0.30067	0.16971	0.35608	0.27549	0.824	0.27442	0.37698	0.38451	0.40939
0.828	0.28264	0.33405	0.29655	0.828	0.30346	0.17344	0.35926	0.27841	0.828	0.27717	0.38035	0.38705	0.41227
0.832	0.28617	0.33903	0.29959	0.832	0.30626	0.17759	0.36211	0.28159	0.832	0.28019	0.384	0.38927	0.41487
0.836	0.28906	0.34439	0.30263	0.836	0.30906	0.18154	0.36529	0.2845	0.836	0.28266	0.38737	0.39181	0.41804
0.84	0.29227	0.34976	0.30642	0.84	0.31232	0.18589	0.36879	0.28821	0.84	0.28595	0.3913	0.39467	0.42122
0.844	0.29548	0.35551	0.31021	0.844	0.31559	0.19046	0.3726	0.29166	0.844	0.28924	0.39551	0.39753	0.42468
0.848	0.29836	0.36125	0.31426	0.848	0.31908	0.19523	0.37641	0.29563	0.848	0.29254	0.39972	0.40039	0.42843
0.852	0.30157	0.36719	0.31831	0.852	0.32258	0.20021	0.38054	0.29934	0.852	0.29583	0.40394	0.40356	0.43218
0.856	0.30478	0.37332	0.32261	0.856	0.32631	0.20519	0.38467	0.30358	0.856	0.2994	0.40843	0.40674	0.43593
0.86	0.30799	0.37926	0.32716	0.86	0.33027	0.21058	0.38943	0.30809	0.86	0.30324	0.41348	0.41023	0.44026
0.864	0.3112	0.38578	0.33197	0.864	0.33447	0.21618	0.3942	0.31286	0.864	0.30708	0.41854	0.41404	0.44459
0.868	0.31408	0.39172	0.33677	0.868	0.33843	0.22158	0.39896	0.31736	0.868	0.31092	0.42331	0.41754	0.44892
0.872	0.31761	0.39804	0.34183	0.872	0.34286	0.22739	0.40405	0.3224	0.872	0.31504	0.42864	0.42167	0.45353
0.876	0.32114	0.40474	0.3474	0.876	0.34752	0.2334	0.40945	0.3277	0.876	0.3197	0.43454	0.42579	0.45815
0.88	0.32467	0.41087	0.35296	0.88	0.35242	0.239	0.41485	0.33273	0.88	0.32409	0.44015	0.43024	0.46305

0.884	0.3282	0.41701	0.35853	0.884	0.35731	0.24481	0.41993	0.33803	0.884	0.32876	0.44605	0.43437	0.46738
0.888	0.33205	0.42314	0.36409	0.888	0.36221	0.25062	0.42533	0.34333	0.888	0.33315	0.45195	0.43882	0.472
0.892	0.3359	0.42946	0.37016	0.892	0.36734	0.25643	0.43105	0.3489	0.892	0.33809	0.45812	0.44326	0.47719
0.896	0.33943	0.43559	0.37623	0.896	0.3727	0.26224	0.43645	0.35473	0.896	0.3433	0.4643	0.44803	0.48181
0.9	0.34328	0.44134	0.38205	0.9	0.37806	0.26784	0.44185	0.36003	0.9	0.34824	0.47076	0.45247	0.48642
0.904	0.34713	0.44728	0.38837	0.904	0.38365	0.27386	0.44725	0.36586	0.904	0.35346	0.47722	0.45724	0.49133
0.908	0.35162	0.45321	0.39445	0.908	0.38902	0.27967	0.45297	0.37142	0.908	0.35895	0.48395	0.46168	0.49623
0.912	0.35611	0.45896	0.40052	0.912	0.39484	0.28548	0.45837	0.37699	0.912	0.36443	0.49041	0.46645	0.50056
0.916	0.3606	0.46433	0.40659	0.916	0.40044	0.29149	0.46377	0.38255	0.916	0.36992	0.49687	0.47089	0.50518
0.92	0.36509	0.46969	0.41291	0.92	0.40627	0.29751	0.46885	0.38785	0.92	0.37569	0.50333	0.47566	0.50951
0.924	0.36991	0.47506	0.41923	0.924	0.41209	0.30394	0.47425	0.39315	0.924	0.38145	0.50978	0.4801	0.51383
0.928	0.37472	0.48023	0.4253	0.928	0.41792	0.31017	0.4787	0.39872	0.928	0.38721	0.51596	0.48455	0.51816
0.932	0.37985	0.48502	0.43112	0.932	0.42352	0.31639	0.48347	0.40349	0.932	0.39243	0.52186	0.48868	0.5222
0.936	0.38499	0.48981	0.43719	0.936	0.42911	0.32282	0.48823	0.40879	0.936	0.39846	0.52803	0.49312	0.52624
0.94	0.39044	0.4946	0.44326	0.94	0.43471	0.32905	0.493	0.41382	0.94	0.40423	0.53393	0.49725	0.53028
0.944	0.39557	0.499	0.44908	0.944	0.44007	0.33506	0.49744	0.41886	0.944	0.40971	0.53955	0.50138	0.53403
0.948	0.40071	0.50303	0.45414	0.948	0.4452	0.34108	0.50189	0.42336	0.948	0.41548	0.54488	0.50519	0.53778
0.952	0.40584	0.50705	0.45971	0.952	0.45009	0.34689	0.5057	0.42813	0.952	0.42097	0.54993	0.509	0.54124
0.956	0.41065	0.51069	0.46476	0.956	0.45475	0.3529	0.50983	0.4329	0.956	0.42645	0.55471	0.51281	0.54471
0.96	0.41578	0.51414	0.47008	0.96	0.45918	0.3583	0.51396	0.43741	0.96	0.43167	0.5592	0.51631	0.54817
0.964	0.42028	0.51701	0.47488	0.964	0.46315	0.36307	0.51746	0.44165	0.964	0.43661	0.56313	0.5198	0.55077
0.968	0.42509	0.51989	0.47944	0.968	0.46711	0.36784	0.52127	0.44589	0.968	0.44182	0.56678	0.5233	0.55394
0.972	0.42926	0.52238	0.48348	0.972	0.4706	0.37241	0.52508	0.45013	0.972	0.44621	0.56987	0.52615	0.55654
0.976	0.43343	0.52468	0.48728	0.976	0.47364	0.37656	0.52857	0.4541	0.976	0.4506	0.57268	0.52901	0.55884
0.98	0.4376	0.52678	0.49057	0.98	0.47643	0.38008	0.53207	0.45781	0.98	0.45472	0.5752	0.53187	0.56086
0.984	0.44081	0.52812	0.4936	0.984	0.47876	0.3834	0.53525	0.46126	0.984	0.45856	0.57689	0.53441	0.56288
0.988	0.44434	0.52966	0.49613	0.988	0.48086	0.38651	0.53842	0.4647	0.988	0.4624	0.57829	0.53632	0.56462
0.992	0.44722	0.531	0.49841	0.992	0.48273	0.38942	0.5416	0.46788	0.992	0.4657	0.5797	0.53854	0.56577
0.996	0.45011	0.53196	0.50018	0.996	0.48413	0.3917	0.54414	0.47106	0.996	0.46872	0.58054	0.54013	0.56663
1	0.45268	0.53291	0.50195	1	0.48529	0.39419	0.547	0.47424	1	0.47146	0.5811	0.54172	0.56779
1.004	0.45525	0.53387	0.50321	1.004	0.48669	0.39647	0.54986	0.47716	1.004	0.47393	0.58166	0.5433	0.56865
1.008	0.45749	0.53464	0.50448	1.008	0.48739	0.39876	0.5524	0.48007	1.008	0.4764	0.58166	0.54457	0.56923
1.012	0.45974	0.53541	0.50524	1.012	0.48832	0.40083	0.55526	0.48272	1.012	0.47859	0.58138	0.54584	0.56981
1.016	0.46198	0.53617	0.50574	1.016	0.48902	0.4029	0.5578	0.48564	1.016	0.48079	0.58138	0.54711	0.57039
1.02	0.46423	0.53713	0.50701	1.02	0.48995	0.40539	0.56034	0.48882	1.02	0.48271	0.58138	0.54807	0.57096
1.024	0.46647	0.53809	0.50751	1.024	0.49065	0.40788	0.56288	0.492	1.024	0.48491	0.58138	0.54934	0.57154
1.028	0.46808	0.53866	0.50777	1.028	0.49135	0.40996	0.56511	0.49518	1.028	0.48655	0.5811	0.54997	0.57212
1.032	0.47032	0.54	0.50852	1.032	0.49182	0.41266	0.56765	0.49889	1.032	0.48847	0.5811	0.55124	0.57298
1.036	0.47257	0.54096	0.50928	1.036	0.49275	0.41556	0.57051	0.50233	1.036	0.4904	0.58138	0.55251	0.57385
1.04	0.47482	0.5423	0.51004	1.04	0.49368	0.41846	0.57337	0.50604	1.04	0.49232	0.58166	0.55378	0.575
1.044	0.47674	0.54364	0.51055	1.044	0.49462	0.42116	0.57591	0.50949	1.044	0.49424	0.58194	0.55505	0.57616
1.048	0.47899	0.54498	0.51156	1.048	0.49578	0.42407	0.57908	0.51346	1.048	0.49616	0.5825	0.55632	0.57731
1.052	0.48187	0.54671	0.51257	1.052	0.49718	0.42718	0.58194	0.51797	1.052	0.49863	0.58335	0.55791	0.57904
1.056	0.48412	0.54843	0.51409	1.056	0.49858	0.43029	0.58544	0.52221	1.056	0.50082	0.58447	0.5595	0.58077
1.06	0.48701	0.54997	0.51535	1.06	0.49998	0.4334	0.58861	0.52671	1.06	0.50274	0.58531	0.56109	0.58193
1.064	0.48957	0.55207	0.51662	1.064	0.50161	0.43714	0.59211	0.53148	1.064	0.50521	0.58643	0.56299	0.58423
1.068	0.49278	0.55437	0.51839	1.068	0.50371	0.44066	0.5956	0.53652	1.068	0.50768	0.58812	0.5649	0.58625
1.072	0.49567	0.55667	0.52016	1.072	0.50604	0.44461	0.5991	0.54129	1.072	0.51043	0.58952	0.56712	0.58827
1.076	0.49888	0.55897	0.52193	1.076	0.50814	0.44834	0.60291	0.54632	1.076	0.5129	0.59121	0.56935	0.59058
1.08	0.50241	0.56146	0.52446	1.08	0.5107	0.45207	0.60704	0.55189	1.08	0.51564	0.59317	0.57125	0.59289
1.084	0.50626	0.56472	0.52724	1.084	0.51373	0.45664	0.61149	0.55745	1.084	0.51921	0.59542	0.57411	0.59578
1.088	0.50979	0.56778	0.52977	1.088	0.51676	0.461	0.61562	0.56302	1.088	0.5225	0.59766	0.57697	0.59837
1.092	0.51363	0.57085	0.5323	1.092	0.52002	0.46535	0.61975	0.56858	1.092	0.52552	0.59963	0.57983	0.60097
1.096	0.51781	0.5743	0.53559	1.096	0.52352	0.47012	0.62419	0.57441	1.096	0.52881	0.60244	0.583	0.60443
1.1	0.52198	0.57794	0.53888	1.1	0.52748	0.4749	0.62896	0.58051	1.1	0.53238	0.60524	0.58586	0.60761
1.104	0.52647	0.58196	0.54242	1.104	0.53145	0.48008	0.63372	0.5866	1.104	0.53622	0.60833	0.58935	0.61107
1.108	0.53128	0.58579	0.54621	1.108	0.53541	0.48527	0.63849	0.5927	1.108	0.53979	0.61114	0.59253	0.61453
1.112	0.53609	0.58962	0.55026	1.112	0.53984	0.49087	0.64357	0.59932	1.112	0.54391	0.61423	0.59602	0.61799
1.116	0.54219	0.59403	0.55431	1.116	0.5445	0.49668	0.64897	0.60595	1.116	0.54802	0.61788	0.60015	0.62203
1.12	0.547	0.59882	0.55886	1.12	0.5494	0.5029	0.65437	0.61284	1.12	0.55269	0.62153	0.60396	0.62636
1.124	0.55245	0.60342	0.56316	1.124	0.55452	0.50892	0.65977	0.61946	1.124	0.55708	0.62546	0.60809	0.6304
1.128	0.55791	0.60878	0.56847	1.128	0.55989	0.51556	0.66517	0.62662	1.128	0.56229	0.62967	0.61254	0.63473
1.132	0.56465	0.61396	0.57378	1.132	0.56548	0.5222	0.67089	0.63351	1.132	0.56751	0.63388	0.6173	0.63963
1.136	0.57106	0.61932	0.5791	1.136	0.57131	0.52925	0.67693	0.64066	1.136	0.57272	0.63837	0.62207	0.64425
1.14	0.57748	0.62488	0.58441	1.14	0.57737	0.53631	0.68296	0.64808	1.14	0.57821	0.64287	0.62715	0.64915
1.144	0.58422	0.63062	0.59023	1.144	0.58366	0.54378	0.68932	0.6555	1.144	0.58397	0.64764	0.63223	0.65435
1.148	0.59127	0.63695	0.5968	1.148	0.59042	0.55145	0.69567	0.66319	1.148	0.59029	0.65326	0.63795	0.65983
1.152	0.59865	0.64288	0.60338	1.152	0.59742	0.55934	0.70266	0.67087	1.152	0.5966	0.65859	0.64366	0.66531
1.156	0.60603	0.64902	0.60996	1.156	0.60441	0.56701	0.70901	0.67829	1.156	0.60291	0.66392	0.64938	0.67079
1.16	0.61373	0.65572	0.61704	1.16	0.61187	0.57573	0.71632	0.68598	1.16	0.6095	0.66982	0.65541	0.67685
1.164	0.62207	0.66243	0.62437	1.164	0.61956	0.58444	0.72363	0.69419	1.164	0.61636	0.676	0.66208	0.6832
1.168	0.63009	0.66932	0.63196	1.168	0.62726	0.59315	0.73061	0.70188	1.168	0.62377	0.68217	0.66843	0.68984
1.172	0.63843	0.67622	0.63955	1.172	0.63495	0.60207	0.7376	0.71009	1.172	0.6309	0.68835	0.6751	0.69618
1.176	0.64678	0.6835	0.64714	1.176	0.64287	0.6112	0.74491	0.71831	1.176	0.63886	0.69481	0.68177	0.70311
1.18	0.65544	0.69097	0.65523	1.18	0.65127	0.62075	0.75253	0.72626	1.18	0.64682	0.70155	0.68908	0.71003
1.184	0.66506	0.6994	0.66434	1.184	0.66036	0.63112	0.76079	0.735	1.184	0.65587	0.70941	0.69702	0.71811
1.188	0.6734	0.70707	0.67294	1.188	0.66922	0.64066	0.7681	0.74295	1.188	0.66411	0.71615	0.70464	0.72532
1.192	0.68271	0.71492	0.68154	1.192	0.67807	0.65083	0.77572	0.75117	1.192	0.67316	0.72401	0.71226	0.73311
1.196	0.69201	0.72335	0.69065	1.196	0.68763	0.66141	0.78367	0.75991	1.196	0.68222	0.73159	0.72052	0.7409
1.2	0.70132	0.7314	0.69975	1.2	0.69696	0.67178	0.79129	0.76813	1.2	0.69127	0.73945	0.72846	0.74869
1.204	0.71062	0.73983	0.70936	1.204	0.70651	0.68237	0.79923	0.77634	1.204	0.7006	0.74703	0.73672	0.75648
1.208	0.72056	0.74845	0.71898	1.208	0.71607	0.69315	0.80749	0.78509	1.208	0.71021	0.75545	0.74529	0.76485
1.212	0.73115	0.75745	0.72884	1.212	0.72656	0.70477	0.81543	0.79383	1.212	0.72036	0.76388	0.7545	0.77351
1.216	0.7411	0.76627	0.73946	1.216	0.73682	0.71577	0.82337	0.80231	1.216	0.73024	0.77258	0.76339	0.78187
1.22	0.75136	0.77527	0.74958	1.22	0.74684	0.72676	0.831	0.81079	1.22	0.74067	0.78128	0.7726	0.79053
1.224	0.76195	0.78447	0.76021	1.224	0.75756	0.73859	0.83926	0.81927	1.224	0.75137	0.79027	0.78213	0.79947
1.228	0.77286	0.79405	0.77108	1.228	0.76852	0.75041	0.8472	0.82802	1.228	0.76235	0.79925	0.79166	0.80871
1.232	0.78345	0.80343	0.78196	1.232	0.77971	0.76224	0.85514	0.83676	1.232	0.77333	0.80852	0.80151	0.81765
1.236	0.79403	0.81301	0.79258	1.236	0.79043	0.77386	0.86277	0.84524	1.236	0.7843	0.81722	0.81072	0.82688
1.24	0.80526	0.82297	0.80371	1.24	0.80162	0.78631	0.87103	0.85372	1.24	0.79583	0.82705	0.82088	0.83641
1.244	0.81681	0.83332	0.8156	1.244	0.81328	0.79896	0.87897	0.863	1.244	0.80763	0.83688	0.83136	0.84622
1.248	0.82868	0.84424	0.82749	1.248	0.82493	0.81183	0.88723	0.87227	1.248	0.81943	0.84726	0.84216	0.85631
1.252	0.84055	0.85439	0.83862	1.252	0.83682	0.82448	0.89517	0.88102	1.252	0.83123	0.85737	0.85264	0.86584
1.256	0.85274	0.86551	0.85076	1.256	0.84918	0.83776	0.90375	0.89029	1.256	0.84385	0.86832	0.86375	0.87593
1.26	0.86526	0.87643	0.86316	1.26	0.862	0.85145	0.91169	0.89957	1.26	0.85675	0.87899	0.87519	0.88603
1.264	0.87745	0.88792	0.8758	1.264	0.87459	0.86494	0.91995	0.90884	1.264	0.86965	0.88994	0.88694	0.89671
1.268	0.88964	0.89923	0.88845	1.268	0.88717	0.87863	0.92821	0.91812	1.268	0.88282	0.90117	0.89837	0.90709
1.272	0.90247	0.91091	0.9011	1.272	0.90046	0.89253	0.93678	0.92766	1.272	0.89627	0.91268	0.91012	0.91777
1.276	0.91562	0.92279	0.91501	1.276	0.91422	0.90705	0.94536	0.93746	1.276	0.91026	0.92447	0.92251	0.92902
1.28	0.9291	0.93505	0.92842	1.28	0.92774	0.92178	0.95457	0.9478	1.28	0.92481	0.93683	0.93458	0.94028
1.284	0.94225	0.94712	0.94182	1.284	0.94149	0.93651	0.96283	0.95734	1.284	0.9388	0.94862	0.94696	0.95153
1.288	0.95669	0.96053	0.95675	1.288	0.95618	0.95228	0.97236	0.9682	1.288	0.95417	0.96154	0.9603	0.96365
1.292	0.97081	0.97375	0.97116	1.292	0.97063	0.96784	0.98158	0.97854	1.292	0.96926	0.97417	0.97332	0.97548

1.296	0.98556	0.98678	0.98558	1.296	0.98531	0.98402	0.99047	0.9894	1.296	0.98436	0.98708	0.98666	0.98788
1.3	1	1	1	1.3	1	1	1	1	1.3	1	1	1	1
					--	--							
**Ethanol-PBS (Spring)**				**Ethanol-PBS (Summer)**					**Ethanol-PBS (Autumn)**				
0.004	0.02023	0.02503	0.02428	0.004	0.03607	0.03375	0.01599	0.00375	0.004	0.04686	0.04379	0.03547	0.05688
0.008	0.01756	0.0213	0.02083	0.008	0.03111	0.03021	0.01292	0.00277	0.008	0.0408	0.03601	0.03163	0.04737
0.012	0.01477	0.01783	0.01759	0.012	0.02615	0.02707	0.0102	0.00178	0.012	0.03555	0.03025	0.02779	0.03971
0.016	0.01226	0.01463	0.01489	0.016	0.02209	0.02386	0.00767	9.84E-04	0.016	0.03054	0.02492	0.02403	0.03302
0.02	0.01016	0.01199	0.01252	0.02	0.01803	0.02137	0.00535	5.54E-04	0.02	0.02634	0.02024	0.02058	0.02694
0.024	0.00817	0.00954	0.01019	0.024	0.01443	0.01856	0.0038	0	0.024	0.02222	0.01619	0.01743	0.02237
0.028	0.00623	0.00754	0.00818	0.028	0.01127	0.01617	0.00221	6.15E-05	0.028	0.01834	0.01279	0.01489	0.01823
0.032	0.00477	0.00568	0.00643	0.032	0.00812	0.01384	0.00101	3.08E-04	0.032	0.01527	0.00967	0.01198	0.01418
0.036	0.0032	0.00397	0.00492	0.036	0.00541	0.0118	2.89E-04	8.00E-04	0.036	0.01212	0.00689	0.0096	0.01101
0.04	0.0021	0.00232	0.00337	0.04	0.00361	0.00982	0	0.00166	0.04	0.00953	0.00486	0.00729	0.00801
0.044	0.00125	0.00136	0.00235	0.044	0.0018	0.00806	1.81E-04	0.00271	0.044	0.00719	0.00316	0.0056	0.00537
0.048	6.88E-04	4.80E-04	0.0014	0.048	9.02E-04	0.00618	0.00116	0.00437	0.048	0.00493	0.0016	0.00407	0.00343
0.052	2.02E-04	0	5.61E-04	0.052	0	0.0046	0.0025	0.00615	0.052	0.00331	6.61E-04	0.00246	0.00185
0.056	0	0	1.30E-04	0.056	4.51E-04	0.00332	0.00481	0.00818	0.056	0.00178	0	0.00115	7.92E-04
0.06	1.62E-04	1.60E-04	0	0.06	0.0018	0.00211	0.00796	0.01132	0.06	9.70E-04	2.36E-04	6.91E-04	3.52E-04
0.064	6.88E-04	6.66E-04	1.73E-04	0.064	0.00316	0.00133	0.01194	0.01452	0.064	3.23E-04	8.49E-04	3.07E-04	0
0.068	0.00125	0.00195	8.42E-04	0.068	0.00541	6.03E-04	0.01686	0.01846	0.068	0	0.00208	0	4.40E-04
0.072	0.00235	0.00336	0.0019	0.072	0.00902	1.76E-04	0.02287	0.02301	0.072	4.04E-04	0.00368	5.37E-04	0.00167
0.076	0.00401	0.00565	0.00324	0.076	0.01353	0	0.03021	0.02812	0.076	0.00105	0.00642	0.00161	0.00352
0.08	0.00575	0.0081	0.00509	0.08	0.01894	1.51E-04	0.03849	0.03396	0.08	0.0025	0.00934	0.00307	0.00581
0.084	0.00785	0.0113	0.00762	0.084	0.0257	4.77E-04	0.04787	0.04061	0.084	0.00436	0.01326	0.00507	0.00863
0.088	0.01056	0.01517	0.01034	0.088	0.03336	0.00118	0.05883	0.04793	0.088	0.00727	0.01741	0.00714	0.01259
0.092	0.01356	0.01916	0.01362	0.092	0.04283	0.00234	0.07091	0.05587	0.092	0.01058	0.02256	0.00983	0.01691
0.096	0.01703	0.02423	0.01759	0.096	0.0532	0.00359	0.08394	0.0646	0.096	0.01511	0.0285	0.01351	0.02263
0.1	0.02092	0.02956	0.02191	0.1	0.06447	0.00517	0.09805	0.07402	0.1	0.01996	0.03464	0.01781	0.02835
0.104	0.02525	0.03542	0.02666	0.104	0.07665	0.00716	0.11324	0.0838	0.104	0.02594	0.04124	0.0228	0.0354
0.108	0.03002	0.04155	0.03184	0.108	0.09017	0.00932	0.12952	0.09432	0.108	0.03256	0.04842	0.02787	0.04332
0.112	0.035	0.04822	0.03724	0.112	0.10415	0.01175	0.1458	0.10515	0.112	0.04007	0.05616	0.03424	0.05195
0.116	0.0403	0.05515	0.04307	0.116	0.11858	0.01442	0.16281	0.11641	0.116	0.04896	0.06375	0.04138	0.06164
0.12	0.04584	0.06208	0.04868	0.12	0.13345	0.01713	0.17945	0.12798	0.12	0.05841	0.07168	0.0486	0.0722
0.124	0.05179	0.06927	0.05472	0.124	0.14833	0.02004	0.19609	0.13936	0.124	0.06908	0.07985	0.05735	0.08409
0.128	0.0579	0.07647	0.06077	0.128	0.16276	0.02306	0.21274	0.15148	0.128	0.08031	0.08744	0.06664	0.09703
0.132	0.06381	0.0834	0.06659	0.132	0.17628	0.02612	0.22829	0.16305	0.132	0.09283	0.0949	0.07708	0.11059
0.136	0.07061	0.09086	0.07242	0.136	0.18981	0.02873	0.24313	0.17517	0.136	0.10616	0.10292	0.08845	0.12574
0.14	0.07789	0.09806	0.07825	0.14	0.20243	0.0321	0.25687	0.18723	0.14	0.12063	0.11	0.10073	0.14211
0.144	0.08521	0.10579	0.08386	0.144	0.21326	0.03541	0.2699	0.19953	0.144	0.13606	0.11755	0.11417	0.15964
0.148	0.09319	0.11352	0.08948	0.148	0.22453	0.03868	0.2822	0.21227	0.148	0.15246	0.12557	0.12829	0.17839
0.152	0.10176	0.12204	0.0953	0.152	0.2349	0.04212	0.29378	0.22574	0.152	0.16951	0.13359	0.14357	0.19838
0.156	0.11107	0.13111	0.10178	0.156	0.24482	0.04563	0.30499	0.23996	0.156	0.18712	0.14209	0.16038	0.21942
0.16	0.12151	0.14097	0.10869	0.16	0.25518	0.0499	0.31621	0.25497	0.16	0.20587	0.15105	0.1772	0.24126
0.164	0.13248	0.15216	0.11603	0.164	0.26555	0.05417	0.32706	0.27078	0.164	0.2238	0.16049	0.19417	0.26345
0.168	0.14461	0.16442	0.12445	0.168	0.27728	0.05894	0.33828	0.28782	0.168	0.24222	0.1704	0.21175	0.28537
0.172	0.15797	0.17775	0.13416	0.172	0.2899	0.06422	0.34949	0.30499	0.172	0.26024	0.18126	0.22864	0.3066
0.176	0.17213	0.19268	0.14495	0.176	0.30343	0.07	0.36071	0.32314	0.176	0.27729	0.19211	0.24453	0.32649
0.18	0.1871	0.20813	0.15661	0.18	0.3183	0.07627	0.37192	0.34148	0.18	0.2928	0.20296	0.2585	0.34481
0.184	0.20288	0.22493	0.16956	0.184	0.33454	0.08305	0.38314	0.35981	0.184	0.30734	0.21382	0.27086	0.36101
0.188	0.21947	0.24172	0.18359	0.188	0.35167	0.09034	0.39436	0.37735	0.188	0.32003	0.22467	0.28084	0.37422
0.192	0.23606	0.25931	0.19827	0.192	0.3697	0.09737	0.40485	0.39519	0.192	0.33045	0.23458	0.28814	0.38461
0.196	0.25265	0.2761	0.2136	0.196	0.38729	0.10465	0.41462	0.41057	0.196	0.33853	0.2426	0.29251	0.3906
0.2	0.26884	0.29236	0.22893	0.2	0.40532	0.11169	0.42366	0.42472	0.2	0.34483	0.24968	0.29459	0.39403
0.204	0.28421	0.30755	0.24404	0.204	0.42245	0.11872	0.43126	0.43703	0.204	0.34887	0.25534	0.29382	0.39368
0.208	0.29797	0.32088	0.25828	0.208	0.43868	0.125	0.43669	0.44749	0.208	0.35041	0.25865	0.29083	0.38954
0.212	0.31051	0.3326	0.27145	0.212	0.45311	0.13077	0.44139	0.45549	0.212	0.34984	0.26006	0.28568	0.38241
0.216	0.32103	0.3422	0.28289	0.216	0.46573	0.1358	0.44428	0.46164	0.216	0.34782	0.25959	0.27893	0.37299
0.22	0.32993	0.3494	0.29261	0.22	0.47701	0.13981	0.44609	0.46533	0.22	0.34378	0.25723	0.27125	0.3611
0.224	0.33681	0.35366	0.29995	0.224	0.48512	0.14283	0.44682	0.46779	0.224	0.33845	0.25204	0.26273	0.34736
0.228	0.34045	0.35553	0.30448	0.228	0.49008	0.14459	0.44573	0.46779	0.228	0.33094	0.24543	0.25305	0.33187
0.232	0.34248	0.35499	0.30642	0.232	0.49279	0.14534	0.44392	0.46656	0.232	0.32294	0.23788	0.24353	0.31593
0.236	0.34207	0.3518	0.30577	0.236	0.49234	0.14509	0.44139	0.4641	0.236	0.31381	0.22892	0.23386	0.29929
0.24	0.34005	0.34646	0.30254	0.24	0.49008	0.14308	0.43813	0.46041	0.24	0.30379	0.21854	0.22418	0.28238
0.244	0.33641	0.33927	0.29692	0.244	0.48467	0.14032	0.43379	0.45672	0.244	0.29296	0.20768	0.21443	0.26521
0.248	0.33155	0.33047	0.28915	0.248	0.47656	0.1363	0.42909	0.45118	0.248	0.28222	0.19636	0.20507	0.24875
0.252	0.32589	0.32088	0.28009	0.252	0.46754	0.13153	0.42475	0.44503	0.252	0.27139	0.18598	0.19601	0.23298
0.256	0.31982	0.31048	0.26951	0.256	0.45672	0.126	0.41968	0.43887	0.256	0.26008	0.17465	0.18695	0.21819
0.26	0.31254	0.29902	0.25785	0.26	0.44409	0.11997	0.41462	0.4321	0.26	0.24885	0.16332	0.17843	0.2034
0.264	0.30606	0.28809	0.2462	0.264	0.43192	0.1137	0.41027	0.42595	0.264	0.23851	0.15294	0.17021	0.19037
0.268	0.29918	0.27637	0.23411	0.268	0.41885	0.10742	0.40557	0.41918	0.268	0.22808	0.14303	0.16207	0.17778
0.272	0.29271	0.26517	0.22223	0.272	0.40532	0.10114	0.40123	0.41303	0.272	0.21855	0.13312	0.15432	0.16633
0.276	0.28583	0.25424	0.21036	0.276	0.39179	0.09461	0.39689	0.40811	0.276	0.20966	0.12463	0.14672	0.15488
0.28	0.27976	0.24332	0.19914	0.28	0.37917	0.08858	0.39327	0.40196	0.28	0.20134	0.11566	0.13958	0.14502
0.284	0.2741	0.23346	0.18856	0.284	0.36655	0.0823	0.39038	0.39765	0.284	0.19367	0.10811	0.13305	0.13586
0.288	0.26884	0.22359	0.17863	0.288	0.35437	0.07678	0.38784	0.39334	0.288	0.18696	0.10103	0.1266	0.12759
0.292	0.26358	0.21453	0.16935	0.292	0.34265	0.07125	0.38567	0.38965	0.292	0.18058	0.0949	0.12046	0.11949
0.296	0.25872	0.20574	0.16093	0.296	0.33138	0.06648	0.38423	0.38719	0.296	0.1746	0.08876	0.11516	0.11253
0.3	0.25427	0.19774	0.15294	0.3	0.32101	0.06196	0.3835	0.38534	0.3	0.16975	0.08338	0.11025	0.10566
0.304	0.25022	0.18974	0.1456	0.304	0.31109	0.05769	0.38314	0.3835	0.304	0.16531	0.07852	0.10595	0.09976
0.308	0.24618	0.18281	0.13891	0.308	0.30207	0.05417	0.3835	0.38227	0.308	0.16127	0.07399	0.10196	0.09413
0.312	0.24253	0.17588	0.13287	0.312	0.29306	0.05091	0.38386	0.38104	0.312	0.15755	0.06989	0.09843	0.08902
0.316	0.2393	0.16975	0.12768	0.316	0.28494	0.04789	0.38567	0.38165	0.316	0.15472	0.06635	0.09574	0.08462
0.32	0.23566	0.16389	0.12272	0.32	0.27728	0.04513	0.38712	0.38165	0.32	0.15222	0.06338	0.09351	0.08127
0.324	0.23242	0.15856	0.11819	0.324	0.27006	0.04312	0.38929	0.38288	0.324	0.14971	0.06069	0.09098	0.07749
0.328	0.22959	0.1535	0.11409	0.328	0.26375	0.04111	0.39182	0.38411	0.328	0.14802	0.05847	0.08944	0.07467
0.332	0.22675	0.1487	0.11063	0.332	0.25744	0.03943	0.39472	0.38596	0.332	0.14656	0.05668	0.08852	0.07238
0.336	0.22433	0.14443	0.10718	0.336	0.25158	0.0379	0.39797	0.38842	0.336	0.14575	0.05521	0.08752	0.07035
0.34	0.2219	0.14044	0.10416	0.34	0.24617	0.03697	0.40159	0.39088	0.34	0.14511	0.05403	0.08691	0.06886
0.344	0.21947	0.1367	0.10157	0.344	0.24121	0.03596	0.40557	0.39273	0.344	0.14454	0.05347	0.08645	0.06771
0.348	0.21745	0.13351	0.09941	0.348	0.2367	0.03536	0.41027	0.39642	0.348	0.14438	0.05309	0.0866	0.06754
0.352	0.21542	0.13057	0.09725	0.352	0.23264	0.03493	0.41462	0.3995	0.352	0.14414	0.05314	0.08699	0.06762
0.356	0.21381	0.12791	0.0953	0.356	0.22858	0.03463	0.41896	0.40319	0.356	0.14438	0.05304	0.08745	0.06762
0.36	0.21219	0.12551	0.09358	0.36	0.22543	0.03461	0.42475	0.40688	0.36	0.14462	0.05408	0.08845	0.06842
0.364	0.21138	0.12364	0.0925	0.364	0.22272	0.03496	0.43054	0.4118	0.364	0.14592	0.05554	0.09006	0.07027
0.368	0.21016	0.12178	0.0912	0.368	0.22002	0.03519	0.43596	0.41611	0.368	0.1468	0.05686	0.09167	0.07159
0.372	0.20976	0.12018	0.09034	0.372	0.21776	0.03561	0.44103	0.42103	0.372	0.14785	0.0588	0.09367	0.07388
0.376	0.20895	0.11858	0.08926	0.376	0.21506	0.03614	0.44682	0.42534	0.376	0.14915	0.06069	0.09574	0.07625
0.38	0.20855	0.11725	0.08861	0.38	0.21371	0.03682	0.45297	0.43026	0.38	0.15125	0.0638	0.09812	0.07889
0.384	0.20814	0.11591	0.08818	0.384	0.21235	0.0377	0.45876	0.4358	0.384	0.15351	0.06658	0.10096	0.08259
0.388	0.20814	0.11485	0.08753	0.388	0.211	0.03828	0.46454	0.44072	0.388	0.15561	0.06975	0.10349	0.08611
0.392	0.20814	0.11405	0.08732	0.392	0.2101	0.03913	0.47069	0.44626	0.392	0.15852	0.07319	0.10695	0.09025
0.396	0.20855	0.11325	0.08689	0.396	0.2092	0.03996	0.47648	0.45179	0.396	0.16159	0.07711	0.11071	0.09448

0.4	0.20895	0.11245	0.08667	0.4	0.20875	0.04086	0.48191	0.45795	0.4	0.16515	0.08131	0.11409	0.09941
0.404	0.20895	0.11165	0.08645	0.404	0.2083	0.04162	0.48661	0.46348	0.404	0.16838	0.08518	0.11793	0.10408
0.408	0.20976	0.11085	0.08624	0.408	0.20784	0.04237	0.49132	0.46841	0.408	0.1725	0.08971	0.12177	0.10918
0.412	0.21057	0.11058	0.08602	0.412	0.20784	0.04312	0.49566	0.47333	0.412	0.17694	0.09443	0.12583	0.11411
0.416	0.21097	0.10952	0.08559	0.416	0.20739	0.04337	0.49891	0.47702	0.416	0.18155	0.09867	0.12983	0.11922
0.42	0.21138	0.10898	0.08559	0.42	0.20694	0.04388	0.50181	0.48133	0.42	0.18615	0.10339	0.13382	0.12389
0.424	0.21219	0.10819	0.08516	0.424	0.20694	0.04438	0.5047	0.4844	0.424	0.19124	0.10764	0.13797	0.129
0.428	0.213	0.10792	0.08494	0.428	0.20694	0.04488	0.50687	0.48686	0.428	0.19633	0.11189	0.14226	0.13384
0.432	0.2134	0.10739	0.08473	0.432	0.20694	0.04513	0.50796	0.48809	0.432	0.20183	0.11613	0.1461	0.1385
0.436	0.21381	0.10685	0.08451	0.436	0.20694	0.04563	0.50904	0.48809	0.436	0.20684	0.11991	0.15017	0.14308
0.44	0.21421	0.10632	0.08408	0.44	0.20649	0.04588	0.50977	0.48809	0.44	0.21184	0.12321	0.15386	0.14705
0.444	0.21462	0.10605	0.08408	0.444	0.20649	0.04614	0.51085	0.48686	0.444	0.21685	0.12652	0.1577	0.15074
0.448	0.21502	0.10605	0.08408	0.448	0.20649	0.04639	0.51158	0.48563	0.448	0.2213	0.12935	0.16123	0.15453
0.452	0.21502	0.10552	0.08365	0.452	0.20649	0.04639	0.51194	0.48317	0.452	0.22509	0.13171	0.16438	0.15708
0.456	0.21542	0.10552	0.08365	0.456	0.20649	0.04639	0.5123	0.48133	0.456	0.22881	0.13359	0.16722	0.16017
0.46	0.21542	0.10579	0.08386	0.46	0.20649	0.04664	0.51302	0.47948	0.46	0.23172	0.13501	0.17006	0.16281
0.464	0.21542	0.10552	0.08386	0.464	0.20649	0.04664	0.51339	0.47702	0.464	0.23382	0.13643	0.1719	0.1651
0.468	0.21542	0.10579	0.08408	0.468	0.20649	0.04664	0.51411	0.47517	0.468	0.23503	0.13737	0.17367	0.1673
0.472	0.21502	0.10605	0.0843	0.472	0.20649	0.04714	0.51447	0.47333	0.472	0.23544	0.13784	0.17489	0.16923
0.476	0.21502	0.10632	0.08451	0.476	0.20649	0.04714	0.5152	0.4721	0.476	0.23536	0.13879	0.17582	0.17091
0.48	0.21462	0.10659	0.08494	0.48	0.20649	0.04739	0.51556	0.47025	0.48	0.23447	0.13926	0.17681	0.17276
0.484	0.21462	0.10712	0.08516	0.484	0.20649	0.04764	0.51556	0.46902	0.484	0.23326	0.13926	0.17704	0.17381
0.488	0.21381	0.10739	0.08559	0.488	0.20694	0.04815	0.51628	0.46841	0.488	0.2318	0.13973	0.17781	0.17557
0.492	0.2134	0.10819	0.08624	0.492	0.20739	0.04815	0.51664	0.46718	0.492	0.23051	0.14067	0.17827	0.17698
0.496	0.213	0.10872	0.08689	0.496	0.20784	0.0489	0.51664	0.46656	0.496	0.22905	0.14114	0.17858	0.17866
0.5	0.21259	0.10925	0.08732	0.5	0.2083	0.04915	0.51664	0.46533	0.5	0.22744	0.14114	0.17935	0.18015
0.504	0.21178	0.11005	0.08818	0.504	0.20875	0.0499	0.51737	0.46471	0.504	0.22615	0.14209	0.17996	0.18156
0.508	0.21138	0.11085	0.08883	0.508	0.2101	0.05041	0.51773	0.46471	0.508	0.22526	0.14303	0.18096	0.18297
0.512	0.21138	0.11138	0.08969	0.512	0.21145	0.05091	0.51809	0.4641	0.512	0.22469	0.1435	0.18188	0.18464
0.516	0.21097	0.11218	0.09034	0.516	0.21235	0.05166	0.51845	0.46348	0.516	0.22308	0.14445	0.1825	0.18588
0.52	0.21057	0.11298	0.09142	0.52	0.21371	0.05267	0.51918	0.46348	0.52	0.22251	0.14539	0.1838	0.18737
0.524	0.21057	0.11378	0.09228	0.524	0.21506	0.05317	0.52026	0.4641	0.524	0.2217	0.14634	0.18495	0.1894
0.528	0.21057	0.11485	0.09336	0.528	0.21686	0.05417	0.52098	0.4641	0.528	0.22106	0.14728	0.18649	0.19046
0.532	0.21097	0.11565	0.09444	0.532	0.21867	0.05518	0.52243	0.4641	0.532	0.22025	0.1487	0.18787	0.19204
0.536	0.21097	0.11645	0.09552	0.536	0.22047	0.05593	0.52352	0.46471	0.536	0.21968	0.14964	0.18964	0.19345
0.54	0.21178	0.11778	0.09682	0.54	0.22272	0.05719	0.52533	0.46594	0.54	0.21952	0.15105	0.19117	0.19539
0.544	0.21219	0.11885	0.09811	0.544	0.22498	0.05819	0.52677	0.46656	0.544	0.21936	0.15247	0.19294	0.19697
0.548	0.213	0.11991	0.09941	0.548	0.22723	0.0592	0.52786	0.46779	0.548	0.21871	0.15389	0.19463	0.19856
0.552	0.21381	0.12098	0.10092	0.552	0.22904	0.06045	0.52931	0.46841	0.552	0.21895	0.15577	0.19647	0.20067
0.556	0.21462	0.12204	0.10221	0.556	0.23129	0.06171	0.53111	0.47025	0.556	0.21904	0.15766	0.19893	0.20234
0.56	0.21583	0.12311	0.10372	0.56	0.23399	0.06271	0.53256	0.47148	0.56	0.21952	0.15955	0.20092	0.20419
0.564	0.21664	0.12471	0.10523	0.564	0.2358	0.06422	0.53365	0.47333	0.564	0.21976	0.16144	0.20299	0.20604
0.568	0.21745	0.12578	0.10675	0.568	0.2385	0.06573	0.53473	0.47456	0.568	0.22041	0.16332	0.20522	0.20833
0.572	0.21907	0.12738	0.10847	0.572	0.24121	0.06698	0.53582	0.4764	0.572	0.22138	0.16568	0.20783	0.2108
0.576	0.22028	0.12871	0.1102	0.576	0.24301	0.06824	0.53654	0.47763	0.576	0.22243	0.16757	0.21036	0.21335
0.58	0.22109	0.13004	0.11149	0.58	0.24527	0.06974	0.5369	0.47887	0.58	0.2234	0.16946	0.21259	0.21537
0.584	0.2223	0.13164	0.11322	0.584	0.24797	0.0715	0.53726	0.48071	0.584	0.22477	0.17229	0.2152	0.21819
0.588	0.22352	0.13324	0.11495	0.588	0.25023	0.07276	0.53726	0.48194	0.588	0.22606	0.17465	0.21781	0.22039
0.592	0.22433	0.13484	0.11668	0.592	0.25203	0.07452	0.53654	0.48317	0.592	0.2272	0.17701	0.22019	0.22303
0.596	0.22514	0.1367	0.1184	0.596	0.25428	0.07627	0.53582	0.4844	0.596	0.22873	0.1789	0.22273	0.22559
0.6	0.22594	0.13857	0.12035	0.6	0.25699	0.07778	0.53509	0.48563	0.6	0.23002	0.18126	0.22541	0.22779
0.604	0.22675	0.1407	0.12229	0.604	0.25924	0.07954	0.53365	0.48686	0.604	0.2318	0.18362	0.22802	0.23017
0.608	0.22756	0.14283	0.12423	0.608	0.2615	0.08155	0.53256	0.48748	0.608	0.23301	0.18598	0.23056	0.23272
0.612	0.22797	0.1447	0.12596	0.612	0.26375	0.08331	0.53039	0.48809	0.612	0.23455	0.18739	0.23263	0.23466
0.616	0.22918	0.14737	0.12812	0.616	0.26601	0.08557	0.52858	0.48933	0.616	0.23576	0.18975	0.23524	0.23668
0.62	0.22999	0.15003	0.13028	0.62	0.26871	0.08758	0.52677	0.48994	0.62	0.23729	0.19211	0.2377	0.23906
0.624	0.2308	0.15243	0.13243	0.624	0.27096	0.08984	0.52424	0.49056	0.624	0.23859	0.194	0.23992	0.24153
0.628	0.2312	0.15563	0.13438	0.628	0.27322	0.09235	0.52207	0.49117	0.628	0.24004	0.19589	0.2423	0.24329
0.632	0.23201	0.15829	0.13675	0.632	0.27547	0.09461	0.51918	0.49117	0.632	0.24117	0.19777	0.24422	0.24487
0.636	0.23323	0.16176	0.13891	0.636	0.27818	0.09737	0.517	0.49179	0.636	0.24263	0.20013	0.24653	0.2469
0.64	0.23404	0.16549	0.1415	0.64	0.28088	0.09988	0.51447	0.4924	0.64	0.24384	0.20202	0.24883	0.24883
0.644	0.23525	0.16895	0.14387	0.644	0.28314	0.10264	0.51158	0.4924	0.644	0.24481	0.20344	0.2506	0.24998
0.648	0.23606	0.17269	0.14668	0.648	0.28584	0.10566	0.50941	0.49363	0.648	0.24594	0.20532	0.2529	0.25165
0.652	0.23768	0.17642	0.14927	0.652	0.28855	0.10867	0.50687	0.49425	0.652	0.24723	0.20721	0.25482	0.25324
0.656	0.23849	0.18068	0.15229	0.656	0.29125	0.11194	0.5047	0.49486	0.656	0.24844	0.2091	0.25674	0.25456
0.66	0.24011	0.18495	0.1551	0.66	0.29441	0.1152	0.50217	0.49548	0.66	0.2495	0.21051	0.2585	0.25605
0.664	0.24132	0.18921	0.15791	0.664	0.29757	0.11847	0.5	0.49609	0.664	0.25063	0.2124	0.26027	0.25702
0.668	0.24334	0.19374	0.16114	0.668	0.30072	0.12223	0.49819	0.49732	0.668	0.25224	0.21429	0.26234	0.25825
0.672	0.24537	0.19854	0.1646	0.672	0.30433	0.12575	0.49674	0.49855	0.672	0.25345	0.21618	0.26441	0.25984
0.676	0.24699	0.20307	0.16784	0.676	0.30748	0.12952	0.49493	0.49978	0.676	0.25459	0.21759	0.26603	0.26081
0.68	0.24941	0.20813	0.17107	0.68	0.31109	0.13328	0.49349	0.50163	0.68	0.2562	0.21901	0.2681	0.26195
0.684	0.25184	0.21293	0.17474	0.684	0.3147	0.1373	0.4924	0.50348	0.684	0.25782	0.22137	0.27002	0.26345
0.688	0.25467	0.21826	0.1782	0.688	0.3183	0.14107	0.49132	0.50532	0.688	0.25984	0.22278	0.27225	0.26459
0.692	0.2571	0.22333	0.18187	0.692	0.32236	0.14534	0.49023	0.50717	0.692	0.2621	0.22514	0.27424	0.26592
0.696	0.26034	0.22812	0.18554	0.696	0.32597	0.14936	0.48951	0.50963	0.696	0.26428	0.22656	0.27639	0.26715
0.7	0.26358	0.23346	0.18964	0.7	0.33003	0.15338	0.48878	0.51209	0.7	0.2667	0.22845	0.27846	0.26829
0.704	0.26681	0.23852	0.19374	0.704	0.33454	0.15765	0.48878	0.51455	0.704	0.26921	0.23033	0.28054	0.26961
0.708	0.27005	0.24358	0.19763	0.708	0.33814	0.16141	0.48842	0.51701	0.708	0.27171	0.23175	0.28238	0.27041
0.712	0.27369	0.24865	0.20194	0.712	0.34265	0.16568	0.48842	0.52009	0.712	0.2747	0.23364	0.2843	0.27155
0.716	0.27774	0.25371	0.20648	0.716	0.34716	0.1697	0.48842	0.52316	0.716	0.27753	0.23552	0.28637	0.27261
0.72	0.28178	0.25878	0.21101	0.72	0.35122	0.17372	0.48878	0.52563	0.72	0.28052	0.23694	0.28791	0.2734
0.724	0.28583	0.26384	0.21533	0.724	0.35528	0.17774	0.48878	0.5287	0.724	0.28359	0.23836	0.28952	0.27454
0.728	0.29028	0.26864	0.21986	0.728	0.35978	0.18151	0.48915	0.53178	0.728	0.28634	0.23977	0.29121	0.27534
0.732	0.29473	0.2737	0.22461	0.732	0.36384	0.18527	0.48987	0.53486	0.732	0.28949	0.24119	0.29305	0.27631
0.736	0.29918	0.2785	0.22936	0.736	0.3679	0.18929	0.49059	0.53793	0.736	0.29215	0.2426	0.29466	0.27745
0.74	0.30363	0.28303	0.23389	0.74	0.37196	0.19256	0.49132	0.54101	0.74	0.29514	0.24402	0.29551	0.27771
0.744	0.30808	0.28809	0.23864	0.744	0.37556	0.19632	0.49204	0.54408	0.744	0.29805	0.24449	0.2972	0.27859
0.748	0.31254	0.29289	0.24339	0.748	0.38007	0.20009	0.49276	0.54716	0.748	0.30104	0.24591	0.29835	0.27948
0.752	0.31739	0.29716	0.24792	0.752	0.38368	0.20336	0.49385	0.55024	0.752	0.30395	0.24685	0.29942	0.28009
0.756	0.32144	0.30115	0.25202	0.756	0.38683	0.20687	0.49421	0.5527	0.756	0.30678	0.24732	0.3005	0.28053
0.76	0.32589	0.30542	0.25634	0.76	0.39044	0.21014	0.49493	0.55516	0.76	0.30928	0.24827	0.30157	0.28124
0.764	0.33034	0.30942	0.26044	0.764	0.39405	0.21315	0.49602	0.55762	0.764	0.31195	0.24968	0.30288	0.28203
0.768	0.33439	0.31315	0.26411	0.768	0.3972	0.21616	0.49674	0.56008	0.768	0.31429	0.25015	0.30395	0.28256
0.772	0.33843	0.31661	0.26735	0.772	0.40036	0.21893	0.49747	0.56193	0.772	0.31664	0.25063	0.3048	0.28273
0.776	0.34288	0.32088	0.27124	0.776	0.40352	0.22219	0.49855	0.565	0.776	0.31954	0.25204	0.30672	0.28441
0.78	0.34652	0.32434	0.27404	0.78	0.40622	0.2247	0.49891	0.56685	0.78	0.32164	0.25251	0.30772	0.28458
0.784	0.35017	0.32754	0.27642	0.784	0.40848	0.22721	0.49964	0.5687	0.784	0.32407	0.25298	0.30864	0.28546
0.788	0.3534	0.33047	0.27879	0.788	0.41073	0.22947	0.49964	0.57054	0.788	0.32609	0.25298	0.30948	0.28626
0.792	0.35624	0.33314	0.28095	0.792	0.41253	0.23174	0.5	0.57239	0.792	0.32827	0.25346	0.31071	0.28696
0.796	0.35907	0.33607	0.28268	0.796	0.41434	0.23349	0.5	0.57362	0.796	0.33029	0.25393	0.31186	0.28775
0.8	0.3623	0.33874	0.2844	0.8	0.41569	0.23575	0.50036	0.57546	0.8	0.33223	0.2544	0.31317	0.28863
0.804	0.36433	0.34113	0.2857	0.804	0.41659	0.23751	0.5	0.57608	0.804	0.33433	0.25487	0.31432	0.28951
0.808	0.36716	0.3438	0.28678	0.808	0.41794	0.23927	0.5	0.57792	0.808	0.33651	0.25534	0.31601	0.29075

0.812	0.36999	0.3462	0.28807	0.812	0.41885	0.24103	0.49964	0.57977	0.812	0.33845	0.25582	0.31747	0.29189
0.816	0.37202	0.34886	0.28915	0.816	0.4202	0.24279	0.49964	0.581	0.816	0.34063	0.25676	0.31908	0.29321
0.82	0.37444	0.35073	0.29002	0.82	0.42065	0.24454	0.49928	0.58223	0.82	0.34233	0.25723	0.32077	0.29436
0.824	0.37606	0.35259	0.29045	0.824	0.4211	0.2458	0.49819	0.58346	0.824	0.34411	0.2577	0.32184	0.29559
0.828	0.37849	0.35526	0.29131	0.828	0.422	0.24756	0.49819	0.58469	0.828	0.34645	0.25912	0.32384	0.29717
0.832	0.38011	0.35739	0.29217	0.832	0.4229	0.24906	0.49819	0.58654	0.832	0.34831	0.26006	0.32599	0.29893
0.836	0.38213	0.35926	0.29261	0.836	0.42335	0.25057	0.49747	0.58777	0.836	0.35081	0.26054	0.32791	0.3007
0.84	0.38415	0.36139	0.29347	0.84	0.42426	0.25258	0.49783	0.58961	0.84	0.35291	0.26242	0.32998	0.3029
0.844	0.38618	0.36379	0.29433	0.844	0.42516	0.25409	0.49747	0.59146	0.844	0.35566	0.26337	0.33259	0.30519
0.848	0.3882	0.36592	0.29477	0.848	0.42651	0.25585	0.49711	0.59331	0.848	0.35833	0.26525	0.33505	0.30748
0.852	0.39022	0.36805	0.29563	0.852	0.42741	0.25735	0.49711	0.59454	0.852	0.36083	0.26667	0.33774	0.30985
0.856	0.39184	0.36992	0.29628	0.856	0.42831	0.25886	0.49674	0.59638	0.856	0.36334	0.26856	0.34019	0.31241
0.86	0.39387	0.37232	0.29714	0.86	0.42967	0.26037	0.49674	0.59823	0.86	0.3656	0.26997	0.3428	0.31478
0.864	0.39548	0.37418	0.29779	0.864	0.43057	0.26212	0.49674	0.60007	0.864	0.36843	0.27233	0.34557	0.31743
0.868	0.3971	0.37578	0.29843	0.868	0.43147	0.26363	0.49638	0.6013	0.868	0.37085	0.27375	0.34779	0.31927
0.872	0.39872	0.37792	0.2993	0.872	0.43282	0.26489	0.49638	0.60315	0.872	0.37352	0.27611	0.35086	0.322
0.876	0.40034	0.37978	0.30016	0.876	0.43417	0.26664	0.49638	0.605	0.876	0.3765	0.27847	0.35409	0.325
0.88	0.40196	0.38165	0.30103	0.88	0.43553	0.26815	0.49602	0.60623	0.88	0.37885	0.28035	0.3567	0.32782
0.884	0.40317	0.38298	0.30146	0.884	0.43643	0.26941	0.49566	0.60746	0.884	0.38119	0.28224	0.35939	0.33046
0.888	0.40439	0.38458	0.3021	0.888	0.43688	0.27091	0.4953	0.60869	0.888	0.38337	0.28366	0.36184	0.33283
0.892	0.406	0.38618	0.30297	0.892	0.43823	0.27217	0.49457	0.60992	0.892	0.3858	0.28602	0.3643	0.3353
0.896	0.40681	0.38778	0.30362	0.896	0.43913	0.27368	0.49421	0.61053	0.896	0.38782	0.28791	0.36737	0.33785
0.9	0.40762	0.38858	0.30405	0.9	0.43959	0.27468	0.49349	0.61115	0.9	0.38943	0.28979	0.36967	0.33997
0.904	0.40843	0.39018	0.30491	0.904	0.44049	0.27619	0.49276	0.61176	0.904	0.39129	0.29168	0.37198	0.34217
0.908	0.40965	0.39098	0.30577	0.908	0.44094	0.27744	0.49168	0.61238	0.908	0.39315	0.29357	0.37428	0.34472
0.912	0.41046	0.39204	0.30642	0.912	0.44139	0.27845	0.49096	0.61238	0.912	0.39452	0.29498	0.37658	0.34683
0.916	0.41086	0.39311	0.30685	0.916	0.44184	0.2797	0.48987	0.61238	0.916	0.39565	0.2964	0.37889	0.34868
0.92	0.41167	0.39391	0.30772	0.92	0.44184	0.28096	0.48878	0.61176	0.92	0.39662	0.29782	0.38042	0.35053
0.924	0.41288	0.39471	0.30836	0.924	0.44229	0.28222	0.4877	0.61115	0.924	0.39751	0.29923	0.38273	0.35221
0.928	0.41288	0.39551	0.30923	0.928	0.44229	0.28322	0.48625	0.61053	0.928	0.39832	0.30065	0.38426	0.35397
0.932	0.41369	0.39604	0.30988	0.932	0.44229	0.28423	0.48517	0.60992	0.932	0.39856	0.30159	0.3858	0.35546
0.936	0.4141	0.39684	0.31074	0.936	0.44229	0.28548	0.48408	0.60869	0.936	0.39921	0.30301	0.38733	0.35687
0.94	0.41491	0.39737	0.31182	0.94	0.44274	0.28674	0.483	0.60807	0.94	0.39945	0.30395	0.38887	0.35846
0.944	0.41531	0.39791	0.31268	0.944	0.44274	0.28799	0.48155	0.60684	0.944	0.39961	0.30537	0.38964	0.35995
0.948	0.41572	0.39844	0.31355	0.948	0.44274	0.289	0.48046	0.60561	0.948	0.39961	0.30631	0.39117	0.36136
0.952	0.41612	0.39897	0.31462	0.952	0.44229	0.29025	0.47902	0.60438	0.952	0.39969	0.30678	0.39194	0.3626
0.956	0.41693	0.3995	0.31549	0.956	0.44229	0.29176	0.47793	0.60315	0.956	0.39994	0.30772	0.39347	0.36427
0.96	0.41733	0.40004	0.31678	0.96	0.44229	0.29302	0.47685	0.6013	0.96	0.39985	0.30867	0.39424	0.36568
0.964	0.41774	0.4003	0.31786	0.964	0.44184	0.29427	0.4754	0.59946	0.964	0.39961	0.30961	0.39501	0.36682
0.968	0.41855	0.40084	0.31937	0.968	0.44184	0.29578	0.47467	0.59823	0.968	0.39961	0.31056	0.39655	0.36832
0.972	0.41895	0.40137	0.32067	0.972	0.44184	0.29729	0.47395	0.59638	0.972	0.39977	0.3115	0.39731	0.36982
0.976	0.41936	0.4019	0.32196	0.976	0.44184	0.29854	0.47287	0.59515	0.976	0.39977	0.31244	0.39808	0.37131
0.98	0.42017	0.40244	0.32348	0.98	0.44184	0.3003	0.47214	0.59331	0.98	0.4001	0.31339	0.39962	0.37316
0.984	0.42098	0.40297	0.3252	0.984	0.44184	0.30181	0.47142	0.59146	0.984	0.40002	0.31433	0.40038	0.37466
0.988	0.42138	0.4035	0.32671	0.988	0.44274	0.30356	0.47106	0.59023	0.988	0.40042	0.31528	0.40192	0.37633
0.992	0.42219	0.40404	0.32866	0.992	0.44274	0.30532	0.47069	0.58838	0.992	0.4009	0.31669	0.40269	0.378
0.996	0.423	0.40457	0.33038	0.996	0.44274	0.30683	0.46997	0.58715	0.996	0.40058	0.31716	0.40345	0.37959
1	0.42381	0.40537	0.33254	1	0.44364	0.30884	0.46961	0.58592	1	0.40139	0.31858	0.40576	0.38126
1.004	0.42462	0.40643	0.3347	1.004	0.44454	0.31085	0.46925	0.58469	1.004	0.4022	0.31999	0.40653	0.38329
1.008	0.42583	0.4075	0.33686	1.008	0.445	0.31311	0.46925	0.58346	1.008	0.40284	0.32141	0.40806	0.38522
1.012	0.42664	0.4083	0.33902	1.012	0.4459	0.31512	0.46889	0.58223	1.012	0.40389	0.32283	0.4096	0.38734
1.016	0.42826	0.40937	0.34139	1.016	0.4468	0.31738	0.46852	0.58162	1.016	0.40462	0.32424	0.41113	0.38936
1.02	0.42988	0.4107	0.3442	1.02	0.44815	0.31964	0.46889	0.581	1.02	0.40599	0.32613	0.41344	0.39165
1.024	0.4315	0.4123	0.347	1.024	0.44995	0.32215	0.46925	0.58039	1.024	0.40753	0.32849	0.41497	0.39394
1.028	0.43231	0.41336	0.34938	1.028	0.45131	0.32441	0.46925	0.57915	1.028	0.40866	0.3299	0.41727	0.39597
1.032	0.43392	0.41523	0.3524	1.032	0.45266	0.32717	0.46997	0.57977	1.032	0.41044	0.33179	0.41958	0.39852
1.036	0.43554	0.41736	0.35564	1.036	0.45491	0.33019	0.47069	0.57915	1.036	0.41214	0.33415	0.42188	0.40125
1.04	0.43757	0.41923	0.35888	1.04	0.45672	0.3332	0.47178	0.57915	1.04	0.41407	0.33651	0.42418	0.40416
1.044	0.43959	0.42136	0.36233	1.044	0.45897	0.33596	0.4725	0.57915	1.044	0.41618	0.33934	0.42649	0.40697
1.048	0.44161	0.42349	0.36579	1.048	0.46078	0.33923	0.47395	0.57977	1.048	0.41892	0.3417	0.42879	0.40997
1.052	0.44404	0.42589	0.36945	1.052	0.46393	0.34274	0.47504	0.58039	1.052	0.42159	0.345	0.43186	0.4134
1.056	0.44647	0.42856	0.37334	1.056	0.46664	0.34651	0.47685	0.58162	1.056	0.42458	0.34784	0.43493	0.41684
1.06	0.4489	0.43095	0.37701	1.06	0.46934	0.35003	0.47829	0.58223	1.06	0.42716	0.35114	0.43724	0.42036
1.064	0.45173	0.43389	0.38133	1.064	0.47295	0.35354	0.47974	0.58346	1.064	0.43007	0.35444	0.44107	0.42406
1.068	0.45456	0.43709	0.38564	1.068	0.4761	0.35781	0.48227	0.58469	1.068	0.43355	0.35822	0.44415	0.42811
1.072	0.4582	0.44002	0.39018	1.072	0.47971	0.36183	0.48444	0.58592	1.072	0.4371	0.36199	0.44798	0.43233
1.076	0.46144	0.44348	0.39471	1.076	0.48287	0.3661	0.48661	0.58777	1.076	0.44009	0.36577	0.45182	0.43629
1.08	0.46468	0.44641	0.39946	1.08	0.48647	0.37037	0.48915	0.58961	1.08	0.44413	0.36954	0.45566	0.44052
1.084	0.46832	0.45041	0.40443	1.084	0.49143	0.37539	0.4924	0.59208	1.084	0.44817	0.37426	0.4595	0.44528
1.088	0.47236	0.45468	0.40961	1.088	0.49549	0.37991	0.4953	0.59392	1.088	0.45221	0.37851	0.46411	0.44968
1.092	0.47601	0.45841	0.41457	1.092	0.5	0.38468	0.49783	0.59638	1.092	0.45625	0.38276	0.46795	0.45408
1.096	0.48005	0.46267	0.41997	1.096	0.50496	0.38971	0.50145	0.59884	1.096	0.4611	0.38795	0.47255	0.45936
1.1	0.4845	0.46747	0.42558	1.1	0.50992	0.39523	0.5047	0.60192	1.1	0.46514	0.39267	0.47716	0.46377
1.104	0.48855	0.472	0.43119	1.104	0.51488	0.40051	0.50796	0.60438	1.104	0.46998	0.39786	0.48177	0.46905
1.108	0.4934	0.4768	0.43681	1.108	0.52029	0.40603	0.51158	0.60746	1.108	0.47483	0.40352	0.48714	0.47433
1.112	0.49826	0.48186	0.44263	1.112	0.5257	0.41206	0.51556	0.61053	1.112	0.48049	0.40871	0.49251	0.47962
1.116	0.50312	0.48693	0.44889	1.116	0.53156	0.41809	0.51954	0.61361	1.116	0.48534	0.41485	0.49712	0.4849
1.12	0.50797	0.49279	0.45515	1.12	0.53787	0.42437	0.52424	0.61792	1.12	0.49099	0.42051	0.50326	0.49106
1.124	0.51323	0.49812	0.46163	1.124	0.54373	0.43039	0.52822	0.62099	1.124	0.49745	0.42664	0.50864	0.49635
1.128	0.5189	0.50398	0.46832	1.128	0.5505	0.43718	0.53292	0.6253	1.128	0.50311	0.43278	0.51478	0.50251
1.132	0.52497	0.51011	0.47523	1.132	0.55681	0.44421	0.53799	0.62899	1.132	0.50957	0.43986	0.52092	0.50867
1.136	0.53063	0.51678	0.48214	1.136	0.56357	0.45099	0.54269	0.6333	1.136	0.51604	0.44646	0.52706	0.51572
1.14	0.5363	0.52317	0.48904	1.14	0.57033	0.45827	0.54776	0.63761	1.14	0.5225	0.45354	0.53321	0.52188
1.144	0.54277	0.52984	0.49617	1.144	0.5771	0.46581	0.55318	0.64191	1.144	0.52897	0.46109	0.53935	0.52892
1.148	0.54924	0.53703	0.50394	1.148	0.58521	0.47359	0.55933	0.64745	1.148	0.53624	0.46911	0.54626	0.53685
1.152	0.55612	0.54423	0.51214	1.152	0.59288	0.48188	0.56512	0.65237	1.152	0.54432	0.47714	0.55393	0.54477
1.156	0.563	0.55143	0.5197	1.156	0.60009	0.48992	0.57127	0.65729	1.156	0.55159	0.48516	0.56084	0.5527
1.16	0.57028	0.55862	0.5279	1.16	0.60821	0.49871	0.57815	0.66222	1.16	0.55886	0.49365	0.56852	0.55974
1.164	0.57757	0.56662	0.53654	1.164	0.61677	0.5075	0.58502	0.66775	1.164	0.56694	0.50262	0.5762	0.56855
1.168	0.58526	0.57462	0.54517	1.168	0.62534	0.51654	0.5919	0.67391	1.168	0.57583	0.51159	0.58464	0.57647
1.172	0.59294	0.58288	0.55402	1.172	0.6339	0.52583	0.59913	0.67944	1.172	0.58391	0.52055	0.59232	0.58528
1.176	0.60063	0.59141	0.5633	1.176	0.64247	0.53537	0.60673	0.6856	1.176	0.59279	0.53046	0.60077	0.59408
1.18	0.60913	0.60047	0.57258	1.18	0.65239	0.54542	0.61505	0.69236	1.18	0.60168	0.54037	0.60921	0.60289
1.184	0.61843	0.61033	0.58273	1.184	0.66321	0.55597	0.62373	0.69975	1.184	0.61218	0.55122	0.61919	0.61345
1.188	0.62653	0.61939	0.59223	1.188	0.67268	0.56652	0.63169	0.7059	1.188	0.62188	0.56113	0.62841	0.62314
1.192	0.63583	0.62899	0.60237	1.192	0.68305	0.57732	0.64038	0.71328	1.192	0.63077	0.57199	0.63762	0.63283
1.196	0.64474	0.63912	0.61274	1.196	0.69342	0.58837	0.64942	0.72067	1.196	0.64127	0.58331	0.64683	0.64251
1.2	0.65445	0.64924	0.62353	1.2	0.70379	0.59967	0.65883	0.72805	1.2	0.65177	0.59511	0.65681	0.65308
1.204	0.66375	0.65964	0.63432	1.204	0.71416	0.61097	0.66823	0.73543	1.204	0.66228	0.60596	0.66603	0.66364
1.208	0.67427	0.67003	0.64533	1.208	0.72543	0.62277	0.678	0.74343	1.208	0.67278	0.61776	0.67678	0.67421
1.212	0.68479	0.68123	0.65677	1.212	0.73715	0.63508	0.68813	0.75143	1.212	0.68328	0.63003	0.68752	0.68566
1.216	0.69531	0.69296	0.66843	1.216	0.74887	0.64814	0.69863	0.75943	1.216	0.69459	0.64324	0.69904	0.6971
1.22	0.70624	0.70388	0.6803	1.22	0.7606	0.66095	0.70912	0.76804	1.22	0.70591	0.65599	0.70979	0.70855

1.224	0.71757	0.71588	0.69282	1.224	0.77322	0.67451	0.72069	0.77727	1.224	0.71803	0.66967	0.72131	0.72
1.228	0.7293	0.72787	0.70556	1.228	0.78584	0.68832	0.73227	0.7865	1.228	0.73014	0.68336	0.73359	0.73232
1.232	0.74104	0.74013	0.71873	1.232	0.79892	0.70239	0.74421	0.79573	1.232	0.74226	0.69751	0.74587	0.74377
1.236	0.75277	0.75239	0.73168	1.236	0.81199	0.71645	0.75579	0.80496	1.236	0.75519	0.71167	0.75739	0.75698
1.24	0.76532	0.76545	0.74528	1.24	0.82552	0.73127	0.76809	0.8148	1.24	0.76812	0.7263	0.77044	0.76931
1.244	0.77826	0.77878	0.75953	1.244	0.8404	0.74609	0.78075	0.82526	1.244	0.78185	0.7414	0.78349	0.78251
1.248	0.79162	0.79264	0.77442	1.248	0.85482	0.76191	0.79414	0.83634	1.248	0.79478	0.75697	0.79655	0.7966
1.252	0.80497	0.80596	0.78888	1.252	0.86925	0.77773	0.80753	0.8468	1.252	0.80852	0.77254	0.8096	0.80893
1.256	0.81913	0.82062	0.80443	1.256	0.88368	0.79406	0.82127	0.85787	1.256	0.82306	0.78906	0.82418	0.8239
1.26	0.8337	0.83528	0.82018	1.26	0.89946	0.81063	0.83611	0.86956	1.26	0.8376	0.80605	0.838	0.83887
1.264	0.84867	0.85021	0.83594	1.264	0.91524	0.82771	0.85058	0.88125	1.264	0.85215	0.82304	0.85182	0.85383
1.268	0.86404	0.8654	0.85235	1.268	0.93102	0.84504	0.86541	0.89294	1.268	0.86669	0.84097	0.86718	0.86792
1.272	0.87902	0.88113	0.8694	1.272	0.9477	0.86287	0.88061	0.90463	1.272	0.88204	0.85937	0.88253	0.88377
1.276	0.8952	0.89739	0.88667	1.276	0.96483	0.88146	0.89653	0.91694	1.276	0.89901	0.87778	0.89789	0.89962
1.28	0.91179	0.91364	0.90437	1.28	0.98242	0.90029	0.91317	0.93047	1.28	0.91436	0.89713	0.91401	0.91547
1.284	0.92798	0.9299	0.9225	1.284	1	0.91863	0.92945	0.9434	1.284	0.93052	0.91647	0.93013	0.93132
1.288	0.94538	0.94723	0.94172	1.288	--	0.93922	0.94682	0.95816	1.288	0.94829	0.93677	0.94702	0.94893
1.292	0.96358	0.96455	0.96071	1.292	--	0.95906	0.96418	0.9717	1.292	0.96526	0.95753	0.96468	0.96654
1.296	0.98139	0.98214	0.98014	1.296	--	0.97966	0.98191	0.98585	1.296	0.98223	0.97829	0.98234	0.98327
1.3	1	1	1	1.3	--	1	1	1	1.3	1	1	1	1

**Water-ABS (Spring)**				**Water-ABS (Summer)**					**Water-ABS (Autumn)**				
0.004	0.07595	0.06754	0.08394	0.004	0.12661	0.10357	0.12237	0	0.004	0.08969	0.05695	0.03491	0.02975
0.008	0.07006	0.06324	0.07819	0.008	0.11982	0.09803	0.11297	1.50E-04	0.008	0.08342	0.05423	0.03095	0.02739
0.012	0.06537	0.05973	0.07322	0.012	0.11444	0.09283	0.10571	2.50E-04	0.012	0.07838	0.0515	0.02668	0.02528
0.016	0.06162	0.05671	0.0693	0.016	0.10935	0.08798	0.09973	3.25E-04	0.016	0.07436	0.04908	0.0249	0.02377
0.02	0.05808	0.05448	0.06616	0.02	0.10511	0.08348	0.09503	4.25E-04	0.02	0.07096	0.04635	0.02338	0.02232
0.024	0.05537	0.05225	0.06328	0.024	0.10086	0.07932	0.09075	4.75E-04	0.024	0.0683	0.04453	0.02175	0.02106
0.028	0.05298	0.05065	0.06093	0.028	0.09775	0.07586	0.08691	6.50E-04	0.028	0.06604	0.04302	0.02018	0.01993
0.032	0.05074	0.04922	0.05884	0.032	0.09464	0.07239	0.08349	8.00E-04	0.032	0.06403	0.0415	0.01891	0.01895
0.036	0.04891	0.04795	0.05672	0.036	0.09153	0.06893	0.0805	8.00E-04	0.036	0.06231	0.03999	0.01733	0.01804
0.04	0.04719	0.04651	0.05499	0.04	0.08898	0.06581	0.07794	9.00E-04	0.04	0.06088	0.03847	0.01596	0.01706
0.044	0.04568	0.04572	0.05361	0.044	0.08643	0.06304	0.07537	0.00103	0.044	0.05952	0.03696	0.01484	0.01621
0.048	0.04417	0.0446	0.05178	0.048	0.08389	0.06027	0.07324	0.00108	0.048	0.05829	0.03605	0.01464	0.01558
0.052	0.04277	0.04364	0.05081	0.052	0.08162	0.0575	0.07153	0.00108	0.052	0.05715	0.03484	0.01362	0.01483
0.056	0.04141	0.04269	0.04932	0.056	0.07964	0.05438	0.06982	0.0011	0.056	0.05616	0.03363	0.01199	0.01417
0.06	0.04048	0.04205	0.04869	0.06	0.07795	0.0523	0.06811	0.00128	0.06	0.05534	0.03272	0.01235	0.0136
0.064	0.03933	0.04126	0.04783	0.064	0.07597	0.04953	0.0664	0.00123	0.064	0.05473	0.03181	0.01133	0.01287
0.068	0.03824	0.0403	0.04673	0.068	0.07427	0.04711	0.06512	0.0012	0.068	0.05382	0.0309	0.01032	0.01225
0.072	0.03709	0.03966	0.04589	0.072	0.07229	0.04468	0.06332	0.00118	0.072	0.05296	0.02999	0.00996	0.01155
0.076	0.03605	0.03903	0.04524	0.076	0.07059	0.0426	0.06221	0.00123	0.076	0.05198	0.02878	0.00905	0.01108
0.08	0.03516	0.03823	0.04443	0.08	0.06861	0.04053	0.0608	0.00115	0.08	0.05116	0.02817	0.00864	0.0102
0.084	0.03417	0.03759	0.04354	0.084	0.06691	0.03845	0.05948	0.00115	0.084	0.05046	0.02726	0.00849	0.00951
0.088	0.03318	0.03664	0.04276	0.088	0.06521	0.03637	0.05832	0.00108	0.088	0.04973	0.02605	0.00783	0.00885
0.092	0.03251	0.036	0.04184	0.092	0.06352	0.03395	0.05708	0.00103	0.092	0.04882	0.02514	0.00752	0.00822
0.096	0.03162	0.03536	0.04095	0.096	0.0621	0.03221	0.05589	9.75E-04	0.096	0.04817	0.02454	0.00752	0.00759
0.1	0.03063	0.03472	0.03988	0.1	0.06012	0.03014	0.05414	9.25E-04	0.1	0.04759	0.02363	0.00717	0.00693
0.104	0.02985	0.03393	0.03904	0.104	0.05871	0.0284	0.0529	8.25E-04	0.104	0.04657	0.02242	0.00691	0.0063
0.108	0.0287	0.03329	0.03768	0.108	0.05673	0.02632	0.05149	7.50E-04	0.108	0.04571	0.02151	0.0062	0.0057
0.112	0.02776	0.03249	0.03648	0.112	0.05503	0.02459	0.05012	7.00E-04	0.112	0.04481	0.0206	0.006	0.00526
0.116	0.02667	0.03186	0.03549	0.116	0.05333	0.02286	0.04867	6.75E-04	0.116	0.04395	0.01969	0.00569	0.0046
0.12	0.02568	0.03106	0.03462	0.12	0.05163	0.02113	0.04739	5.00E-04	0.12	0.04304	0.01848	0.00493	0.00403
0.124	0.0248	0.03026	0.03376	0.124	0.04996	0.01974	0.04602	4.50E-04	0.124	0.04214	0.01787	0.00442	0.00346
0.128	0.02375	0.02963	0.0329	0.128	0.04815	0.01801	0.04457	4.50E-04	0.128	0.04124	0.01696	0.00391	0.00302
0.132	0.02256	0.02883	0.03188	0.132	0.0464	0.01663	0.04277	4.00E-04	0.132	0.03993	0.01575	0.00325	0.00246
0.136	0.02146	0.02803	0.03096	0.136	0.04462	0.01524	0.04132	3.25E-04	0.136	0.03894	0.01515	0.00295	0.00211
0.14	0.02058	0.02724	0.02986	0.14	0.04283	0.01386	0.03995	3.25E-04	0.14	0.03784	0.01424	0.00244	0.00161
0.144	0.01927	0.02628	0.02884	0.144	0.04131	0.01247	0.0382	4.75E-04	0.144	0.03653	0.01363	0.00183	0.00139
0.148	0.01834	0.02565	0.02759	0.148	0.03938	0.01143	0.03636	3.75E-04	0.148	0.0353	0.01303	0.00173	0.00104
0.152	0.01735	0.02469	0.02649	0.152	0.03749	0.01005	0.03457	3.50E-04	0.152	0.03411	0.01242	9.15E-04	7.87E-04
0.156	0.0163	0.02389	0.02555	0.156	0.03573	0.00935	0.03273	4.25E-04	0.156	0.03263	0.01181	7.62E-04	5.04E-04
0.16	0.01537	0.02294	0.02445	0.16	0.03418	0.00797	0.03123	7.50E-04	0.16	0.03128	0.01121	7.11E-04	3.15E-04
0.164	0.01417	0.02198	0.02327	0.164	0.03234	0.00693	0.0294	7.75E-04	0.164	0.03001	0.0106	3.05E-04	1.57E-04
0.168	0.01318	0.02119	0.02212	0.168	0.03084	0.00623	0.02786	8.75E-04	0.168	0.02849	0.00969	3.56E-04	0
0.172	0.01224	0.02039	0.02082	0.172	0.02903	0.00554	0.02602	0.00108	0.172	0.02701	0.00909	5.08E-04	9.44E-05
0.176	0.01136	0.01943	0.01951	0.176	0.02716	0.0045	0.02423	0.00145	0.176	0.02554	0.00788	5.08E-04	2.52E-04
0.18	0.01021	0.01848	0.01828	0.18	0.02532	0.00381	0.02265	0.00163	0.18	0.02402	0.00727	4.57E-04	2.52E-04
0.184	0.00932	0.01752	0.01721	0.184	0.02377	0.00312	0.02111	0.0019	0.184	0.02234	0.00666	0	1.89E-04
0.188	0.00833	0.01641	0.01616	0.188	0.02215	0.00277	0.01948	0.0021	0.188	0.02091	0.00606	5.08E-04	3.15E-04
0.192	0.0075	0.01545	0.01527	0.192	0.02048	0.00242	0.01782	0.00245	0.192	0.01951	0.00545	2.54E-04	5.35E-04
0.196	0.00646	0.0145	0.01412	0.196	0.01864	0.00173	0.01615	0.00265	0.196	0.01783	0.00454	7.62E-04	6.93E-04
0.2	0.00573	0.01354	0.013	0.2	0.01703	0.00104	0.01466	0.00305	0.2	0.01668	0.00394	6.61E-04	9.13E-04
0.204	0.0051	0.01242	0.01192	0.204	0.01545	0.00104	0.01303	0.00323	0.204	0.01509	0.00333	0.00152	0.00116
0.208	0.00427	0.01163	0.01093	0.208	0.01389	6.93E-04	0.01137	0.00368	0.208	0.01369	0.00273	0.00142	0.00148
0.212	0.00349	0.01035	0.0097	0.212	0.01225	3.46E-04	0.00978	0.00413	0.212	0.0123	0.00212	0.00183	0.00186
0.216	0.00292	0.0094	0.00858	0.216	0.01064	0	0.00825	0.00453	0.216	0.0109	6.06E-04	0.00208	0.0023
0.22	0.0024	0.0086	0.00758	0.22	0.00928	0	0.00671	0.00508	0.22	0.00967	3.03E-04	0.0029	0.00283
0.224	0.00167	0.00733	0.00654	0.224	0.00781	0	0.00551	0.0057	0.224	0.00828	0	0.00361	0.00362
0.228	0.00109	0.00637	0.00541	0.228	0.00631	0	0.00414	0.00628	0.228	0.00693	3.03E-04	0.00407	0.00447
0.232	7.29E-04	0.00542	0.00445	0.232	0.00504	3.46E-04	0.00299	0.0071	0.232	0.00586	6.06E-04	0.00452	0.0056
0.236	4.17E-04	0.00446	0.00366	0.236	0.0039	6.93E-04	0.00209	0.00798	0.236	0.00476	6.06E-04	0.00584	0.00696
0.24	2.08E-04	0.00366	0.00272	0.24	0.00291	0.00139	9.83E-04	0.009	0.24	0.00361	0.00121	0.00696	0.00869
0.244	5.21E-05	0.00271	0.00201	0.244	0.00173	0.00208	4.27E-04	0.01023	0.244	0.00262	0.00182	0.00828	0.01048
0.248	0	0.00207	0.00136	0.248	9.34E-04	0.00312	0	0.01156	0.248	0.00184	0.00273	0.00961	0.01287
0.252	2.08E-04	0.00127	7.58E-04	0.252	4.53E-04	0.0045	2.56E-04	0.01333	0.252	0.00127	0.00454	0.01123	0.01571
0.256	3.13E-04	6.37E-04	4.18E-04	0.256	1.41E-04	0.00589	3.85E-04	0.01531	0.256	6.15E-04	0.00636	0.01311	0.01898
0.26	5.21E-04	3.19E-04	0	0.26	0	0.00762	0.00111	0.01753	0.26	2.05E-04	0.00879	0.01535	0.0227
0.264	0.0012	0	0	0.264	1.13E-04	0.01005	0.00214	0.02036	0.264	0	0.01181	0.01779	0.02868
0.268	0.00193	0	1.83E-04	0.268	6.22E-04	0.01247	0.0038	0.02356	0.268	0	0.01515	0.02058	0.03331
0.272	0.00266	1.59E-04	5.49E-04	0.272	0.00127	0.01524	0.00598	0.02721	0.272	3.28E-04	0.01909	0.02368	0.03907
0.276	0.00385	4.78E-04	0.00107	0.276	0.00235	0.01836	0.00902	0.03154	0.276	7.79E-04	0.02393	0.02678	0.04555
0.28	0.0049	0.00112	0.00212	0.28	0.00419	0.02182	0.0129	0.03662	0.28	0.00176	0.02999	0.03131	0.05307
0.284	0.00656	0.00207	0.00343	0.284	0.0062	0.02563	0.01765	0.04245	0.284	0.00303	0.03696	0.03598	0.0617
0.288	0.00844	0.0035	0.00547	0.288	0.00905	0.03014	0.02359	0.04905	0.288	0.00476	0.04483	0.04101	0.07143
0.292	0.01094	0.00558	0.00785	0.292	0.01234	0.03533	0.03029	0.0566	0.292	0.00709	0.05362	0.04676	0.08216
0.296	0.01375	0.00781	0.01075	0.296	0.01647	0.04122	0.03828	0.06523	0.296	0.00976	0.06362	0.05321	0.09415
0.3	0.01719	0.01099	0.01444	0.3	0.02125	0.0478	0.04777	0.07506	0.3	0.01299	0.07513	0.06032	0.10775
0.304	0.02136	0.01465	0.01891	0.304	0.02696	0.05507	0.05879	0.08602	0.304	0.01685	0.08755	0.06846	0.12249
0.308	0.02615	0.01911	0.02406	0.308	0.03375	0.06339	0.07067	0.0981	0.308	0.02156	0.10148	0.07755	0.13867
0.312	0.03183	0.02469	0.02989	0.312	0.04111	0.07274	0.08434	0.11163	0.312	0.02673	0.11603	0.08751	0.15636
0.316	0.0387	0.03106	0.03672	0.316	0.04977	0.08313	0.09973	0.12639	0.316	0.03279	0.13238	0.09869	0.17587
0.32	0.04667	0.03839	0.04448	0.32	0.05956	0.09491	0.11639	0.14239	0.32	0.03956	0.14995	0.11094	0.19634

0.324	0.05579	0.04683	0.05311	0.324	0.07002	0.10772	0.13391	0.15938	0.324	0.04727	0.16843	0.12405	0.21774
0.328	0.06657	0.05655	0.06302	0.328	0.08219	0.12158	0.15271	0.17786	0.328	0.056	0.18812	0.13859	0.24072
0.332	0.07866	0.06754	0.07401	0.332	0.0952	0.13647	0.17194	0.19712	0.332	0.06571	0.20812	0.15358	0.26433
0.336	0.09194	0.0798	0.08604	0.336	0.10907	0.15241	0.19202	0.21738	0.336	0.07604	0.22902	0.16994	0.28826
0.34	0.10668	0.09334	0.09911	0.34	0.12406	0.16903	0.21253	0.23814	0.34	0.08719	0.25053	0.18692	0.31249
0.344	0.12304	0.10831	0.11376	0.344	0.14019	0.18635	0.23218	0.2594	0.344	0.09892	0.27204	0.20471	0.33642
0.348	0.14075	0.12488	0.12945	0.348	0.15745	0.20471	0.25184	0.28141	0.348	0.1113	0.29355	0.22254	0.36003
0.352	0.15971	0.14288	0.14671	0.352	0.17556	0.22307	0.27021	0.30342	0.352	0.12433	0.31536	0.24074	0.38301
0.356	0.18008	0.16231	0.16501	0.356	0.19451	0.24143	0.28773	0.32568	0.356	0.13716	0.33656	0.25853	0.40473
0.36	0.20201	0.18382	0.18488	0.36	0.21516	0.26082	0.30439	0.34844	0.36	0.15041	0.35807	0.2758	0.42613
0.364	0.22618	0.20771	0.20764	0.364	0.23752	0.28126	0.32106	0.3722	0.364	0.16414	0.3814	0.29359	0.44691
0.368	0.25212	0.23367	0.23169	0.368	0.26156	0.30204	0.33601	0.39696	0.368	0.17767	0.40351	0.31036	0.46674
0.372	0.28062	0.26219	0.25837	0.372	0.28703	0.32283	0.35054	0.42348	0.372	0.19078	0.42684	0.32662	0.48626
0.376	0.31291	0.29388	0.28818	0.376	0.31532	0.34603	0.36549	0.45249	0.376	0.20431	0.45168	0.3439	0.50672
0.38	0.34886	0.3294	0.32165	0.38	0.34673	0.37201	0.38173	0.48476	0.38	0.21866	0.47925	0.3622	0.52844
0.384	0.38949	0.36907	0.35983	0.384	0.38181	0.40111	0.39882	0.52027	0.384	0.23383	0.50924	0.38151	0.55268
0.388	0.43481	0.41255	0.40167	0.388	0.42057	0.43298	0.41762	0.55929	0.388	0.25023	0.54196	0.40286	0.57881
0.392	0.48482	0.46113	0.44874	0.392	0.46386	0.46865	0.43941	0.60256	0.392	0.26826	0.57861	0.42573	0.60903
0.396	0.53899	0.51354	0.5	0.396	0.5111	0.50779	0.46334	0.64933	0.396	0.28876	0.6189	0.45114	0.64303
0.4	0.59577	0.56977	0.55544	0.4	0.56203	0.55005	0.48983	0.69861	0.4	0.31131	0.66253	0.47858	0.67986
0.404	0.65463	0.62886	0.61349	0.404	0.61635	0.59404	0.51846	0.74938	0.404	0.33631	0.70857	0.50704	0.71952
0.408	0.71402	0.68923	0.6739	0.408	0.67266	0.63907	0.54922	0.80066	0.408	0.36337	0.75583	0.53702	0.76076
0.412	0.7734	0.7496	0.73483	0.412	0.73009	0.6841	0.58084	0.85018	0.412	0.39247	0.804	0.56701	0.80294
0.416	0.82966	0.80806	0.79419	0.416	0.78639	0.72601	0.61246	0.8962	0.416	0.42281	0.84974	0.59597	0.84355
0.42	0.88071	0.86222	0.8499	0.42	0.84015	0.76411	0.64237	0.93597	0.42	0.45396	0.89276	0.6224	0.88006
0.424	0.92447	0.91048	0.89984	0.424	0.88966	0.79633	0.67057	0.96773	0.424	0.48553	0.93093	0.64629	0.9128
0.428	0.96041	0.9503	0.94195	0.428	0.9321	0.82092	0.6945	0.98975	0.428	0.51586	0.96274	0.66611	0.9383
0.432	0.98489	0.97961	0.97359	0.432	0.96577	0.83685	0.71372	1	0.432	0.54456	0.98546	0.68135	0.9553
0.436	0.99844	0.9965	0.9932	0.436	0.9884	0.84309	0.7274	0.99775	0.436	0.57039	0.99788	0.68999	0.96317
0.44	1	1	1	0.44	1	0.83928	0.73338	0.98374	0.44	0.59211	1	0.69253	0.9616
0.444	0.9901	0.99012	0.99372	0.444	1	0.82646	0.73295	0.95848	0.444	0.60892	0.99152	0.68898	0.95058
0.448	0.97083	0.96751	0.9749	0.448	0.98755	0.80464	0.72569	0.92346	0.448	0.62122	0.97304	0.68034	0.93075
0.452	0.94166	0.93294	0.9443	0.452	0.96463	0.77589	0.71159	0.88069	0.452	0.62778	0.94547	0.66662	0.90336
0.456	0.90676	0.88946	0.9056	0.456	0.93295	0.74195	0.69279	0.83342	0.456	0.6286	0.91124	0.64934	0.87094
0.46	0.86769	0.83912	0.85983	0.46	0.89419	0.70419	0.66929	0.7839	0.46	0.62409	0.87186	0.63002	0.83442
0.464	0.82601	0.78433	0.80962	0.464	0.85005	0.66436	0.64237	0.73437	0.464	0.61507	0.82914	0.60919	0.79539
0.468	0.78434	0.72874	0.75785	0.468	0.80365	0.62314	0.61417	0.6866	0.468	0.60236	0.78491	0.58784	0.75667
0.472	0.74319	0.67299	0.70607	0.472	0.75555	0.58227	0.58469	0.64183	0.472	0.58637	0.73978	0.56752	0.71826
0.476	0.70412	0.61978	0.65586	0.476	0.70802	0.54243	0.55478	0.60106	0.476	0.56834	0.69615	0.5477	0.68143
0.48	0.66818	0.56993	0.60853	0.48	0.66162	0.50329	0.52529	0.56404	0.48	0.54907	0.65374	0.5294	0.64617
0.484	0.6338	0.52373	0.56433	0.484	0.6172	0.46692	0.49667	0.53128	0.484	0.52857	0.61315	0.51212	0.61375
0.488	0.60358	0.48216	0.52406	0.488	0.57561	0.43228	0.46975	0.50226	0.488	0.50808	0.57498	0.49687	0.58416
0.492	0.57545	0.44457	0.48771	0.492	0.53713	0.40007	0.44454	0.47675	0.492	0.48799	0.54044	0.48264	0.55677
0.496	0.55045	0.41112	0.45502	0.496	0.50177	0.37063	0.42061	0.45449	0.496	0.46831	0.50833	0.46943	0.53222
0.5	0.52753	0.38149	0.42573	0.5	0.46951	0.34361	0.39882	0.43523	0.5	0.44945	0.47895	0.45876	0.51018
0.504	0.50669	0.35537	0.39984	0.504	0.44037	0.31936	0.37959	0.41822	0.504	0.43183	0.45198	0.44859	0.49035
0.508	0.48794	0.33259	0.37735	0.508	0.41434	0.29754	0.36207	0.40347	0.508	0.41502	0.42866	0.43996	0.47335
0.512	0.47127	0.31252	0.35748	0.512	0.39114	0.27814	0.34627	0.39046	0.512	0.40026	0.40745	0.43233	0.45793
0.516	0.45564	0.29468	0.3397	0.516	0.37049	0.26082	0.33217	0.37896	0.516	0.38591	0.38867	0.42522	0.44439
0.52	0.4421	0.27955	0.32427	0.52	0.35238	0.24593	0.32063	0.3692	0.52	0.37362	0.37292	0.41912	0.43306
0.524	0.4296	0.26617	0.31119	0.524	0.33682	0.23277	0.31037	0.3607	0.524	0.36255	0.35898	0.41454	0.4233
0.528	0.41866	0.25438	0.29995	0.528	0.32324	0.22134	0.30183	0.35344	0.528	0.35271	0.34717	0.40997	0.41512
0.532	0.40824	0.24435	0.29027	0.532	0.31136	0.21164	0.29457	0.34694	0.532	0.3441	0.33717	0.40641	0.40819
0.536	0.39886	0.23543	0.2819	0.536	0.30146	0.20333	0.28901	0.34169	0.536	0.33672	0.32899	0.40336	0.40221
0.54	0.39001	0.22778	0.27484	0.54	0.29325	0.19605	0.28431	0.33744	0.54	0.33057	0.32293	0.40082	0.3978
0.544	0.3822	0.22077	0.26883	0.544	0.28618	0.19016	0.28089	0.33368	0.544	0.32565	0.31778	0.39879	0.39403
0.548	0.37438	0.21488	0.26386	0.548	0.27995	0.18531	0.27833	0.33043	0.548	0.32114	0.31354	0.39727	0.39056
0.552	0.36761	0.20962	0.25968	0.552	0.27514	0.18081	0.27705	0.32818	0.552	0.31827	0.31112	0.39574	0.38836
0.556	0.36136	0.205	0.25601	0.556	0.27147	0.17769	0.27619	0.32643	0.556	0.31582	0.3093	0.39472	0.38647
0.56	0.35459	0.20086	0.25314	0.56	0.26807	0.17492	0.27619	0.32518	0.56	0.31418	0.30809	0.39422	0.38521
0.564	0.34834	0.1972	0.25105	0.564	0.26553	0.1725	0.27662	0.32418	0.564	0.31295	0.30779	0.39371	0.38458
0.568	0.34208	0.19369	0.24895	0.568	0.26355	0.17077	0.2779	0.32393	0.568	0.31254	0.30839	0.3932	0.38364
0.572	0.33688	0.19098	0.24765	0.572	0.2627	0.16938	0.28004	0.32393	0.572	0.31295	0.30991	0.39269	0.38364
0.576	0.33115	0.18844	0.24634	0.576	0.26156	0.16834	0.28175	0.32418	0.576	0.31377	0.31112	0.39269	0.38395
0.58	0.32542	0.18573	0.24555	0.58	0.26072	0.16765	0.28431	0.32443	0.58	0.315	0.31233	0.39218	0.38364
0.584	0.31969	0.18366	0.24503	0.584	0.26072	0.16696	0.28687	0.32518	0.584	0.31664	0.31445	0.39218	0.38427
0.588	0.31448	0.18175	0.24477	0.588	0.26072	0.16661	0.28944	0.32593	0.588	0.31827	0.31657	0.39218	0.38458
0.592	0.30927	0.17999	0.24425	0.592	0.261	0.16661	0.292	0.32693	0.592	0.31991	0.31869	0.39218	0.38521
0.596	0.30458	0.17824	0.24399	0.596	0.261	0.16626	0.29499	0.32768	0.596	0.32196	0.32081	0.39218	0.38553
0.6	0.29937	0.17697	0.24399	0.6	0.26156	0.16626	0.29798	0.32868	0.6	0.32442	0.32293	0.39218	0.38616
0.604	0.29468	0.17585	0.24425	0.604	0.26241	0.16661	0.30097	0.32993	0.604	0.32647	0.32505	0.39218	0.38679
0.608	0.29051	0.17474	0.24451	0.608	0.26326	0.16661	0.30354	0.33093	0.608	0.32893	0.32717	0.39269	0.38741
0.612	0.28635	0.17378	0.24477	0.612	0.26383	0.16696	0.3061	0.33193	0.612	0.33057	0.32899	0.39218	0.38773
0.616	0.28218	0.17314	0.24555	0.616	0.26468	0.1673	0.30867	0.33293	0.616	0.33303	0.33081	0.39269	0.38836
0.62	0.27853	0.17251	0.24608	0.62	0.26581	0.16799	0.31166	0.33368	0.62	0.33508	0.33263	0.3932	0.38867
0.624	0.27483	0.17219	0.24686	0.624	0.26694	0.16834	0.31422	0.33443	0.624	0.33713	0.33444	0.3932	0.38899
0.628	0.27155	0.17171	0.24738	0.628	0.26779	0.16903	0.31636	0.33518	0.628	0.33959	0.33566	0.39371	0.38899
0.632	0.26832	0.17155	0.24817	0.632	0.26864	0.16938	0.31849	0.33593	0.632	0.34082	0.33717	0.39371	0.3893
0.636	0.26567	0.17139	0.24895	0.636	0.26977	0.17007	0.32063	0.33668	0.636	0.34287	0.33838	0.39422	0.38962
0.64	0.26296	0.17155	0.25	0.64	0.27118	0.17077	0.32234	0.33719	0.64	0.34451	0.3399	0.39523	0.38993
0.644	0.26035	0.17155	0.25105	0.644	0.27175	0.17146	0.32405	0.33769	0.644	0.34615	0.34081	0.39523	0.38962
0.648	0.25848	0.17171	0.25209	0.648	0.27316	0.17215	0.32576	0.33794	0.648	0.34779	0.34202	0.39574	0.38993
0.652	0.25634	0.17203	0.2534	0.652	0.2743	0.1725	0.32747	0.33844	0.652	0.34943	0.34323	0.39676	0.38993
0.656	0.25457	0.17219	0.25445	0.656	0.27543	0.17319	0.32875	0.33869	0.656	0.35066	0.34384	0.39727	0.38993
0.66	0.2527	0.17267	0.25575	0.66	0.27656	0.17423	0.33046	0.33869	0.66	0.3523	0.34444	0.39777	0.38962
0.664	0.25119	0.17299	0.2568	0.664	0.27769	0.17492	0.33217	0.33894	0.664	0.35353	0.34505	0.39828	0.38962
0.668	0.24993	0.17362	0.25785	0.668	0.27882	0.17596	0.33345	0.33894	0.668	0.35476	0.34626	0.3993	0.38993
0.672	0.24895	0.1741	0.25941	0.672	0.28052	0.177	0.33558	0.33944	0.672	0.3564	0.34717	0.40032	0.39025
0.676	0.24775	0.17474	0.26072	0.676	0.28137	0.17804	0.33644	0.33944	0.676	0.35722	0.34777	0.40133	0.39025
0.68	0.24702	0.17537	0.26203	0.68	0.28278	0.17943	0.33772	0.33944	0.68	0.35845	0.34838	0.40235	0.39056
0.684	0.24644	0.17601	0.2636	0.684	0.2842	0.18046	0.33943	0.33944	0.684	0.35968	0.34959	0.40286	0.39088
0.688	0.24592	0.17697	0.26464	0.688	0.28561	0.1822	0.34028	0.33944	0.688	0.3605	0.3502	0.40387	0.39119
0.692	0.24561	0.17776	0.26621	0.692	0.28703	0.18324	0.34199	0.33969	0.692	0.36173	0.35111	0.40489	0.39151
0.696	0.24535	0.17856	0.26778	0.696	0.28844	0.18531	0.34285	0.33969	0.696	0.36255	0.35171	0.40591	0.39214
0.7	0.2454	0.17968	0.26935	0.7	0.29014	0.18739	0.34456	0.33994	0.7	0.36337	0.35292	0.40692	0.39308
0.704	0.24561	0.18079	0.27118	0.704	0.29184	0.18982	0.34627	0.33994	0.704	0.3646	0.35414	0.40794	0.39403
0.708	0.24566	0.18191	0.27301	0.708	0.29382	0.19189	0.34755	0.33994	0.708	0.36542	0.35504	0.40895	0.39497
0.712	0.24644	0.18334	0.27484	0.712	0.29552	0.19501	0.34968	0.34044	0.712	0.36665	0.35626	0.40997	0.39591
0.716	0.24697	0.18493	0.2772	0.716	0.2975	0.19813	0.35182	0.34069	0.716	0.36747	0.35777	0.4115	0.39749
0.72	0.24801	0.18668	0.27955	0.72	0.29976	0.20159	0.35396	0.34119	0.72	0.3687	0.35898	0.41302	0.39906
0.724	0.24879	0.18844	0.2819	0.724	0.30231	0.20506	0.35609	0.34144	0.724	0.36993	0.3611	0.41861	0.40095
0.728	0.25025	0.19035	0.28478	0.728	0.30457	0.20956	0.35823	0.34194	0.728	0.37116	0.36322	0.41963	0.40284
0.732	0.25181	0.19258	0.28792	0.732	0.30768	0.21406	0.36079	0.34294	0.732	0.37239	0.36565	0.42115	0.40536

0.736	0.25369	0.19513	0.29106	0.736	0.31079	0.21926	0.36336	0.34369	0.736	0.37403	0.36837	0.42318	0.40756
0.74	0.2554	0.19767	0.29446	0.74	0.31391	0.22445	0.36549	0.34469	0.74	0.37526	0.3711	0.42471	0.41008
0.744	0.2577	0.20054	0.29812	0.744	0.3173	0.23034	0.36848	0.34594	0.744	0.37731	0.37413	0.42623	0.41323
0.748	0.2602	0.20389	0.3023	0.748	0.32126	0.23692	0.37147	0.34719	0.748	0.37936	0.37807	0.42877	0.41669
0.752	0.26285	0.20707	0.30649	0.752	0.32522	0.24385	0.37447	0.34844	0.752	0.38141	0.38201	0.43081	0.42015
0.756	0.26567	0.21058	0.31093	0.756	0.32975	0.25078	0.37703	0.34994	0.756	0.38345	0.38625	0.43335	0.42362
0.76	0.26869	0.21424	0.31564	0.76	0.33428	0.25875	0.38045	0.35169	0.76	0.38591	0.39079	0.43589	0.42771
0.764	0.27218	0.21854	0.32087	0.764	0.33909	0.26671	0.38344	0.35344	0.764	0.38837	0.39624	0.43894	0.43243
0.768	0.27588	0.22284	0.3261	0.768	0.34418	0.27572	0.38686	0.35569	0.768	0.39124	0.402	0.44199	0.43684
0.772	0.27905	0.22714	0.33133	0.772	0.34927	0.28438	0.38985	0.35769	0.772	0.39411	0.40715	0.44504	0.44156
0.776	0.28374	0.2324	0.33787	0.776	0.35578	0.29442	0.39412	0.36045	0.776	0.39821	0.41381	0.4491	0.44754
0.78	0.28843	0.23718	0.34388	0.78	0.36172	0.30412	0.39754	0.3627	0.78	0.40108	0.42018	0.45266	0.45258
0.784	0.2926	0.24212	0.35016	0.784	0.36738	0.31347	0.40053	0.36545	0.784	0.40477	0.42593	0.45622	0.45824
0.788	0.29677	0.24721	0.35643	0.788	0.3736	0.32317	0.40352	0.3677	0.788	0.40846	0.43229	0.45978	0.46359
0.792	0.30093	0.25231	0.36271	0.792	0.37954	0.33287	0.40651	0.37045	0.792	0.41215	0.43865	0.46435	0.46926
0.796	0.30614	0.25757	0.36925	0.796	0.38577	0.34292	0.40993	0.3732	0.796	0.41625	0.44593	0.46841	0.47556
0.8	0.31083	0.26282	0.37578	0.8	0.39228	0.35296	0.41249	0.3762	0.8	0.42035	0.45259	0.47299	0.48154
0.804	0.31552	0.26792	0.38232	0.804	0.39822	0.36266	0.41506	0.37896	0.804	0.42445	0.45925	0.47705	0.48752
0.808	0.32021	0.27318	0.38886	0.808	0.40472	0.37236	0.41762	0.38196	0.808	0.42896	0.46622	0.48163	0.49381
0.812	0.32542	0.27843	0.3954	0.812	0.41123	0.38206	0.42061	0.38496	0.812	0.43347	0.47289	0.48671	0.50011
0.816	0.3301	0.28353	0.40167	0.816	0.41717	0.39141	0.42275	0.38796	0.816	0.43798	0.47985	0.49179	0.50609
0.82	0.33479	0.28831	0.40795	0.82	0.4234	0.40042	0.42488	0.39071	0.82	0.4429	0.48682	0.49637	0.51207
0.824	0.33948	0.29293	0.4137	0.824	0.42906	0.40873	0.42702	0.39346	0.824	0.447	0.49288	0.50145	0.51774
0.828	0.34417	0.29755	0.41998	0.828	0.43556	0.41773	0.42916	0.39646	0.828	0.4515	0.49985	0.50653	0.52403
0.832	0.34938	0.30248	0.42573	0.832	0.44122	0.42639	0.43129	0.39946	0.832	0.45642	0.50651	0.51212	0.53033
0.836	0.35354	0.30663	0.43149	0.836	0.44688	0.43401	0.433	0.40247	0.836	0.46134	0.51257	0.51771	0.53568
0.84	0.35875	0.31109	0.4375	0.84	0.45282	0.44198	0.43514	0.40547	0.84	0.46585	0.51924	0.5233	0.54198
0.844	0.36292	0.31555	0.44299	0.844	0.45876	0.4496	0.43728	0.40847	0.844	0.47118	0.5253	0.52889	0.54796
0.848	0.36813	0.31969	0.44848	0.848	0.46414	0.45688	0.43941	0.41147	0.848	0.4761	0.53135	0.53448	0.55362
0.852	0.3723	0.32351	0.45371	0.852	0.46951	0.46415	0.44155	0.41447	0.852	0.48102	0.53711	0.54007	0.55929
0.856	0.37699	0.32733	0.45894	0.856	0.47517	0.47038	0.44368	0.41722	0.856	0.48594	0.54287	0.54617	0.56464
0.86	0.38167	0.33163	0.46417	0.86	0.48083	0.47697	0.44582	0.42022	0.86	0.49086	0.54862	0.55227	0.57031
0.864	0.38584	0.3353	0.4694	0.864	0.48621	0.4832	0.44796	0.42323	0.864	0.49619	0.55438	0.55837	0.57598
0.868	0.39001	0.3388	0.47437	0.868	0.4913	0.48874	0.45009	0.42598	0.868	0.50152	0.55953	0.56447	0.58101
0.872	0.39418	0.34231	0.47908	0.872	0.49668	0.49428	0.45266	0.42898	0.872	0.50685	0.56498	0.57056	0.58636
0.876	0.39886	0.34629	0.48457	0.876	0.50262	0.49983	0.45522	0.43223	0.876	0.51259	0.57074	0.57768	0.59171
0.88	0.40303	0.34979	0.48954	0.88	0.50799	0.50468	0.45778	0.43523	0.88	0.51832	0.57589	0.58378	0.59707
0.884	0.40668	0.35314	0.49425	0.884	0.5128	0.50918	0.46035	0.43798	0.884	0.52324	0.58134	0.58988	0.6021
0.888	0.41085	0.35648	0.49922	0.888	0.51818	0.51368	0.46291	0.44073	0.888	0.52898	0.58619	0.59648	0.60714
0.892	0.41501	0.35999	0.50418	0.892	0.52355	0.51818	0.46633	0.44399	0.892	0.53513	0.59164	0.60258	0.61249
0.896	0.41918	0.36365	0.50941	0.896	0.52921	0.52269	0.46932	0.44699	0.896	0.54087	0.59739	0.60868	0.61784
0.9	0.42283	0.367	0.51438	0.9	0.5343	0.5265	0.47274	0.44974	0.9	0.54661	0.60254	0.61478	0.62256
0.904	0.42699	0.37082	0.51961	0.904	0.53996	0.531	0.47659	0.45274	0.904	0.55276	0.6083	0.62088	0.6276
0.908	0.43064	0.37448	0.5251	0.908	0.54562	0.5355	0.48043	0.45599	0.908	0.5585	0.61406	0.62698	0.63295
0.912	0.43429	0.37815	0.53033	0.912	0.55128	0.54035	0.4847	0.45924	0.912	0.56506	0.61951	0.63307	0.63799
0.916	0.43793	0.38213	0.53609	0.916	0.55694	0.5452	0.48898	0.46224	0.916	0.57121	0.62496	0.63866	0.64334
0.92	0.44158	0.38611	0.54158	0.92	0.56288	0.55074	0.49368	0.4655	0.92	0.57736	0.63102	0.64476	0.64869
0.924	0.44575	0.39041	0.54759	0.924	0.5691	0.55663	0.4988	0.46875	0.924	0.58391	0.63708	0.65086	0.65467
0.928	0.44939	0.39471	0.55387	0.928	0.57561	0.56287	0.50479	0.47225	0.928	0.59047	0.64344	0.65594	0.66034
0.932	0.45304	0.39901	0.56015	0.932	0.58212	0.5691	0.50991	0.47575	0.932	0.59662	0.6495	0.66153	0.66601
0.936	0.45721	0.40395	0.56695	0.936	0.58863	0.57707	0.51675	0.4795	0.936	0.60359	0.65647	0.66763	0.6723
0.94	0.46137	0.40905	0.57401	0.94	0.59598	0.58504	0.52359	0.4835	0.94	0.61015	0.66344	0.67322	0.67891
0.944	0.46554	0.41462	0.58133	0.944	0.60362	0.59404	0.53085	0.48751	0.944	0.61712	0.67071	0.67881	0.68584
0.948	0.47023	0.42004	0.58865	0.948	0.61126	0.60339	0.53811	0.49151	0.948	0.62409	0.67798	0.6844	0.69276
0.952	0.47492	0.42577	0.5965	0.952	0.61918	0.61379	0.54623	0.49601	0.952	0.63106	0.68585	0.68999	0.7
0.956	0.48013	0.43198	0.60486	0.956	0.62767	0.62452	0.55478	0.50051	0.956	0.63844	0.69403	0.69609	0.70819
0.96	0.48534	0.43836	0.61349	0.96	0.63616	0.6363	0.56332	0.50527	0.96	0.64581	0.70251	0.70219	0.71637
0.964	0.49002	0.44473	0.62186	0.964	0.64493	0.64773	0.5723	0.51027	0.964	0.65319	0.71069	0.70778	0.72456
0.968	0.49628	0.45142	0.63075	0.968	0.65427	0.6602	0.5817	0.51552	0.968	0.66098	0.71948	0.71439	0.73369
0.972	0.50201	0.45827	0.63991	0.972	0.66388	0.67302	0.59152	0.52102	0.972	0.66836	0.72857	0.72099	0.74313
0.976	0.50826	0.46496	0.64906	0.976	0.6735	0.68549	0.60092	0.52653	0.976	0.67656	0.73705	0.72658	0.75289
0.98	0.51451	0.47165	0.65847	0.98	0.68284	0.69865	0.61032	0.53203	0.98	0.68435	0.74553	0.7337	0.76233
0.984	0.52128	0.47834	0.66736	0.984	0.69218	0.71112	0.61972	0.53778	0.984	0.69214	0.75401	0.7398	0.77209
0.988	0.52753	0.48487	0.67652	0.988	0.70151	0.72324	0.6287	0.54303	0.988	0.70034	0.7625	0.74691	0.78185
0.992	0.53482	0.49124	0.68567	0.992	0.71085	0.73502	0.6381	0.54854	0.992	0.70853	0.77098	0.75352	0.79161
0.996	0.54107	0.49697	0.69378	0.996	0.71934	0.74576	0.64579	0.55379	0.996	0.71591	0.77764	0.76013	0.80074
1	0.54837	0.50255	0.70214	1	0.72754	0.75649	0.65348	0.55854	1	0.72411	0.78491	0.76673	0.81018
1.004	0.55514	0.50781	0.70973	1.004	0.73546	0.76654	0.66117	0.56329	1.004	0.73149	0.79188	0.77334	0.81868
1.008	0.56139	0.51242	0.71679	1.008	0.74282	0.7752	0.66715	0.56754	1.008	0.73887	0.79703	0.77944	0.82686
1.012	0.56764	0.51641	0.72306	1.012	0.74905	0.78247	0.67313	0.57105	1.012	0.74584	0.80158	0.78554	0.83442
1.016	0.57337	0.51991	0.72882	1.016	0.7547	0.78871	0.67783	0.57455	1.016	0.7524	0.80521	0.79113	0.84103
1.02	0.5791	0.52278	0.73405	1.02	0.76008	0.7946	0.68211	0.57705	1.02	0.75896	0.80824	0.79672	0.84701
1.024	0.58483	0.52533	0.73823	1.024	0.76461	0.79875	0.68552	0.5793	1.024	0.7647	0.81036	0.8018	0.85236
1.028	0.589	0.5266	0.74137	1.028	0.76744	0.80118	0.68766	0.58055	1.028	0.76962	0.81097	0.80586	0.85614
1.032	0.59369	0.52788	0.74477	1.032	0.77026	0.80326	0.6898	0.5813	1.032	0.77412	0.81127	0.80993	0.8596
1.036	0.59733	0.52835	0.74712	1.036	0.77196	0.80464	0.69108	0.58155	1.036	0.77822	0.81036	0.81349	0.86212
1.04	0.60098	0.52835	0.74843	1.04	0.77281	0.80464	0.69151	0.58105	1.04	0.7815	0.80885	0.81654	0.8637
1.044	0.60358	0.52772	0.74922	1.044	0.77309	0.80395	0.69108	0.58005	1.044	0.78396	0.80642	0.81857	0.86401
1.048	0.60619	0.52676	0.74922	1.048	0.77225	0.80222	0.69065	0.5783	1.048	0.78601	0.8037	0.8206	0.8637
1.052	0.60775	0.52533	0.74922	1.052	0.77111	0.80014	0.68937	0.57655	1.052	0.78806	0.80067	0.82213	0.86307
1.056	0.60931	0.52357	0.74843	1.056	0.76942	0.79771	0.68809	0.57405	1.056	0.78888	0.79703	0.82314	0.86149
1.06	0.61036	0.5215	0.74686	1.06	0.76687	0.7946	0.68595	0.57105	1.06	0.78929	0.79249	0.82314	0.85897
1.064	0.61088	0.51911	0.74529	1.064	0.76404	0.79113	0.68381	0.56804	1.064	0.78888	0.78855	0.82314	0.85645
1.068	0.6114	0.51688	0.74372	1.068	0.76121	0.78767	0.68168	0.56504	1.068	0.78888	0.7837	0.82314	0.85362
1.072	0.6114	0.51402	0.74189	1.072	0.75782	0.78386	0.67954	0.56129	1.072	0.78765	0.77885	0.82213	0.85016
1.076	0.61088	0.51131	0.73928	1.076	0.75385	0.78005	0.67698	0.55779	1.076	0.78601	0.7737	0.8206	0.84638
1.08	0.61036	0.50844	0.73666	1.08	0.74989	0.77658	0.67441	0.55404	1.08	0.78437	0.76886	0.81959	0.8426
1.084	0.60983	0.50621	0.73509	1.084	0.74678	0.77347	0.67271	0.55054	1.084	0.78314	0.76462	0.81857	0.83883
1.088	0.60931	0.50335	0.73274	1.088	0.7431	0.77035	0.67057	0.54703	1.088	0.78068	0.75977	0.81654	0.83536
1.092	0.60827	0.50032	0.73013	1.092	0.73914	0.76689	0.66843	0.54328	1.092	0.77781	0.75523	0.814	0.83127
1.096	0.60723	0.49793	0.72777	1.096	0.73546	0.76446	0.66715	0.53978	1.096	0.77535	0.75068	0.81247	0.82781
1.1	0.60671	0.49586	0.72594	1.1	0.73235	0.76169	0.66544	0.53628	1.1	0.77249	0.74644	0.81044	0.82403
1.104	0.60567	0.49331	0.72411	1.104	0.72896	0.75927	0.66416	0.53303	1.104	0.77003	0.7422	0.80841	0.82088
1.108	0.60463	0.49108	0.72202	1.108	0.72585	0.75719	0.66288	0.52978	1.108	0.76716	0.73796	0.80586	0.81742
1.112	0.6041	0.48901	0.72019	1.112	0.72302	0.75546	0.66202	0.52703	1.112	0.76429	0.73463	0.80383	0.81459
1.116	0.60306	0.48726	0.71888	1.116	0.72047	0.75407	0.66202	0.52452	1.116	0.76183	0.73129	0.8018	0.81207
1.12	0.60254	0.48582	0.71757	1.12	0.71821	0.75303	0.66202	0.52227	1.12	0.75978	0.72887	0.79977	0.80987
1.124	0.60202	0.48407	0.71627	1.124	0.71623	0.75199	0.6616	0.51977	1.124	0.75732	0.72584	0.79824	0.80798
1.128	0.6015	0.48296	0.71548	1.128	0.71481	0.75165	0.66202	0.51802	1.128	0.75568	0.72372	0.79672	0.8064
1.132	0.6015	0.48168	0.7147	1.132	0.71368	0.75165	0.66331	0.51627	1.132	0.75363	0.7216	0.7957	0.80514
1.136	0.60202	0.4812	0.71417	1.136	0.71255	0.75165	0.66459	0.51477	1.136	0.7524	0.72008	0.79418	0.8042
1.14	0.60202	0.48025	0.71417	1.14	0.7117	0.75234	0.66587	0.51327	1.14	0.75076	0.71827	0.79265	0.80325
1.144	0.60254	0.47993	0.71417	1.144	0.71142	0.75303	0.66758	0.51227	1.144	0.75035	0.71706	0.79214	0.80294

1.148	0.60358	0.48009	0.7147	1.148	0.7117	0.75442	0.67014	0.51177	1.148	0.74994	0.71706	0.79163	0.80325
1.152	0.60515	0.48009	0.71574	1.152	0.71226	0.75546	0.67313	0.51152	1.152	0.74994	0.71645	0.79163	0.80388
1.156	0.60619	0.48009	0.71627	1.156	0.71311	0.75719	0.67612	0.51102	1.156	0.74994	0.71615	0.79113	0.80451
1.16	0.60827	0.48073	0.71783	1.16	0.71425	0.75892	0.67954	0.51102	1.16	0.75035	0.71645	0.79163	0.80577
1.164	0.61036	0.48152	0.7194	1.164	0.71594	0.76169	0.68381	0.51127	1.164	0.75117	0.71706	0.79214	0.80735
1.168	0.61244	0.48264	0.72097	1.168	0.71764	0.76446	0.68809	0.51152	1.168	0.7524	0.71796	0.79316	0.80924
1.172	0.61504	0.48359	0.72306	1.172	0.7199	0.76758	0.69236	0.51227	1.172	0.75363	0.71918	0.79418	0.81112
1.176	0.61817	0.48519	0.72542	1.176	0.72217	0.7707	0.69706	0.51302	1.176	0.75568	0.72008	0.7957	0.81396
1.18	0.62182	0.48694	0.72777	1.18	0.72556	0.77416	0.70261	0.51377	1.18	0.75814	0.72221	0.79773	0.81679
1.184	0.62598	0.48917	0.73143	1.184	0.72952	0.7797	0.70902	0.51552	1.184	0.76183	0.72493	0.80078	0.82088
1.188	0.62963	0.49092	0.73431	1.188	0.73292	0.78317	0.71458	0.51677	1.188	0.76429	0.72645	0.80332	0.82435
1.192	0.6338	0.49347	0.73745	1.192	0.73688	0.78802	0.72099	0.51827	1.192	0.76798	0.72887	0.80586	0.82812
1.196	0.63796	0.4957	0.74111	1.196	0.74084	0.79286	0.7274	0.52002	1.196	0.77167	0.7316	0.80942	0.83253
1.2	0.64265	0.49841	0.74451	1.2	0.74565	0.79806	0.73423	0.52177	1.2	0.77617	0.73402	0.81298	0.83662
1.204	0.64682	0.50112	0.74817	1.204	0.75018	0.80326	0.7415	0.52377	1.204	0.78027	0.73675	0.81654	0.84103
1.208	0.65203	0.50382	0.75235	1.208	0.75527	0.80845	0.74833	0.52602	1.208	0.78519	0.74008	0.8206	0.84544
1.212	0.65724	0.50717	0.75654	1.212	0.76065	0.81434	0.75645	0.52828	1.212	0.79093	0.74371	0.82518	0.85079
1.216	0.66297	0.51051	0.76098	1.216	0.76602	0.82057	0.76414	0.53078	1.216	0.79667	0.74705	0.82975	0.85583
1.22	0.66818	0.5137	0.76569	1.22	0.77168	0.82612	0.77183	0.53328	1.22	0.802	0.75038	0.83432	0.86055
1.224	0.67443	0.51752	0.77066	1.224	0.77762	0.83304	0.78038	0.53578	1.224	0.80856	0.75401	0.83991	0.8659
1.228	0.68068	0.52134	0.77589	1.228	0.78384	0.83963	0.78892	0.53878	1.228	0.81512	0.75795	0.8455	0.87156
1.232	0.68693	0.52533	0.78086	1.232	0.79035	0.84621	0.79747	0.54178	1.232	0.82168	0.76189	0.8516	0.87692
1.236	0.69318	0.52931	0.78609	1.236	0.79686	0.85244	0.80602	0.54453	1.236	0.82865	0.76553	0.8577	0.88195
1.24	0.69943	0.53377	0.79184	1.24	0.80393	0.86006	0.81542	0.54754	1.24	0.83562	0.76946	0.8638	0.88793
1.244	0.70673	0.53839	0.79759	1.244	0.81157	0.86768	0.82524	0.55079	1.244	0.84381	0.77401	0.87092	0.8936
1.248	0.71402	0.54349	0.80387	1.248	0.81978	0.87565	0.8355	0.55404	1.248	0.85242	0.77916	0.87854	0.8999
1.252	0.72131	0.54795	0.81015	1.252	0.8277	0.88327	0.84575	0.55729	1.252	0.86103	0.7834	0.88515	0.90588
1.256	0.72965	0.55336	0.81695	1.256	0.83619	0.89193	0.85686	0.56079	1.256	0.87005	0.78855	0.89328	0.91249
1.26	0.73798	0.55878	0.82401	1.26	0.84524	0.90024	0.86797	0.56454	1.26	0.87948	0.794	0.90141	0.9191
1.264	0.74631	0.56435	0.83081	1.264	0.85429	0.9089	0.87951	0.56855	1.264	0.88973	0.79945	0.91005	0.92602
1.268	0.75465	0.57009	0.83787	1.268	0.86363	0.91756	0.89104	0.5723	1.268	0.89957	0.8046	0.91818	0.93326
1.272	0.7635	0.57614	0.84545	1.272	0.87353	0.92691	0.90344	0.5763	1.272	0.91063	0.81006	0.92733	0.94082
1.276	0.77288	0.58235	0.85356	1.276	0.884	0.93661	0.91625	0.58055	1.276	0.92211	0.81581	0.93647	0.94837
1.28	0.7833	0.58888	0.86192	1.28	0.89475	0.94666	0.92864	0.58505	1.28	0.934	0.82218	0.94613	0.95656
1.284	0.7932	0.59541	0.86977	1.284	0.90522	0.95636	0.94189	0.58956	1.284	0.94589	0.82823	0.95579	0.96443
1.288	0.80362	0.60274	0.87918	1.288	0.91739	0.96744	0.95642	0.59456	1.288	0.95901	0.8355	0.96697	0.97324
1.292	0.81403	0.61007	0.88781	1.292	0.92899	0.97783	0.97052	0.59931	1.292	0.97253	0.84247	0.97764	0.98206
1.296	0.82549	0.61708	0.89697	1.296	0.94115	0.98892	0.98505	0.60431	1.296	0.98606	0.84944	0.98882	0.99087
1.3	0.83643	0.62456	0.90612	1.3	0.95332	1	1	0.60931	1.3	1	0.85671	1	1

**Water-PBS (Spring)**				**Water-PBS (Summer)**					**Water-PBS (Autumn)**				
0.004	0.03729	0.04268	0.02129	0.004	0.0342	0.04699	0.03315	0.0022	0.004	0.01627	0.02148	0.00325	0.00283
0.008	0.03379	0.03926	0.01858	0.008	0.03101	0.04393	0.03012	0.00195	0.008	0.01404	0.01886	0.00249	0.00217
0.012	0.03091	0.03602	0.01624	0.012	0.02815	0.04092	0.02689	0.00163	0.012	0.01223	0.01659	0.00162	0.00147
0.016	0.02796	0.03312	0.01424	0.016	0.02544	0.03826	0.02462	0.00133	0.016	0.01038	0.01466	9.38E-04	8.83E-04
0.02	0.02565	0.03022	0.01228	0.02	0.02309	0.03566	0.02234	0.00114	0.02	0.00882	0.01273	3.61E-04	4.05E-04
0.024	0.02319	0.02783	0.01047	0.024	0.02074	0.03316	0.02007	7.05E-04	0.024	0.00739	0.0108	7.22E-05	2.21E-04
0.028	0.02126	0.02561	0.009	0.028	0.01875	0.03111	0.01798	5.15E-04	0.028	0.00611	0.00938	7.22E-05	1.10E-04
0.032	0.01923	0.02322	0.00771	0.032	0.01671	0.02891	0.01608	3.25E-04	0.032	0.00494	0.00818	0	0
0.036	0.01723	0.02134	0.00645	0.036	0.0146	0.02652	0.01419	2.17E-04	0.036	0.00369	0.00682	1.08E-04	1.10E-04
0.04	0.01551	0.01946	0.00523	0.04	0.01285	0.02405	0.01248	0	0.04	0.0028	0.0055	5.41E-04	4.05E-04
0.044	0.01382	0.01758	0.00433	0.044	0.01126	0.02163	0.01058	0	0.044	0.00204	0.00436	9.74E-04	8.83E-04
0.048	0.01207	0.01571	0.00346	0.048	0.00972	0.01948	0.00907	1.08E-04	0.048	0.00124	0.00338	0.00184	0.00155
0.052	0.01049	0.014	0.00274	0.052	0.00816	0.01749	0.00755	1.36E-04	0.052	4.78E-04	0.00246	0.00285	0.00247
0.056	0.00895	0.01229	0.00216	0.056	0.00665	0.0155	0.00603	4.07E-04	0.056	1.27E-04	0.00171	0.00415	0.00353
0.06	0.00761	0.01093	0.00156	0.06	0.00548	0.01376	0.00489	7.59E-04	0.06	0	0.0012	0.00585	0.00504
0.064	0.00624	0.00922	9.08E-04	0.064	0.00424	0.01217	0.00376	0.00122	0.064	6.37E-05	7.27E-04	0.00769	0.00662
0.068	0.00477	0.00768	4.83E-04	0.068	0.00298	0.01045	0.00243	0.00187	0.068	2.23E-04	9.48E-05	0.0101	0.00839
0.072	0.00368	0.00615	3.09E-04	0.072	0.00208	0.00889	0.00167	0.00268	0.072	7.64E-04	0	0.01288	0.01071
0.076	0.00267	0.00512	0	0.076	0.00141	0.00739	0.00108	0.00388	0.076	0.00156	0	0.01613	0.0134
0.08	0.00175	0.00376	3.86E-05	0.08	7.53E-04	0.00605	1.71E-04	0.00526	0.08	0.00267	2.21E-04	0.02021	0.01664
0.084	0.00109	0.00273	1.55E-04	0.084	2.71E-04	0.0047	0	0.00696	0.084	0.00417	9.16E-04	0.02479	0.02039
0.088	5.26E-04	0.00188	2.51E-04	0.088	1.20E-04	0.00347	9.48E-05	0.00905	0.088	0.00602	0.00158	0.03024	0.02477
0.092	2.11E-04	0.00119	8.89E-04	0.092	0	0.00253	3.60E-04	0.01163	0.092	0.0086	0.00294	0.03652	0.03003
0.096	0	5.12E-04	0.00178	0.096	7.53E-04	0.00161	8.34E-04	0.01463	0.096	0.01165	0.00464	0.04374	0.03589
0.1	2.46E-04	1.71E-04	0.00276	0.1	0.00144	8.87E-04	0.00186	0.01821	0.1	0.01528	0.00657	0.05222	0.04255
0.104	7.37E-04	0	0.00454	0.104	0.00268	2.42E-04	0.00357	0.02249	0.104	0.0199	0.00938	0.06192	0.05028
0.108	0.00158	1.71E-04	0.00653	0.108	0.00443	5.37E-05	0.00565	0.02748	0.108	0.02538	0.0126	0.07311	0.05907
0.112	0.00298	6.83E-04	0.00904	0.112	0.00671	0	0.00869	0.03336	0.112	0.03143	0.01636	0.08585	0.06916
0.116	0.00484	0.00154	0.01199	0.116	0.00975	1.07E-04	0.01229	0.03997	0.116	0.03872	0.02091	0.10003	0.08046
0.12	0.00754	0.00273	0.01563	0.12	0.01322	6.18E-04	0.01665	0.04786	0.12	0.04706	0.02609	0.11634	0.0933
0.124	0.01077	0.00444	0.02013	0.124	0.01791	0.00134	0.02215	0.05675	0.124	0.05658	0.03232	0.13424	0.10773
0.128	0.01516	0.007	0.02534	0.128	0.0233	0.00258	0.02879	0.06689	0.128	0.06734	0.0393	0.1542	0.12374
0.132	0.02014	0.01007	0.03114	0.132	0.02962	0.00419	0.03619	0.07816	0.132	0.07931	0.0471	0.17574	0.14141
0.136	0.02631	0.014	0.03809	0.136	0.03709	0.00626	0.0451	0.09073	0.136	0.09275	0.05601	0.1992	0.16084
0.14	0.03421	0.01861	0.04601	0.14	0.04603	0.00873	0.05515	0.10469	0.14	0.10752	0.06577	0.22435	0.18255
0.144	0.04326	0.02424	0.05489	0.144	0.05566	0.01201	0.06672	0.11984	0.144	0.12357	0.07639	0.25192	0.20611
0.148	0.05396	0.03107	0.06513	0.148	0.0668	0.01553	0.07924	0.13632	0.148	0.14096	0.08814	0.28007	0.23077
0.152	0.06666	0.03892	0.07633	0.152	0.07944	0.01948	0.09289	0.15383	0.152	0.15974	0.10058	0.3093	0.25764
0.156	0.08182	0.04814	0.0887	0.156	0.09269	0.02408	0.10806	0.17239	0.156	0.1798	0.11414	0.33925	0.28561
0.16	0.09999	0.05872	0.1026	0.16	0.10804	0.02905	0.12418	0.19193	0.16	0.20113	0.12832	0.37029	0.31579
0.164	0.12055	0.07067	0.11728	0.164	0.1243	0.03442	0.14106	0.21244	0.164	0.22342	0.14301	0.40132	0.34634
0.168	0.14535	0.0845	0.1337	0.168	0.14206	0.04014	0.1587	0.23385	0.168	0.24666	0.15871	0.43272	0.37799
0.172	0.17377	0.09969	0.15128	0.172	0.16072	0.04656	0.17634	0.25635	0.172	0.2715	0.17517	0.46447	0.41112
0.176	0.20745	0.11676	0.17021	0.176	0.18059	0.05339	0.19436	0.27993	0.176	0.29761	0.19223	0.49623	0.44424
0.18	0.24604	0.13588	0.19068	0.18	0.20196	0.06091	0.21199	0.30459	0.18	0.32499	0.20992	0.52799	0.47884
0.184	0.2906	0.15739	0.21309	0.184	0.22424	0.06871	0.22906	0.33115	0.184	0.35397	0.22887	0.5601	0.51417
0.188	0.34112	0.18197	0.23762	0.188	0.24772	0.0773	0.24556	0.35906	0.188	0.38517	0.24878	0.59258	0.55061
0.192	0.39691	0.20912	0.26427	0.192	0.27271	0.08671	0.2613	0.38969	0.192	0.4186	0.26994	0.62578	0.58815
0.196	0.4583	0.23916	0.29344	0.196	0.2992	0.09638	0.27553	0.42221	0.196	0.4549	0.29269	0.65934	0.62679
0.2	0.52461	0.27313	0.32608	0.2	0.32779	0.1074	0.28899	0.45852	0.2	0.49438	0.31701	0.69398	0.66654
0.204	0.59373	0.31052	0.36182	0.204	0.3588	0.11976	0.30132	0.49809	0.204	0.53673	0.34323	0.72935	0.70703
0.208	0.66425	0.35183	0.40103	0.208	0.39222	0.13319	0.31289	0.54118	0.208	0.58226	0.37072	0.7658	0.74788
0.212	0.73371	0.39672	0.44371	0.212	0.42834	0.14797	0.32313	0.58725	0.212	0.63034	0.39978	0.80116	0.78837
0.216	0.80002	0.44537	0.48969	0.216	0.46717	0.16436	0.33299	0.63685	0.216	0.68096	0.43042	0.83617	0.82886
0.22	0.86001	0.49727	0.53875	0.22	0.50932	0.18236	0.34266	0.68834	0.22	0.73254	0.46138	0.86973	0.86676
0.224	0.91229	0.55189	0.59032	0.224	0.55387	0.20144	0.35234	0.74091	0.224	0.78349	0.49266	0.90004	0.9021
0.228	0.95404	0.6084	0.64266	0.228	0.60053	0.22105	0.36163	0.79241	0.228	0.83188	0.52267	0.92638	0.93301
0.232	0.98351	0.66559	0.69559	0.232	0.6493	0.24148	0.37168	0.84254	0.232	0.87742	0.5511	0.94767	0.95915
0.236	0.99895	0.72277	0.74639	0.236	0.69867	0.26163	0.38192	0.88889	0.236	0.91785	0.57605	0.96319	0.98013
0.24	1	0.77808	0.79448	0.24	0.74713	0.28151	0.39216	0.92927	0.24	0.95065	0.59754	0.97185	0.99411
0.244	0.98632	0.82998	0.83736	0.244	0.79349	0.30005	0.40222	0.96152	0.244	0.97421	0.61396	0.97257	1
0.248	0.9586	0.8776	0.87426	0.248	0.83744	0.31698	0.41151	0.98509	0.248	0.98726	0.62439	0.96572	0.9989

0.252	0.92001	0.9196	0.90304	0.252	0.87628	0.33149	0.42061	0.99864	0.252	0.98981	0.62913	0.95237	0.99043
0.256	0.87159	0.95408	0.92255	0.256	0.90969	0.34277	0.42839	1	0.256	0.98058	0.62723	0.9318	0.9746
0.26	0.81686	0.97934	0.93143	0.26	0.93468	0.35083	0.43446	0.98943	0.26	0.9602	0.61933	0.90509	0.95179
0.264	0.75932	0.99522	0.92969	0.264	0.95274	0.35513	0.43977	0.96775	0.264	0.93027	0.60575	0.87406	0.92418
0.268	0.70073	1	0.91617	0.268	0.96147	0.35567	0.44204	0.93496	0.268	0.89111	0.58743	0.83869	0.89179
0.272	0.64214	0.993	0.89183	0.272	0.96117	0.35245	0.44204	0.89295	0.272	0.84589	0.56468	0.8008	0.85536
0.276	0.58531	0.97474	0.85745	0.276	0.95093	0.34573	0.43939	0.84363	0.276	0.79591	0.53878	0.76183	0.81708
0.28	0.53058	0.9452	0.81476	0.28	0.93257	0.33552	0.43427	0.7897	0.28	0.74432	0.51129	0.72285	0.77806
0.284	0.479	0.90679	0.76609	0.284	0.90638	0.32316	0.42649	0.7336	0.284	0.69306	0.48286	0.68424	0.73868
0.288	0.42988	0.86019	0.71336	0.288	0.87357	0.30865	0.41606	0.6775	0.288	0.64275	0.45443	0.64743	0.70077
0.292	0.38463	0.80813	0.6587	0.292	0.83564	0.29226	0.40316	0.62275	0.292	0.59468	0.42631	0.61207	0.6636
0.296	0.34288	0.75316	0.605	0.296	0.79379	0.2756	0.38875	0.57153	0.296	0.54978	0.39946	0.57887	0.629
0.3	0.30463	0.69785	0.55304	0.3	0.75014	0.2584	0.37263	0.52438	0.3	0.50839	0.37419	0.54819	0.59625
0.304	0.2699	0.64322	0.50398	0.304	0.70529	0.24094	0.3548	0.48156	0.304	0.4705	0.35018	0.51969	0.5657
0.308	0.23868	0.59167	0.45859	0.308	0.66104	0.22374	0.33641	0.4428	0.308	0.43579	0.32744	0.4937	0.53736
0.312	0.21096	0.54336	0.41667	0.312	0.61769	0.20708	0.31725	0.40839	0.312	0.40459	0.30659	0.46952	0.51086
0.316	0.18675	0.49966	0.37901	0.316	0.57705	0.19096	0.29848	0.3783	0.316	0.37625	0.28732	0.44787	0.4873
0.32	0.165	0.45971	0.34482	0.32	0.53852	0.17565	0.27932	0.35147	0.32	0.3511	0.26994	0.42802	0.46559
0.324	0.1457	0.42352	0.31391	0.324	0.50269	0.16114	0.26073	0.32762	0.324	0.32818	0.25351	0.40998	0.44534
0.328	0.12921	0.39075	0.28649	0.328	0.46928	0.1477	0.24329	0.30649	0.328	0.30748	0.23867	0.39374	0.42768
0.332	0.11458	0.36173	0.26196	0.332	0.43857	0.13507	0.22603	0.28779	0.332	0.28965	0.22571	0.37931	0.41112
0.336	0.10178	0.3351	0.23974	0.336	0.41028	0.12325	0.2101	0.27098	0.336	0.27341	0.21371	0.36559	0.39639
0.34	0.09066	0.3112	0.22004	0.34	0.38409	0.1125	0.1953	0.25608	0.34	0.2594	0.20329	0.35369	0.38351
0.344	0.0808	0.28952	0.20227	0.344	0.36031	0.10256	0.18127	0.2428	0.344	0.24698	0.19349	0.34286	0.371
0.348	0.07238	0.2704	0.18643	0.348	0.33863	0.09396	0.16856	0.23114	0.348	0.23584	0.18496	0.33312	0.36032
0.352	0.06491	0.25265	0.17214	0.352	0.31846	0.08563	0.15699	0.22085	0.352	0.22629	0.17738	0.32446	0.35075
0.356	0.05838	0.2366	0.15958	0.356	0.3001	0.07811	0.14656	0.21136	0.356	0.21769	0.17075	0.31652	0.34229
0.36	0.05301	0.22209	0.14857	0.36	0.28384	0.07166	0.1367	0.20296	0.36	0.21069	0.16506	0.30966	0.33493
0.364	0.04835	0.20946	0.13892	0.364	0.26879	0.06602	0.12855	0.19586	0.364	0.20464	0.16032	0.30389	0.32867
0.368	0.04431	0.19751	0.13042	0.368	0.25494	0.06091	0.12077	0.18935	0.368	0.19954	0.1558	0.29847	0.32278
0.372	0.04045	0.18658	0.12288	0.372	0.2426	0.05635	0.11356	0.18377	0.372	0.19508	0.15201	0.29342	0.31763
0.376	0.03743	0.17685	0.11632	0.376	0.23086	0.05258	0.1075	0.17865	0.376	0.19158	0.14873	0.28909	0.31358
0.38	0.03498	0.16815	0.11072	0.38	0.22063	0.04936	0.10237	0.17431	0.38	0.18872	0.14617	0.28548	0.3099
0.384	0.0328	0.16012	0.10589	0.384	0.21129	0.04638	0.09763	0.17068	0.384	0.18681	0.14405	0.28223	0.30696
0.388	0.03073	0.15278	0.10164	0.388	0.20287	0.0439	0.09327	0.16735	0.388	0.1849	0.14225	0.27935	0.30438
0.392	0.02926	0.1463	0.09816	0.392	0.19564	0.04202	0.08967	0.16461	0.392	0.18394	0.14083	0.27718	0.30254
0.396	0.02789	0.14066	0.09526	0.396	0.18902	0.04041	0.08682	0.16231	0.396	0.1833	0.13979	0.27502	0.30107
0.4	0.02687	0.13571	0.09314	0.4	0.183	0.03936	0.08379	0.16065	0.4	0.18298	0.13912	0.27357	0.29996
0.404	0.0261	0.13093	0.0914	0.404	0.17788	0.03834	0.0817	0.15908	0.404	0.18298	0.13852	0.27213	0.29886
0.408	0.02551	0.12701	0.09024	0.408	0.17367	0.03754	0.08	0.15789	0.408	0.1833	0.13837	0.27141	0.29849
0.412	0.02505	0.12325	0.08966	0.412	0.17005	0.03738	0.07848	0.1571	0.412	0.18426	0.13846	0.27069	0.29886
0.416	0.02466	0.12018	0.08889	0.416	0.16644	0.03711	0.07715	0.15645	0.416	0.1849	0.13875	0.26996	0.29849
0.42	0.02449	0.11728	0.0887	0.42	0.16343	0.03708	0.0762	0.15621	0.42	0.18553	0.13903	0.26996	0.29886
0.424	0.02456	0.11506	0.08908	0.424	0.16132	0.03724	0.07601	0.15618	0.424	0.18681	0.1396	0.26996	0.29923
0.428	0.02463	0.11318	0.08947	0.428	0.15952	0.03754	0.07544	0.1564	0.428	0.18808	0.14039	0.26996	0.29996
0.432	0.0248	0.1113	0.08986	0.432	0.15801	0.03805	0.07544	0.15683	0.432	0.18903	0.14105	0.27033	0.3007
0.436	0.02501	0.10976	0.09063	0.436	0.15681	0.03842	0.07563	0.1574	0.436	0.19031	0.14156	0.27069	0.30144
0.44	0.02537	0.1084	0.09121	0.44	0.15621	0.03901	0.07582	0.15816	0.44	0.19158	0.14222	0.27105	0.30217
0.444	0.02593	0.10737	0.09217	0.444	0.1556	0.03963	0.0762	0.159	0.444	0.19286	0.14326	0.27177	0.30328
0.448	0.02645	0.10635	0.09295	0.448	0.1553	0.04025	0.07696	0.16003	0.448	0.19413	0.14399	0.27285	0.30401
0.452	0.02698	0.10567	0.09372	0.452	0.155	0.04095	0.07753	0.16117	0.452	0.19508	0.14484	0.27357	0.30475
0.456	0.02775	0.10516	0.09468	0.456	0.1553	0.04159	0.07848	0.16252	0.456	0.19636	0.14592	0.2743	0.30548
0.46	0.02842	0.10464	0.09565	0.46	0.1553	0.04232	0.07924	0.16402	0.46	0.19763	0.14683	0.27538	0.30622
0.464	0.02926	0.10413	0.09662	0.464	0.1556	0.0431	0.08019	0.16548	0.464	0.19859	0.14781	0.27646	0.30696
0.468	0.03007	0.10396	0.09758	0.468	0.15621	0.04374	0.08151	0.16705	0.468	0.19986	0.14895	0.2779	0.30769
0.472	0.03094	0.10379	0.09855	0.472	0.15681	0.04463	0.08265	0.16873	0.472	0.20082	0.14999	0.27899	0.30843
0.476	0.032	0.10362	0.09951	0.476	0.15741	0.04533	0.0836	0.17066	0.476	0.20209	0.15116	0.28007	0.30916
0.48	0.03301	0.10362	0.10048	0.48	0.15831	0.0463	0.08474	0.17255	0.48	0.20336	0.15227	0.28151	0.3099
0.484	0.03417	0.10345	0.10144	0.484	0.15892	0.04697	0.08587	0.17448	0.484	0.20432	0.15325	0.28296	0.31064
0.488	0.03529	0.10362	0.10222	0.488	0.16012	0.04777	0.08739	0.17654	0.488	0.20559	0.15464	0.2844	0.31137
0.492	0.03656	0.10379	0.10338	0.492	0.16102	0.04871	0.08853	0.1787	0.492	0.20655	0.15603	0.28584	0.31211
0.496	0.03782	0.10413	0.10454	0.496	0.16223	0.04974	0.09005	0.18095	0.496	0.20782	0.15742	0.28765	0.31321
0.5	0.03933	0.1043	0.10569	0.5	0.16343	0.05065	0.09137	0.18326	0.5	0.20909	0.15881	0.28909	0.31395
0.504	0.04077	0.10481	0.10705	0.504	0.16494	0.05162	0.09289	0.18553	0.504	0.21037	0.16001	0.29053	0.31505
0.508	0.04245	0.1055	0.10859	0.508	0.16614	0.05277	0.0946	0.18814	0.508	0.21164	0.16159	0.29234	0.31616
0.512	0.0441	0.10601	0.10994	0.512	0.16825	0.05393	0.09631	0.19063	0.512	0.21323	0.16317	0.2945	0.31763
0.516	0.04589	0.10652	0.1113	0.516	0.16945	0.05527	0.09782	0.19318	0.516	0.21451	0.16474	0.29595	0.31837
0.52	0.04782	0.10737	0.11303	0.52	0.17126	0.05661	0.09972	0.1958	0.52	0.2161	0.16632	0.29811	0.31984
0.524	0.05003	0.10823	0.11477	0.524	0.17276	0.05823	0.10143	0.19862	0.524	0.21737	0.1679	0.29992	0.32094
0.528	0.05224	0.10925	0.1167	0.528	0.17517	0.06011	0.10313	0.20133	0.528	0.21896	0.1698	0.30208	0.32278
0.532	0.05466	0.11028	0.11844	0.532	0.17698	0.06172	0.10503	0.20431	0.532	0.22056	0.17138	0.30461	0.32425
0.536	0.05726	0.11147	0.12057	0.536	0.17939	0.0636	0.10712	0.2073	0.536	0.22215	0.17359	0.30641	0.32609
0.54	0.05996	0.11301	0.12308	0.54	0.18179	0.06575	0.1092	0.21028	0.54	0.22406	0.1758	0.30894	0.3283
0.544	0.06291	0.11454	0.12559	0.544	0.1845	0.0679	0.11129	0.21353	0.544	0.22597	0.1777	0.31183	0.33088
0.548	0.06592	0.11642	0.1281	0.548	0.18721	0.07032	0.11318	0.21705	0.548	0.2282	0.18022	0.31435	0.33309
0.552	0.06898	0.1183	0.131	0.552	0.19022	0.073	0.11565	0.22058	0.552	0.23043	0.18275	0.31724	0.33566
0.556	0.07269	0.12052	0.13409	0.556	0.19323	0.07596	0.11793	0.22464	0.556	0.23297	0.18559	0.32049	0.33898
0.56	0.0761	0.12291	0.13756	0.56	0.19685	0.07918	0.12039	0.22871	0.56	0.23584	0.18875	0.32373	0.34266
0.564	0.07992	0.12564	0.14104	0.564	0.20076	0.08268	0.12286	0.23304	0.564	0.23902	0.19223	0.32734	0.34634
0.568	0.08385	0.12837	0.1451	0.568	0.20467	0.08617	0.12532	0.23765	0.568	0.24221	0.1957	0.33095	0.35039
0.572	0.08803	0.13179	0.14935	0.572	0.20919	0.0902	0.12817	0.2428	0.572	0.24603	0.19981	0.33528	0.35517
0.576	0.09224	0.13537	0.15379	0.576	0.214	0.0945	0.13082	0.24822	0.576	0.25017	0.20392	0.33961	0.36032
0.58	0.09641	0.13913	0.15862	0.58	0.21912	0.09907	0.13367	0.25391	0.58	0.25431	0.20834	0.3443	0.36511
0.584	0.10104	0.14322	0.16383	0.584	0.22454	0.1039	0.13689	0.26014	0.584	0.25908	0.21339	0.34936	0.371
0.588	0.10557	0.14766	0.16924	0.588	0.23056	0.10874	0.13974	0.26665	0.588	0.26418	0.21908	0.35441	0.37725
0.592	0.11009	0.15227	0.17484	0.592	0.23658	0.11411	0.14296	0.27342	0.592	0.26959	0.22445	0.35982	0.38388
0.596	0.11479	0.15722	0.18102	0.596	0.2432	0.11976	0.14599	0.28074	0.596	0.27564	0.23045	0.36559	0.39087
0.6	0.11939	0.16234	0.18721	0.6	0.25043	0.12594	0.14903	0.28806	0.6	0.28169	0.23646	0.37137	0.39823
0.604	0.1243	0.16798	0.19358	0.604	0.25765	0.13212	0.15244	0.29619	0.604	0.28806	0.24341	0.3775	0.40596
0.608	0.12921	0.17361	0.20034	0.608	0.26548	0.13857	0.15605	0.30432	0.608	0.29506	0.25004	0.38436	0.41443
0.612	0.13378	0.17958	0.20729	0.612	0.27331	0.14501	0.15927	0.31272	0.612	0.30207	0.25762	0.39086	0.42289
0.616	0.13869	0.18573	0.21444	0.616	0.28174	0.152	0.16249	0.32166	0.616	0.30939	0.26489	0.39771	0.43209
0.62	0.1436	0.19205	0.22178	0.62	0.29047	0.15872	0.16591	0.33061	0.62	0.31703	0.27215	0.40493	0.4413
0.624	0.14816	0.1987	0.22912	0.624	0.2992	0.16597	0.16913	0.33982	0.624	0.32467	0.27973	0.41215	0.45086
0.628	0.15307	0.20553	0.23665	0.628	0.30823	0.17269	0.17236	0.34903	0.628	0.33263	0.28795	0.41936	0.4608
0.632	0.15798	0.21236	0.24419	0.632	0.31756	0.17968	0.17577	0.35879	0.632	0.34091	0.29616	0.42694	0.47074
0.636	0.16254	0.21953	0.25191	0.636	0.32719	0.18666	0.1788	0.36855	0.636	0.34951	0.30438	0.43452	0.48105
0.64	0.16711	0.2267	0.25945	0.64	0.33683	0.19365	0.18203	0.37803	0.64	0.3581	0.31259	0.44246	0.49209
0.644	0.17167	0.23387	0.26698	0.644	0.34616	0.2001	0.18525	0.38752	0.644	0.36638	0.3208	0.44968	0.50202
0.648	0.17623	0.24121	0.2747	0.648	0.35609	0.20681	0.1881	0.39754	0.648	0.37498	0.32933	0.45762	0.51233
0.652	0.18044	0.24855	0.28224	0.652	0.36572	0.21326	0.19113	0.40703	0.652	0.38358	0.33755	0.46519	0.52264
0.656	0.185	0.25606	0.28958	0.656	0.37506	0.21944	0.19417	0.41624	0.656	0.39217	0.34607	0.47277	0.53294
0.66	0.18921	0.26323	0.29692	0.66	0.38439	0.22509	0.1972	0.42519	0.66	0.40045	0.35429	0.48035	0.54325

0.664	0.19307	0.27057	0.30387	0.664	0.39372	0.23073	0.20005	0.4344	0.664	0.40873	0.36219	0.48793	0.55282
0.668	0.19728	0.27774	0.31082	0.668	0.40305	0.2361	0.2027	0.44307	0.668	0.41701	0.37008	0.49515	0.56238
0.672	0.20149	0.28508	0.31797	0.672	0.41208	0.24121	0.20592	0.45202	0.672	0.42497	0.37798	0.50272	0.57232
0.676	0.20535	0.29208	0.32415	0.676	0.42021	0.24577	0.20839	0.45988	0.676	0.43261	0.38588	0.50958	0.58079
0.68	0.20921	0.29908	0.33053	0.68	0.42864	0.25007	0.21142	0.46801	0.68	0.43993	0.39283	0.51644	0.58962
0.684	0.21307	0.30574	0.33671	0.684	0.43647	0.2541	0.21408	0.4756	0.684	0.44726	0.40009	0.52329	0.59809
0.688	0.21657	0.31222	0.34231	0.688	0.44369	0.25787	0.21692	0.48291	0.688	0.45426	0.40736	0.52943	0.60582
0.692	0.21973	0.31871	0.34752	0.692	0.45092	0.26109	0.21939	0.48996	0.692	0.46063	0.41336	0.53556	0.61318
0.696	0.22324	0.32468	0.35235	0.696	0.45784	0.26405	0.22205	0.49646	0.696	0.46732	0.41968	0.5417	0.62017
0.7	0.22675	0.33066	0.35737	0.7	0.46416	0.267	0.22489	0.50297	0.7	0.47305	0.42568	0.54711	0.62643
0.704	0.22991	0.33646	0.36182	0.704	0.47018	0.26942	0.22755	0.5092	0.704	0.4791	0.43169	0.55289	0.63268
0.708	0.23306	0.34158	0.36607	0.708	0.4756	0.2713	0.23001	0.51516	0.708	0.48419	0.43737	0.55758	0.63784
0.712	0.23622	0.34705	0.37012	0.712	0.48132	0.27372	0.23286	0.52085	0.712	0.4896	0.44274	0.56263	0.64336
0.716	0.23938	0.35217	0.37418	0.716	0.48674	0.2756	0.23551	0.52627	0.716	0.49502	0.44811	0.56732	0.64777
0.72	0.24254	0.35695	0.37785	0.72	0.49156	0.27748	0.23836	0.53142	0.72	0.49947	0.45317	0.57165	0.65219
0.724	0.24604	0.36139	0.38133	0.724	0.49607	0.27882	0.24101	0.5363	0.724	0.50425	0.45791	0.57598	0.65624
0.728	0.24885	0.36617	0.3848	0.728	0.50059	0.28071	0.24367	0.54118	0.728	0.50871	0.46264	0.57995	0.65992
0.732	0.25236	0.3706	0.38867	0.732	0.5048	0.28259	0.24651	0.54579	0.732	0.51317	0.46738	0.58392	0.66323
0.736	0.25587	0.37504	0.39234	0.736	0.50962	0.28447	0.24973	0.55067	0.736	0.51762	0.47212	0.58789	0.66654
0.74	0.25973	0.37914	0.39581	0.74	0.51323	0.28608	0.25258	0.555	0.74	0.52144	0.47623	0.59114	0.66912
0.744	0.26359	0.38358	0.39968	0.744	0.51775	0.28823	0.2558	0.55934	0.744	0.5259	0.48097	0.59475	0.6717
0.748	0.2678	0.38802	0.40354	0.748	0.52196	0.29065	0.25922	0.56394	0.748	0.53004	0.48571	0.59835	0.67464
0.752	0.27201	0.39263	0.40759	0.752	0.52648	0.29306	0.26244	0.56828	0.752	0.53418	0.49044	0.60196	0.67722
0.756	0.27622	0.39706	0.41184	0.756	0.53069	0.29548	0.26623	0.57262	0.756	0.538	0.49487	0.60521	0.67943
0.76	0.28113	0.40167	0.41609	0.76	0.53521	0.29817	0.26946	0.57695	0.76	0.54246	0.49929	0.60846	0.682
0.764	0.28604	0.40662	0.42092	0.764	0.53972	0.30139	0.27344	0.58156	0.764	0.54692	0.50403	0.61207	0.68458
0.768	0.29095	0.4114	0.42594	0.768	0.54454	0.30489	0.27761	0.5859	0.768	0.55137	0.50877	0.61568	0.68715
0.772	0.29586	0.41635	0.43077	0.772	0.54905	0.30838	0.28179	0.58996	0.772	0.55519	0.5135	0.61856	0.689
0.776	0.30218	0.42233	0.43715	0.776	0.55507	0.31295	0.28653	0.59565	0.776	0.56061	0.51919	0.62289	0.69268
0.78	0.30744	0.42762	0.44275	0.78	0.56049	0.31698	0.29108	0.59999	0.78	0.56507	0.52393	0.6265	0.69562
0.784	0.31305	0.43342	0.44854	0.784	0.56591	0.32128	0.29544	0.60433	0.784	0.56984	0.52898	0.62975	0.69783
0.788	0.31832	0.43889	0.45434	0.788	0.57133	0.32611	0.29999	0.60866	0.788	0.57398	0.53372	0.63336	0.7004
0.792	0.32358	0.44486	0.46052	0.792	0.57705	0.33095	0.30492	0.61273	0.792	0.57876	0.53846	0.63661	0.70298
0.796	0.32884	0.45118	0.4667	0.796	0.58307	0.33606	0.30966	0.61679	0.796	0.58321	0.5432	0.63985	0.70556
0.8	0.3334	0.45749	0.47307	0.8	0.58909	0.34116	0.31441	0.62086	0.8	0.58767	0.54825	0.6431	0.7085
0.804	0.33761	0.46347	0.47926	0.804	0.59511	0.346	0.31877	0.62438	0.804	0.59181	0.55236	0.64563	0.71071
0.808	0.34182	0.47013	0.48563	0.808	0.60113	0.3511	0.32313	0.62763	0.808	0.59595	0.55678	0.64852	0.71329
0.812	0.34568	0.4761	0.49181	0.812	0.60715	0.35594	0.32749	0.63061	0.812	0.59977	0.56089	0.65068	0.7155
0.816	0.34849	0.48225	0.49741	0.816	0.61287	0.36104	0.33147	0.63332	0.816	0.60327	0.56468	0.65285	0.71734
0.82	0.35095	0.48822	0.50301	0.82	0.61829	0.36534	0.33489	0.63522	0.82	0.60614	0.56816	0.65429	0.71881
0.824	0.3527	0.49334	0.50765	0.824	0.62311	0.36937	0.33773	0.63658	0.824	0.60837	0.57037	0.65537	0.71918
0.828	0.3541	0.49915	0.51248	0.828	0.62822	0.3734	0.34058	0.63766	0.828	0.6106	0.57321	0.65609	0.72028
0.832	0.35516	0.50444	0.51673	0.832	0.63304	0.37717	0.34285	0.63847	0.832	0.61251	0.57542	0.65681	0.72102
0.836	0.35551	0.50905	0.5202	0.836	0.63695	0.38012	0.34456	0.6382	0.836	0.61378	0.57669	0.65645	0.72065
0.84	0.35586	0.51349	0.52329	0.84	0.64057	0.38308	0.34589	0.6382	0.84	0.61474	0.57795	0.65609	0.72028
0.844	0.35551	0.51724	0.52581	0.844	0.64358	0.38523	0.34684	0.63739	0.844	0.61505	0.57858	0.65501	0.71918
0.848	0.3548	0.52083	0.52793	0.848	0.64629	0.38684	0.34741	0.6363	0.848	0.61474	0.57985	0.65357	0.7177
0.852	0.35375	0.52339	0.5289	0.852	0.64809	0.38791	0.34741	0.63414	0.852	0.6141	0.57921	0.65176	0.7155
0.856	0.3527	0.52561	0.52967	0.856	0.649	0.38845	0.34722	0.63197	0.856	0.61283	0.57858	0.6496	0.71329
0.86	0.3513	0.52731	0.52986	0.86	0.6499	0.38872	0.34627	0.62953	0.86	0.61187	0.57763	0.64743	0.71034
0.864	0.35024	0.52851	0.52967	0.864	0.6499	0.38872	0.34513	0.62682	0.864	0.61028	0.57637	0.64491	0.7074
0.868	0.34849	0.52902	0.5287	0.868	0.6493	0.38765	0.34361	0.62357	0.868	0.60773	0.57479	0.64166	0.70372
0.872	0.34709	0.52919	0.52754	0.872	0.64809	0.38711	0.3421	0.62031	0.872	0.6055	0.5729	0.63877	0.70004
0.876	0.34603	0.52902	0.52658	0.876	0.64689	0.38576	0.34039	0.61706	0.876	0.60359	0.57068	0.63625	0.69636
0.88	0.34463	0.52868	0.52465	0.88	0.64478	0.38415	0.33849	0.61354	0.88	0.60073	0.56847	0.633	0.69231
0.884	0.34323	0.52731	0.52233	0.884	0.64207	0.38254	0.33622	0.60975	0.884	0.59818	0.56563	0.62939	0.68789
0.888	0.34217	0.52595	0.52001	0.888	0.63906	0.38039	0.33413	0.60595	0.888	0.59499	0.5631	0.62578	0.68347
0.892	0.34112	0.52424	0.51769	0.892	0.63605	0.37824	0.33204	0.60216	0.892	0.59245	0.56057	0.62253	0.67906
0.896	0.34042	0.52253	0.51518	0.896	0.63274	0.37636	0.32996	0.59836	0.896	0.58958	0.55773	0.61928	0.67427
0.9	0.33972	0.52014	0.51209	0.9	0.62883	0.37394	0.32768	0.5943	0.9	0.58608	0.55489	0.61568	0.66949
0.904	0.33902	0.51827	0.50958	0.904	0.62491	0.37179	0.32541	0.59077	0.904	0.58321	0.55205	0.61243	0.66544
0.908	0.33867	0.51571	0.50707	0.908	0.621	0.36964	0.3237	0.58698	0.908	0.58035	0.5492	0.60918	0.66102
0.912	0.33832	0.51349	0.50456	0.912	0.61709	0.36749	0.3218	0.58346	0.912	0.57748	0.54604	0.60593	0.65661
0.916	0.33796	0.51058	0.50185	0.916	0.61287	0.36561	0.3201	0.57993	0.916	0.57462	0.54352	0.60305	0.65256
0.92	0.33796	0.50854	0.49934	0.92	0.60836	0.36346	0.3182	0.57668	0.92	0.57175	0.54067	0.5998	0.64814
0.924	0.33796	0.50632	0.49703	0.924	0.60444	0.36158	0.31706	0.57343	0.924	0.56889	0.53815	0.59727	0.64409
0.928	0.33832	0.50358	0.4949	0.928	0.60053	0.3597	0.31535	0.57018	0.928	0.56634	0.53562	0.59438	0.64041
0.932	0.33832	0.50119	0.49278	0.932	0.59601	0.35782	0.31403	0.56693	0.932	0.56379	0.53278	0.5915	0.63636
0.936	0.33902	0.49881	0.49084	0.936	0.5924	0.35621	0.31308	0.56394	0.936	0.56156	0.53025	0.58897	0.63268
0.94	0.33972	0.49676	0.4893	0.94	0.58849	0.35513	0.31213	0.56096	0.94	0.55933	0.52835	0.58681	0.62937
0.944	0.34042	0.49505	0.48756	0.944	0.58488	0.35379	0.31137	0.55825	0.944	0.55742	0.52614	0.58428	0.62606
0.948	0.34112	0.49317	0.48582	0.948	0.58126	0.35298	0.3108	0.55554	0.948	0.55519	0.52393	0.58212	0.62275
0.952	0.34182	0.49164	0.48447	0.952	0.57765	0.35191	0.31042	0.5531	0.952	0.55328	0.52203	0.57995	0.62017
0.956	0.34323	0.48942	0.4835	0.956	0.57464	0.3511	0.31061	0.55067	0.956	0.55201	0.52014	0.57815	0.61759
0.96	0.34428	0.48822	0.48254	0.96	0.57163	0.35057	0.31061	0.5485	0.96	0.55042	0.51856	0.5767	0.61502
0.964	0.34533	0.48668	0.48157	0.964	0.56862	0.35003	0.31061	0.54606	0.964	0.54915	0.51698	0.5749	0.61244
0.968	0.34709	0.48549	0.48119	0.968	0.56591	0.35003	0.31099	0.54416	0.968	0.54787	0.51572	0.57345	0.6106
0.972	0.34849	0.48447	0.4808	0.972	0.5632	0.35003	0.31156	0.54226	0.972	0.54723	0.51477	0.57201	0.60839
0.976	0.34989	0.48361	0.48061	0.976	0.56109	0.35003	0.31251	0.54037	0.976	0.54628	0.5135	0.57093	0.60655
0.98	0.35165	0.4831	0.48041	0.98	0.55899	0.35057	0.31346	0.53901	0.98	0.54564	0.51287	0.56985	0.60471
0.984	0.3534	0.48225	0.48061	0.984	0.55688	0.3511	0.3146	0.53739	0.984	0.54501	0.51224	0.56912	0.60324
0.988	0.35516	0.48191	0.4808	0.988	0.55537	0.35164	0.31573	0.53603	0.988	0.54469	0.51161	0.5684	0.60177
0.992	0.35726	0.48156	0.48138	0.992	0.55417	0.35245	0.31725	0.53495	0.992	0.54469	0.51161	0.56768	0.60066
0.996	0.35866	0.48139	0.48177	0.996	0.55267	0.35325	0.31877	0.53359	0.996	0.54437	0.51098	0.56696	0.59956
1	0.36112	0.48122	0.48254	1	0.55146	0.3546	0.32066	0.53251	1	0.54469	0.51129	0.56696	0.59845
1.004	0.36322	0.48139	0.4837	1.004	0.55056	0.35594	0.32256	0.53169	1.004	0.54501	0.51161	0.56696	0.59809
1.008	0.36533	0.48208	0.48466	1.008	0.54966	0.35701	0.32465	0.53061	1.008	0.54532	0.51193	0.56696	0.59735
1.012	0.36744	0.48242	0.48582	1.012	0.54905	0.3589	0.32692	0.53007	1.012	0.54596	0.51224	0.56696	0.59698
1.016	0.36989	0.48276	0.48679	1.016	0.54845	0.36051	0.32901	0.52926	1.016	0.5466	0.51287	0.56732	0.59698
1.02	0.37235	0.48361	0.48853	1.02	0.54875	0.36266	0.33185	0.52926	1.02	0.54755	0.51414	0.56804	0.59698
1.024	0.3748	0.48464	0.49007	1.024	0.54875	0.36508	0.33432	0.52898	1.024	0.54851	0.5154	0.56876	0.59698
1.028	0.37726	0.48549	0.49142	1.028	0.54875	0.36722	0.33697	0.52871	1.028	0.54946	0.51603	0.56949	0.59698
1.032	0.37971	0.48703	0.49355	1.032	0.54935	0.36991	0.33963	0.52871	1.032	0.55106	0.51761	0.57021	0.59772
1.036	0.38217	0.48856	0.49548	1.036	0.55026	0.37233	0.34285	0.52898	1.036	0.55233	0.51888	0.57129	0.59809
1.04	0.38498	0.4901	0.4976	1.04	0.55086	0.37529	0.34589	0.52898	1.04	0.55424	0.52045	0.57273	0.59882
1.044	0.38778	0.49129	0.49973	1.044	0.55146	0.37797	0.34892	0.52953	1.044	0.55583	0.52235	0.57382	0.59956
1.048	0.39059	0.493	0.50224	1.048	0.55267	0.38147	0.35253	0.5298	1.048	0.55774	0.52425	0.57526	0.60029
1.052	0.39375	0.49522	0.50475	1.052	0.55387	0.38496	0.35575	0.53061	1.052	0.55965	0.52614	0.57706	0.6014
1.056	0.39691	0.49761	0.50784	1.056	0.55568	0.38845	0.35954	0.53142	1.056	0.56188	0.52835	0.57887	0.60287
1.06	0.39971	0.49932	0.51035	1.06	0.55688	0.39168	0.36296	0.53224	1.06	0.56411	0.53056	0.58031	0.60361
1.064	0.40322	0.50171	0.51364	1.064	0.55869	0.39571	0.36694	0.53332	1.064	0.56634	0.53309	0.58248	0.60508
1.068	0.40673	0.50427	0.51653	1.068	0.56109	0.39974	0.37111	0.53468	1.068	0.5692	0.53593	0.58464	0.60692
1.072	0.41024	0.50734	0.51982	1.072	0.5629	0.40377	0.37547	0.53603	1.072	0.57207	0.53878	0.58681	0.60839

1.076	0.4141	0.5099	0.52329	1.076	0.56531	0.40807	0.37984	0.53711	1.076	0.57462	0.54162	0.58933	0.60986
1.08	0.41796	0.5128	0.52677	1.08	0.56772	0.41237	0.38439	0.53847	1.08	0.57748	0.54478	0.59186	0.61134
1.084	0.42217	0.51639	0.53102	1.084	0.57073	0.4172	0.38913	0.54064	1.084	0.58099	0.54794	0.59511	0.61354
1.088	0.42708	0.51997	0.53488	1.088	0.57374	0.42231	0.39406	0.54226	1.088	0.58417	0.55141	0.59799	0.61575
1.092	0.43129	0.52287	0.53855	1.092	0.57645	0.42714	0.3988	0.54389	1.092	0.58703	0.55489	0.60052	0.61759
1.096	0.4362	0.5268	0.543	1.096	0.57976	0.43198	0.40411	0.54606	1.096	0.59054	0.55868	0.60413	0.62017
1.1	0.44111	0.53056	0.54744	1.1	0.58337	0.43762	0.40961	0.54796	1.1	0.59436	0.56279	0.60738	0.62275
1.104	0.44602	0.53465	0.55207	1.104	0.58668	0.443	0.41492	0.55039	1.104	0.59786	0.56658	0.61098	0.62532
1.108	0.45093	0.53875	0.55652	1.108	0.5906	0.44864	0.42061	0.55256	1.108	0.60168	0.57068	0.61423	0.62753
1.112	0.45655	0.54302	0.56115	1.112	0.59451	0.45428	0.42649	0.555	1.112	0.60582	0.57511	0.6182	0.63047
1.116	0.46216	0.5478	0.56617	1.116	0.59902	0.46046	0.43275	0.55771	1.116	0.60996	0.58016	0.62217	0.63342
1.12	0.46813	0.55258	0.57158	1.12	0.60324	0.46691	0.4392	0.56042	1.12	0.61442	0.5849	0.62614	0.63673
1.124	0.47374	0.5577	0.5768	1.124	0.60775	0.47309	0.44565	0.56313	1.124	0.61856	0.58964	0.63011	0.63968
1.128	0.48005	0.56282	0.58221	1.128	0.61287	0.47954	0.45209	0.56638	1.128	0.62365	0.59501	0.63444	0.64336
1.132	0.48637	0.56811	0.58819	1.132	0.61769	0.48626	0.45873	0.56964	1.132	0.62875	0.60038	0.63913	0.64704
1.136	0.49304	0.57357	0.59399	1.136	0.62281	0.49324	0.46575	0.57289	1.136	0.63384	0.60607	0.64382	0.65072
1.14	0.4997	0.57887	0.59998	1.14	0.62822	0.50023	0.47277	0.57641	1.14	0.63862	0.61144	0.64852	0.6544
1.144	0.50672	0.58467	0.60616	1.144	0.63394	0.50748	0.47997	0.58021	1.144	0.64403	0.61744	0.65357	0.65845
1.148	0.51374	0.59116	0.61273	1.148	0.64027	0.51528	0.48794	0.584	1.148	0.64976	0.62344	0.65934	0.66286
1.152	0.52145	0.59799	0.61929	1.152	0.64659	0.52307	0.4959	0.58806	1.152	0.65549	0.62976	0.66475	0.66728
1.156	0.52882	0.6043	0.62586	1.156	0.65261	0.53086	0.50406	0.59213	1.156	0.6609	0.63608	0.67053	0.67206
1.16	0.53689	0.6113	0.6332	1.16	0.65953	0.53946	0.5124	0.59647	1.16	0.66727	0.64334	0.67666	0.67722
1.164	0.54531	0.61796	0.64073	1.164	0.66646	0.54779	0.52132	0.6008	1.164	0.67364	0.64998	0.68244	0.68237
1.168	0.55373	0.62547	0.64807	1.168	0.67368	0.55665	0.53023	0.60541	1.168	0.68001	0.65693	0.68893	0.68789
1.172	0.5625	0.63298	0.65561	1.172	0.68091	0.56525	0.53933	0.61002	1.172	0.68669	0.66451	0.69507	0.69341
1.176	0.57127	0.64066	0.66333	1.176	0.68813	0.57439	0.54882	0.61462	1.176	0.69306	0.67209	0.70156	0.6993
1.18	0.58004	0.64971	0.67144	1.18	0.69596	0.58352	0.55906	0.61977	1.18	0.70039	0.67999	0.70878	0.70519
1.184	0.59022	0.65756	0.68052	1.184	0.70469	0.59373	0.56968	0.62574	1.184	0.70866	0.68883	0.71636	0.71255
1.188	0.59934	0.66559	0.68883	1.188	0.71281	0.60368	0.57973	0.63061	1.188	0.71567	0.69673	0.72285	0.71844
1.192	0.60881	0.67429	0.69752	1.192	0.72154	0.61389	0.59035	0.6363	1.192	0.72331	0.70526	0.73043	0.72506
1.196	0.61864	0.68334	0.70641	1.196	0.73027	0.6241	0.60135	0.642	1.196	0.73159	0.71379	0.73801	0.73169
1.2	0.62881	0.69273	0.71548	1.2	0.73931	0.63458	0.61235	0.64796	1.2	0.73923	0.72263	0.74595	0.73868
1.204	0.63899	0.70178	0.72437	1.204	0.74804	0.64505	0.62335	0.65338	1.204	0.74719	0.73148	0.75353	0.74568
1.208	0.64951	0.71082	0.73383	1.208	0.75707	0.65634	0.6353	0.65961	1.208	0.75547	0.74064	0.76183	0.75304
1.212	0.66074	0.72055	0.74349	1.212	0.7667	0.66789	0.64744	0.66612	1.212	0.76375	0.75012	0.77049	0.76077
1.216	0.67196	0.73045	0.75334	1.216	0.77603	0.67945	0.65976	0.67262	1.216	0.77234	0.7596	0.77915	0.76886
1.22	0.68249	0.74018	0.76261	1.22	0.78566	0.69127	0.67228	0.67885	1.22	0.78094	0.76939	0.78745	0.77659
1.224	0.69442	0.75009	0.77304	1.224	0.7956	0.70363	0.68518	0.6859	1.224	0.79049	0.7795	0.79647	0.78469
1.228	0.70565	0.76118	0.78328	1.228	0.80553	0.71653	0.69826	0.69267	1.228	0.79941	0.78961	0.80549	0.79315
1.232	0.71792	0.77177	0.7939	1.232	0.81577	0.72889	0.71173	0.69945	1.232	0.80896	0.79972	0.81451	0.80236
1.236	0.7295	0.78218	0.80414	1.236	0.8257	0.74152	0.72538	0.70595	1.236	0.81787	0.81014	0.82354	0.81082
1.24	0.74178	0.79379	0.81457	1.24	0.83594	0.75495	0.73923	0.713	1.24	0.82775	0.82057	0.83328	0.81929
1.244	0.75406	0.80522	0.82539	1.244	0.84617	0.76892	0.75364	0.72032	1.244	0.83793	0.83099	0.84338	0.82849
1.248	0.76704	0.81683	0.8362	1.248	0.85671	0.78316	0.76881	0.72763	1.248	0.84812	0.84236	0.85349	0.83806
1.252	0.78002	0.82827	0.84741	1.252	0.86694	0.79714	0.78418	0.73495	1.252	0.85831	0.85279	0.86323	0.84689
1.256	0.79336	0.84005	0.85919	1.256	0.87748	0.81165	0.79954	0.74254	1.256	0.8685	0.86448	0.8737	0.85646
1.26	0.80704	0.85268	0.87097	1.26	0.88802	0.82669	0.81585	0.7504	1.26	0.87964	0.87585	0.88452	0.8664
1.264	0.82037	0.86497	0.88295	1.264	0.89885	0.84228	0.83197	0.7588	1.264	0.89079	0.88722	0.89571	0.87597
1.268	0.83405	0.8776	0.89473	1.268	0.90969	0.85786	0.84885	0.76639	1.268	0.90193	0.89923	0.90617	0.8859
1.272	0.84809	0.89024	0.90709	1.272	0.92053	0.87371	0.86554	0.77452	1.272	0.91308	0.91091	0.91736	0.89621
1.276	0.86282	0.90355	0.91965	1.276	0.93136	0.89064	0.88317	0.78292	1.276	0.92454	0.92323	0.92855	0.90688
1.28	0.87756	0.91704	0.9322	1.28	0.9431	0.90784	0.90176	0.79159	1.28	0.93632	0.93556	0.93974	0.91792
1.284	0.89229	0.93018	0.94495	1.284	0.95364	0.92503	0.91978	0.79972	1.284	0.9481	0.94788	0.95092	0.92823
1.288	0.90843	0.94486	0.95866	1.288	0.96568	0.94384	0.9395	0.80921	1.288	0.96084	0.96114	0.96319	0.93964
1.292	0.92422	0.95954	0.97257	1.292	0.97742	0.96238	0.95904	0.81815	1.292	0.97357	0.9741	0.9751	0.95068
1.296	0.94071	0.97371	0.98609	1.296	0.98886	0.98119	0.97952	0.8271	1.296	0.98663	0.98705	0.98773	0.96172
1.3	0.95685	0.9889	1	1.3	1	1	1	0.83604	1.3	1	1	1	0.97276
								--					
								--					
								--					
								--					
								--					
								--					
								--					
								--					
								--					
								--					
								--					
